# Mitomycin C-induced DNA double-strand breaks are enhanced by catalytical inactivation of DNA polymerase κ in mice

**DOI:** 10.1186/s41021-025-00343-x

**Published:** 2025-11-06

**Authors:** Naoko A. Wada, Akira Takeiri, Shigeki Motoyama, Kaori Matsuzaki, Kenji Tanaka, Saori Matsuo, Etsuko Fujii-Takeiri, Hiromi Tateishi, Kaoru Matsumoto, Naoko Niimi, Akira Sassa, Petr Grúz, Kenichi Masumura, Masayuki Mishima, Kou-Ichi Jishage, Kei-Ichi Sugiyama, Takehiko Nohmi

**Affiliations:** 1 Research Division, Chugai Life Science Park Yokohama, Chugai Pharmaceutical Co., Ltd., 216 Totsuka-Cho, Totsuka-Ku, Yokohama, Kanagawa 244-8602 Japan; 2 Translational Research Division, Chugai Life Science Park Yokohama, Chugai Pharmaceutical Co., Ltd., 216 Totsuka-Cho, Totsuka-Ku, Yokohama, Kanagawa 244-8602 Japan; 3 Chugai Research Institute for Medical Science, Inc., 216 Totsuka-Cho, Totsuka-Ku, Yokohama, Kanagawa 244-8602 Japan; 4 Diabetic Neuropathy Project, Tokyo Metropolitan Institute of Medical Science, 2-1-6 Kamikitazawa, Setagaya-Ku, Tokyo, 156-8506 Japan; 5 Department of Biology, Graduate School of Science, Chiba University, 1-33 Yayoi-Cho, Inage-Ku, Chiba, Chiba, 263-8522 Japan; 6 Division of Genome Safety Science, National Institute of Health Sciences, 3-25-26, Tonomachi, Kawasaki-Ku, Kawasaki, Kanagawa 210-9501 Japan; 7 Division of Risk Assessment, National Institute of Health Sciences, 3-25-26, Tonomachi, Kawasaki-Ku, Kawasaki, Kanagawa 210-9501 Japan; 8 Division of Pathology, National Institute of Health Sciences, 3-25-26, Tonomachi, Kawasaki-Ku, Kawasaki, Kanagawa 210-9501 Japan

**Keywords:** Translesion DNA synthesis, DNA polymerase κ, Mitomycin C, DNA double-strand breaks, *gpt* delta mice

## Abstract

**Background:**

DNA polymerase κ (Polk), a member of Y-family DNA polymerases, plays an important role in translesion DNA synthesis (TLS), allowing DNA replication forks to bypass DNA damage or DNA adducts to continue daughter strand synthesis. Polk is also believed to contribute to the replication-independent repair of DNA lesions such as cross-links. TLS circumvents stalls of DNA replication and promotes gap filling in DNA repair which would otherwise result in DNA double-strand breaks (DSBs) and cell death. Mitomycin C (MMC) is a widely used chemotherapeutic drug which generates DNA cross-links and induces DSBs. To clarify how Polk contributes to the prevention of MMC-induced DSB in various organs or tissues, immunohistochemical staining of γH2AX was conducted in catalytically inactivated Polk knock-in (Polk KI) mice and Polk wild-type (Polk^+^) mice treated with MMC or saline.

**Results:**

The γH2AX induction by MMC was enhanced by inactivation of Polk across many organs or tissues to varying degrees. Obvious enhancement was observed in liver, bladder, adrenal cortex, thyroid, and spermatids, whereas less enhancement was shown in brain and retina. The results suggest that Polk plays a role in preventing DSBs caused by MMC in most organs or tissues. Elevated DSB frequencies were observed in both proliferative cells, such as bladder epithelium cells, and less or slowly proliferative cells, such as hepatocytes. Increased DSB levels in inactivated Polk KI mice relative to Polk^+^ mice were also observed in saline-treated mice in the adrenal cortex and other tissues.

**Conclusion:**

Polk plays a systemic role in mitigating MMC-induced DSBs, likely through both DNA replication-dependent and -independent mechanisms. Furthermore, Polk appears to protect against DSBs caused by endogenous mutagens in some organs such as the adrenal cortex, prostate, and retina.

**Supplementary Information:**

The online version contains supplementary material available at 10.1186/s41021-025-00343-x.

## Introduction

Humans are continuously exposed to a variety of exogenous and endogenous genotoxins, including polycyclic aromatic hydrocarbons [1], UV irradiation [2] and reactive oxygen species (ROS) [3]. These agents generate DNA adducts, cross-links, or modulated nucleotides and are believed to insult genome integrity, potentially leading to cancer. In addition, anti-cancer chemotherapy and radiotherapy, while targeting the DNA of cancer cells, unintentionally damage the DNA of normal cells [4]. To remove the DNA lesions induced by such genotoxins, cells employ several DNA repair systems, including base excision repair (BER), nucleotide excision repair (NER), and the Fanconi anemia (FA) pathways [5, 6]. However, despite these repair mechanisms, not all the lesions are removed before DNA replication starts. Consequently, replicative DNA polymerases (Pols), such as Pol α, δ, and 𝜀, inevitably encounter DNA lesions, potentially leading to stalled replication forks and DNA strand breaks (DSBs). To overcome this lethal situation, cells possess multiple specialized Pols that can insert dNTPs opposite the lesions in template DNA and continue primer extension, allowing replication to proceed without DSBs [7]. This process is called translesion DNA synthesis (TLS). In mammals, several Y-family Pols (Pol κ (Polk), REV1, Pol, η Pol ι) and B-family Pol ζ are typical examples of TLS Pols. [8–11]. TLS Pols work in cooperation with DNA repair proteins from pathways such as BER, NER, homologous recombination, and FA [5, 6].

Among the TLS Pols, Polk was initially characterized by its ability to bypass a variety of structurally unrelated DNA lesions in vitro. The examples are *N*^2^-guanine adducts induced by benzo[*a*]pyrene [12, 13], abasic DNA lesion [14], thymidine glycol [15] and interstrand cross-links [16]. There are no reports suggesting a relation between Polk deficiency and human disease. Moreover, differences between Polk KI or KO mice and the wild type mice in general conditions such as appearance, body weight, and behavior have not been reported. However, Polk KO mice exhibited increases in spontaneous mutation frequencies of base substitutions mainly at G:C base pairs in kidney, liver, and lung in old age [17]. Mice expressing inactivated Polk (inactivated Polk KI mice) also exhibited the mutator phenotype [18]. More recently, it was reported that mutation frequency in colons exhibiting dextran-induced inflammation was strongly enhanced by inactivation of Polk in mice [19, 20]. These findings suggest that Polk may protect DNA from endogenous and inflammation-induced mutagens.

In addition to the point mutations, Polk appears to protect the genome against double-strand breaks in DNA (DSBs). Polk and other TLS Pols are believed to contribute to the repair of interstrand cross-links by traversing unhooked interstrand cross-links during parental DNA strands replication [16, 21]. Polk is also suggested to be involved in the replication-independent repair of interstrand crosslinks [22]. Polk may be involved in the repair of interstrand cross-links generated by lipid peroxidation products such as malondialdehyde [23]. Micronucleated erythrocytes and γH2AX-positive cells in the bone marrow are more frequently observed in inactivated Polk KI mice than in Polk^+^ mice when treated with mitomycin C (MMC) [24], a crosslinking agent widely used as a first-line chemotherapeutic drug. MMC-induced loss of heterozygosity and sister chromatid exchanges are also enhanced by the knockout of Polk in human cells [25].

In the present study, to clarify how Polk contributes to the prevention of MMC-induced DSB in various organs or tissues, we systemically examined the protective role of Polk against MMC-induced DSBs using γH2AX as a marker of DSBs. Inactivated Polk KI mice and Polk^+^ mice were treated with MMC, and DSBs were evaluated quantitatively and semi-quantitatively in the liver, bladder, adrenal cortex, thyroid, spermatids, and other tissues. Our results suggest that Polk systemically protects against MMC-induced DSBs. In addition, Polk may also protect against endogenously generated DSBs in some organs. The function of Polk in mitigating MMC-induced DSBs across various tissues and organs is discussed.

## Materials and methods

### Chemical treatment

**Fig. 1:**
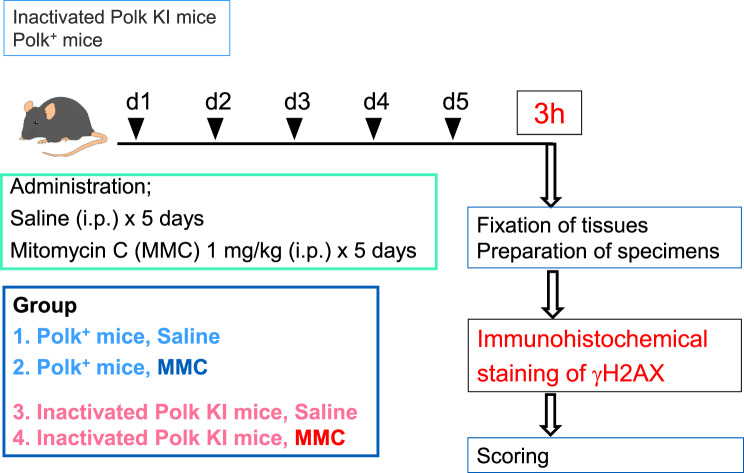
An experimental scheme is illustrated. Saline or mitomycin C (MMC) was administered to 6 Polk^+^ mice (3 male and 3 female), and 6 inactivated Polk KI mice (3 male and 3 female) at a dose of 1 mg/kg/day for 5 days. Organs or tissues were collected 3 h after the last administration. γH2AX was immunohistochemically stained, and each specimen was evaluated

All the organs or tissues evaluated in this study were obtained from the previous study [24]. The experimental scheme is illustrated in Fig. 1. Nine- to 12-week-old *gpt* delta mice and inactivated Polk KI *gpt* delta mice were used as Polk^+^ and inactivated Polk KI mice, respectively. The formal name of inactivated Polk KI mice is C57BL6-*Polk*^*tm3(mPolk*)*Csk*^ *Tg(gptdelta)1 Nmi*. Saline or mitomycin C (MMC, Kyowa Hakko Kirin, Japan) was intraperitoneally injected into 6 Polk^+^ mice (3 males and 3 females) and 6 inactivated Polk KI mice (3 males and 3 females) at a dose of 1 mg/kg/day for 5 days. Organs or tissues were collected 3 h after the last administration. All animal care and experiment procedures in the study were conducted in compliance with the internal regulations for animal use at Chugai Pharmaceutical Co., Ltd., which have been approved by the Association for Assessment and Accreditation of Laboratory Animal Care (AAALAC) International.

### Histopathological evaluation

The organs and tissues were fixed with 4% paraformaldehyde and embedded in paraffin. Prepared section slides from the paraffin blocks were microwaved at 98 °C for 20 minutes in an antigen retrieval reagent (Target Retrieval Solution, DAKO S1699, Agilent Technologies, USA). After immersion in a blocking solution (Protein Block, DAKO X0909, Agilent Technologies, USA), the slides were immunohistochemically stained by anti-γH2AX (Ser139) mouse monoclonal antibody (ab26350, Abcam, UK) and secondary antibody (EnVision+Kit/HRP, DAKO K4001, Agilent Technologies, USA). The slides were stained with Mayer’s Hematoxylin to visualize nuclei. Hematoxylin-eosin (HE)-stained specimens were also prepared.

### Semi-quantitative evaluation of DSBs by γH2AX staining

**Fig. 2:**
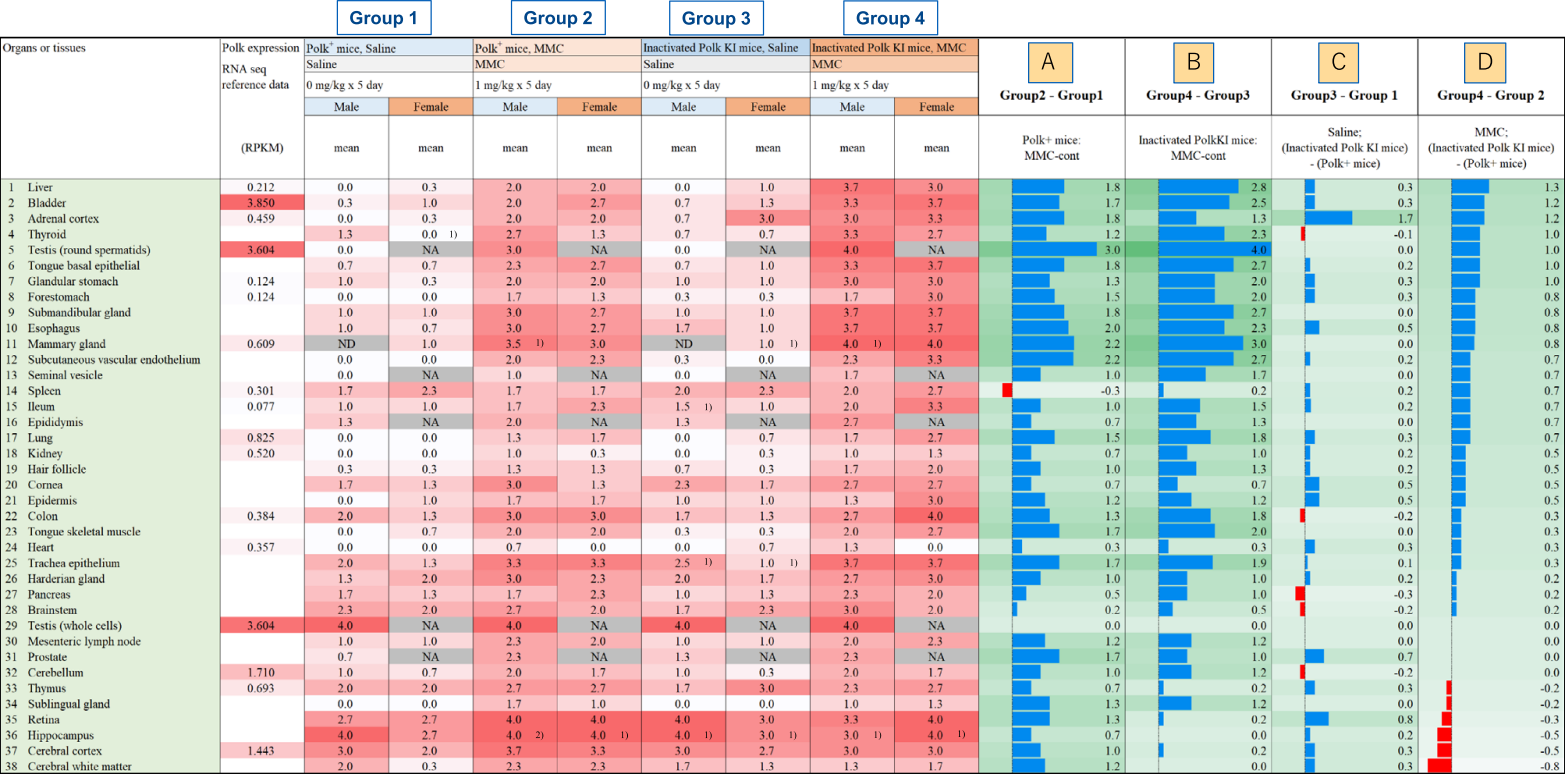
The mean γH2AX staining score of three mice in each group is presented. The score was evaluated semi-quantitatively according to the criteria (see text). The difference of mean score was calculated to elucidate the following items: group comparison A=(mean score of group 2)-(mean score of group 1), increase in score by MMC-treatment from control level in Polk^+^ mice; group comparison B=(mean score of group 4)-(mean score of group 3), increase in score by MMC-treatment from control level in inactivated Polk KI mice; group comparison C=(mean score of group 3)-(mean score of group 1), increase in score by Polk-inactivation in saline-treated mice (Polk effects on intrinsic mutagens); group comparison D=(mean score of group 4)-(mean score of group 2), increase in score by Polk-inactivation in MMC-treated mice (Polk effects on MMC). NA means “not applicable”. ND means “not done” due to missing samples. 1) the average values from 2 animals are shown due to a missing sample from 1 animal. 2) a single value from 1 animal is shown due to missing samples from 2 animals

The level of γH2AX staining in various organs or tissues was semi-quantitatively scored according to the following criteria: score 0, there were no stained cells or the staining was too faint to count the cells; score 1, the frequency of stained cells was less than approx. 5%; score 2, the frequency of stained cells was approx. 5% to 30%; score 3, the frequency of stained cells was approx. 30% to 60%; score 4, the frequency of stained cells was approx. 60% or more. The frequency was arbitrarily determined under microscopic observation (Olympus, Japan). The mice were classified into four groups, i.e., Group 1 as saline-treated Polk^+^ mice; Group 2 as MMC-treated Polk^+^ mice; Group 3 as saline-treated inactivated Polk KI mice; Group 4 as MMC-treated inactivated Polk KI mice. The mean scores from three male or three female mice in each group were calculated and the mean scores were compared among the four groups (Fig. 2). In addition, the differences in the mean scores between two groups, such as Group 1 versus Group 2, were calculated and presented as Group comparisons (Fig. 2).

### Quantitative evaluation of DSBs by γH2AX staining

Numbers of γH2AX-stained cells were quantitatively counted in liver, bladder, adrenal cortex, thyroid, and testis (spermatids), as described below. These were the top 5 organs or tissues that showed the most obvious effects of Polk inactivation on MMC-induced DSBs in the semi-quantitative evaluation of γH2AX staining (Fig. 2).

**Fig. 3:**
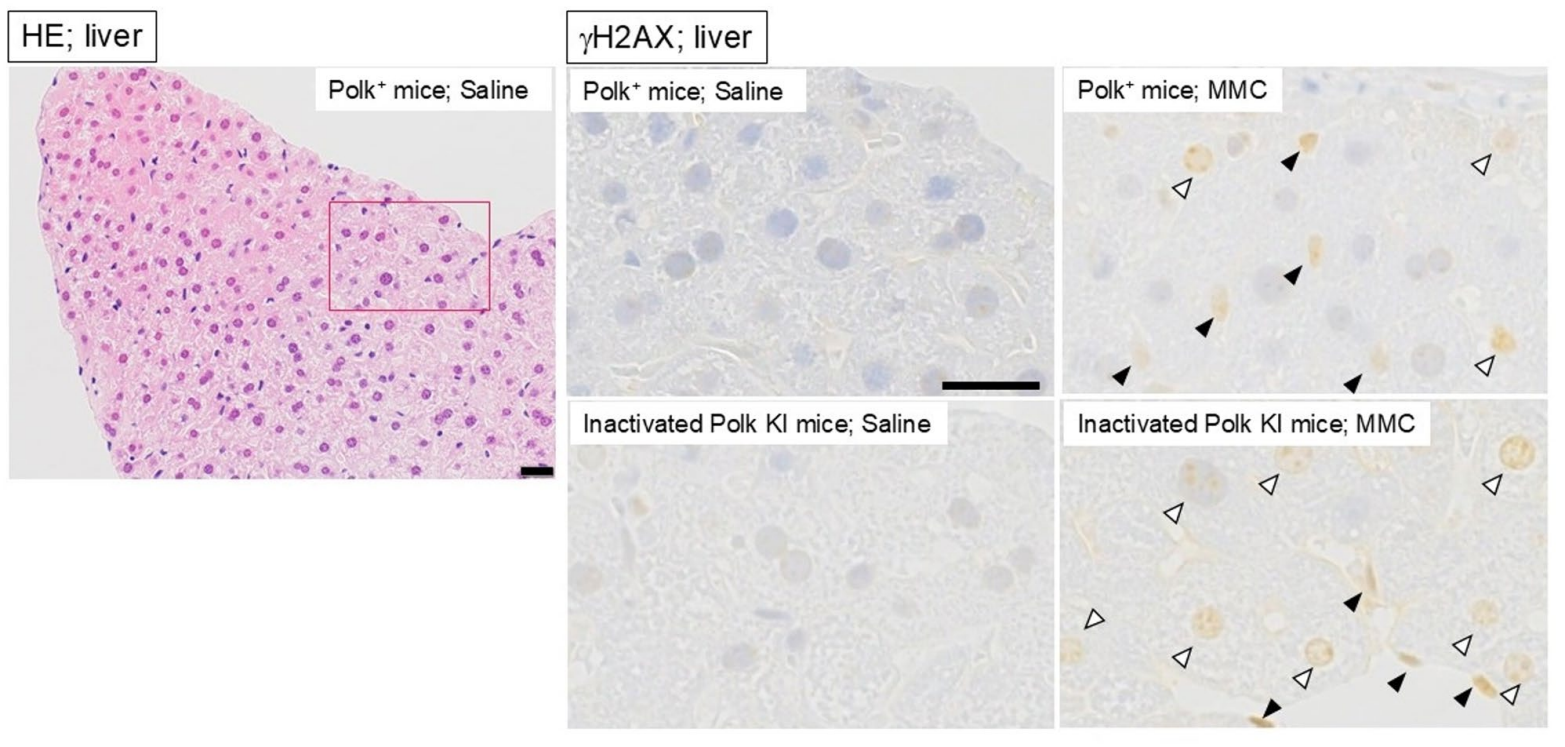
Representative images of liver. Hematoxylin-eosin (HE) staining in saline-treated Polk+ mice and immunohistochemical staining with anti-γH2AX antibody in each group are presented. Representative γH2AX positive hepatocyte (open arrow), and γH2AX positive sinusoidal cell (closed arrow) are shown. A red box on a HE image is an area enlarged in saline-treated Polk+ mice. Scale bars represent 20 μm

Liver: The numbers of γH2AX-positive cells in hepatocytes and sinusoidal cells were separately counted. One hundred cells from each of the 3 regions of a liver section (total 300 cells for each mouse) were counted to find γH2AX-positive cells. Hepatocytes whose round nucleus had focus- or pan-staining with anti-γH2AX antibody were defined as positive cells (Fig. 3). Sinusoidal cells whose oval or ellipse nucleus had pan-staining with anti-γH2AX antibody were defined as positive cells.

**Fig. 4:**
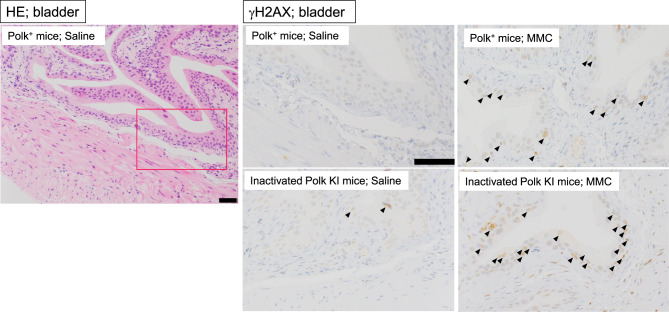
Representative images of bladder. Hematoxylin-eosin (HE) staining in saline-treated Polk^+^ mice and immunohistochemical staining with anti-γH2AX antibody in each group are presented. A red box on a HE image is an area enlarged in saline-treated Polk^+^ mice. Representative γH2AX positive epithelium cells (arrow) are shown. Scale bars represent 50 μm

Bladder: one hundred epithelium cells from each of the 3 regions on the section (total 300 cells for each mouse) were counted to find γH2AX positive cells. The cells whose oval-shaped nucleus had foci of anti-γH2AX staining were defined as positive cells (Fig. 4).

**Fig. 5:**
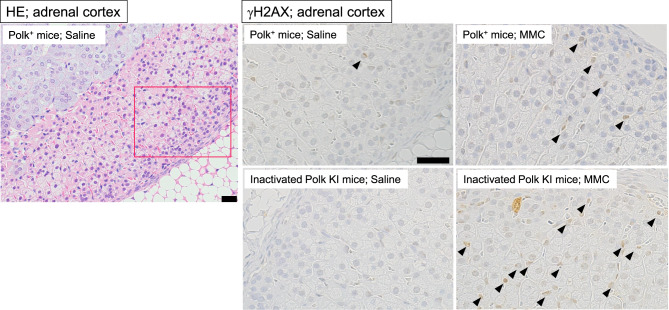
Representative images of adrenal cortex. Hematoxylin-eosin (HE) staining in saline-treated Polk^+^ mice and immunohistochemical staining with anti-γH2AX antibody in each group are presented. A red box on a HE image is an area enlarged in saline-treated Polk^+^ mice. Representative γH2AX positive cells (arrow) are shown. Scale bars represent 50 μm

Adrenal cortex: one hundred adrenal cortex cells from each of the 3 regions on the section (total 300 cells for each mouse) were counted to find γH2AX positive cells. The cells whose round-shaped nucleus had pan-staining with anti-γH2AX antibody were defined as positive cells (Fig. 5).

**Fig. 6:**
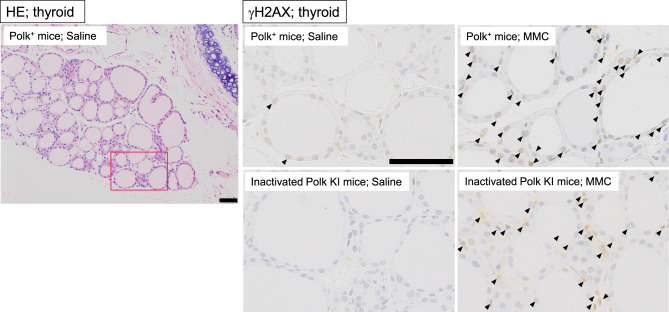
Representative images of thyroid. Hematoxylin-eosin (HE) staining in saline-treated Polk+ mice and immunohistochemical staining with anti-γH2AX antibody in each group are presented. A red box on a HE image is an area enlarged in saline-treated Polk^+^ mice. Representative γH2AX positive cells (follicular epithelial and parafollicular cells) are shown (arrow). Scale bars represent 50 μm

Thyroid: up to one hundred follicular epithelium cells or parafollicular cells from each of the 3 regions on the section (total 300 cells for each mouse) were counted to find γH2AX positive cells. The cells whose round-shaped nucleus had pan-staining with anti-γH2AX antibody were defined as positive cells (Fig. 6).

**Fig. 7:**
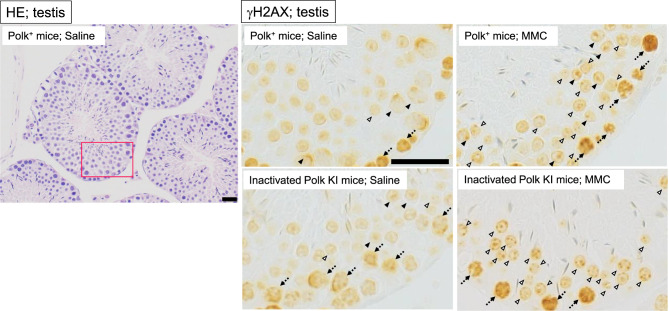
Representative images of testis. Hematoxylin-eosin (HE) staining in saline-treated Polk^+^ mice and immunohistochemical staining with anti-γH2AX antibody in each group are presented. Representative γH2AX positive round spermatids (open arrow) are shown. Spontaneous intense staining of γH2AX in spermatocytes (broken arrow) and foci of XY-body in pachytene stage spermatocytes (closed arrow) were excluded from the evaluation. A red box on a HE image is an area enlarged in saline-treated Polk^+^ mice. Scale bars represent 20 μm

Spermatids: among the 12 stages (I to XII) of the mouse spermatogenic cycle, spermatids in stages I to VII were evaluated (Supplementary S1). On stages I to VII, round spermatids, which were in the period after the second meiotic division and before nucleus condensation, were included. It takes approximately 6 days from stage I to VII. Therefore, in the present study where MMC was administered for 5 days, most of the spermatids in stages I to VII would have experienced MMC exposure during the first and second meiotic division. Pachytene stage spermatocytes placed in the vicinity of the basal lamina, whose nucleus had spontaneous strong staining of γH2AX, were not evaluated. The round spermatids with more than one focus of γH2AX staining were defined as positive cells (Fig. 7). The foci of XY-bodies which were spontaneously stained with anti-γH2AX antibody were excluded from the focus counting. Thirty cells from each seminiferous tubule and 300 cells from each male mouse were evaluated to find the positive cells.

### Statistical analysis

Regarding the quantitative evaluation, the incidence of γH2AX positive cells was statistically compared with Fisher’s exact test using a significance level of *p* < 0.01. The statistical software JMP (JMP Statistical Discovery, USA) was used for the analysis. Regarding the semi-quantitative analysis, statistical analysis was not conducted.

## Results

Saline or mitomycin C was intraperitoneally injected into Polk^+^ mice and Polk KI mice at a dose of 1 mg/kg/day for 5 days. Organs or tissues were collected 3 h after the last administration (Fig. 1). The organs and tissues were fixed, and section slides were prepared to stain immunohistochemically with anti-γH2AX (Ser139) mouse monoclonal antibody. To evaluate the protective effects of Polk against DSBs induced by MMC, semi-quantitative analyses of γH2AX stained cells were conducted in more than 30 organs or tissues (Fig. 2, microscopic images of each organ/tissue are shown in supplementary S2 to S33 and score data for individual mice are presented in supplementary S34). The γH2AX score was increased by MMC treatment in almost all tissues other than spleen and testis (whole cells) in Polk^+^ mice, suggesting systemic DNA damage induced by MMC (Fig. 2, No. 14 and 29, Group comparison A). In spleen and testis (whole cells), the increase of γH2AX score by MMC may be masked by the high background staining of γH2AX derived from spontaneous DSBs (see more detail in Discussion). In inactivated Polk KI mice, the γH2AX score was also systemically increased by MMC treatments, except in the testis (whole cells), hippocampus and cerebral white matter (Fig. 2, No. 29, 36, and 38, Group comparison B). In comparison between saline-treated groups, the score was higher in inactivated Polk KI mice than in Polk^+^ mice in some tissues such as adrenal cortex, prostate, and retina (Fig. 2, No. 3, 31, and 35, Group comparison C). In comparison between MMC-treated groups, the score was higher in inactivated Polk KI mice than in Polk^+^ mice in many tissues, including liver, bladder, and adrenal cortex (Fig. 2, No. 1–3, Group comparison D). However, the opposite tendency, i.e., lower scores in inactivated Polk KI mice than in Polk^+^ mice, was observed in retina, hippocampus, cerebral cortex, and cerebral white matter (Fig. 2, No. 35–38, Group comparison D, see more detail in Discussion). The representative microscopic images of HE staining and γH2AX immunohistochemical staining in liver, bladder, adrenal cortex, thyroid, and testis are presented in Figs. 3, 4, 5, 6 and 7, respectively.

To further define the protective roles of Polk against DSBs, we conducted quantitative analyses of γH2AX-stained cells in the following 5 organs or tissues (Tables 1 to 7).

**Table 1:**
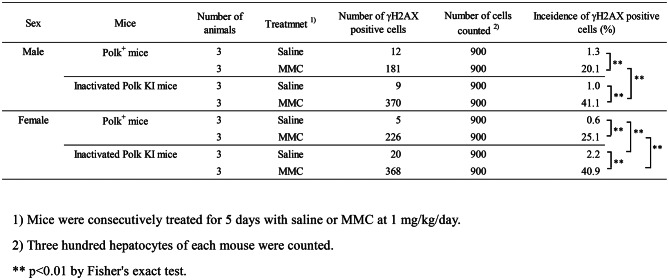
Incidence of γH2AX positive hepatocytes of saline- or MMC-treated Polk^+^ mice and inactivated Polk KI mice

**Table 2:**
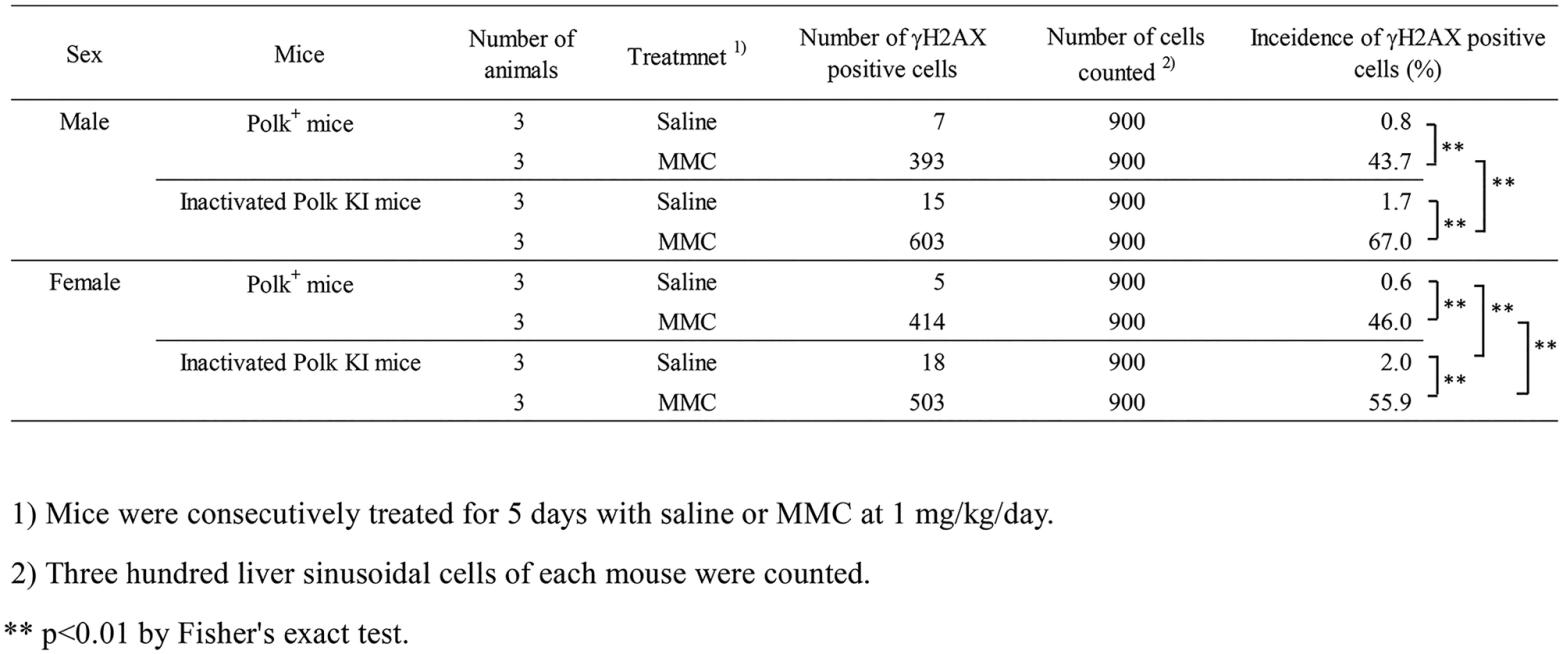
Incidence of γH2AX positive liver sinusoidal cells of saline- or MMC-treated Polk^+^ mice and inactivated Polk KI mice

**Table 3:**
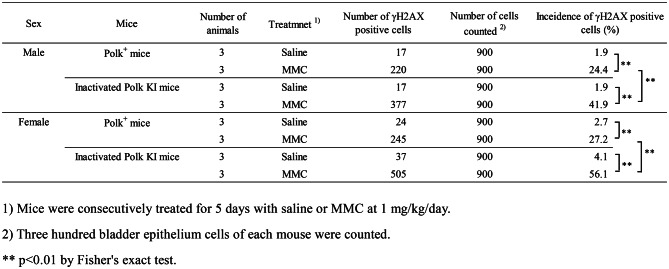
Incidence of γH2AX positive bladder epithelium cells of saline- or MMC-treated Polk^+^ mice and inactivated Polk KI mice

**Table 4:**
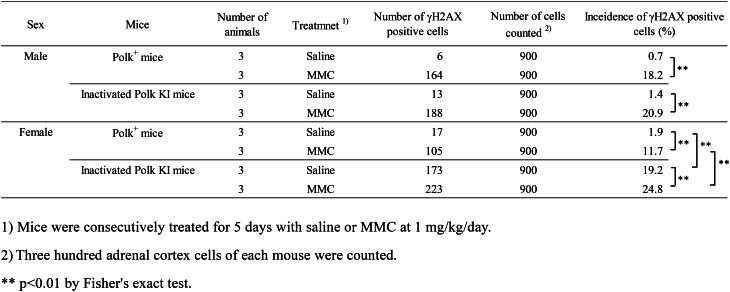
Incidence of γH2AX positive adrenal cortex cells of saline- or MMC-treated Polk^+^ mice and inactivated Polk KI mice

**Table 5:**
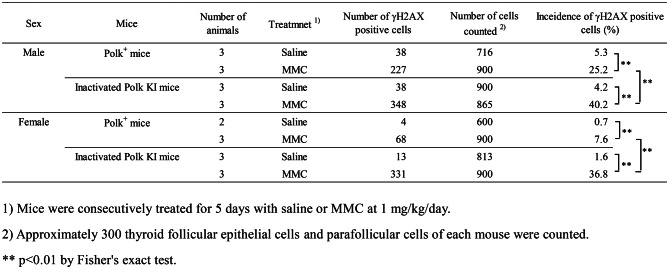
Incidence of γH2AX positive thyroid cells of saline- or MMC-treated Polk^+^ mice and inactivated Polk KI mice

**Table 6:**
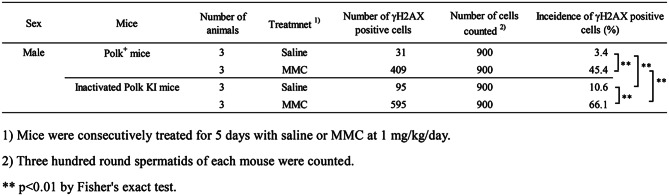
Incidence of γH2AX positive spermatids of saline- or MMC-treated Polk^+^ mice and inactivated Polk KI mice

**Table 7:**
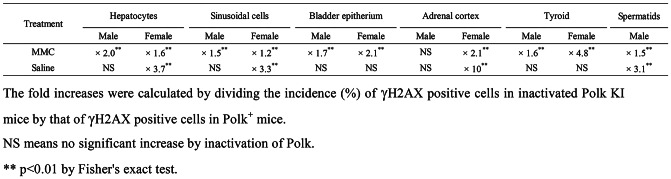
Fold increases in scores of γH2AX positive cells by inactivation of Polk

### Liver (hepatocytes)

The percentage of γH2AX positive hepatocytes was significantly higher in MMC-treated Polk^+^ mice than in saline-treated Polk^+^ mice (Table 1, Fig. 3). The increase was significant both in males (saline: 1.3% vs MMC: 20.1%, *p* < 0.01) and in females (saline: 0.6% vs MMC: 25.1%, *p* < 0.01). In inactivated Polk KI mice, the increase was also significant both in males (saline: 1.0% vs MMC: 41.1%, *p* < 0.01) and females (saline: 2.2% vs MMC: 40.9%, *p* < 0.01). When compared in Polk^+^ and inactivated Polk KI mice, the percentage of γH2AX positive hepatocytes was significantly higher in MMC-treated inactivated Polk KI mice than in MMC-treated Polk^+^ mice. The increase was observed both in males (Polk^+^ mice: 20.1% vs inactivated Polk KI mice: 41.1%, *p* < 0.01) and females (Polk^+^ mice: 25.1% vs inactivated Polk KI mice: 40.9%, *p* < 0.01). In saline-treated female mice, the percentage of γH2AX positive hepatocytes was higher in inactivated Polk KI mice than in Polk^+^ mice (Polk^+^ mice: 0.6% vs inactivated Polk KI mice: 2.2%, *p* < 0.01). The results suggest that Polk plays a protective role against MMC-induced DSBs in the hepatocytes of male and female mice, and also that it protects the genome even from spontaneous DSBs in the hepatocytes of female mice (Table 7).

### Liver (sinusoidal cells)

In liver sinusoidal cells, a similar trend was observed as with the hepatocytes (Table 2, Fig. 3). The percentage of γH2AX positive cells was significantly higher in MMC-treated Polk^+^ mice than in saline-treated Polk^+^ mice. The increase was significant in males (saline: 0.8% vs MMC: 43.7%, *p* < 0.01) and in females (saline: 0.6% vs MMC: 46.0%, *p* < 0.01). In inactivated Polk KI mice, the increase was also significant both in males (saline: 1.7% vs MMC: 67.0%, *p* < 0.01) and females (saline: 2.0% vs MMC: 55.9%, *p* < 0.01). When compared in Polk^+^ and inactivated Polk KI mice, the percentage of γH2AX positive sinusoidal cells was significantly higher in MMC-treated inactivated Polk KI mice than in MMC-treated Polk^+^ mice. The increase was observed both in males (Polk^+^ mice: 43.7% vs inactivated Polk KI mice: 67.0%, *p* < 0.01) and females (Polk^+^ mice: 46.0% vs inactivated Polk KI mice: 55.9%, *p* < 0.01). In saline-treated female mice, the percentage of γH2AX positive sinusoidal cells was higher in inactivated Polk KI mice than in Polk^+^ mice (Polk^+^ mice: 0.6% vs inactivated Polk KI mice: 2.0%, *p* < 0.01). The results suggest that Polk suppresses DSBs induced by MMC treatments in sinusoidal cells in male and female mice, and that it also protects against spontaneous DSBs in females (Table 7).

### Bladder

The percentage of γH2AX positive bladder epithelium cells was significantly higher in MMC-treated Polk^+^ mice than in saline-treated Polk^+^ mice (Table 3, Fig. 4). The increase was significant both in males (saline:1.9% vs MMC: 24.4%, *p* < 0.01) and females (saline: 2.7% vs MMC: 27.2%, *p* < 0.01). In inactivated Polk KI mice, the increase was also significant both in males (saline: 1.9% vs MMC: 41.9%, *p* < 0.01) and females (saline: 4.1% vs MMC: 56.1%, *p* < 0.01). When compared in Polk^+^ and inactivated Polk KI mice, the percentage of γH2AX positive cells was significantly higher in MMC-treated inactivated Polk KI mice than in MMC-treated Polk^+^ mice. The increase was observed both in males (Polk^+^ mice: 24.4% vs inactivated Polk KI mice: 41.9%, *p* < 0.01) and females (Polk^+^ mice: 27.2% vs inactivated Polk KI mice: 56.1%, *p* < 0.01). The results suggest that Polk exerts protective effects against MMC-induced DSBs in bladder epithelium cells in male and female mice (Table 7).

### Adrenal cortex

The percentage of γH2AX positive adrenal cortex cells was significantly higher in MMC-treated Polk^+^ mice than in saline-treated Polk^+^ mice (Table 4, Fig. 5). The increase was significant in males (saline: 0.7% vs MMC: 18.2%, *p* < 0.01) and in females (saline: 1.9% vs MMC: 11.7%, *p* < 0.01). In inactivated Polk KI mice, the increase was significant in males (saline: 1.4% vs MMC: 20.9%, *p* < 0.01) and in females (saline: 19.2% vs MMC: 24.8%, *p* < 0.01). The moderate increase, i.e., 1.3-fold, induced by MMC treatments in females may be due to the high number of background DSBs in the adrenal cortex of inactivated Polk KI female mice. When compared in Polk^+^ mice and inactivated Polk KI mice, the percentage of γH2AX positive adrenal cortex cells was significantly higher in MMC-treated inactivated Polk KI female mice than in MMC-treated Polk^+^ female mice (Polk+ mice: 11.7% vs inactivated Polk KI mice: 24.8%, *p* < 0.01). In saline-treated female mice, the percentage of γH2AX positive cells of inactivated Polk KI mice was higher than that of saline-treated Polk^+^ mice (Polk^+^ mice: 1.9% vs inactivated Polk KI mice: 19.2%, *p* < 0.01). Therefore, Polk appears to suppress not only MMC-induced DSBs but also spontaneous DSBs in the adrenal cortex of female mice. No protective effects of Polk against DSBs were observed in male mice (Table 7).

### Thyroid

The percentage of γH2AX positive thyroid cells was significantly higher in MMC-treated Polk^+^ mice than in saline-treated Polk^+^ mice (Table 5, Fig. 6). The increase was significant both in males (saline: 5.3% vs MMC: 25.2%, *p* < 0.01) and females (saline: 0.7% vs MMC: 7.6%, *p* < 0.01). In inactivated Polk KI mice, the increase was also significant both in males (saline: 4.2% vs MMC: 40.2%, *p* < 0.01) and females (saline: 1.6% vs MMC: 36.8%, *p* < 0.01). When compared in Polk^+^ and inactivated Polk KI mice, the percentage of γH2AX positive thyroid cells was significantly higher in MMC-treated inactivated Polk KI mice than in MMC-treated Polk^+^ mice. The increase was significant both in males (Polk^+^ mice: 25.2% vs inactivated Polk KI mice: 40.2%, *p* < 0.01) and females (Polk^+^ mice: 7.6% vs inactivated Polk KI mice: 36.8%, *p* < 0.01). The results suggest that Polk exerts protective effects against MMC-induced DSBs in the thyroid of male and female mice.

### Testis (spermatids)

The percentage of γH2AX positive spermatids was significantly higher (*p* < 0.01) in MMC-treated Polk^+^ mice (45.4%) than in saline-treated Polk^+^ mice (3.4%) (Table 6, Fig. 7). In inactivated Polk KI mice, the increase was also significant (saline: 10.6% vs MMC: 66.1%, *p* < 0.01). When compared in Polk^+^ mice and inactivated Polk KI mice, the percentage of γH2AX positive spermatids was significantly higher (*p* < 0.01) in MMC-treated inactivated Polk KI mice (66.1%) than in MMC-treated Polk^+^ mice (45.4%). In saline-treated mice, the percentage of γH2AX positive spermatids was higher in inactivated Polk KI mice than in saline-treated Polk^+^ mice (Polk^+^ mice: 3.4% vs inactivated Polk KI mice: 10.6%, *p* < 0.01). It seems that Polk suppresses spontaneous DSBs as well as MMC-induced DSBs in the testis (Table 7).

## Discussion

The DNA cross-linking agent MMC is widely used as a first-line chemotherapeutic drug against a variety of tumors; however, various side effects have been reported. Cardiac toxicity [26], renal toxicity [27], hepatotoxicity [28], pulmonary toxicity [29], and hematopoietic toxicity [30] are observed in clinical usage as dose-limiting toxicities. As for genotoxicity, micronucleus induction in liver, bone marrow, and male germ cells were reported [31–33]. Furthermore, DSB induction in blood, bone marrow, liver, stomach, spleen, testis, lung, and kidney were investigated [34]. Although the systemic genotoxicity of MMC is inferred from these studies, protective mechanisms against MMC-induced DSB have not been thoroughly examined in vivo.

**Fig. 8:**
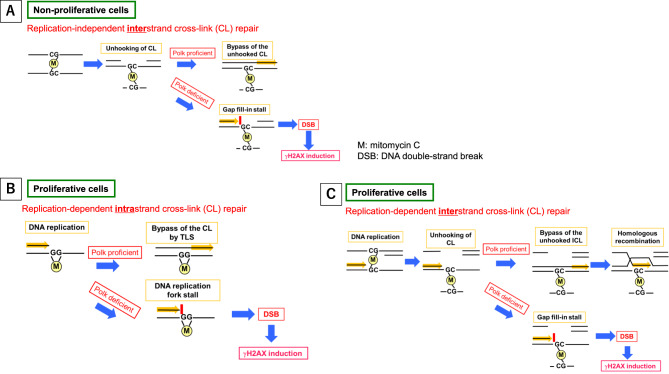
Mechanisms of enhancement of γH2AX induction by inactivation of Polk function are illustrated. **A**) in less or slowly proliferative cells, replication-independent interstrand cross-link (CL) repair is involved. In this pathway, the interstrand CLs are initially unhooked by endonucleases from one of the two DNA strands. Polk then facilitates bypass of the unhooked interstrand CLs. When Polk function is inactivated, this pathway would be disrupted, enhancing DSB induction and leading to increased γH2AX induction. On the other hand, in proliferative cells, a replication-dependent repair pathway is involved. DNA syntheses at replication forks are stalled at the sites of intrastrand CLs (**B**) or interstrand CLs (**C**) of MMC. **B**) at intrastrand CLs, stalled DNA syntheses are resumed by Polk-mediated TLS, but in the absence of polk function, the stalled DNA syntheses persist, inducing DSBs. **C**) the interstrand CLs are initially unhooked by endonucleases from one of the two DNA strands. Then, Polk bypasses the unhooked interstrand CLs to generate an intact daughter DNA strand. Furthermore, broken opposite strand DNA is restored by homologous recombination. Under Polk-inactivated conditions, persistent stalling of DNA syntheses induces DSBs

DSBs are among the most harmful DNA lesions because they inevitably and rapidly lead to cell necrosis or apoptosis when the quantity of lesions exceeds the capacity for cells to repair themselves [35]. Even if the DSBs are repaired with DNA repair systems such as homologous recombination or non-homologous end-joining, these repairs are sometimes imperfect, resulting in short sequence deletions or large allele losses, both of which severely impair cell functions [36]. MMC generates intrastrand cross-links at GpG sites in the same strand of DNA and interstrand cross-links at CpG sites in opposite strands of DNA (Fig. 8) [37]. Conventional DNA Pols, such as Pol δ, are stuck at the intrastrand cross-links and the DNA replication is stalled. Persistent replication block may induce DSBs if TLS Pols do not work to traverse the cross-links. Interstrand cross-links, which make two complementary DNA strands inseparable, also block DNA replication. There are two pathways to remove the lesion: S-phase-dependent and -independent interstrand cross-link repair [38–40]. In both pathways, the cross-links are initially unhooked by endonucleases from one of the two DNA strands, followed by TLS bypassing the unhooked interstrand cross-link. In our previous study, the inactivation of Polk enhanced the frequency of micronuclei formation and γH2AX induction in MMC-treated mouse bone marrow [24]. In the present study, in order to deepen our understanding of how Polk prevents DSB generation, we investigated γH2AX induction, a marker of DSB (supplementary S35), across various tissues or organs in Polk^+^ mice and inactivated Polk KI mice.

The semi-quantitative evaluation of γH2AX staining revealed that MMC treatment increased the staining score of almost all tissues and organs in both Polk^+^ mice and inactivated Polk KI mice compared to saline-treated control (Fig. 2, Group comparison A and B). This finding suggests that MMC induces DNA damage systemically regardless of Polk status. In Polk^+^ mice, MMC treatments increased the score in all tissues or organs except for spleen (Fig. 2, No. 14, Group comparison A, supplementary S10) and the whole area of testis (Fig. 2, No. 29, Group comparison A, Fig. 7). The finding is consistent with a report by Matsuda et al. where a mass spectrometry measurement showed no significant increase in γH2AX in spleen and testis of MMC-treated wild type mice [34]. Notably, in our present study, strong γH2AX staining was already present in the spleen and testis of saline-treated mice, likely reflecting physiological DSBs or chromatin remodeling unique to these tissues. In the spleen, T cell receptor gene rearrangement [41] and immunoglobulin gene rearrangement in B cells likely account for the baseline levels of γH2AX staining [42]. Reduction of γH2AX staining by MMC-treatment in Polk^+^ mice (Fig. 2, Group comparison A, Supplementary S10) may reflect an immunosuppressive effect of MMC on B cells. Similarly in the testis, chromatin remodeling and associated silencing of male sex chromosomes, contributing to prominent γH2AX staining [43]. This inherent high background staining may mask any additional increase caused by MMC in these organs. In testis, however, MMC-induced γH2AX staining was detected when we focused on round spermatids at specific stage of spermatogenesis, as described in the section of materials and methods (supplementary S1).

In inactivated Polk KI mice, MMC treatments increased the scores in almost all tissues or organs (Fig. 2, Group comparison B). However, the increase was not observed in testis (whole area) and spleen probably for the same reasons as in Polk^+^ mice as described above (Fig. 2, No. 29 and 14, Group comparison B). In inactivated Polk KI mice, in addition to the spleen and testis, score gain was not observed in brain (i.e., hippocampus, cerebral cortex, and cerebral white matter in Fig. 2, No. 36–38, Group comparison B). Spontaneous phosphorylation of γH2AX has been reported in the mouse brain [44]. ROS generated by catecholamine oxidation may contribute to this spontaneous γH2AX induction in the brain [45]. In the present study, high or mid scores for γH2AX positive cells were observed in the hippocampus, cerebral cortex, and cerebral white matter, which is consistent with the previous report by Merighi et al. [44]. Polk inactivation might enhance spontaneous γH2AX induction, which may mask the effect of MMC-treatment.

In the saline-treated groups, the score was higher in inactivated Polk KI mice than in Polk^+^ mice in adrenal cortex, prostate, and retina (Fig. 2, No. 3, 31, and 35, Group comparison C). Intrinsic ROS generation in these tissues was reported [46–48]. The intrinsic ROS can generate lipid peroxidation products such as malondialdehyde, which is known to induce DNA cross-links [49]. Polk might contribute to the repair of DNA damage caused by intrinsic ROS in these tissues.

In comparison between MMC-treated groups, the γH2AX score was higher in inactivated Polk KI mice than in Polk^+^ mice across many tissues, such as liver, bladder, adrenal cortex, thyroid, spermatids (Fig. 2, No. 1–5, Group comparison D, Table 1–7). The results suggest that Polk plays a systemic role in preventing DSBs caused by MMC. Conversely, decreases in the score were observed in brain (hippocampus, cerebral cortex, and cerebral white matter, Fig. 2, No. 36–38, Group comparison D) and retina in MMC-treated inactivated Polk KI mice compared to MMC-treated Polk^+^ mice (Fig. 2, No. 35, Group comparison D). As mentioned above, Polk inactivation might enhance the spontaneous DSB induction through intrinsically generated ROS in the brain [44, 45]. Consequently, DSB repair pathways such as HR or NHEJ in inactivated Polk KI mice could be more highly stimulated than in Polk^+^ mice. The limited ability of MMC to penetrate the blood-brain barrier has been reported [50]. Therefore, the observed increase in γH2AX scores in the brain may be attributed to ROS generated by MMC exposure [51]. Although the exact reasons for the lower scores in the brains of MMC-treated inactivated Polk KI mice compared to MMC-treated Polk^+^ mice are currently unknown, enhanced DSB repair pathways might mitigate DSB induction in MMC-treated inactivated Polk KI mice. Retina has been identified as a target tissue of endogenous ROS [48]. The reduced increase in γH2AX score following MMC treatment in the retina of inactivated Polk KI mice could also be explained by the saturation of γH2AX phosphorylation and/or stimulation of DSB repair pathway activity by endogenous ROS.

Polk mRNA expression levels in C57BL/6J mice have been reported [52] and are summarized in Fig. 2 (2^nd^ column from the left). According to the report, Polk expression levels can be classified into four groups: very high-expression levels in bladder and testis (Fig. 2, No. 2, 5 and 29), high-expression levels in cerebellum and cerebral cortex (Fig. 2, No. 32 and 37), middle-expression levels in adrenal cortex, mammary gland, lung, kidney, and thymus (Fig. 2, No. 3, 11, 17, 18, 33), and low-expression levels in liver, stomach, spleen, ileum, colon, and heart (Fig. 2, No. 1, 7, 8, 14, 15, 22, and 24). The very high expression of Polk in the bladder and testis aligns with score increase from Polk inactivation in MMC-treated mice in these tissues (Fig. 2, No. 2, 5, and 29, Tables 3, 6 and 7). Conversely, the results seem inconsistent in the liver and stomach, i.e., the score increase from Polk inactivation was high, whereas the expression levels of Polk are low (Table 1, 2, 7 and Fig. 2, No. 1, 7, and 8, Group comparison D). Therefore, Polk expression levels do not necessarily correlate directly with its contribution to the prevention of MMC-induced and spontaneous DSBs (Fig. 2, comparison between 2^nd^ column form left and Group comparison C or D).

The quantitative evaluation of γH2AX was conducted in liver, bladder, adrenal cortex, thyroid, and spermatids, as these were the top 5 organs or tissues that showed the most obvious effects of Polk inactivation on MMC-induced DSBs in the semi-quantitative analysis (Table 1–7, Fig. 2, No. 1–5, Group comparison D). Summaries of comparisons between Polk^+^ mice and inactivated Polk KI mice, and between male and female mice, are shown in Table 7 and 8, respectively. In these organs or tissues of MMC-treated mice, a significantly higher induction frequency of γH2AX positive cells was observed in inactivated Polk KI mice than in Polk^+^ mice, indicating a clear protective role of Polk against MMC-induced DSBs (Table 7). This protective effect was observed in proliferative tissues such as bladder epithelial cells (Fig. 2, No. 2, Table 3, Fig. 4) and the squamous basal cell layer of trachea epithelium (Fig. 2, No. 25, Group comparison D, supplementary S21), suggesting that Polk contributes to protection during DNA replication (Fig. 8 B, C). However, Polk also demonstrated a protective role in non-proliferative tissues, such as hepatocytes (Fig. 2, No. 1, Table 1, Figs. 3, 8 A). It is reported that Polk is involved in the replication-independent repair of interstrand cross-links in *Xenopus* egg extracts [22] and the repair pathway is presented [53]. The present results suggest that Polk is involved in both repair pathways because the inactivation of Polk enhanced the γH2AX scores in proliferative cells as well as in less or slow proliferative cells when the mice were treated with MMC (Table 1–7, Fig. 2 Group comparison D, Fig. 8 A, B, C). In addition to Polk, another specialized Pol, e.g., Pol ζ, is suggested to be involved in TLS in both types of interstrand cross-link repair [38]. The inactivation of Pol ζ enhances the killing sensitivity against MMC in human cells [54]. This involvement of Pol ζ in the repair of interstrand crosslinks may offset the contribution of Polk, potentially explaining the relatively moderate increases in γH2AX positive cells observed with Polk inactivation across organs and tissues in MMC-treated mice (Table 7). Obvious differences in the incidence of γH2AX positive cells between males and females was observed in bladder epithelium, adrenal cortex, and thyroid (Table 8). In the adrenal cortex, saline-treated inactivated Polk KI female mice showed more than 10-fold higher values than male mice (19.2% female vis 1.4% male). Furthermore, Polk inactivation significantly enhanced the incidence of γH2AX positive cells in saline-treated female mice (Polk^+^ female mice: 1.9% vs inactivated Polk KI female mice: 19.2%, Table 4). This enhancement was not observed in male mice. A three-fold higher cell turnover in adrenal cortex of female mice compared to male mice was reported [55]. Therefore, Polk may prevent the accumulation of spontaneous DSBs, which may be induced in highly proliferating adrenal cortex cells in female mice. In the thyroid, saline-treated Polk^+^ male mice showed an 8-fold higher incidence of γH2AX positive cells than female mice (Table 8). In contrast, higher oxidative stress in thyroid of female rats compared to male rats was reported [56]. In the bladder, saline-treated inactivated Polk KI female mice showed approximately 2-fold higher values than male mice (Table 8). As far as we surveyed, we found no reports suggesting sex differences in ROS generation or cell proliferation in the bladder. Therefore, the underlying mechanisms for the observed sex differences in γH2AX induction in the thyroid and bladder remain unclear. In the liver, no consistent sex-specific patterns in γH2AX induction were observed between the hepatocytes and the sinusoidal cells. Although quantitative analysis was not performed in the colon in the present study, Hakura et al. reported that male mice exhibited greater sensitivity to DSS-induced inflammation and dysplasia in the colon compared to female mice, regardless of Polk status. Furthermore, the mutation frequencies in inactivated Polk KI male mice were higher than those observed in female mice [20].

**Table 8:**
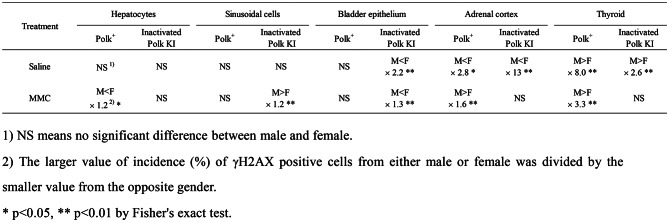
Fold difference of incidence of γH2AX positive cells between male and female mice

Heart, liver, kidney, lung, hematopoietic tissue (bone marrow), and testis have been reported as target organs of MMC-induced toxicity [26–30, 32, 33]. In liver hepatocytes, sinusoidal cells, and spermatids in the testis, an obvious increase in γH2AX positive cells was observed in both Polk^+^ mice and inactivated Polk KI mice following MMC treatment (Figs. 3 and 7, Table 1, 2 and 6). We previously reported that γH2AX positive bone marrow cells were increased by MMC-treatment in mice [24]. Furthermore, Polk inactivation enhanced the incidence of γH2AX positive cells in liver, bone marrow, and testis in both the present study and our previous study [24]. These findings suggest that MMC-induced organ or tissue toxicity may be linked to DSB induction and that Polk plays a role, at least partially, in preventing such toxicities in these organs or tissues. On the other hand, in kidney, heart, and lung, the induction frequency of γH2AX positive cells was moderate (Supplementary S14, S20, S13) in the present study. MMC-induced renal toxicity is suggested to be unrelated to DNA damage, instead being mediated by thrombotic microangiopathy [27]. Although ROS generation is believed to contribute to the MMC-induced cardiotoxicity and pulmonary toxicity, cumulative dosing is required to induce this toxicity [26, 29]. Therefore, the 5-day treatment duration in this study might be insufficient for detecting MMC-induced DNA damage in heart and lungs, or the toxicities induced in these organs may not be directly linked to DNA damage.

## Conclusion

In this study, we comprehensively investigated the effects of Polk on MMC-induced γH2AX in Polk^+^ mice and inactivated Polk KI mice. The results suggested that Polk protect against MMC-induced DNA damage across various tissues through DNA replication-dependent and -independent repair mechanisms. Furthermore, Polk may contribute to avoidance from DNA damage in response to intrinsically generated mutagens in some organs.

## Electronic supplementary material

Below is the link to the electronic supplementary material.

Supplementary Material 1

Supplementary Material 2

## Data Availability

The datasets generated and analyzed in the current study are available from the corresponding author on reasonable request.

## Abbreviations

BER: Base excision repair
CL: Cross-link
DSB: DNA double strand break
FA: Fanconi anemia
HE: Hematoxylin-eosin
inactivated Polk KI mice: inactivated DNA polymerase κ knock-in mice
KI: Knock-in
KO: Knock-out
MMC: Mitomycin C
NER: Nucleotide excision repair
Pol: DNA polymerase
Polk: DNA polymerase κ
Polk^+^ mice: DNA polymerase κ wild type mice
ROS: Reactive oxygen species
TLS: Translesion DNA synthesis
