# Supplementary material for: Mitomycin C-induced DNA double-strand breaks are enhanced by catalytical inactivation of DNA polymerase κ in mice
Source: Genes Environ. 2025 Nov 6;47:22. doi: 10.1186/s41021-025-00343-x (PMC12590760; doi:10.1186/s41021-025-00343-x)
Supplement: Supplementary file 1 — Supplementary Material 1 [file 41021_2025_343_MOESM1_ESM.pdf]

Supplementary S1

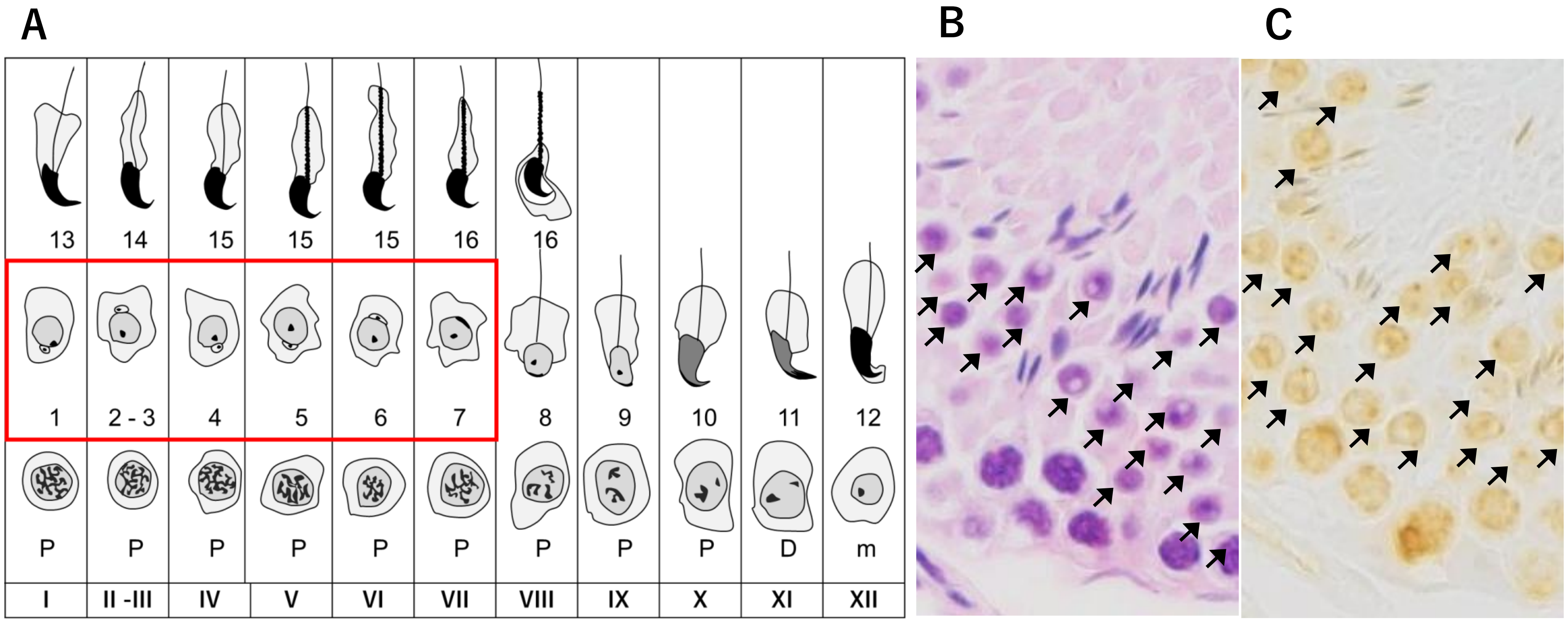

(A) Mouse spermatogenic cycle is illustrated. 1-16, step 1-16 spermatids; P, pachytene spermatocytes; D, diplotene spermatocytes; m, meiotic division. Among 12 stages (I to XII), round spermatids in stage I to VII (red box, step 1 to 7) were evaluated for γH2AX foci staining in this study. (B) Hematoxylin-eosin staining. (C) Immunohistochemical staining with anti-γH2AX antibody. Arrows represent step 1 to7 round spermatids.

Supplementary S2

Tongue basal epithelial

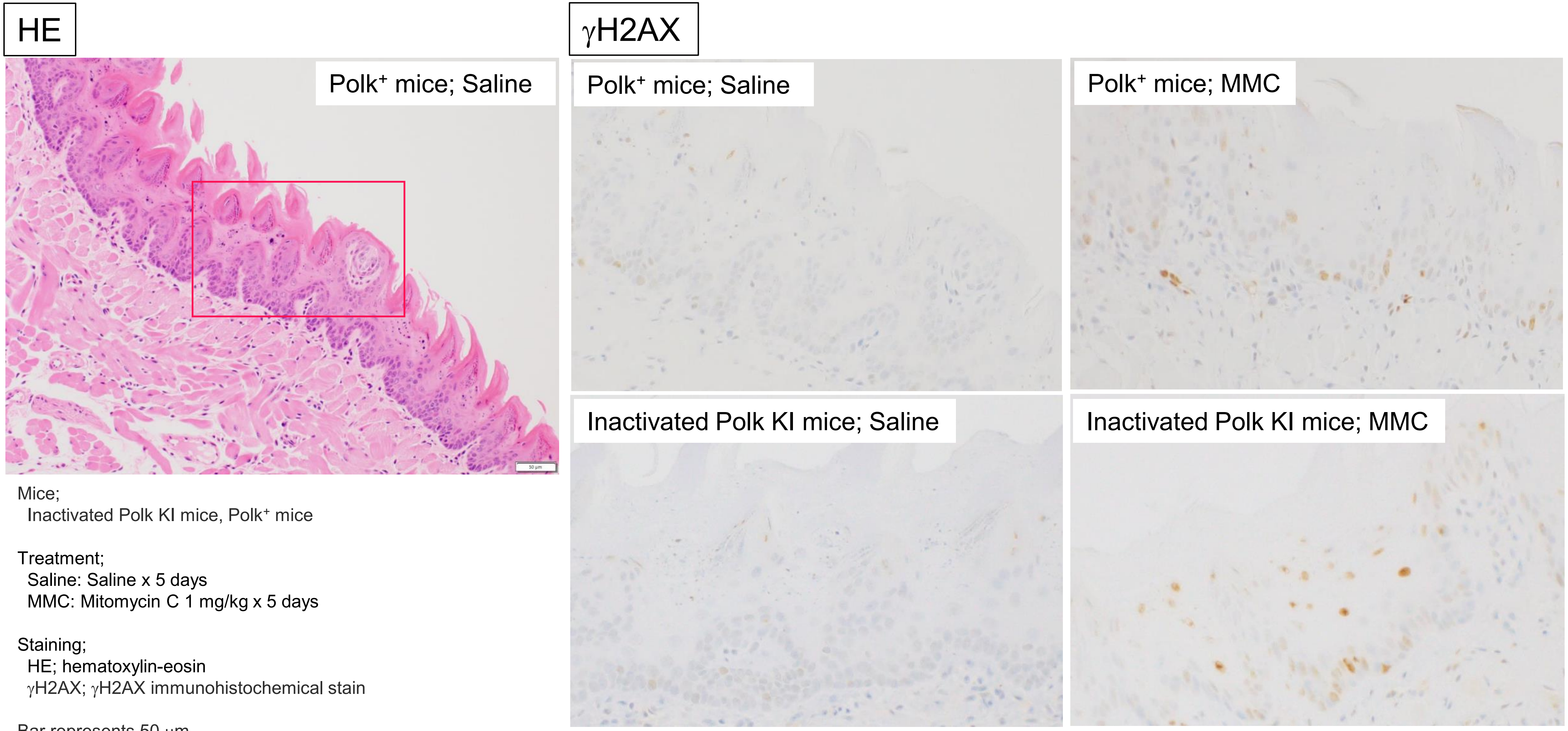

Mice;
Inactivated Polk KI mice, Polk^{+} mice

Treatment;
Saline: Saline x 5 days
MMC: Mitomycin C 1 mg/kg x 5 days

Staining;
HE; hematoxylin-eosin
γH2AX; γH2AX immunohistochemical stain

Bar represents 50 μm

Supplementary S3
Glandular stomach

HE

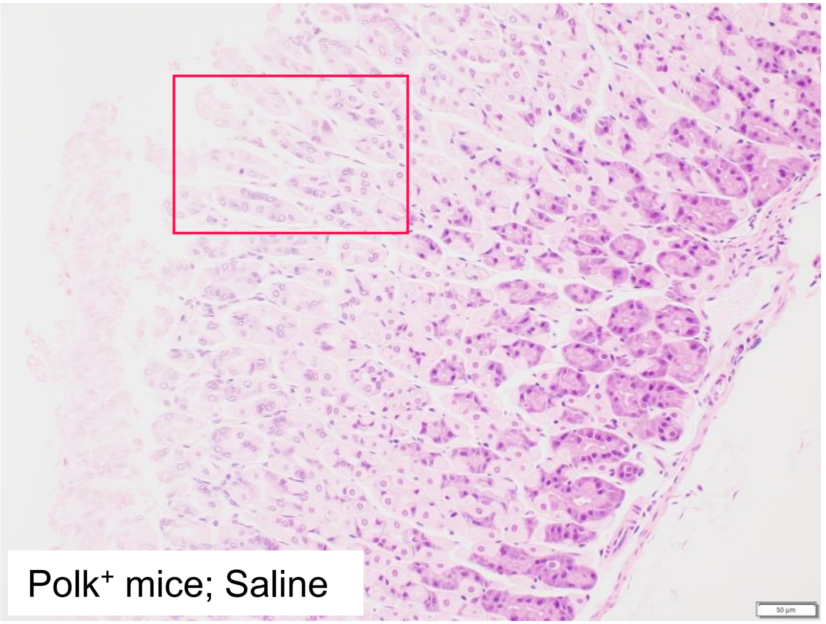

γH2AX

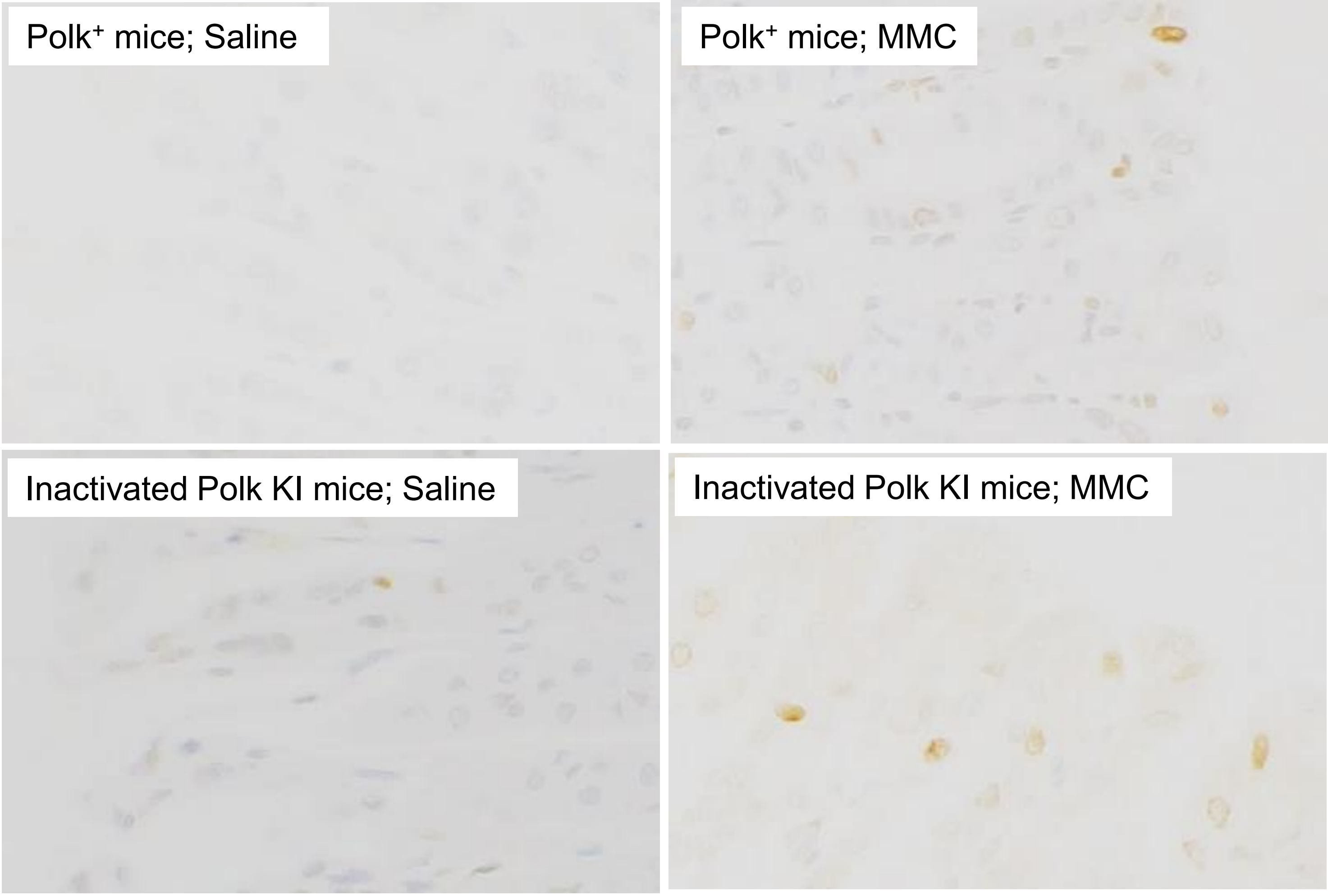

Mice;
Inactivated Polk KI mice, Polk^{+} mice

Treatment;
Saline: Saline x 5 days
MMC: Mitomycin C 1 mg/kg x 5 days

Staining;
HE; hematoxylin-eosin
γH2AX; γH2AX immunohistochemical stain

Bar represents 50 μm

Supplementary S4

Forestomach

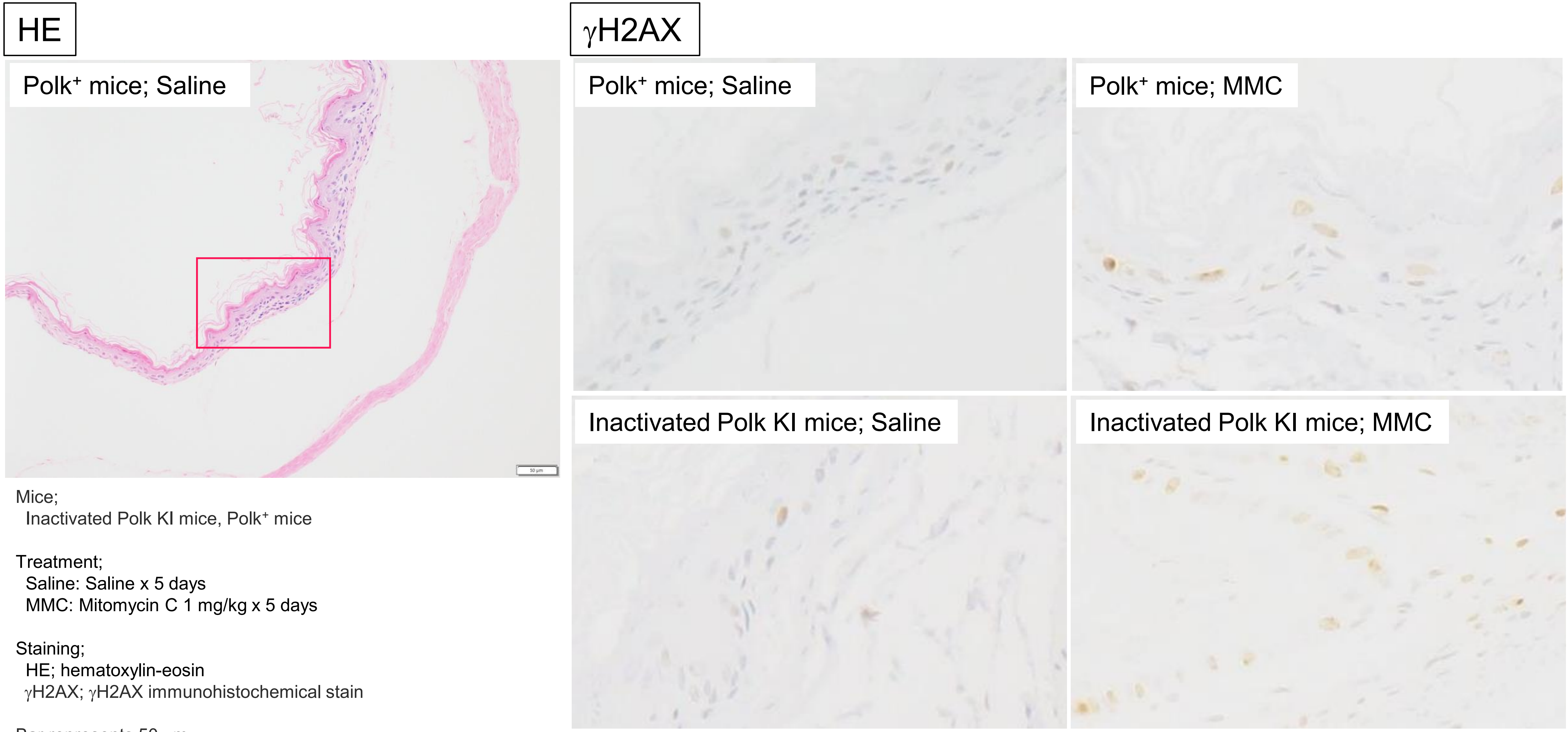

Mice;
Inactivated Polk KI mice, Polk⁺ mice

Treatment;
Saline: Saline x 5 days
MMC: Mitomycin C 1 mg/kg x 5 days

Staining;
HE; hematoxylin-eosin
γH2AX; γH2AX immunohistochemical stain

Bar represents 50 μm

Supplementary S5
Submandibular gland

HE

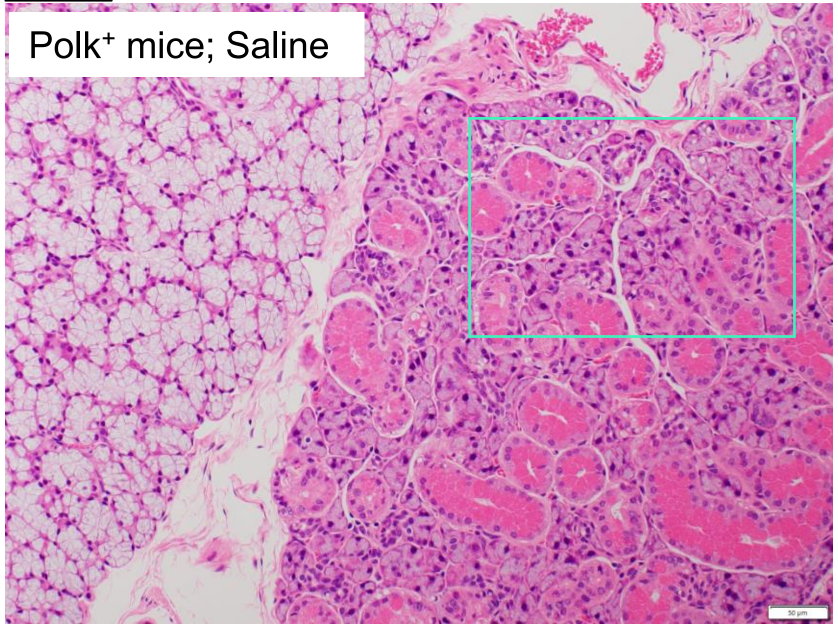

γH2AX

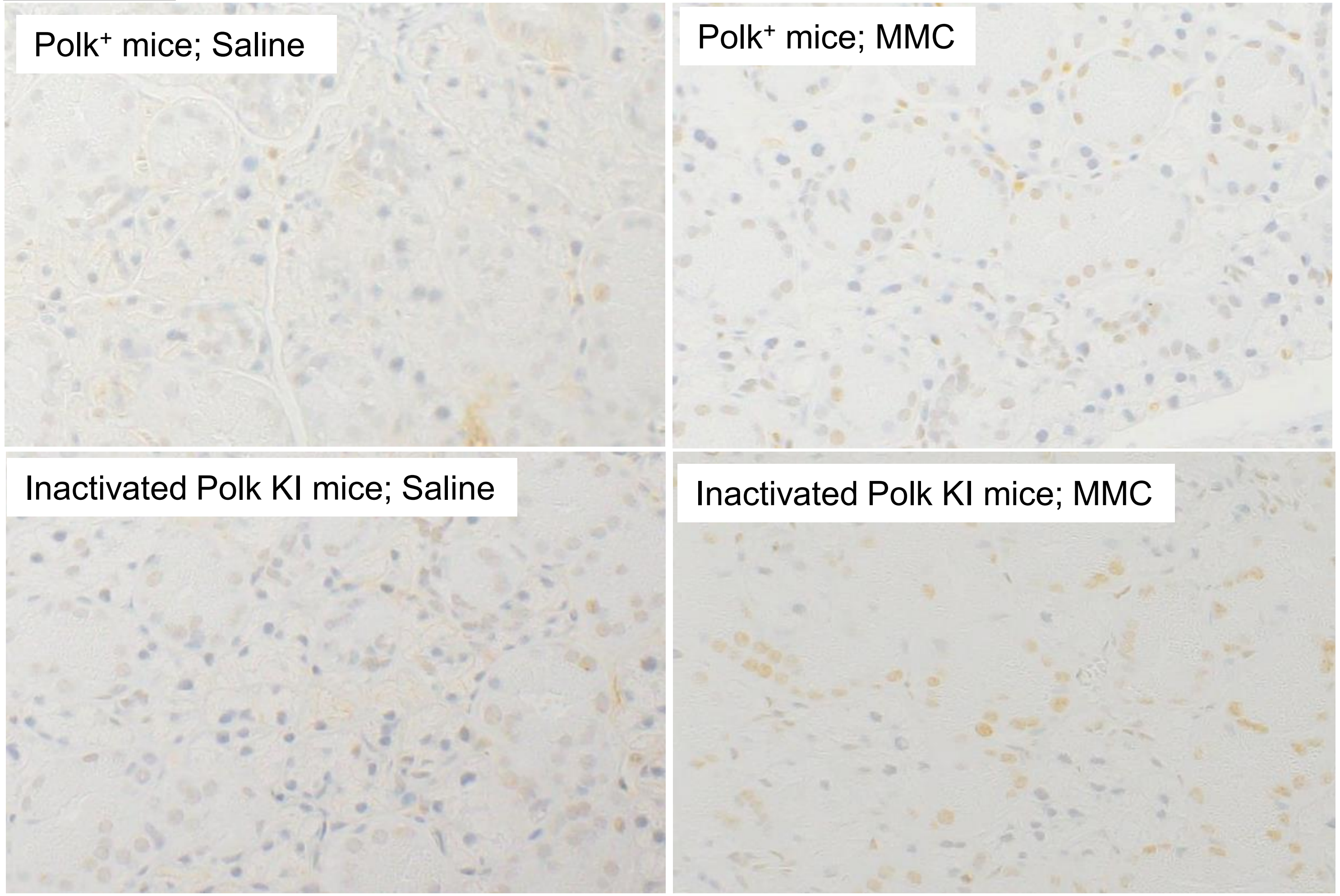

Mice;
 Inactivated Polk KI mice, Polk^{+} mice

Treatment;
 Saline: Saline x 5 days
 MMC: Mitomycin C 1 mg/kg x 5 days

Staining;
 HE; hematoxylin-eosin
 γH2AX; γH2AX immunohistochemical stain

Bar represents 50 μm

Supplementary S6

Esophagus

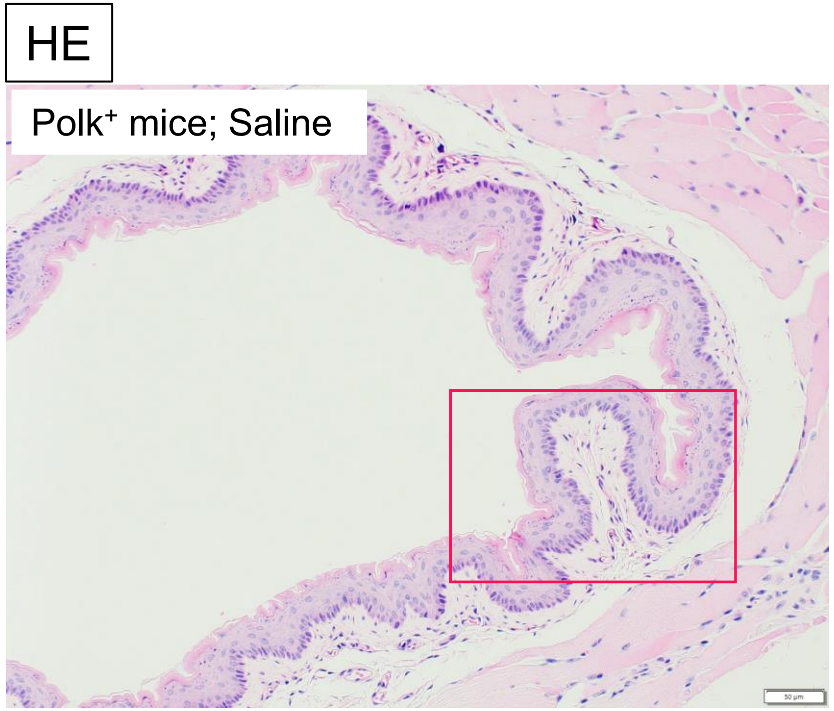

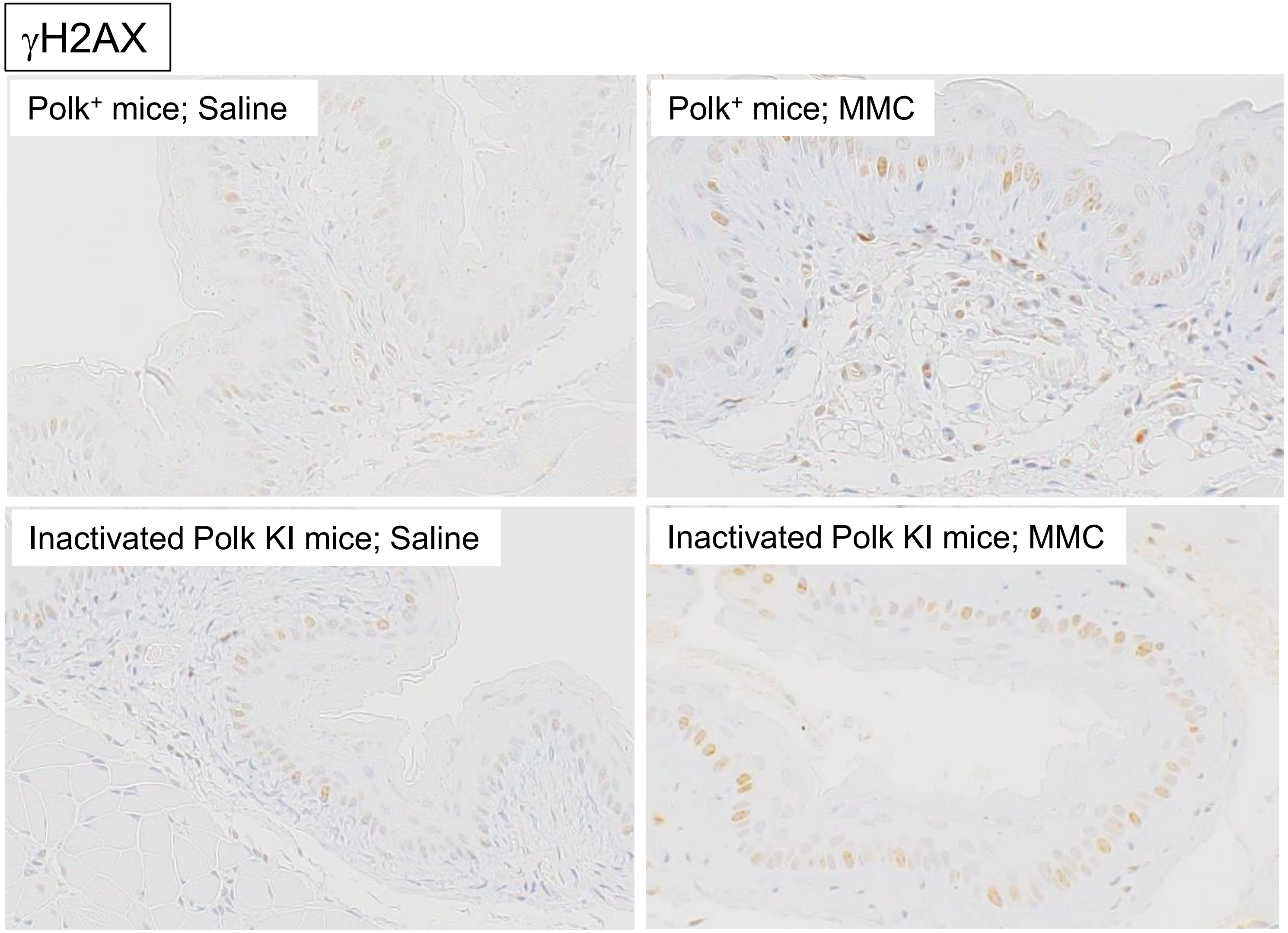

Mice;
Inactivated Polk KI mice, Polk^{+} mice

Treatment;
Saline: Saline x 5 days
MMC: Mitomycin C 1 mg/kg x 5 days

Staining;
HE; hematoxylin-eosin
γH2AX; γH2AX immunohistochemical stain

Bar represents 50 μm

Supplementary S7

Mammary gland

HE

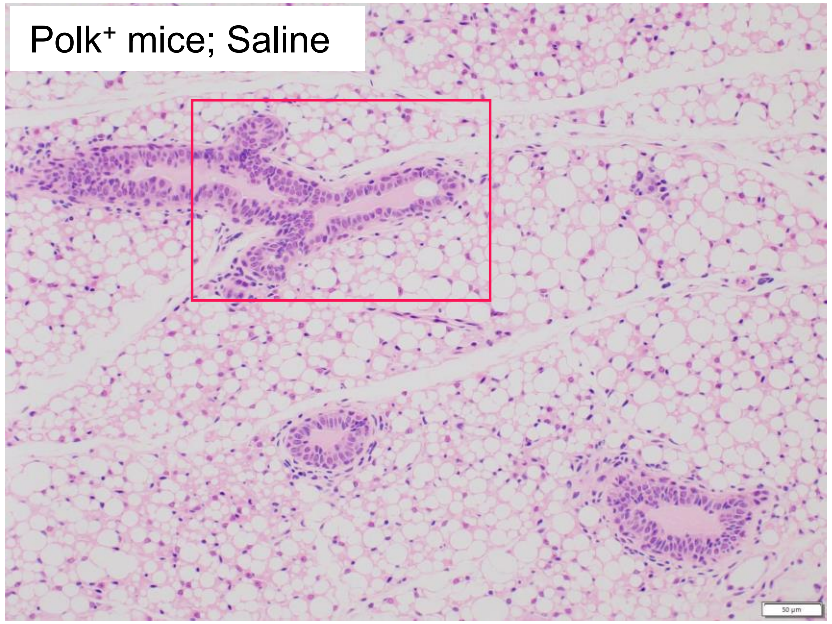

γH2AX

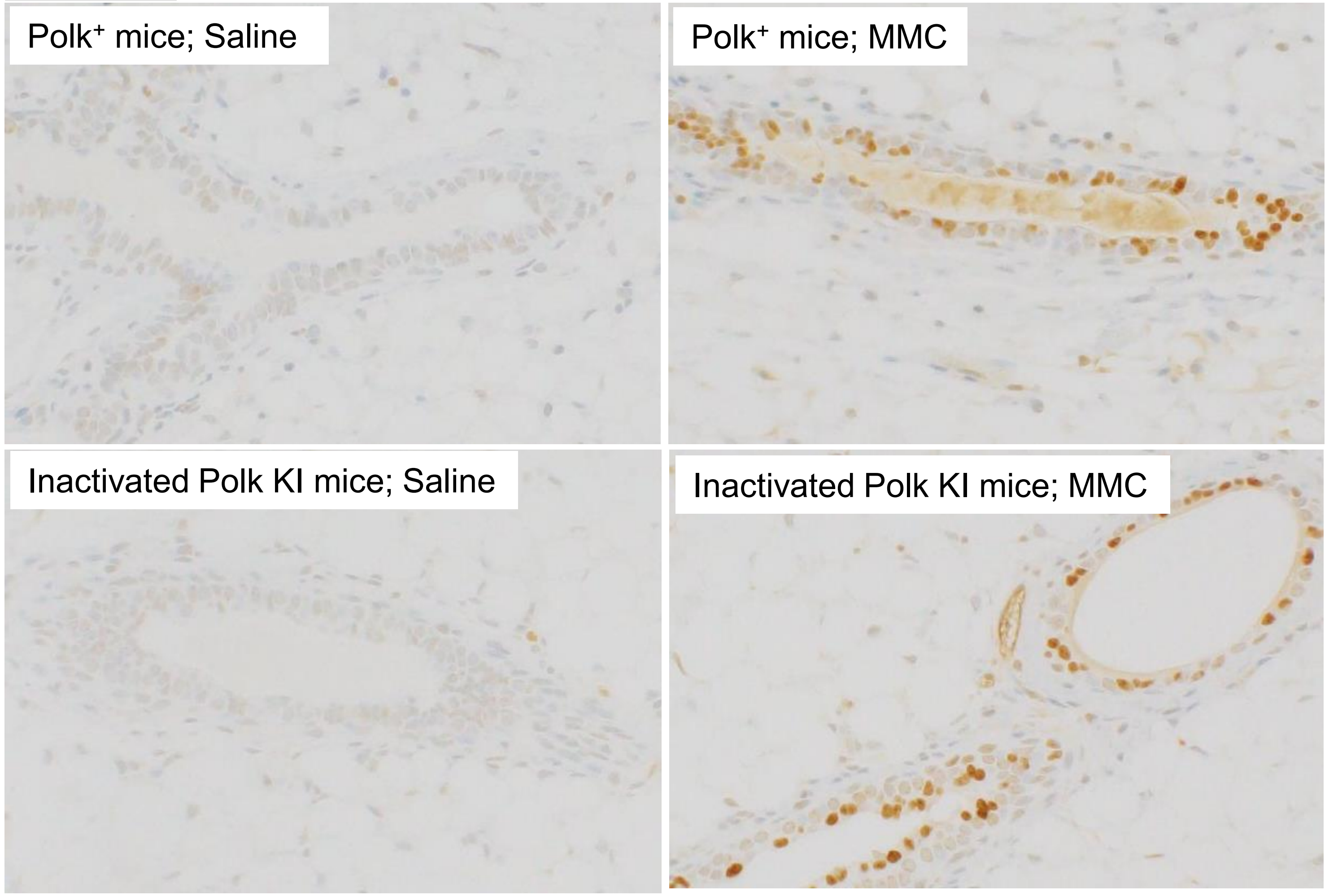

Mice;
Inactivated Polk KI mice, Polk^{+} mice

Treatment;
Saline: Saline x 5 days
MMC: Mitomycin C 1 mg/kg x 5 days

Staining;
HE; hematoxylin-eosin
γH2AX; γH2AX immunohistochemical stain

Bar represents 50 μm

Supplementary S8

Skin vascular endothelium

HE

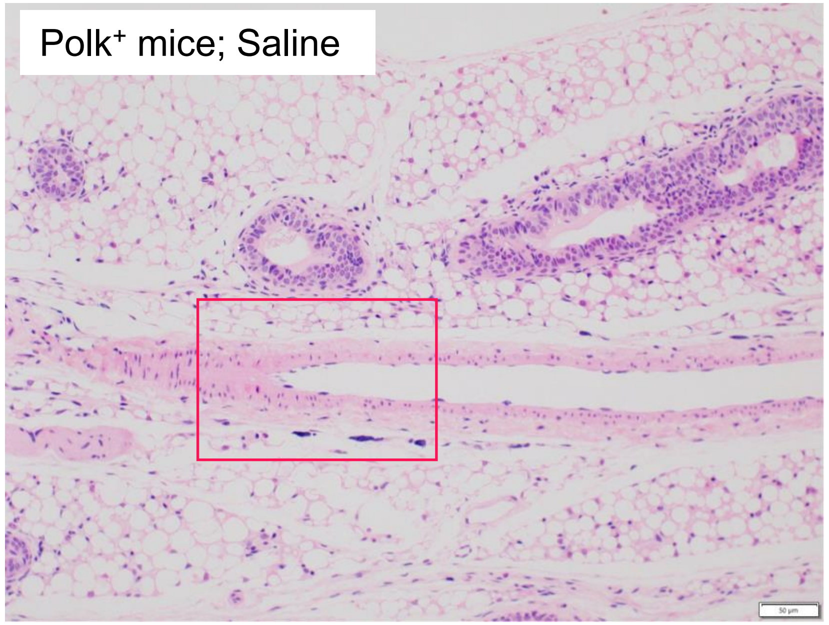

γH2AX

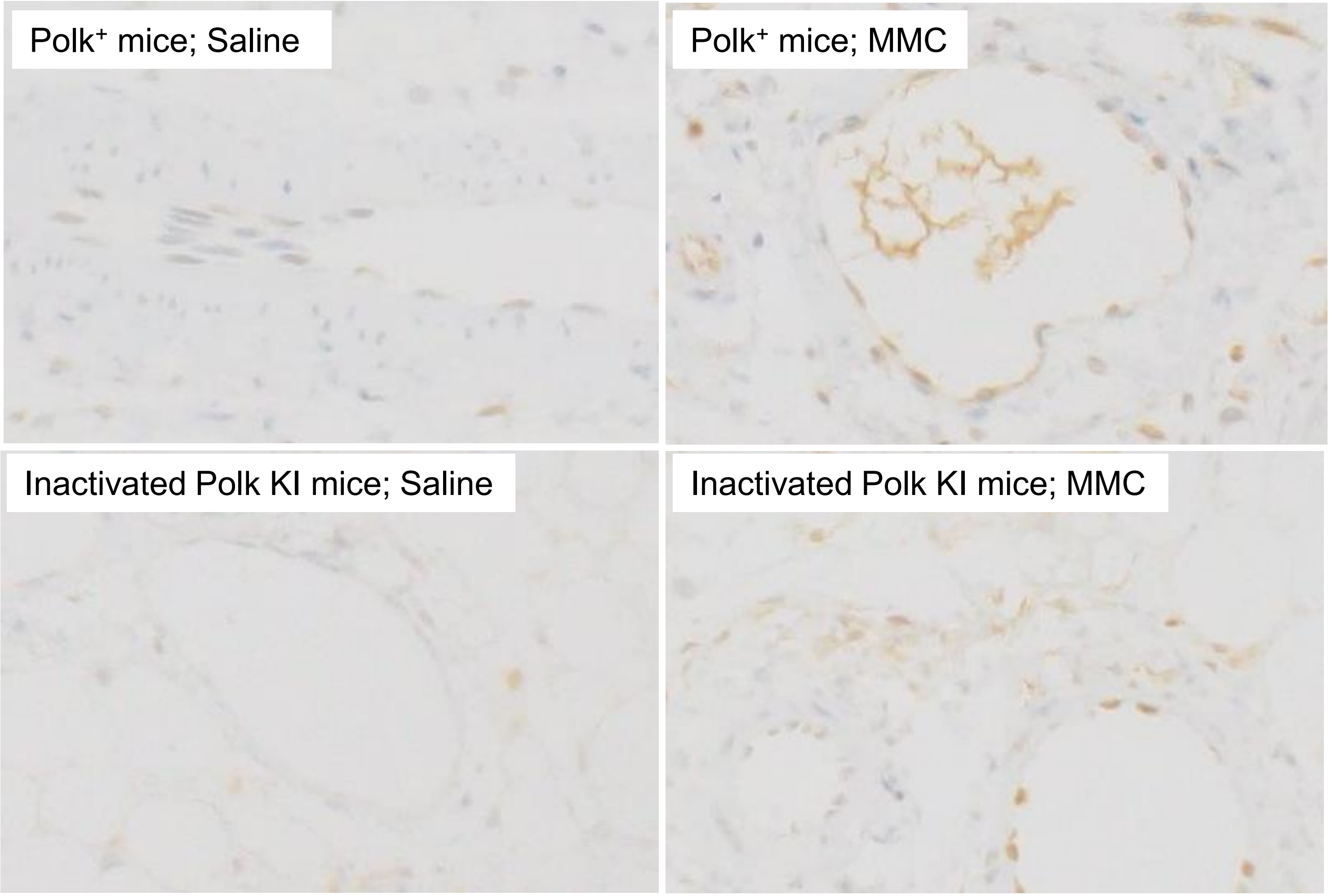

Mice;
Inactivated Polk KI mice, Polk⁺ mice

Treatment;
Saline: Saline x 5 days
MMC: Mitomycin C 1 mg/kg x 5 days

Staining;
HE; hematoxylin-eosin
γH2AX; γH2AX immunohistochemical stain

Bar represents 50 μm

Supplementary S9

Seminal vesicle

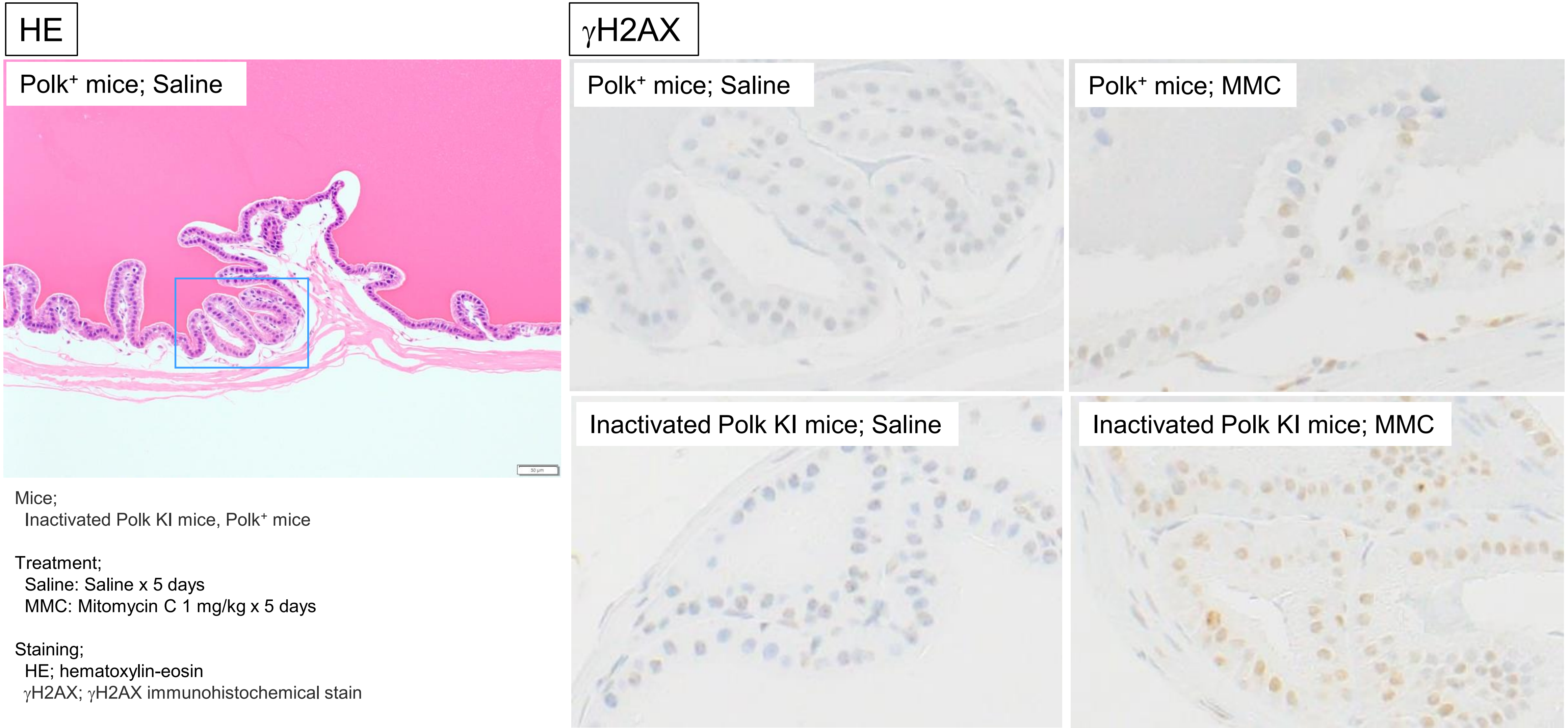

Mice;
Inactivated Polk KI mice, Polk^{+} mice

Treatment;
Saline: Saline x 5 days
MMC: Mitomycin C 1 mg/kg x 5 days

Staining;
HE; hematoxylin-eosin
γH2AX; γH2AX immunohistochemical stain

Bar represents 50 μm

Supplementary S10

Spleen

HE

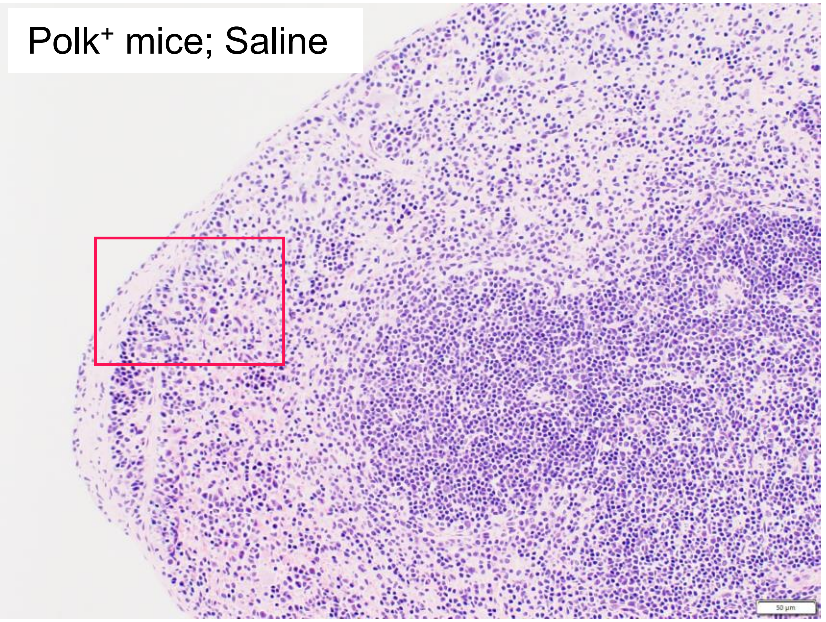

γH2AX

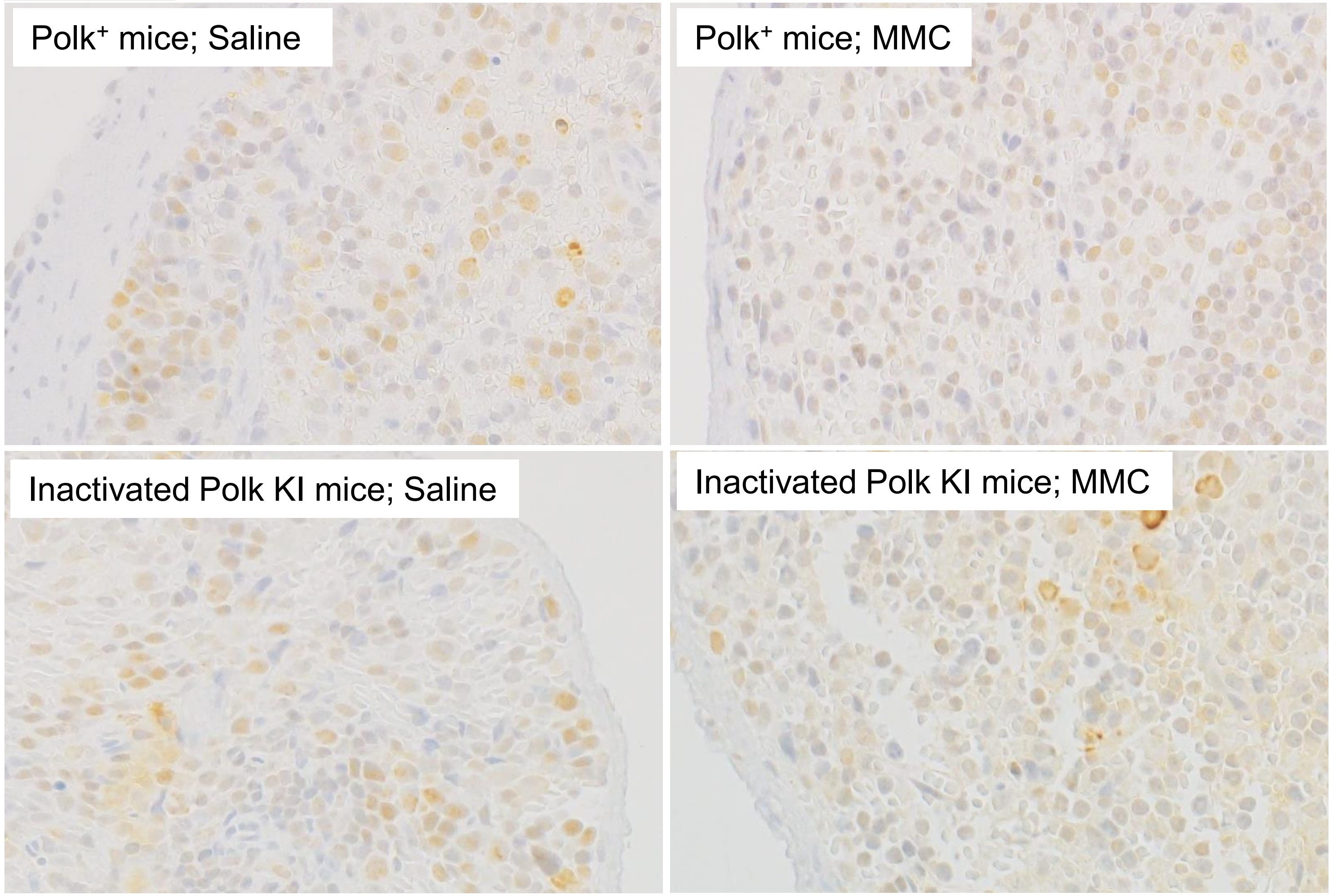

Mice;
Inactivated Polk KI mice, Polk$^+$ mice

Treatment;
Saline: Saline x 5 days
MMC: Mitomycin C 1 mg/kg x 5 days

Staining;
HE; hematoxylin-eosin
γH2AX; γH2AX immunohistochemical stain

Bar represents 50 μm

Supplementary S11

Ileum

HE

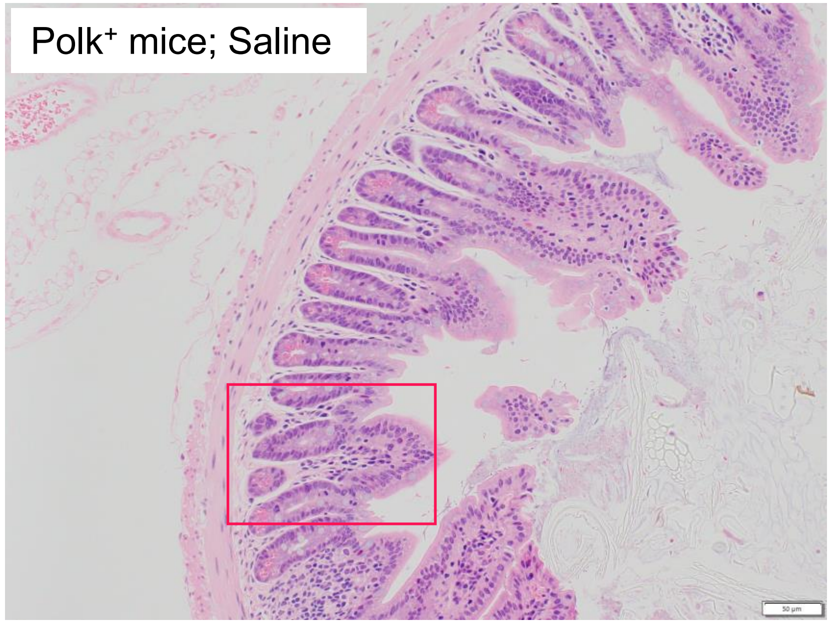

Mice;
Inactivated Polk KI mice, Polk$^+$ mice

Treatment;
Saline: Saline x 5 days
MMC: Mitomycin C 1 mg/kg x 5 days

Staining;
HE; hematoxylin-eosin
γH2AX; γH2AX immunohistochemical stain

Bar represents 50 μm

γH2AX

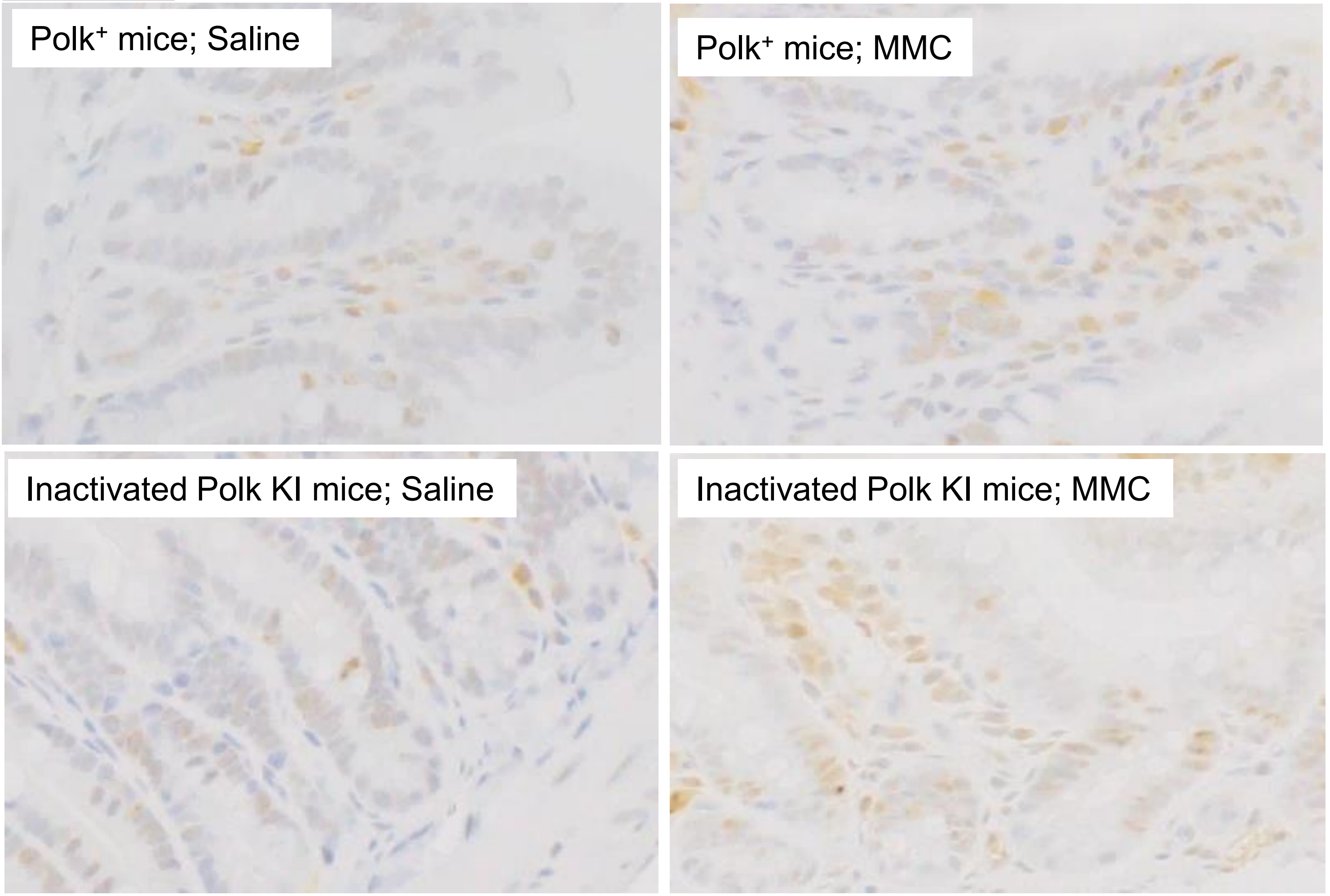

Supplementary S12

Epididymis

HE

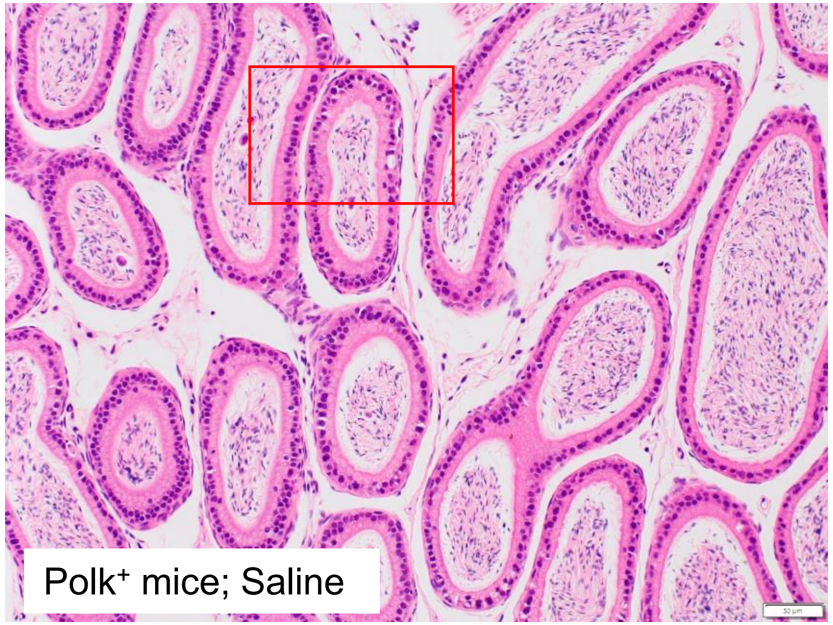

γH2AX

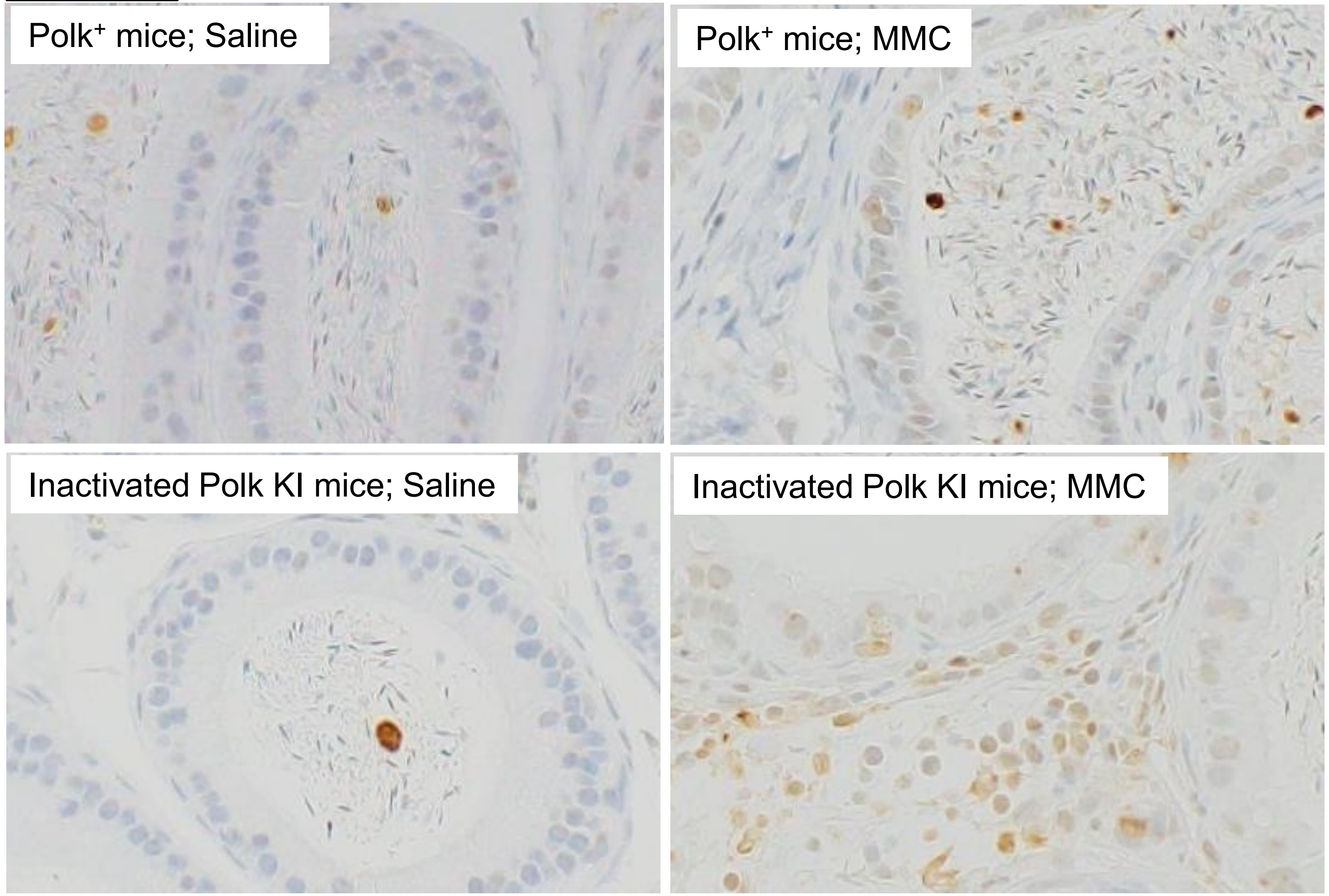

Mice;
- Inactivated Polk KI mice, Polk+ mice

Treatment;
- Saline: Saline x 5 days
- MMC: Mitomycin C 1 mg/kg x 5 days

Staining;
- HE; hematoxylin-eosin
- γH2AX; γH2AX immunohistochemical stain

Bar represents 50 μm

Supplementary S13

lung

HE

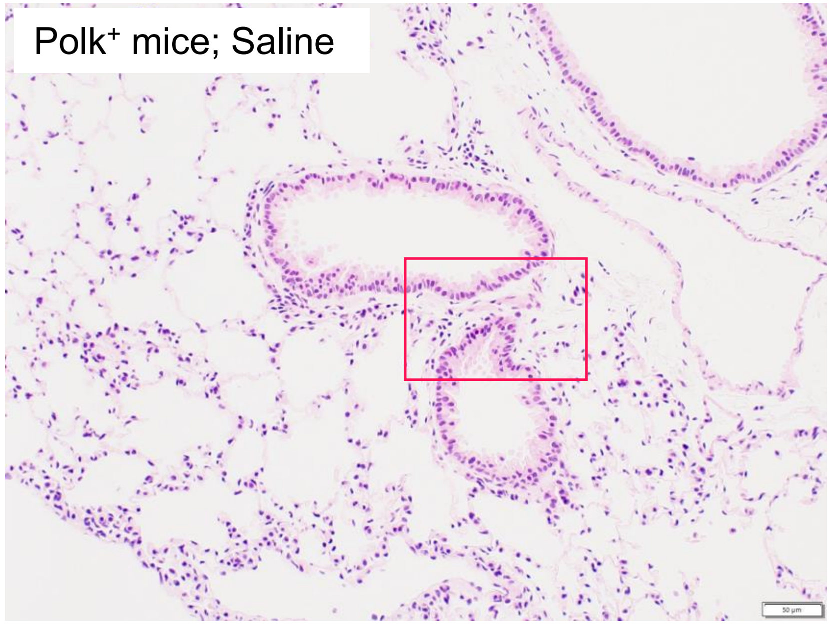

γH2AX

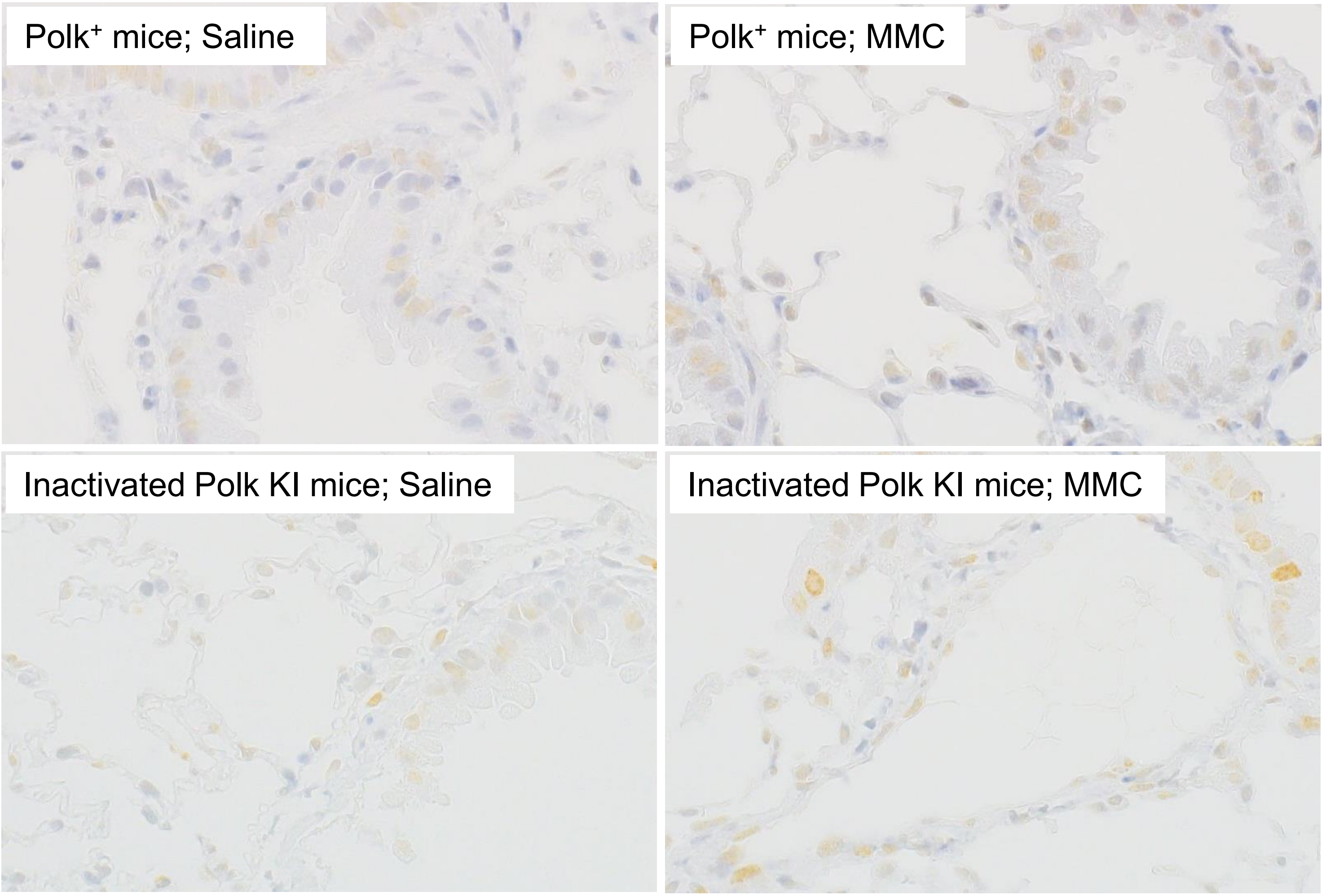

Mice;
Inactivated Polk KI mice, Polk^{+} mice

Treatment;
Saline: Saline x 5 days
MMC: Mitomycin C 1 mg/kg x 5 days

Staining;
HE; hematoxylin-eosin
γH2AX; γH2AX immunohistochemical stain

Bar represents 50 μm

Supplementary S14

kidney

HE

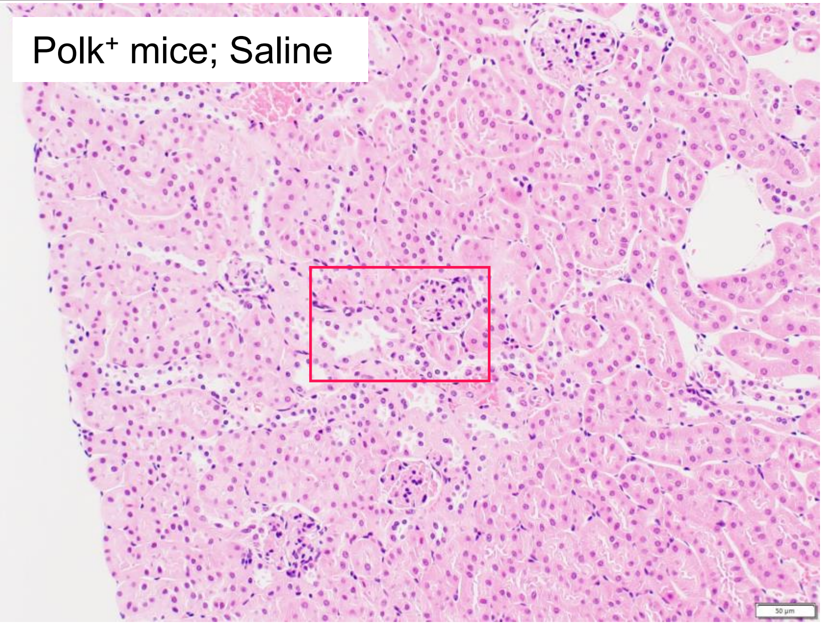

γH2AX

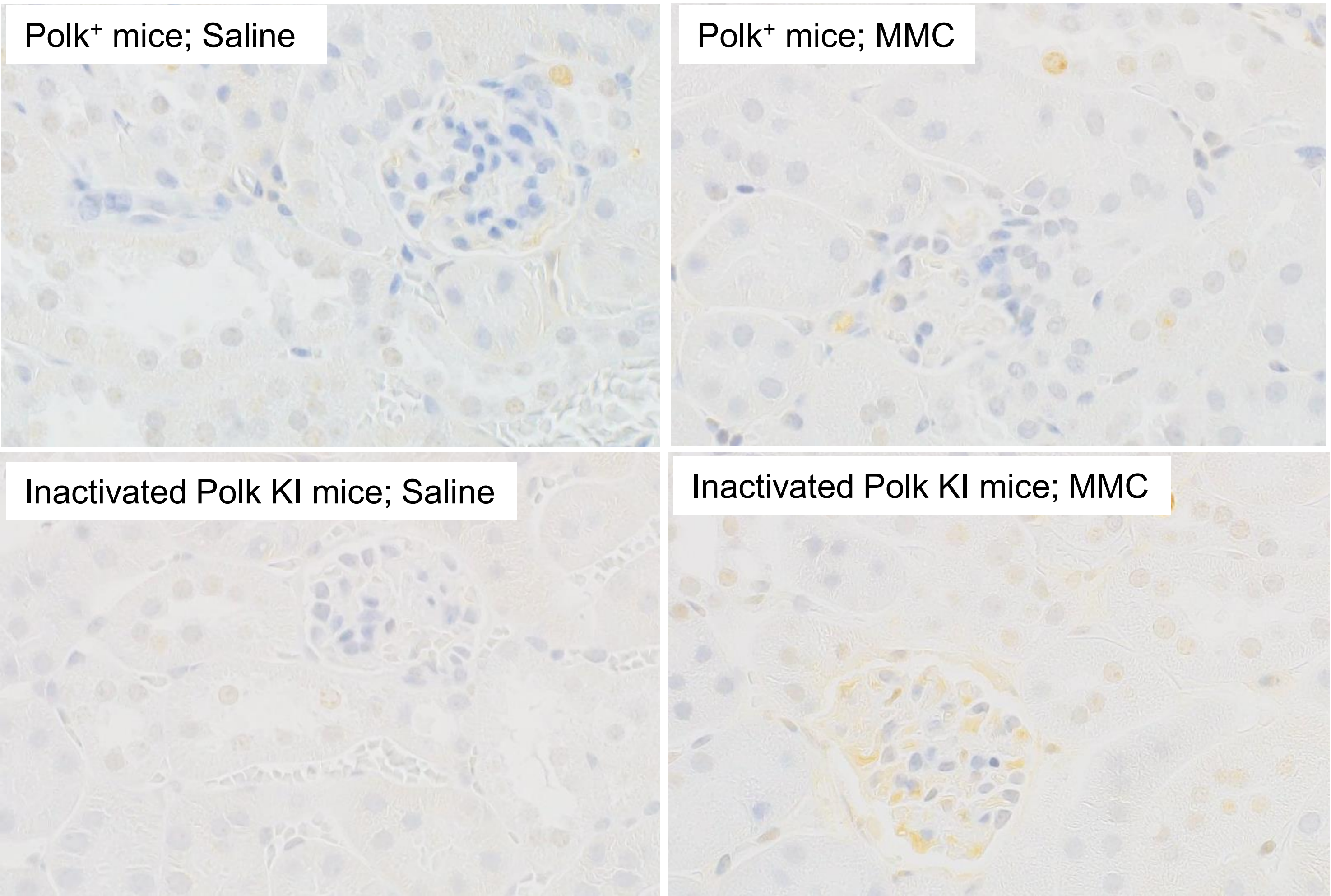

Mice;
Inactivated Polk KI mice, Polk+ mice

Treatment;
Saline: Saline x 5 days
MMC: Mitomycin C 1 mg/kg x 5 days

Staining;
HE; hematoxylin-eosin
γH2AX; γH2AX immunohistochemical stain

Bar represents 50 μm

Supplementary S15

Skin (hair follicle)

HE

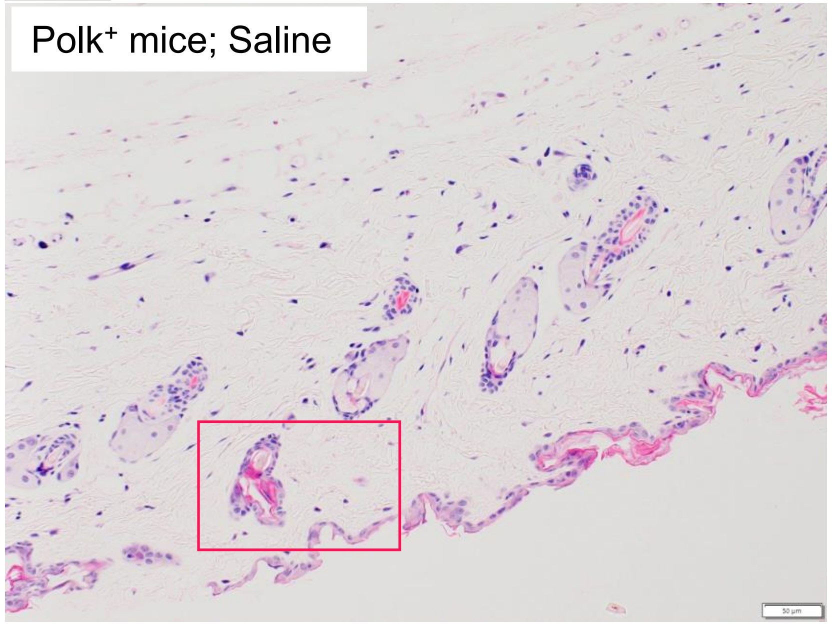

Mice;
Inactivated Polk KI mice, Polk^{+} mice

Treatment;
Saline: Saline x 5 days
MMC: Mitomycin C 1 mg/kg x 5 days

Staining;
HE; hematoxylin-eosin
γH2AX; γH2AX immunohistochemical stain

Bar represents 50 μm

γH2AX

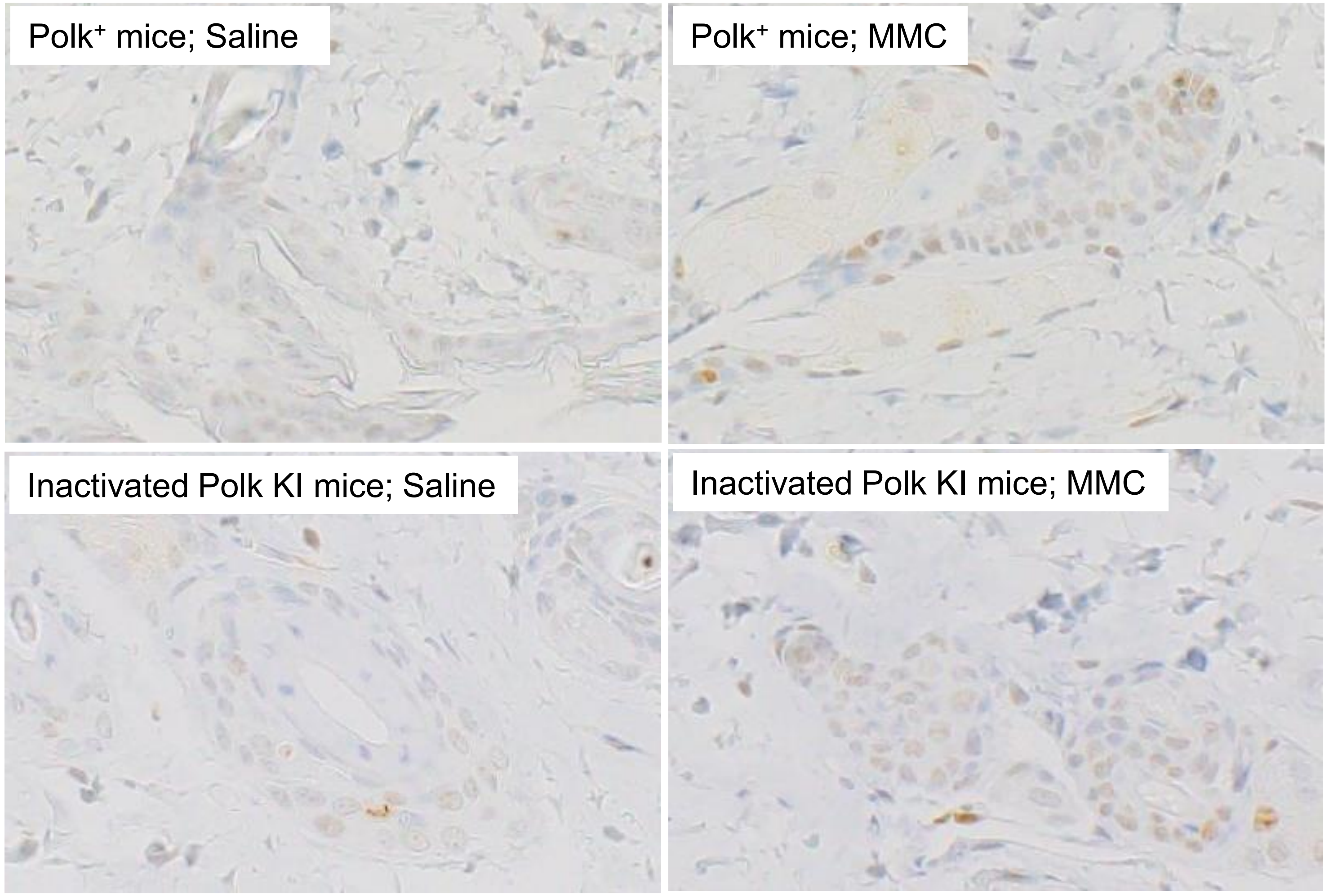

Supplementary S16

Cornea

HE

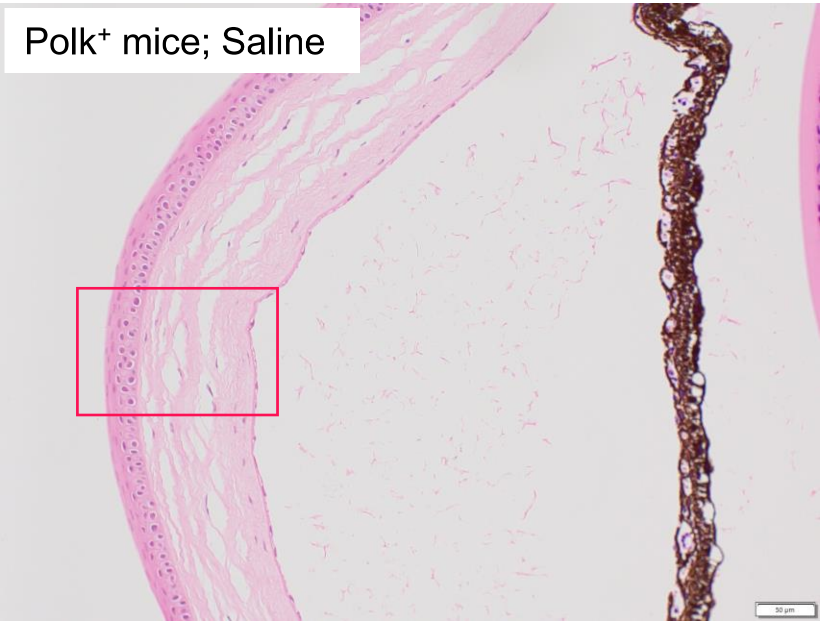

Mice;
Inactivated Polk KI mice, Polk+ mice

Treatment;
Saline: Saline x 5 days
MMC: Mitomycin C 1 mg/kg x 5 days

Staining;
HE; hematoxylin-eosin
γH2AX; γH2AX immunohistochemical stain

Bar represents 50 μm

γH2AX

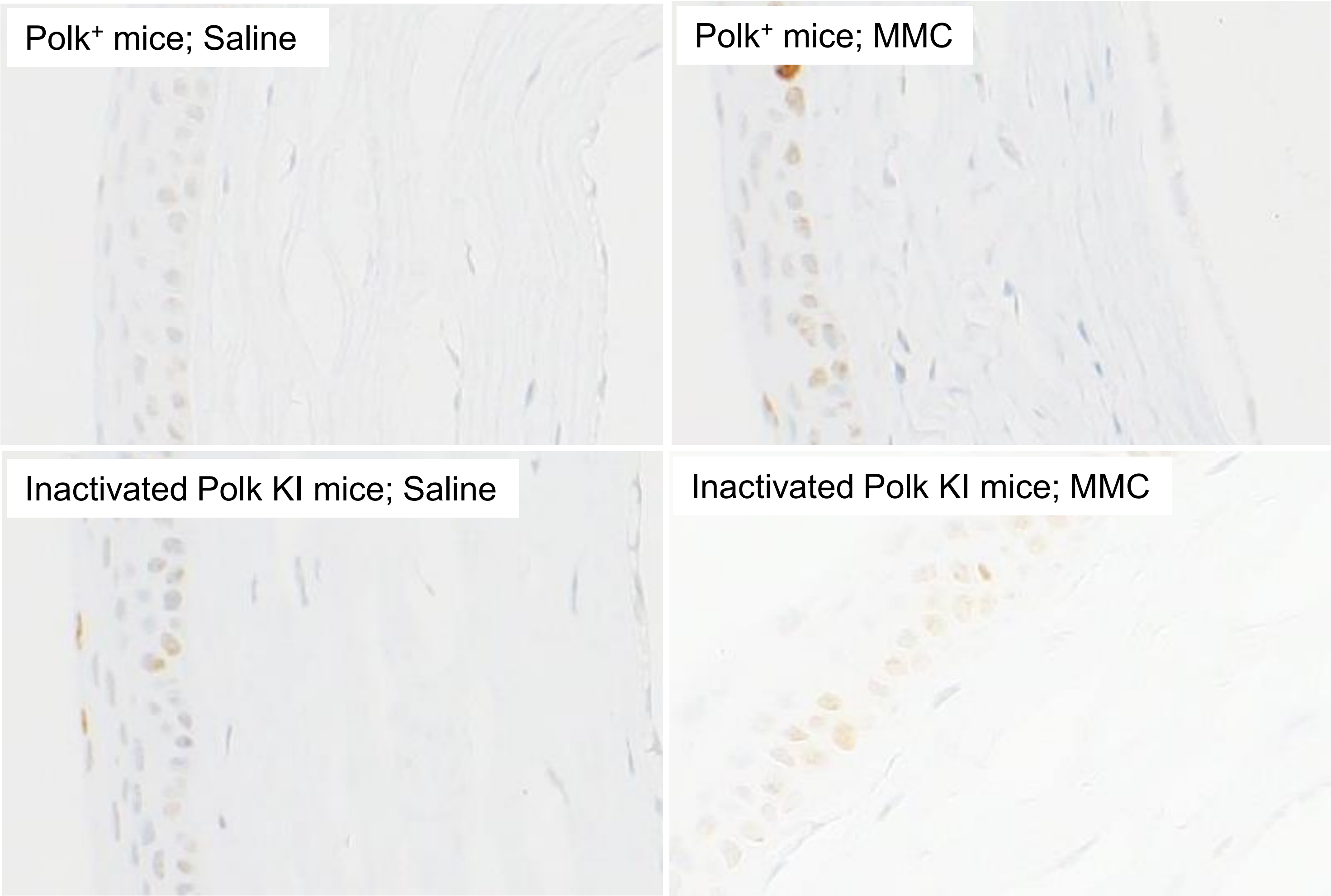

Supplementary S17

Epidermis

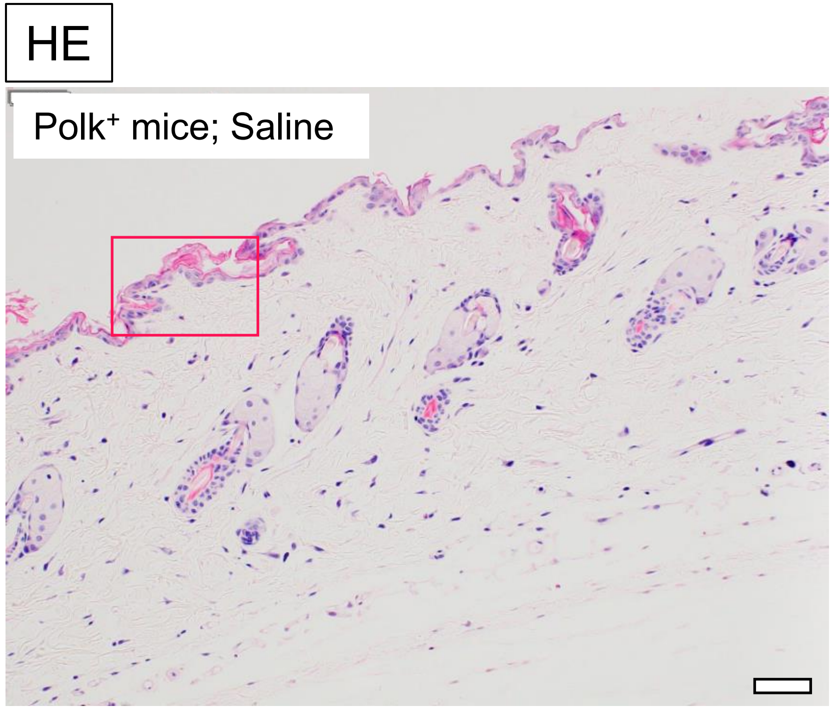

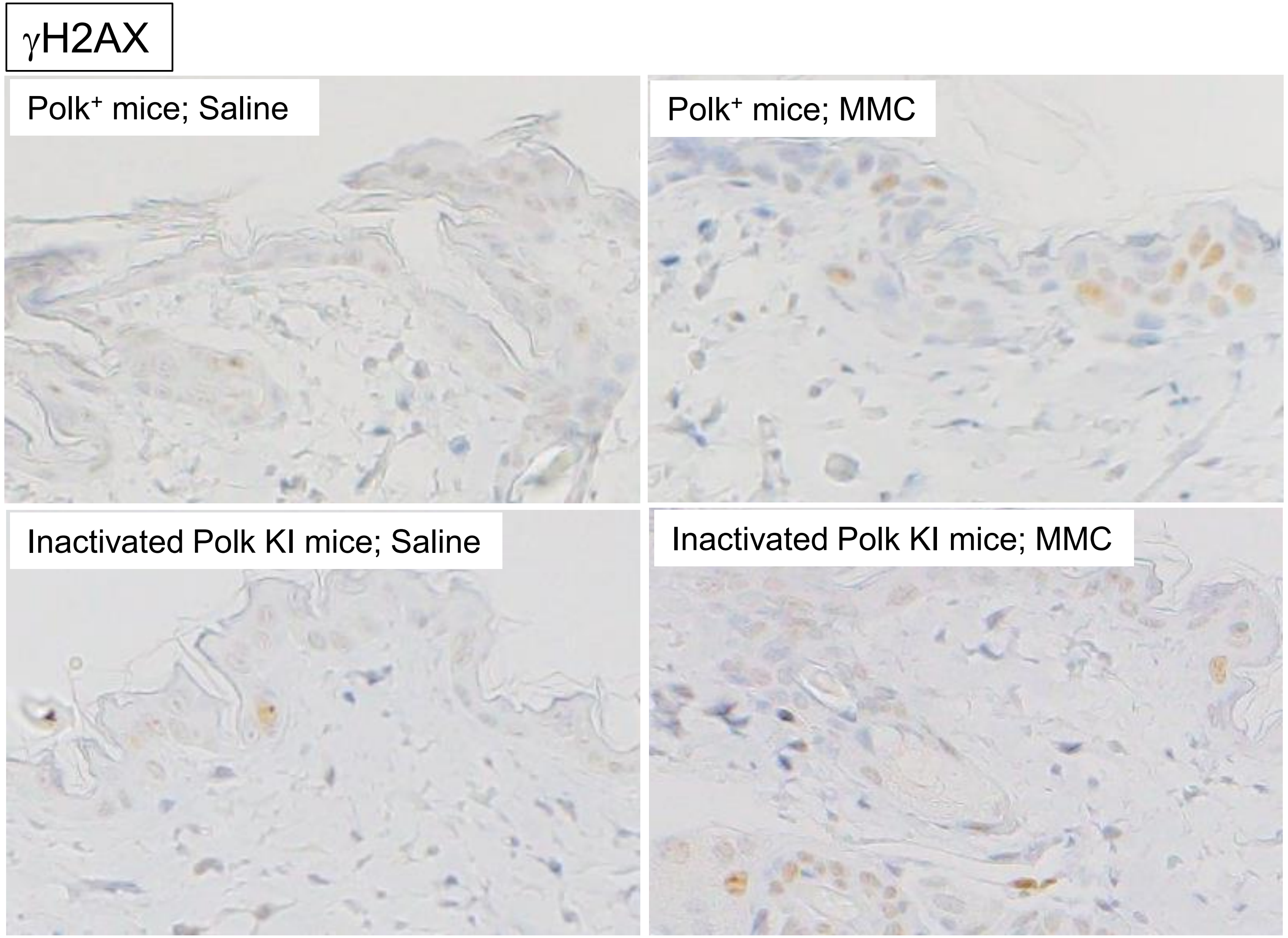

Mice;
Inactivated Polk KI mice, Polk^{+} mice

Treatment;
Saline: Saline x 5 days
MMC: Mitomycin C 1 mg/kg x 5 days

Staining;
HE; hematoxylin-eosin
γH2AX; γH2AX immunohistochemical stain

Bar represents 50 μm

Supplementary S18

Colon

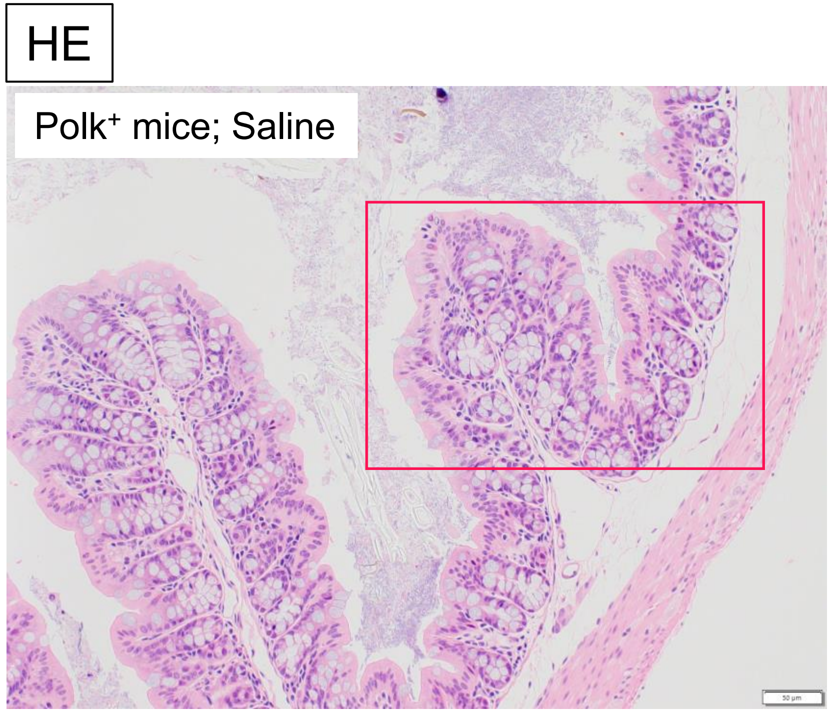

Mice;
Inactivated Polk KI mice, Polk⁺ mice

Treatment;
Saline: Saline x 5 days
MMC: Mitomycin C 1 mg/kg x 5 days

Staining;
HE; hematoxylin-eosin
γH2AX; γH2AX immunohistochemical stain

Bar represents 50 μm

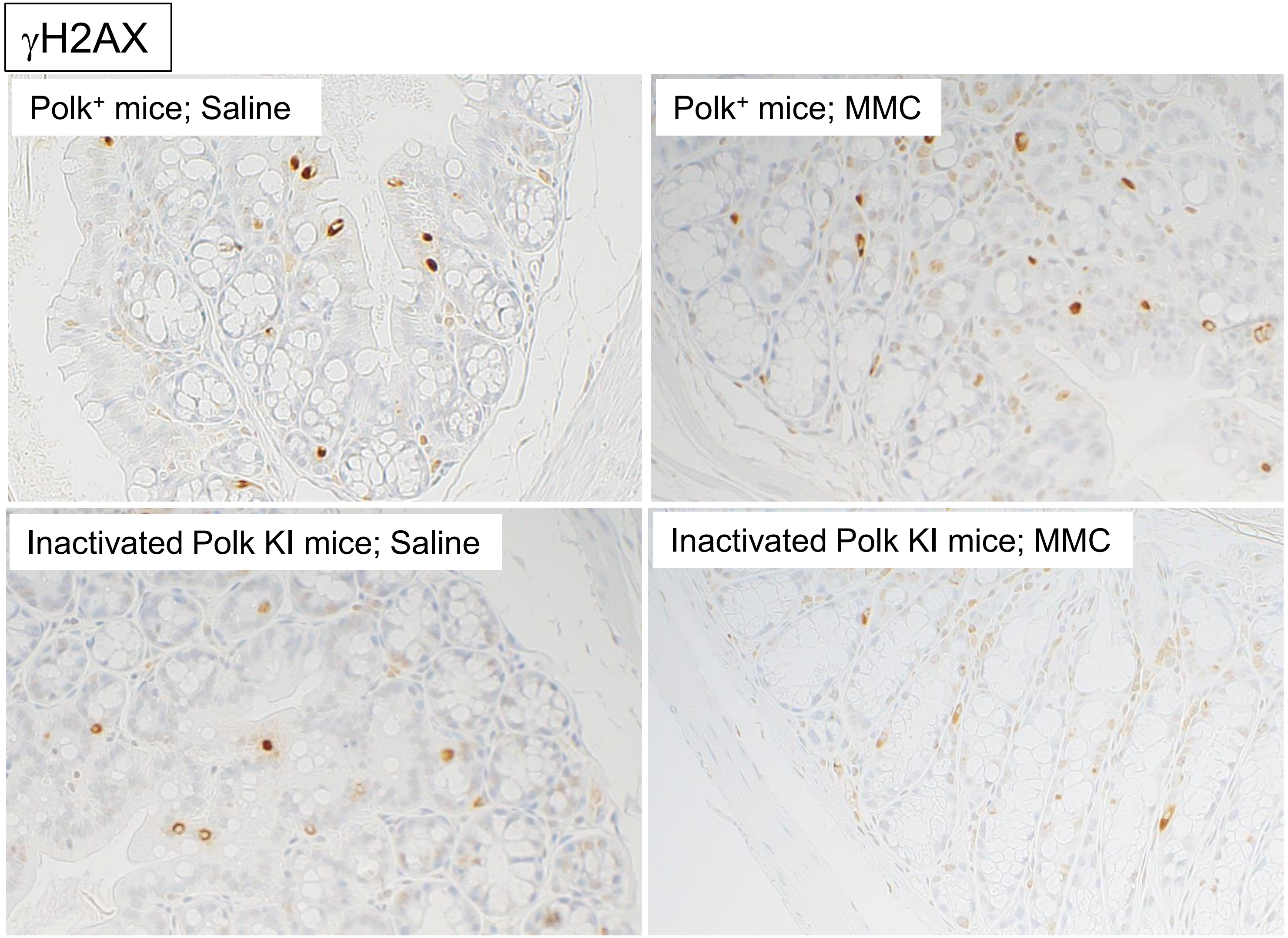

Supplementary S19

Tongue skeletal muscle

HE

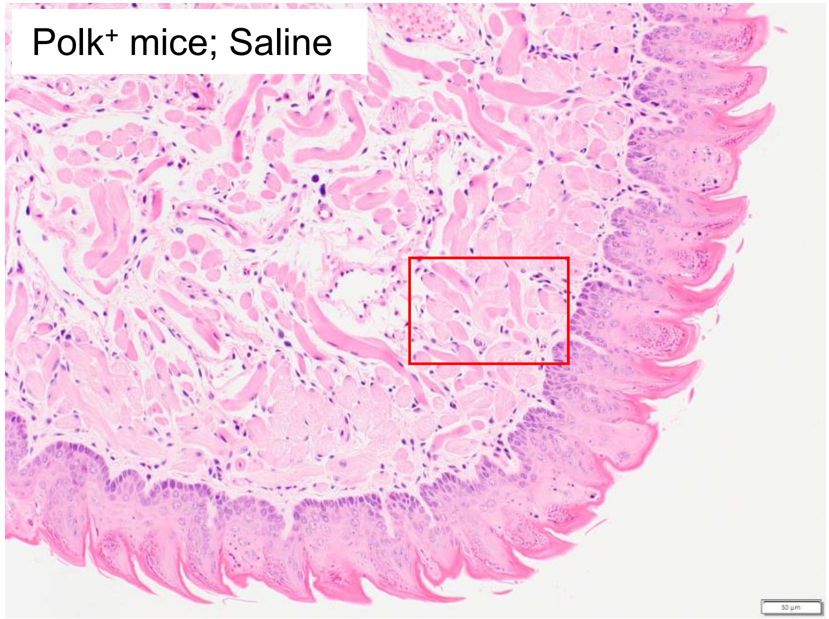

γH2AX

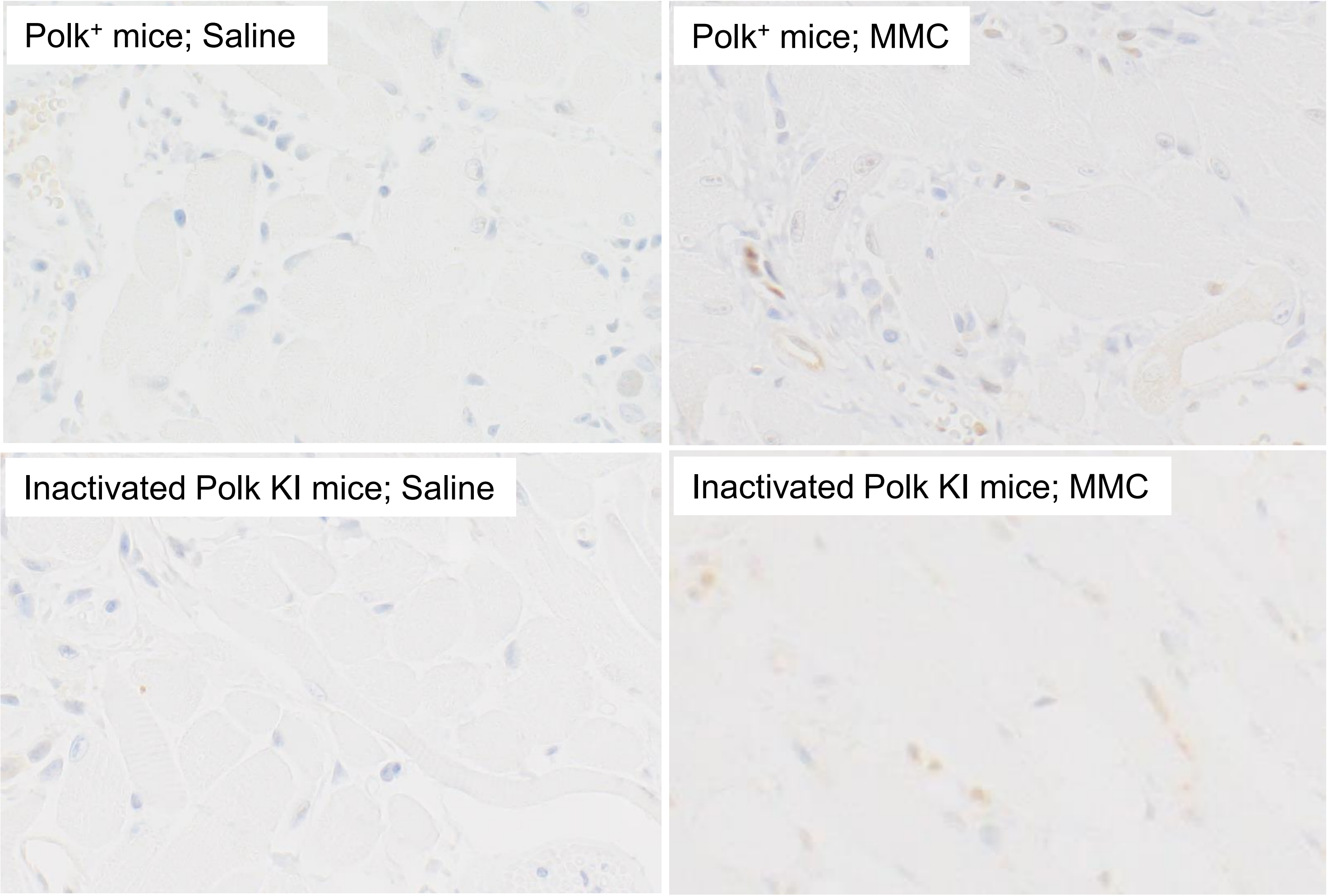

Mice;
Inactivated Polk KI mice, Polk⁺ mice

Treatment;
Saline: Saline x 5 days
MMC: Mitomycin C 1 mg/kg x 5 days

Staining;
HE; hematoxylin-eosin
γH2AX; γH2AX immunohistochemical stain

Bar represents 50 μm

Supplementary S20

Heart

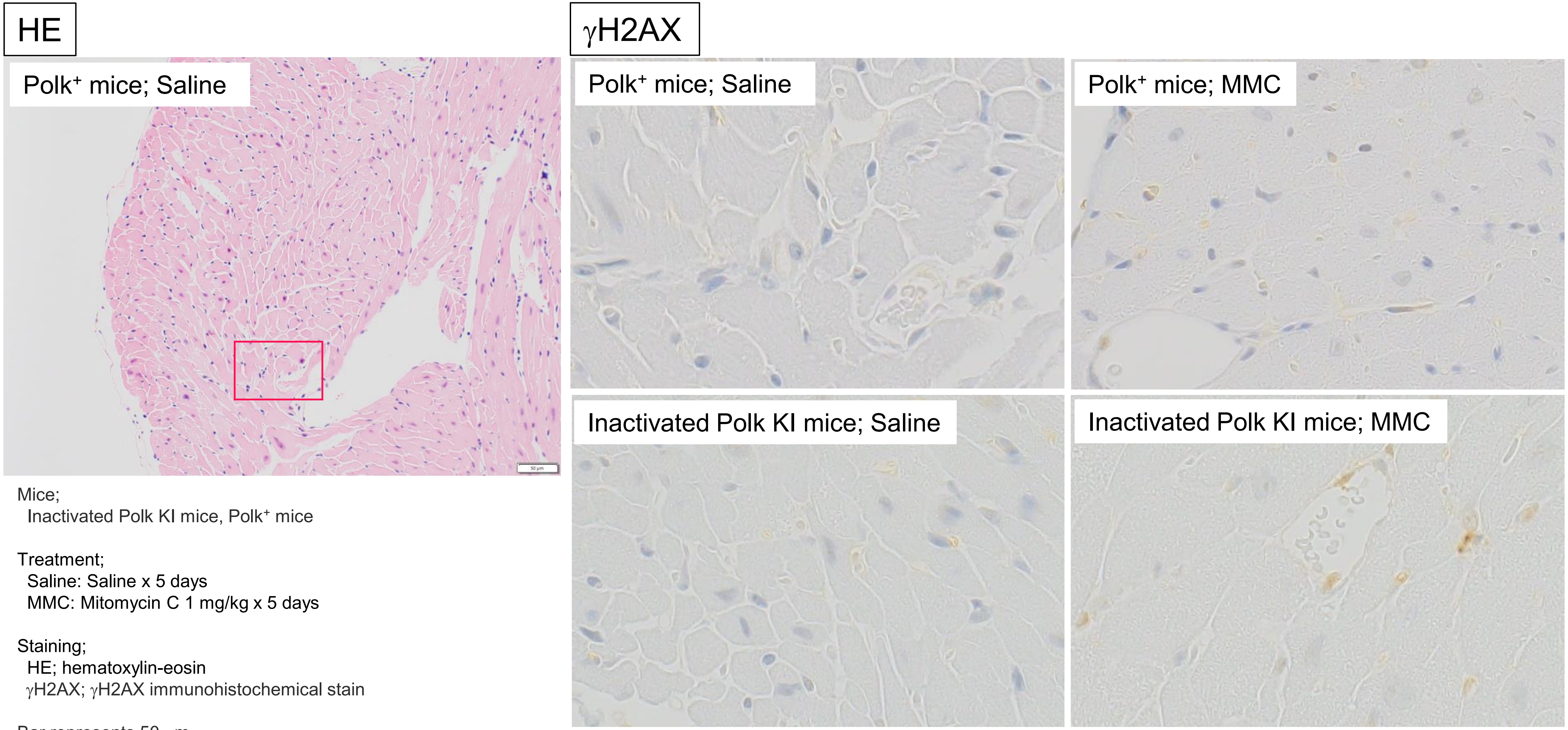

Mice;
Inactivated Polk KI mice, Polk⁺ mice

Treatment;
Saline: Saline x 5 days
MMC: Mitomycin C 1 mg/kg x 5 days

Staining;
HE; hematoxylin-eosin
γH2AX; γH2AX immunohistochemical stain

Bar represents 50 μm

Supplementary S21

Tracheal epithelium

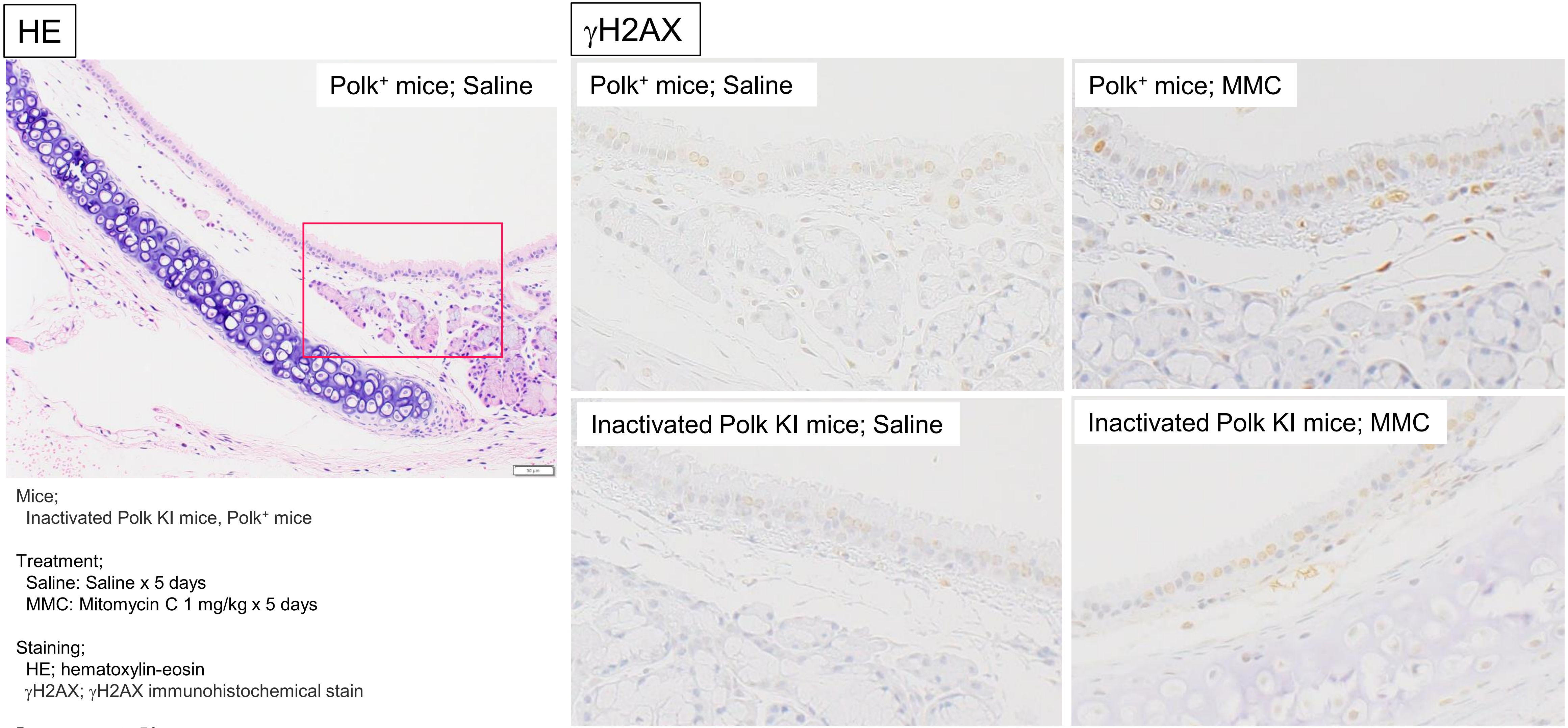

Mice;
Inactivated Polk KI mice, Polk⁺ mice

Treatment;
Saline: Saline x 5 days
MMC: Mitomycin C 1 mg/kg x 5 days

Staining;
HE; hematoxylin-eosin
γH2AX; γH2AX immunohistochemical stain

Bar represents 50 μm

Supplementary S22

Harder gland

HE

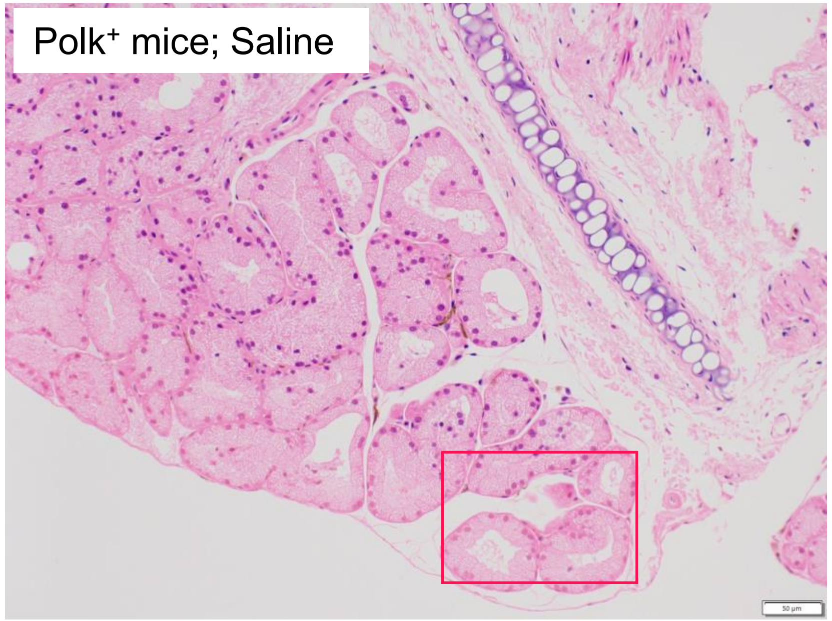

γH2AX

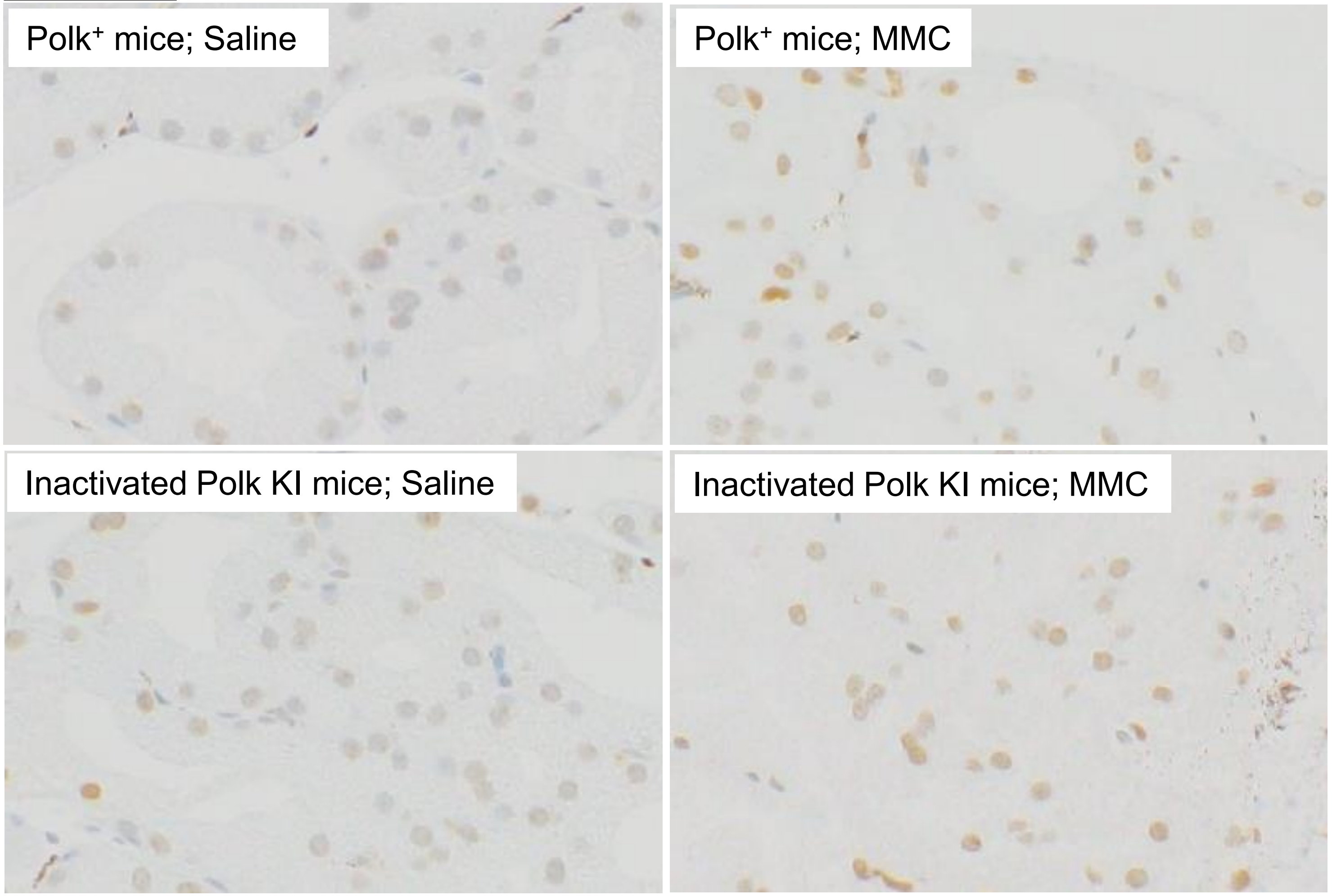

Mice;
Inactivated Polk KI mice, Polk^{+} mice

Treatment;
Saline: Saline x 5 days
MMC: Mitomycin C 1 mg/kg x 5 days

Staining;
HE; hematoxylin-eosin
γH2AX; γH2AX immunohistochemical stain

Bar represents 50 μm

Supplementary S23

Pancreas

HE

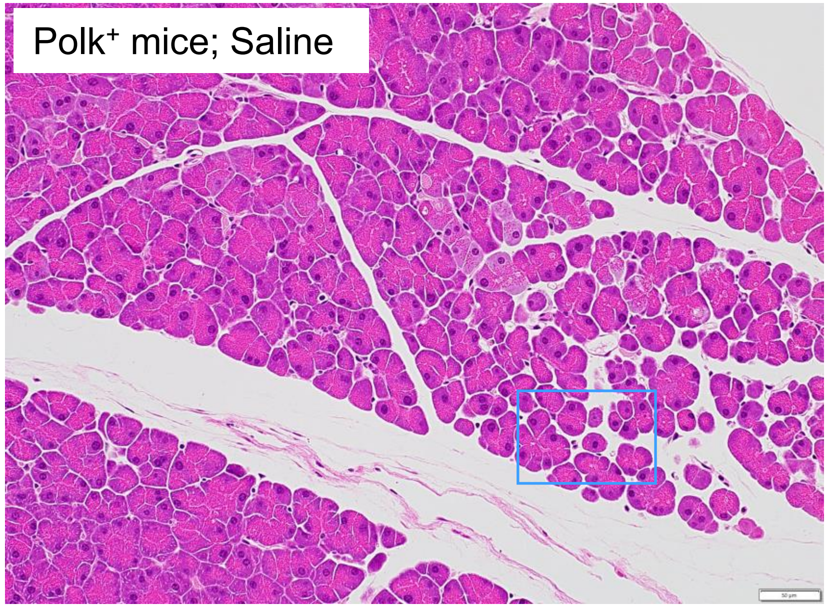

γH2AX

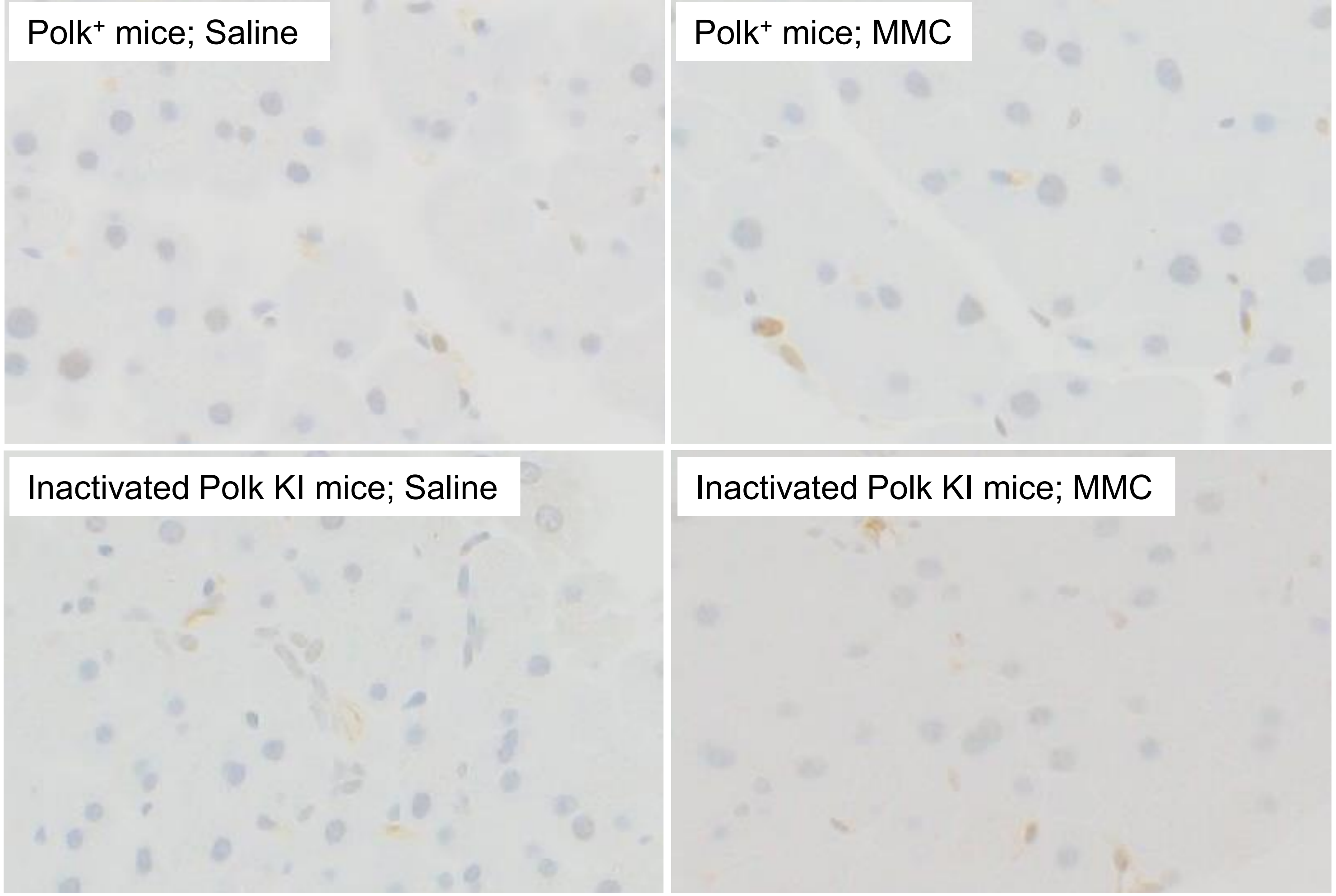

Mice;
Inactivated Polk KI mice, Polk+ mice

Treatment;
Saline: Saline x 5 days
MMC: Mitomycin C 1 mg/kg x 5 days

Staining;
HE; hematoxylin-eosin
γH2AX; γH2AX immunohistochemical stain

Bar represents 50 μm

Supplementary S24

Brainstem

HE

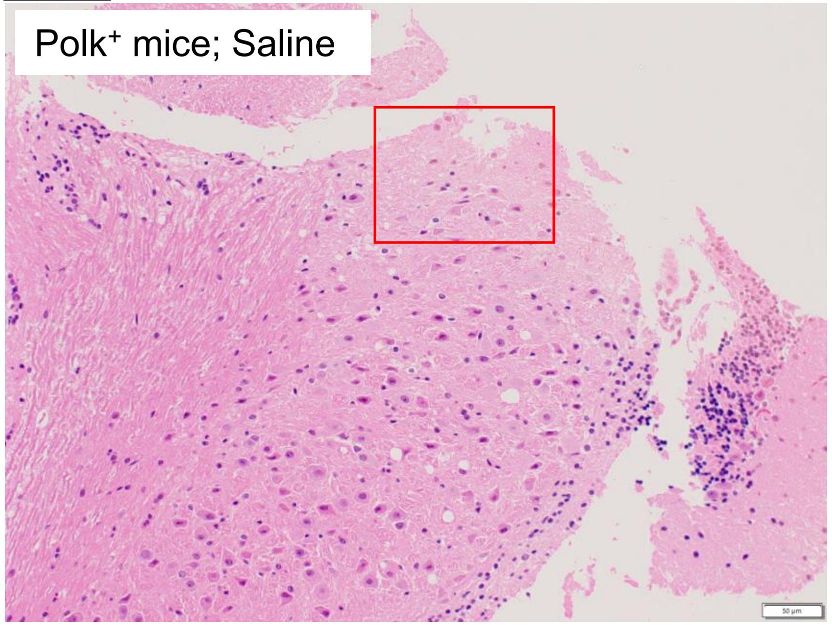

γH2AX

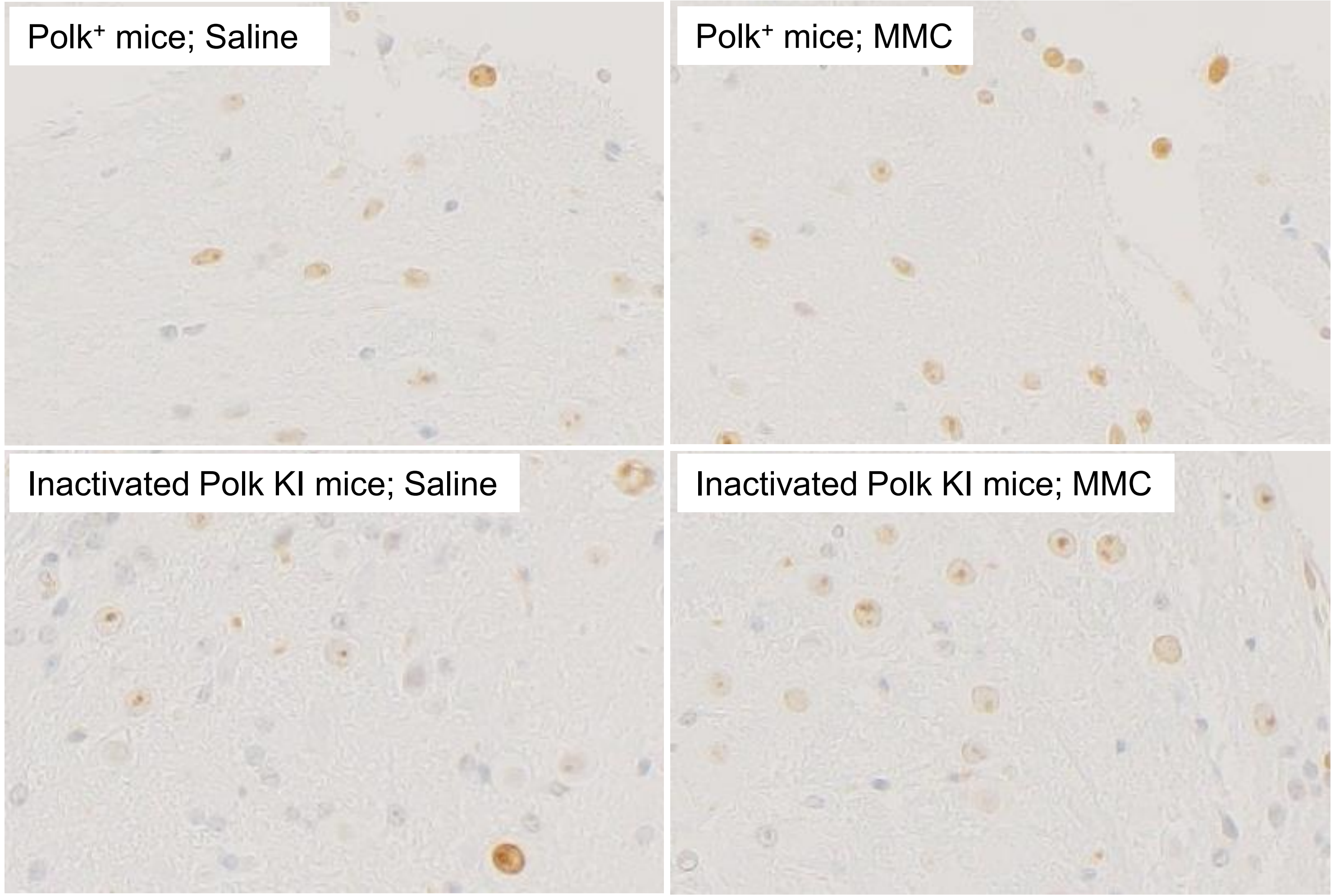

Mice;
Inactivated Polk KI mice, Polk$^+$ mice

Treatment;
Saline: Saline x 5 days
MMC: Mitomycin C 1 mg/kg x 5 days

Staining;
HE; hematoxylin-eosin
γH2AX; γH2AX immunohistochemical stain

Bar represents 50 µm

Supplementary S25

Mesenteric lymph node

HE

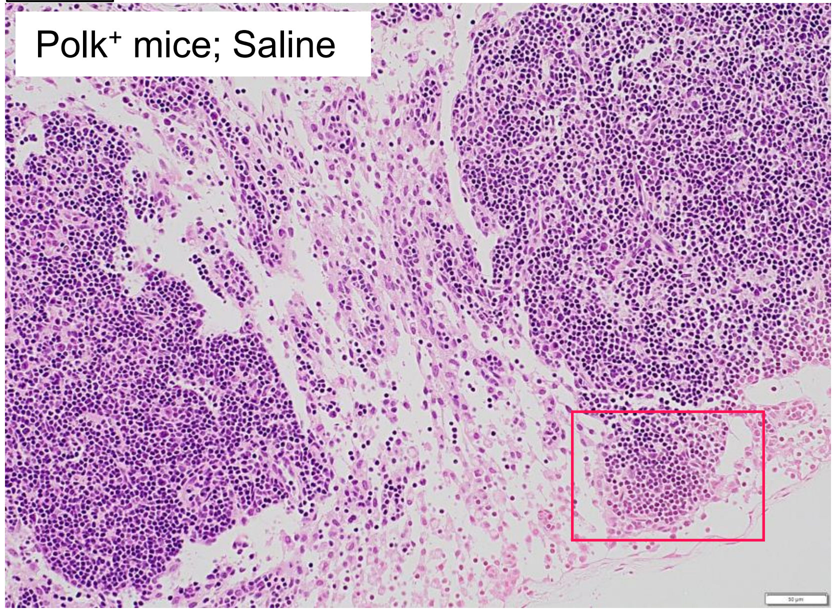

γH2AX

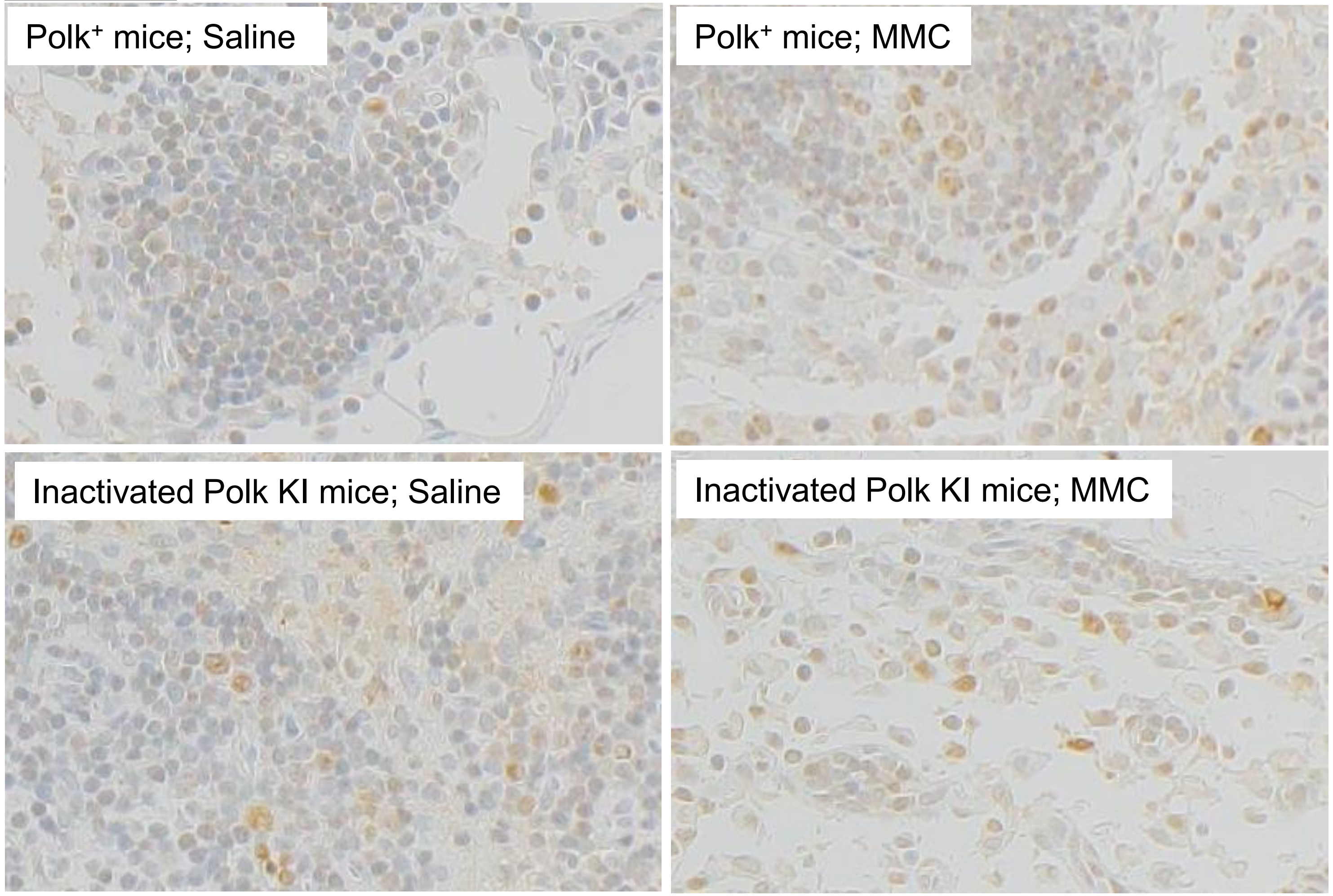

Mice;
Inactivated Polk KI mice, Polk⁺ mice

Treatment;
Saline: Saline x 5 days
MMC: Mitomycin C 1 mg/kg x 5 days

Staining;
HE; hematoxylin-eosin
γH2AX; γH2AX immunohistochemical stain

Bar represents 50 μm

Supplementary S26

Prostate

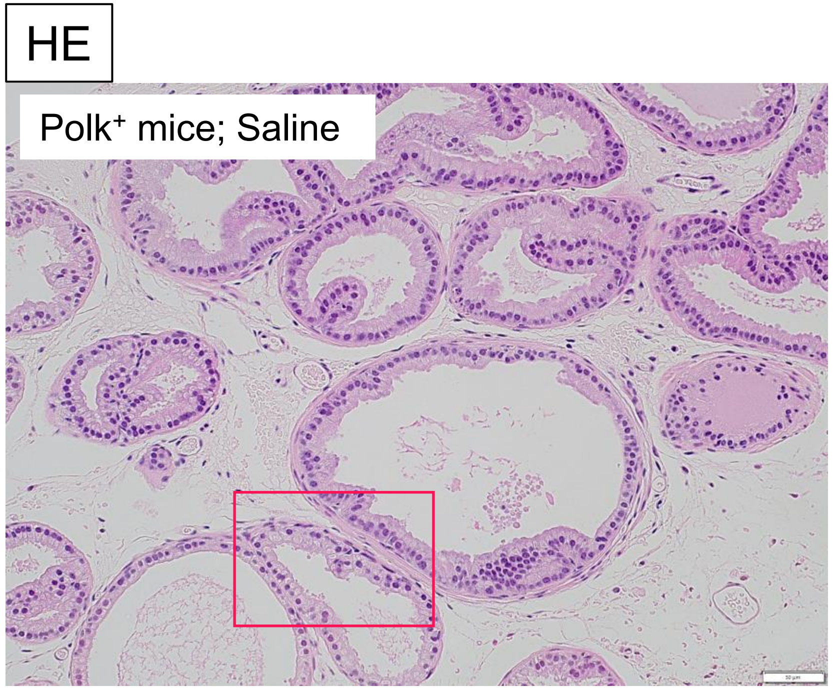

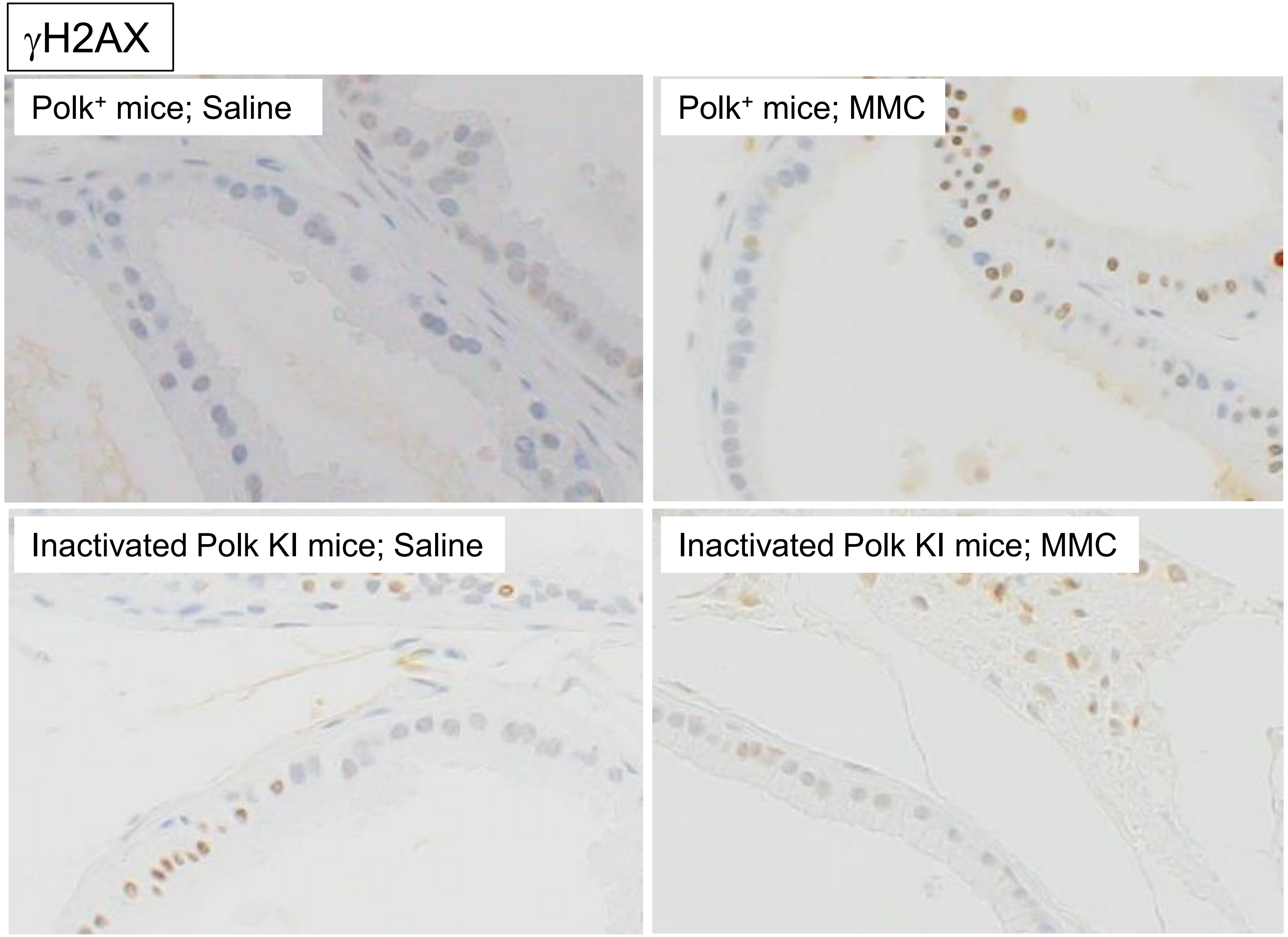

Mice;
  Inactivated Polk KI mice, Polk^{+} mice

Treatment;
  Saline: Saline x 5 days
  MMC: Mitomycin C 1 mg/kg x 5 days

Staining;
  HE; hematoxylin-eosin
  γH2AX; γH2AX immunohistochemical stain

Bar represents 50 μm

Supplementary S27

Cerebellum

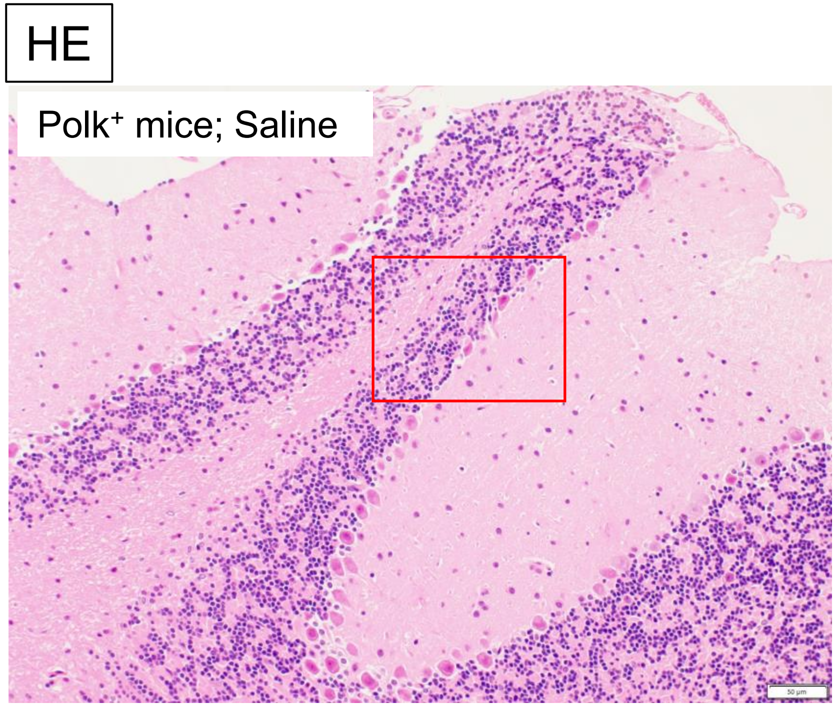

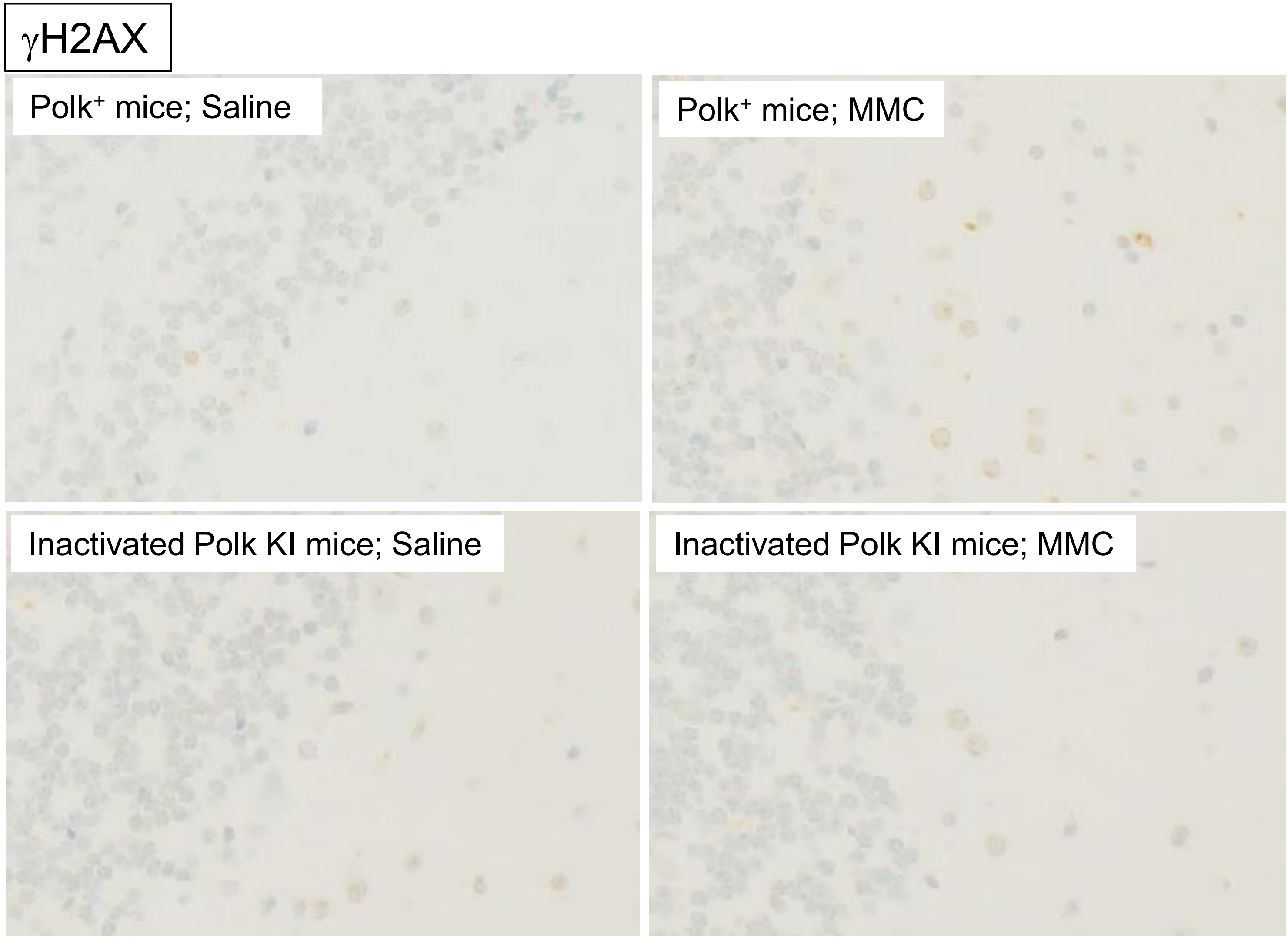

Mice;
Inactivated Polk KI mice, Polk^{+} mice

Treatment;
Saline: Saline x 5 days
MMC: Mitomycin C 1 mg/kg x 5 days

Staining;
HE; hematoxylin-eosin
γH2AX; γH2AX immunohistochemical stain

Bar represents 50 μm

Supplementary S28

Thymus

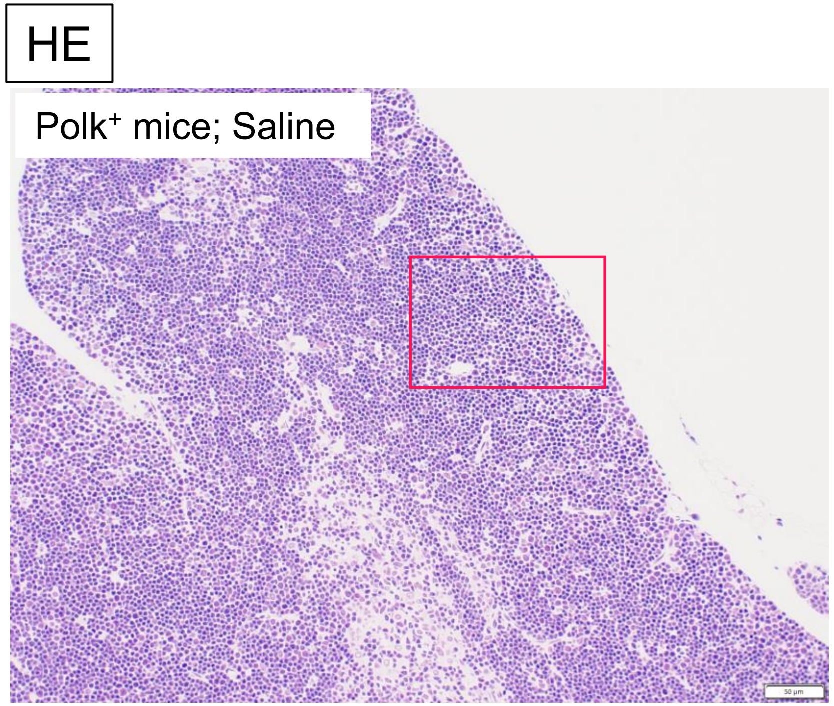

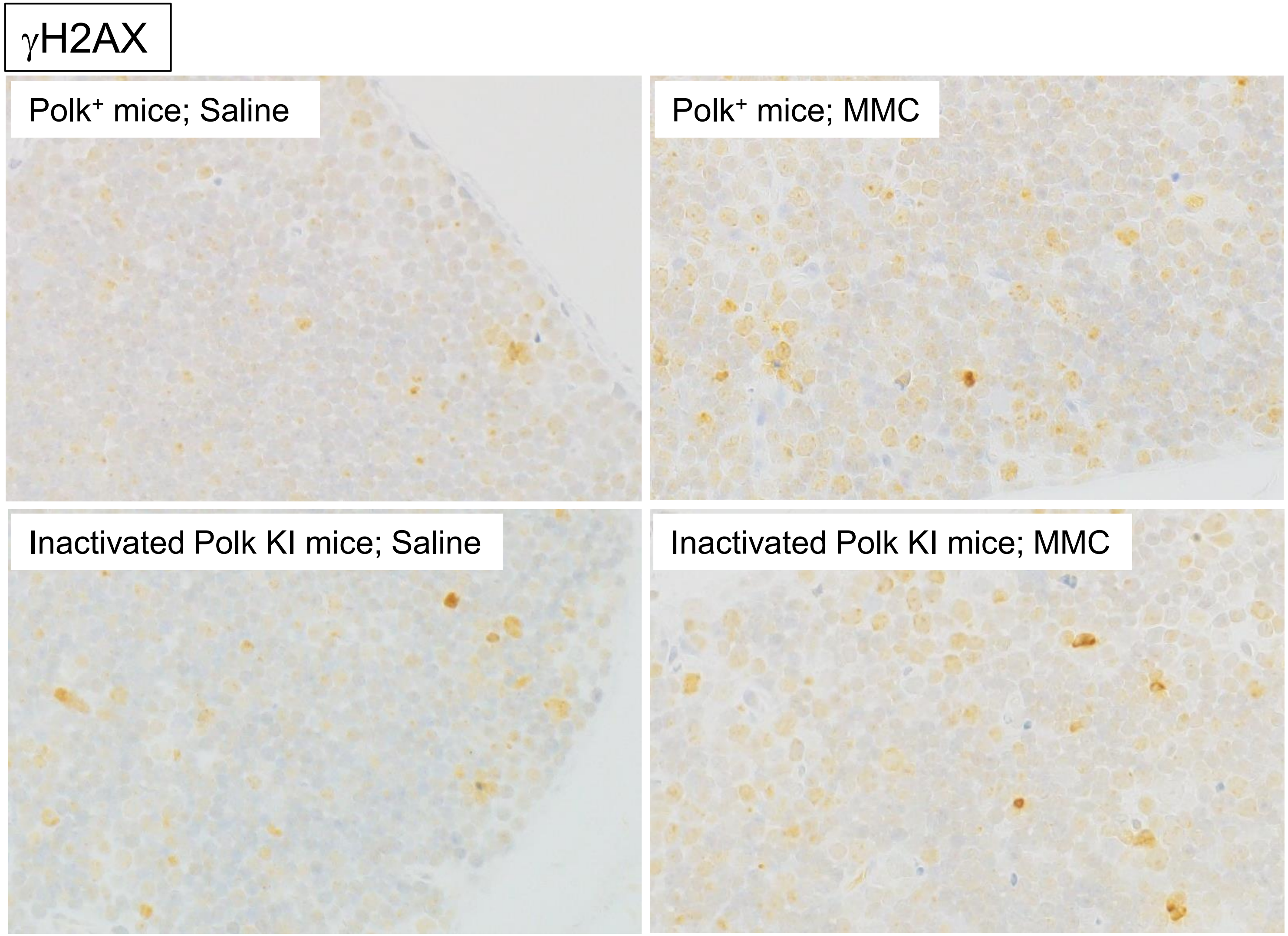

Mice;
Inactivated Polk KI mice, Polk^{+} mice

Treatment;
Saline: Saline x 5 days
MMC: Mitomycin C 1 mg/kg x 5 days

Staining;
HE; hematoxylin-eosin
γH2AX; γH2AX immunohistochemical stain

Bar represents 50 μm

Supplementary S29

Sublingual grand

HE

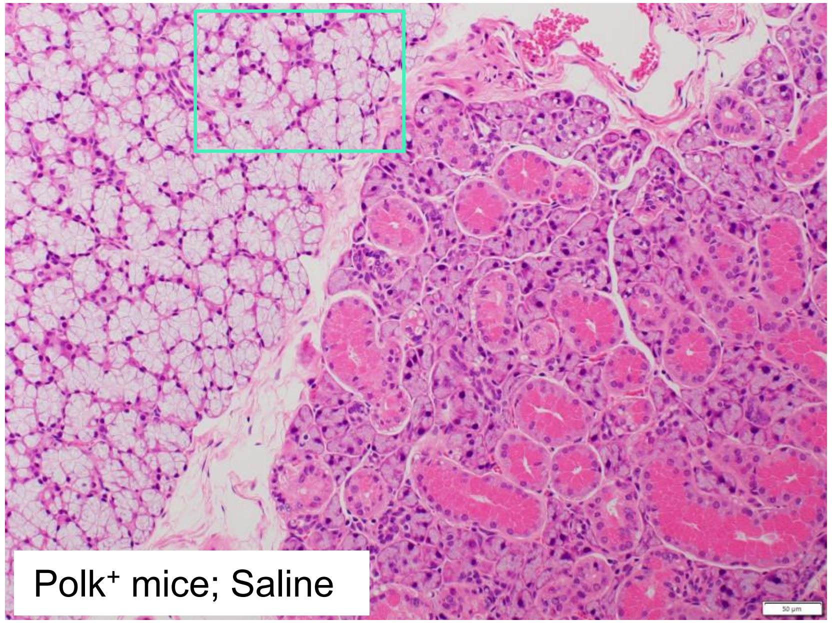

γH2AX

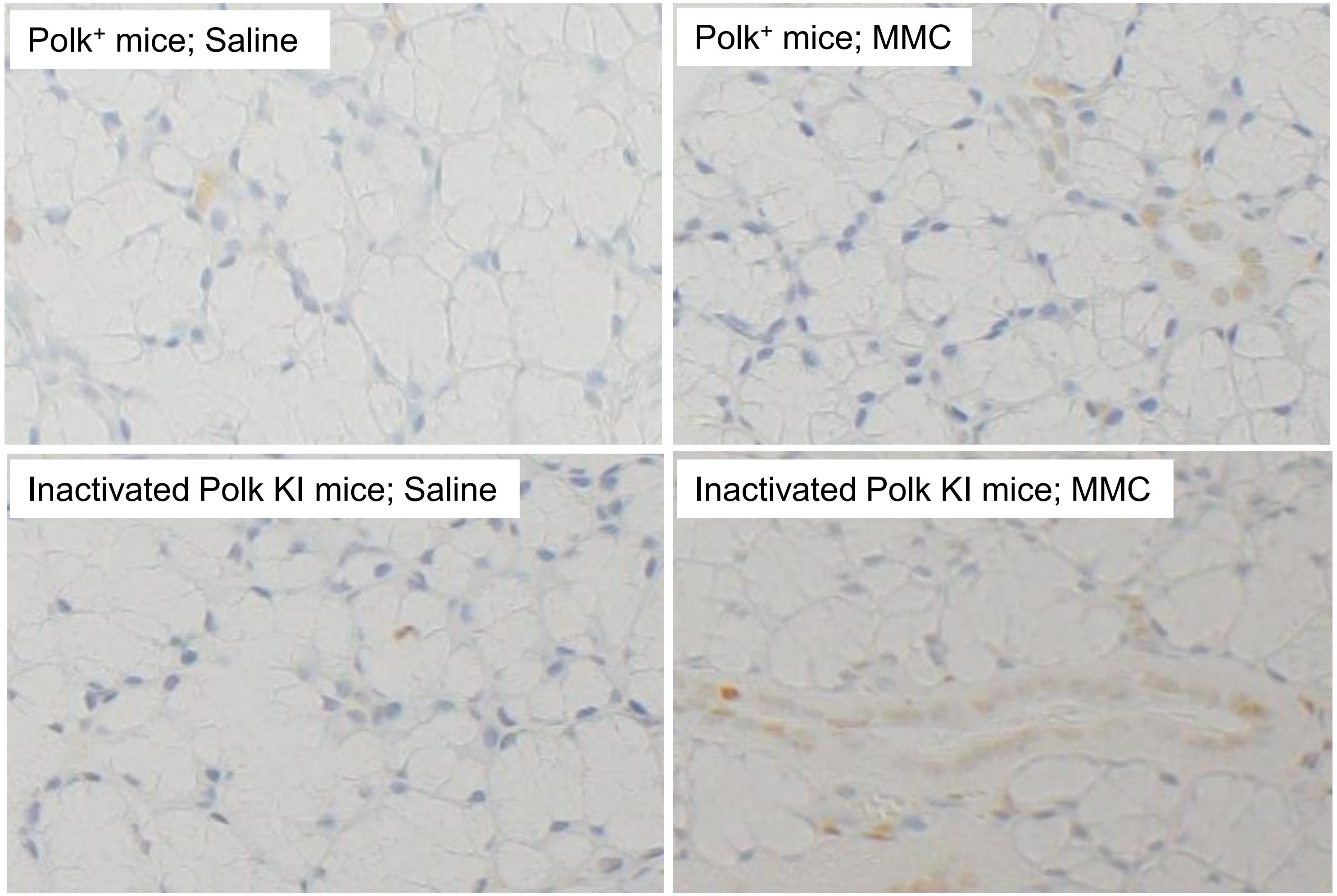

Mice;
Inactivated Polk KI mice, Polk$^+$ mice

Treatment;
Saline: Saline x 5 days
MMC: Mitomycin C 1 mg/kg x 5 days

Staining;
HE; hematoxylin-eosin
γH2AX; γH2AX immunohistochemical stain

Bar represents 50 μm

Supplementary S30

Retina

HE

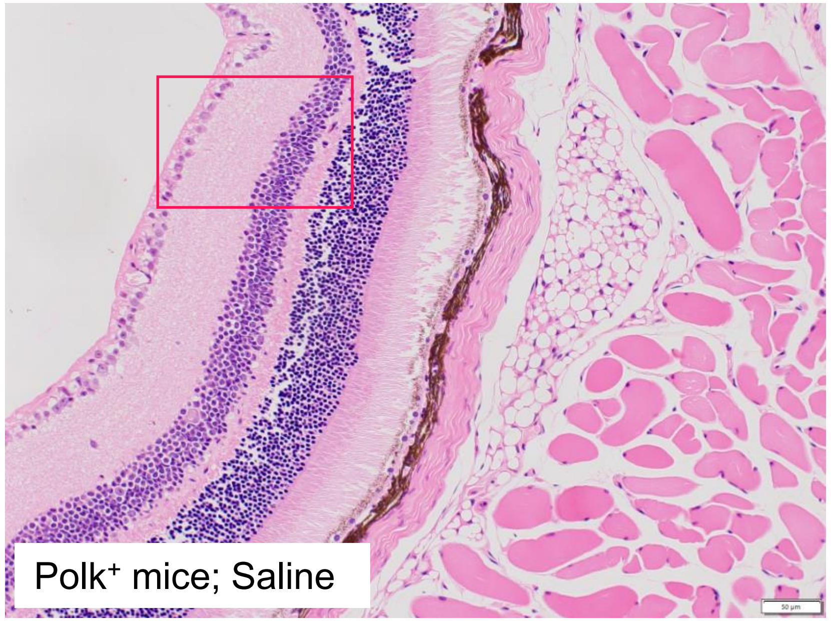

γH2AX

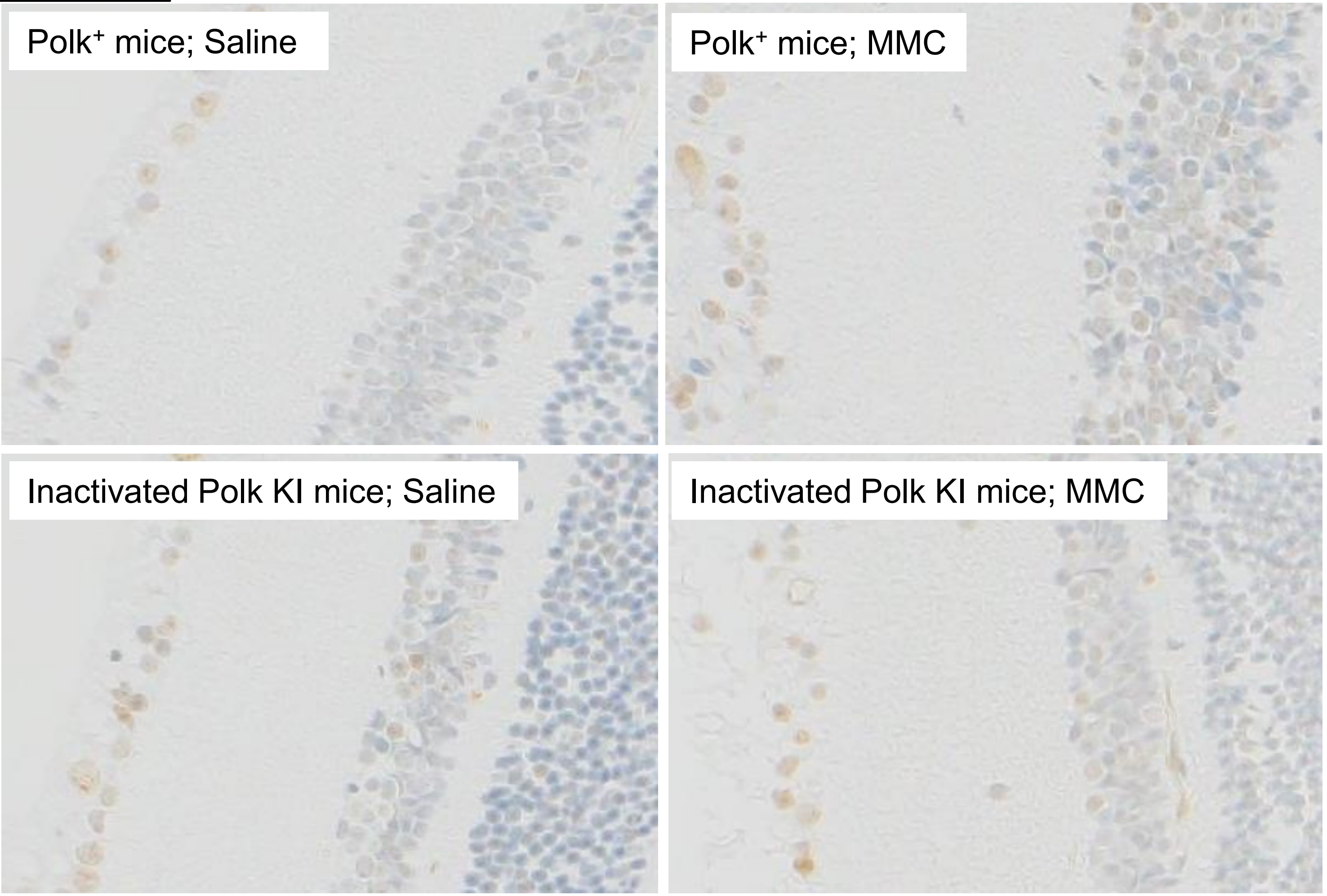

Mice;
 Inactivated Polk KI mice, Polk^{+} mice

Treatment;
 Saline: Saline x 5 days
 MMC: Mitomycin C 1 mg/kg x 5 days

Staining;
 HE; hematoxylin-eosin
 γH2AX; γH2AX immunohistochemical stain

Bar represents 50 μm

Supplementary S31

Hippocampus

HE

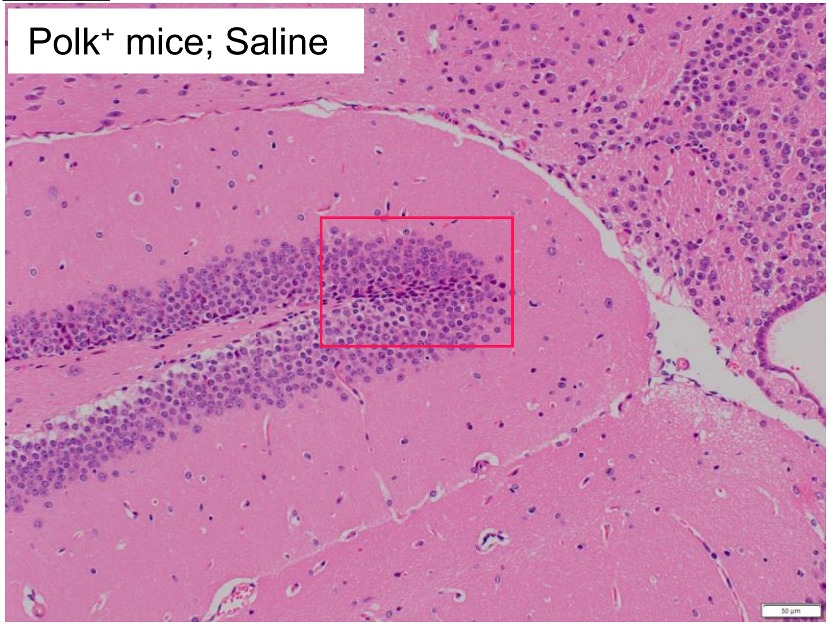

γH2AX

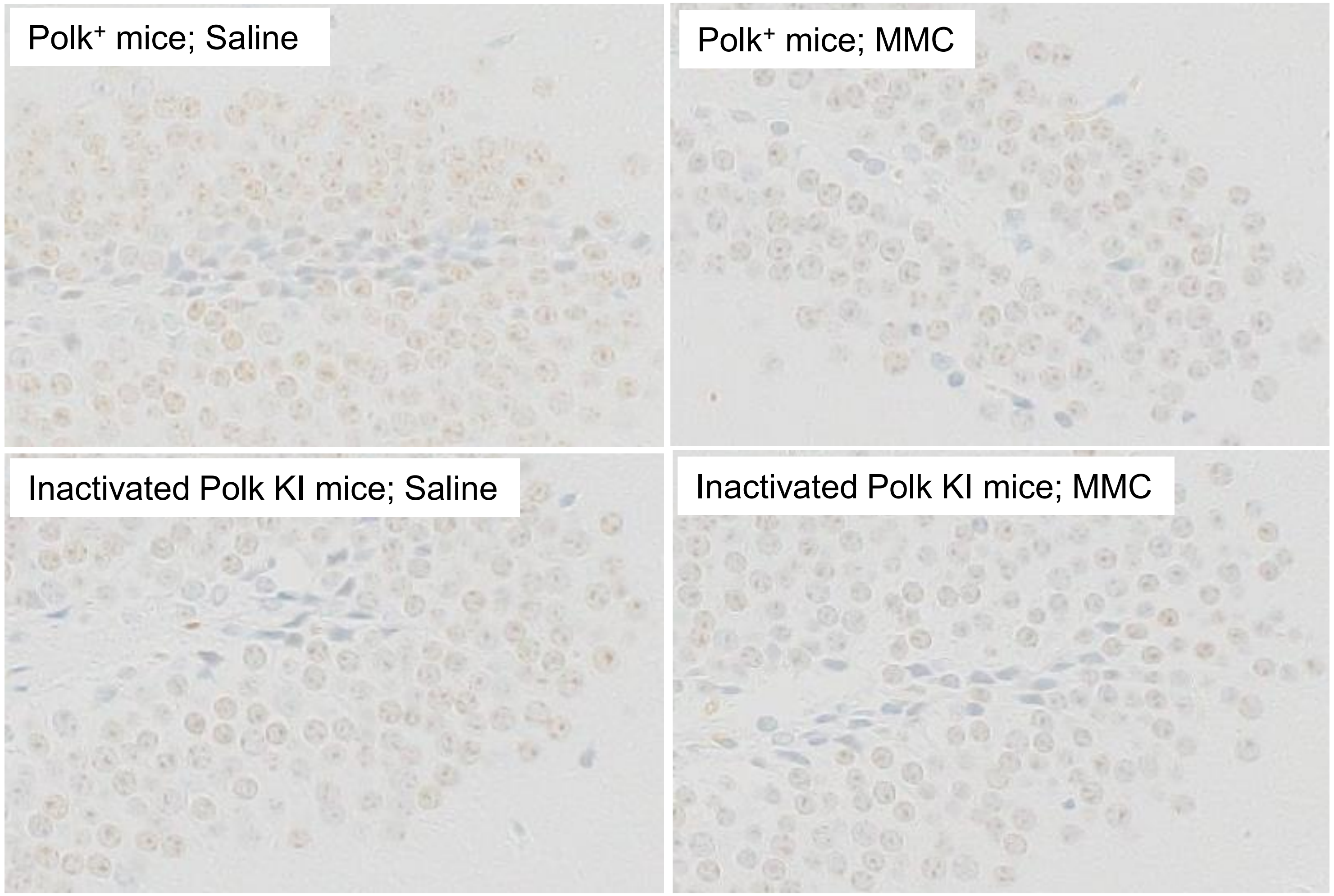

Mice;
  Inactivated Polk KI mice, Polk$^+$ mice

Treatment;
  Saline: Saline x 5 days
  MMC: Mitomycin C 1 mg/kg x 5 days

Staining;
  HE; hematoxylin-eosin
  γH2AX; γH2AX immunohistochemical stain

Bar represents 50 μm

Supplementary S32

Cerebral cortex

HE

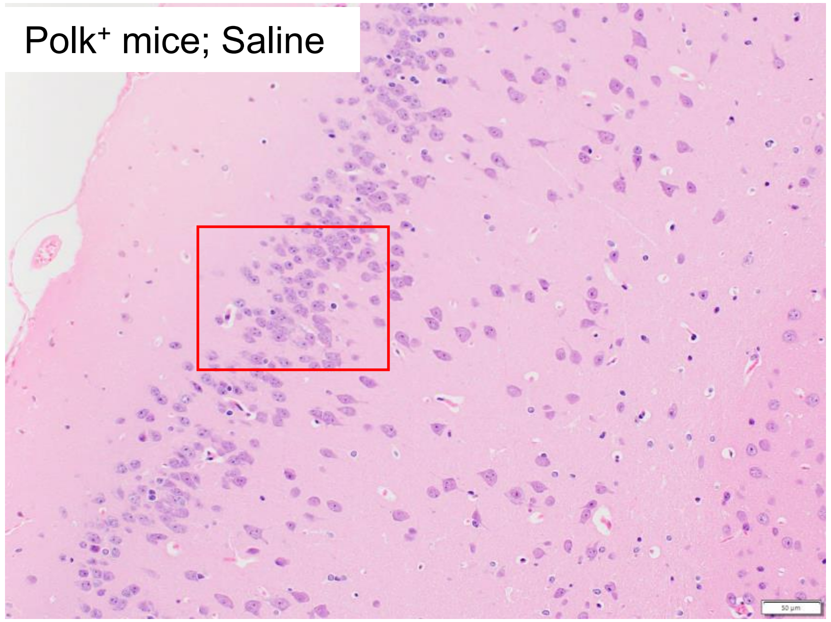

Mice;
Inactivated Polk KI mice, Polk⁺ mice

Treatment;
Saline: Saline x 5 days
MMC: Mitomycin C 1 mg/kg x 5 days

Staining;
HE; hematoxylin-eosin
γH2AX; γH2AX immunohistochemical stain

Bar represents 50 μm

γH2AX

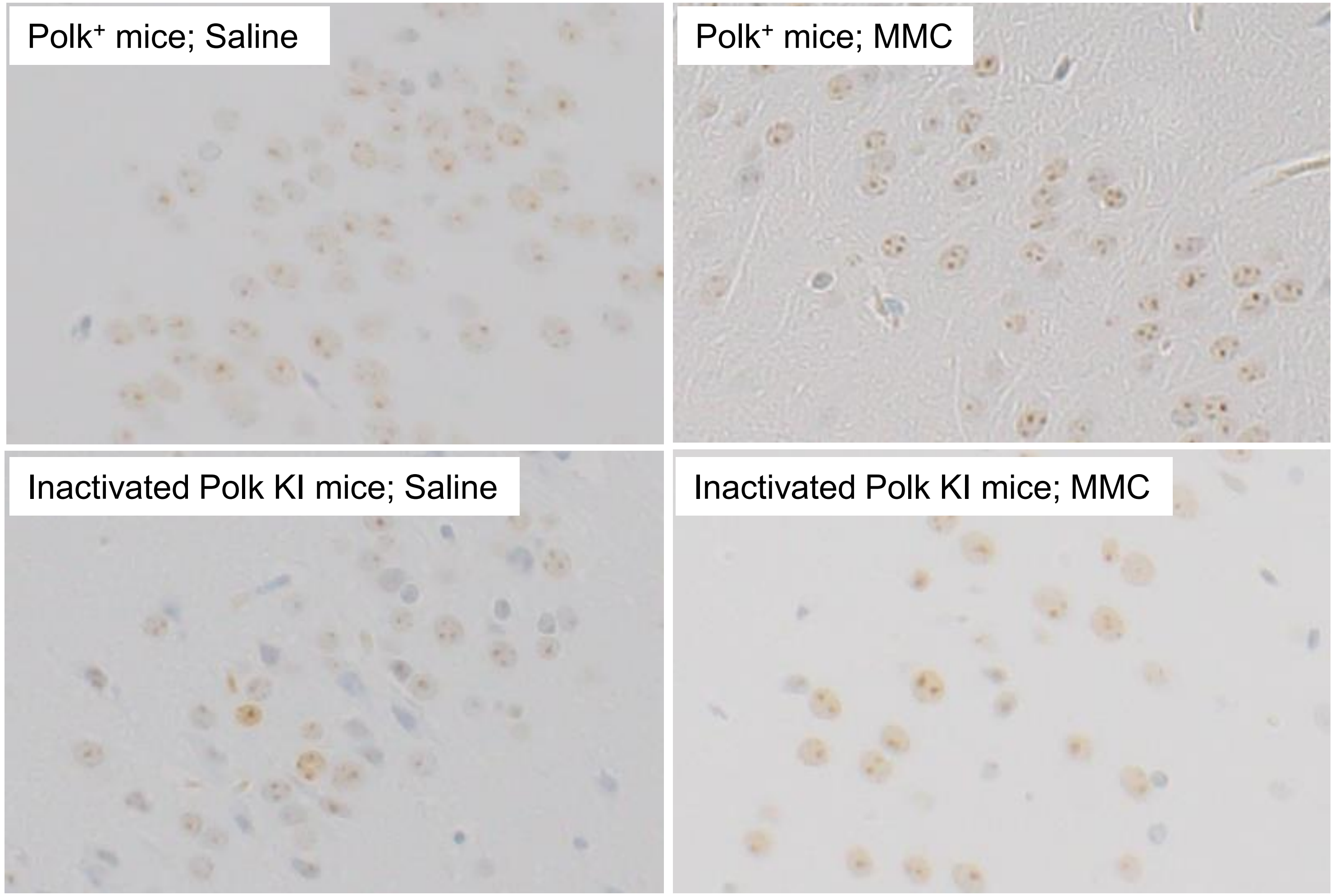

Supplementary S33

Cerebral white matter

HE

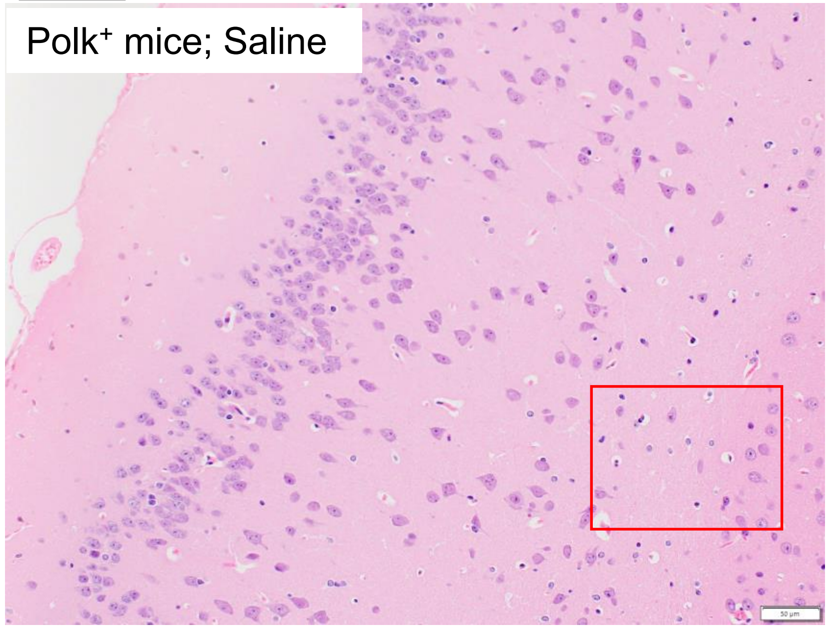

γH2AX

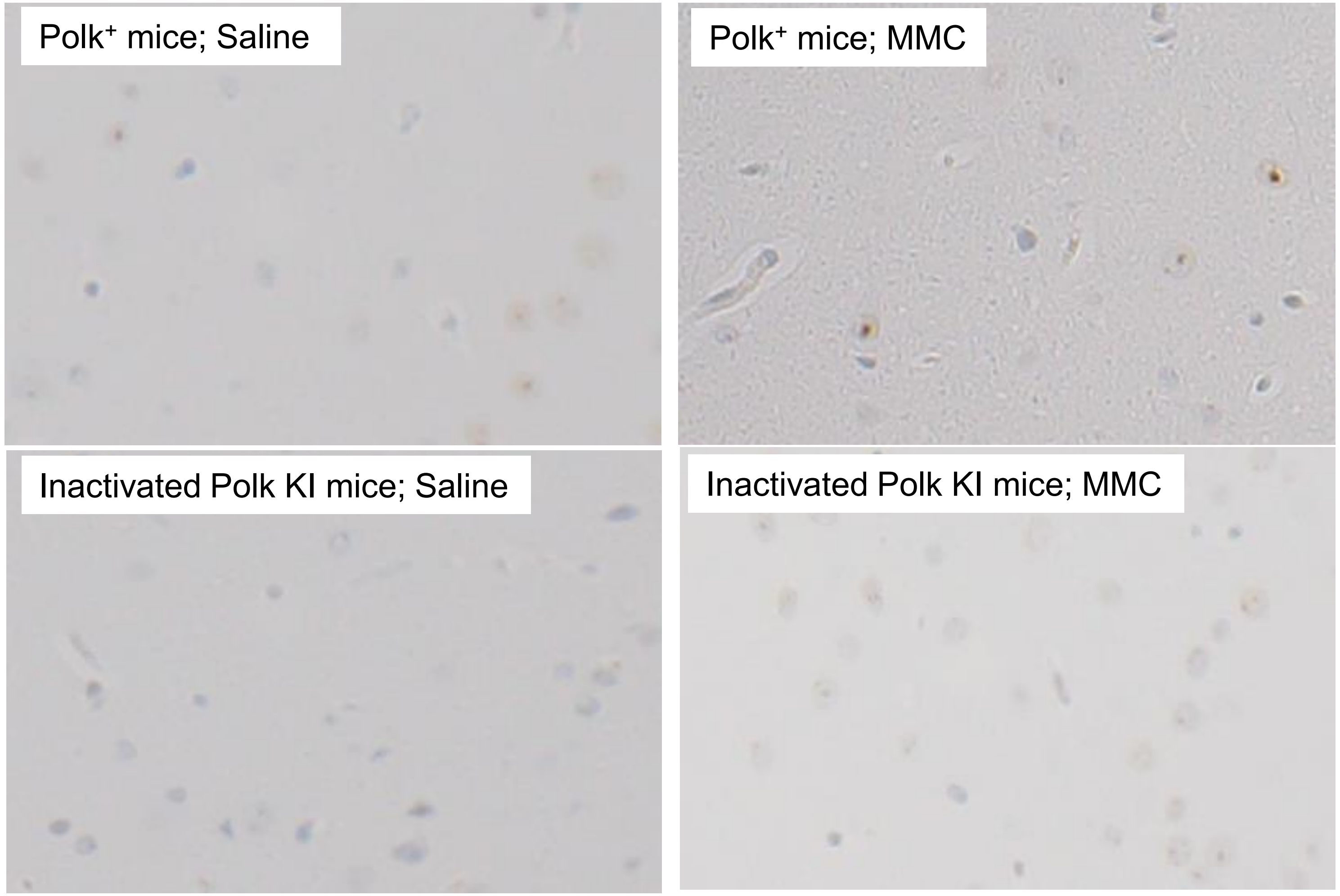

Mice;
Inactivated Polk KI mice, Polk+ mice

Treatment;
Saline: Saline x 5 days
MMC: Mitomycin C 1 mg/kg x 5 days

Staining;
HE; hematoxylin-eosin
γH2AX; γH2AX immunohistochemical stain

Bar represents 50 μm

Supplementary S34

Group	Polk expression	$Polk^+$ mice, Saline						$Polk^+$ mice, MMC						Inactivated Polk KI mice, Saline						Inactivated Polk KI mice, MMC					
Treatmnet	RNA seq reference data (RPKM)	Saline						MMC						Saline						MMC					
Dose		0 mg/kg x 5 day						1 mg/kg x 5 day						0 mg/kg x 5 day						1 mg/kg x 5 day					
Sex		Male			Female			Male			Female			Male			Female			Male			Female		
Animal No.		1101	1102	1103	5101	5102	5103	1201	1202	1203	5201	5202	5203	1301	1302	1303	5301	5302	5303	1401	1402	1403	5401	5402	5403
1 Liver	0.212	0	0	0	0	0	1	2	2	2	2	2	2	0	0	0	1	2	0	4	3	4	3	3	3
2 Bladder	3.850	0	0	1	1	1	1	2	2	2	2	3	3	1	0	1	2	2	0	3	3	4	4	3	4
3 Adrenal cortex	0.459	0	0	0	0	0	1	1	4	1	2	2	2	1	0	1	3	3	3	3	3	3	3	3	4
4 Thyroid		1	1	2	0	missing	0	2	3	3	1	2	1	1	0	1	1	0	1	3	4	3	3	3	2
5 Testis (round spermatids)	3.604	0	0	0	NA	NA	NA	3	3	3	NA	NA	NA	0	0	0	NA	NA	NA	4	4	4	NA	NA	NA
6 Tongue basal epithelial		0	2	0	0	1	1	2	2	3	3	3	2	1	0	1	1	1	1	4	3	3	4	4	3
7 Glandular stomach	0.124	1	1	1	0	0	1	2	2	2	2	2	2	1	1	1	1	1	1	3	3	3	3	3	3
8 Forestomach	0.124	0	0	0	0	0	0	2	1	2	1	1	2	0	1	0	0	0	1	2	2	1	3	3	3
9 Submandibular gland		1	1	1	1	1	1	3	3	3	2	3	3	1	1	1	1	1	1	4	3	4	4	3	4
10 Esophagus		1	1	1	1	0	1	3	2	4	2	3	3	2	1	2	1	1	1	3	4	4	4	4	3
11 Mammary gland	0.609	missing	missing	missing	1	1	1	3	missing	4	3	3	3	missing	missing	missing	1	1	missing	4	4	missing	4	4	4
12 Subcutaneous vascular endothelium		0	0	0	0	0	0	4	1	1	3	2	2	1	0	0	0	0	0	3	3	1	4	3	3
13 Seminal vesicle		0	0	0	NA	NA	NA	0	2	1	NA	NA	NA	0	0	0	NA	NA	NA	1	3	1	NA	NA	NA
14 Spleen	0.301	1	2	2	2	3	2	2	1	2	2	1	2	2	2	2	2	3	2	2	2	2	3	2	3
15 Ileum	0.077	1	1	1	1	1	1	1	2	2	2	3	2	missing	1	2	1	1	1	1	2	3	3	3	4
16 Epididymis		1	2	1	NA	NA	NA	3	2	1	NA	NA	NA	1	2	1	NA	NA	NA	2	4	2	NA	NA	NA
17 Lung	0.825	0	0	0	0	0	0	1	1	2	2	2	1	0	0	0	0	2	0	2	1	2	3	2	3
18 Kidney	0.520	0	0	0	0	0	0	1	1	1	1	0	0	0	0	0	0	0	1	1	1	1	2	1	1
19 Hair follicle		1	0	0	0	0	1	1	1	2	1	1	2	1	1	0	1	0	0	2	2	1	2	2	2
20 Cornea		1	2	2	1	2	1	4	2	3	2	2	0	2	3	2	1	0	4	4	2	2	2	3	3
21 Epidermis		0	0	0	1	1	1	2	1	2	2	1	2	1	1	1	1	1	1	2	1	1	3	3	3
22 Colon	0.384	2	2	2	1	2	1	3	3	3	3	3	3	2	2	1	1	1	2	3	2	3	4	4	4
23 Tongue skeletal muscle		0	0	0	0	1	1	2	2	2	2	2	2	1	0	0	1	0	0	2	2	2	3	3	2
24 Heart	0.357	0	0	0	0	0	0	0	1	1	0	0	0	0	0	0	0	0	2	2	1	1	0	0	0
25 Trachea epithelium		2	2	2	1	1	2	3	4	3	4	3	3	missing	2	3	1	missing	1	4	4	3	3	4	4
26 Harderian gland		1	1	2	2	2	2	3	3	3	2	3	2	2	1	3	1	1	3	3	3	2	4	1	4
27 Pancreas		1	2	2	2	1	1	2	1	2	3	2	2	1	1	1	1	1	2	2	2	3	2	2	2
28 Brainstem		2	2	3	1	2	3	3	3	2	2	2	2	2	1	2	3	2	2	3	3	3	2	2	2
29 Testis (whole cells)	3.604	4	4	4	NA	NA	NA	4	4	4	NA	NA	NA	4	4	4	NA	NA	NA	4	4	4	NA	NA	NA
30 Mesenteric lymph node		1	1	1	1	1	1	2	3	2	2	2	2	1	1	1	1	1	1	2	2	2	2	2	3
31 Prostate		0	1	1	NA	NA	NA	3	2	2	NA	NA	NA	2	1	1	NA	NA	NA	3	2	2	NA	NA	NA
32 Cerebellum	1.710	1	1	1	0	1	1	2	2	2	1	2	2	1	1	1	0	0	1	2	2	2	2	1	2
33 Thymus	0.693	2	2	2	2	2	2	2	3	3	3	2	3	2	2	1	3	3	3	2	2	3	2	3	3
34 Sublingual gland		0	0	0	0	0	0	2	1	2	1	1	1	0	0	0	0	0	0	2	0	1	2	1	1
35 Retina		3	2	3	2	3	3	4	4	4	4	4	4	4	4	4	3	3	3	4	3	3	4	4	4
36 Hippocampus		4	4	4	2	2	4	4	missing	missing	4	missing	4	missing	4	4	3	3	missing	4	2	missing	4	4	missing
37 Cerebral cortex	1.443	3	3	3	2	2	2	4	3	4	3	4	3	3	3	3	2	3	3	4	3	2	4	2	3
38 Cerebral white matter		2	2	2	0	0	1	3	2	2	3	2	2	2	2	1	2	1	1	2	2	0	2	1	2

Polk expression data ;
https://www.ncbi.nlm.nih.gov/gene/27015/?report=expression
