# Supplementary material for: Mitomycin C-induced DNA double-strand breaks are enhanced by catalytical inactivation of DNA polymerase κ in mice
Source: Genes Environ. 2025 Nov 6;47:22. doi: 10.1186/s41021-025-00343-x (PMC12590760; doi:10.1186/s41021-025-00343-x)
Supplement: Supplementary file 2 — Supplementary Material 2 [file 41021_2025_343_MOESM2_ESM.pdf]

Supplementary S1

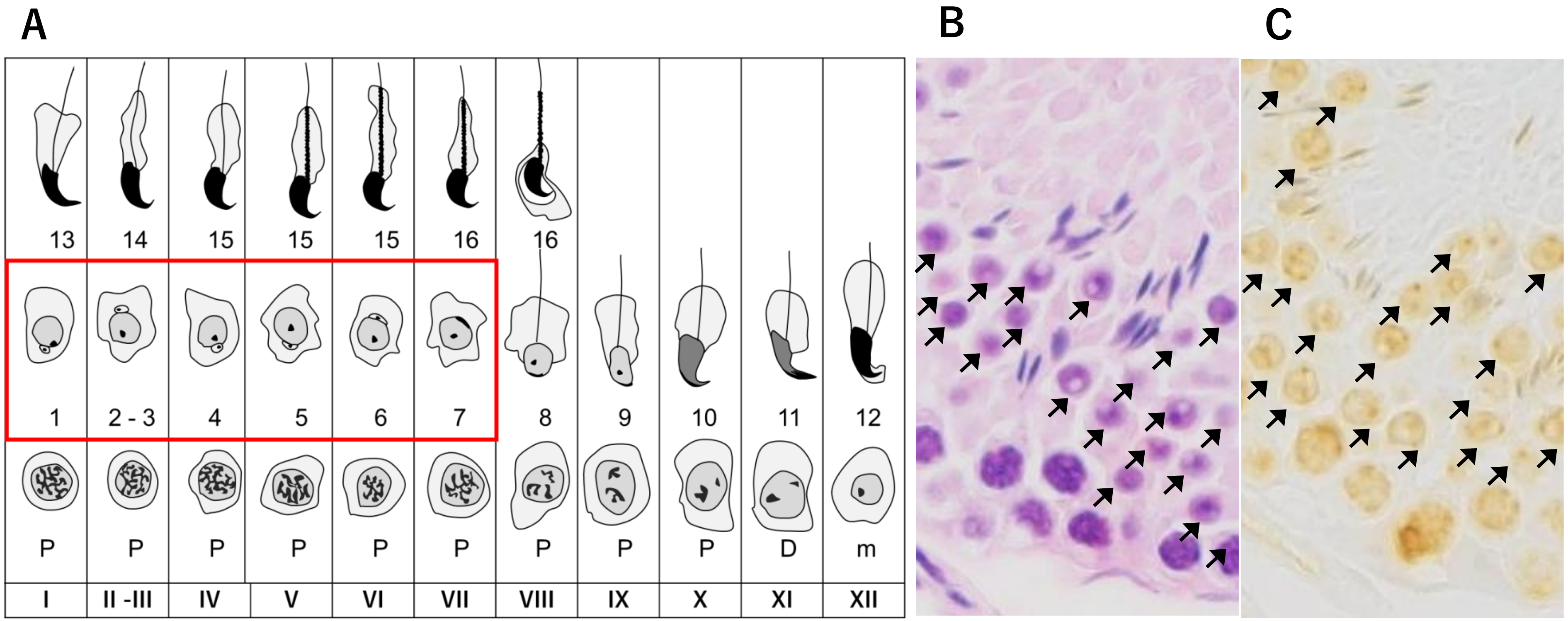

(A) Mouse spermatogenic cycle is illustrated. 1-16, step 1-16 spermatids; P, pachytene spermatocytes; D, diplotene spermatocytes; m, meiotic division. Among 12 stages (I to XII), round spermatids in stage I to VII (red box, step 1 to 7) were evaluated for γH2AX foci staining in this study. (B) Hematoxylin-eosin staining. (C) Immunohistochemical staining with anti-γH2AX antibody. Arrows represent step 1 to7 round spermatids.

Supplementary S2

Tongue basal epithelial

HE

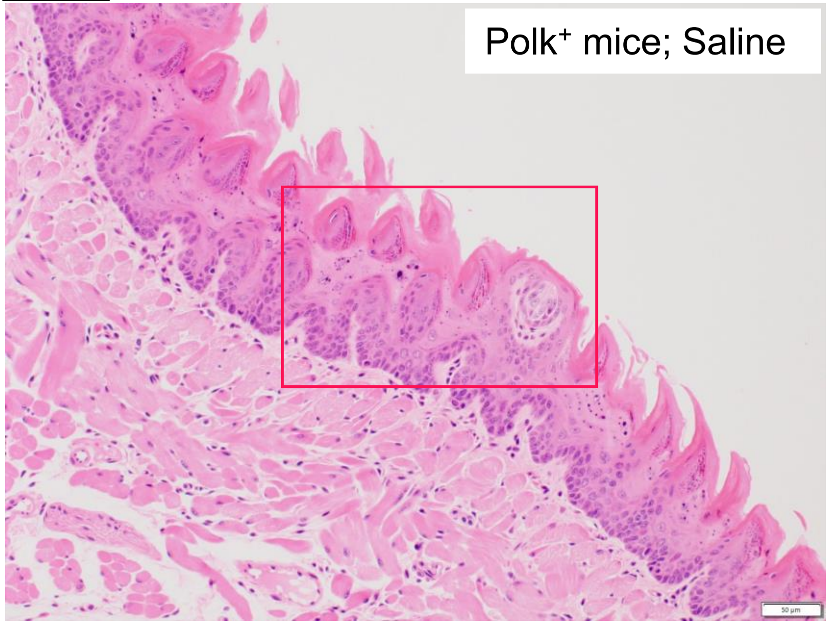

γH2AX

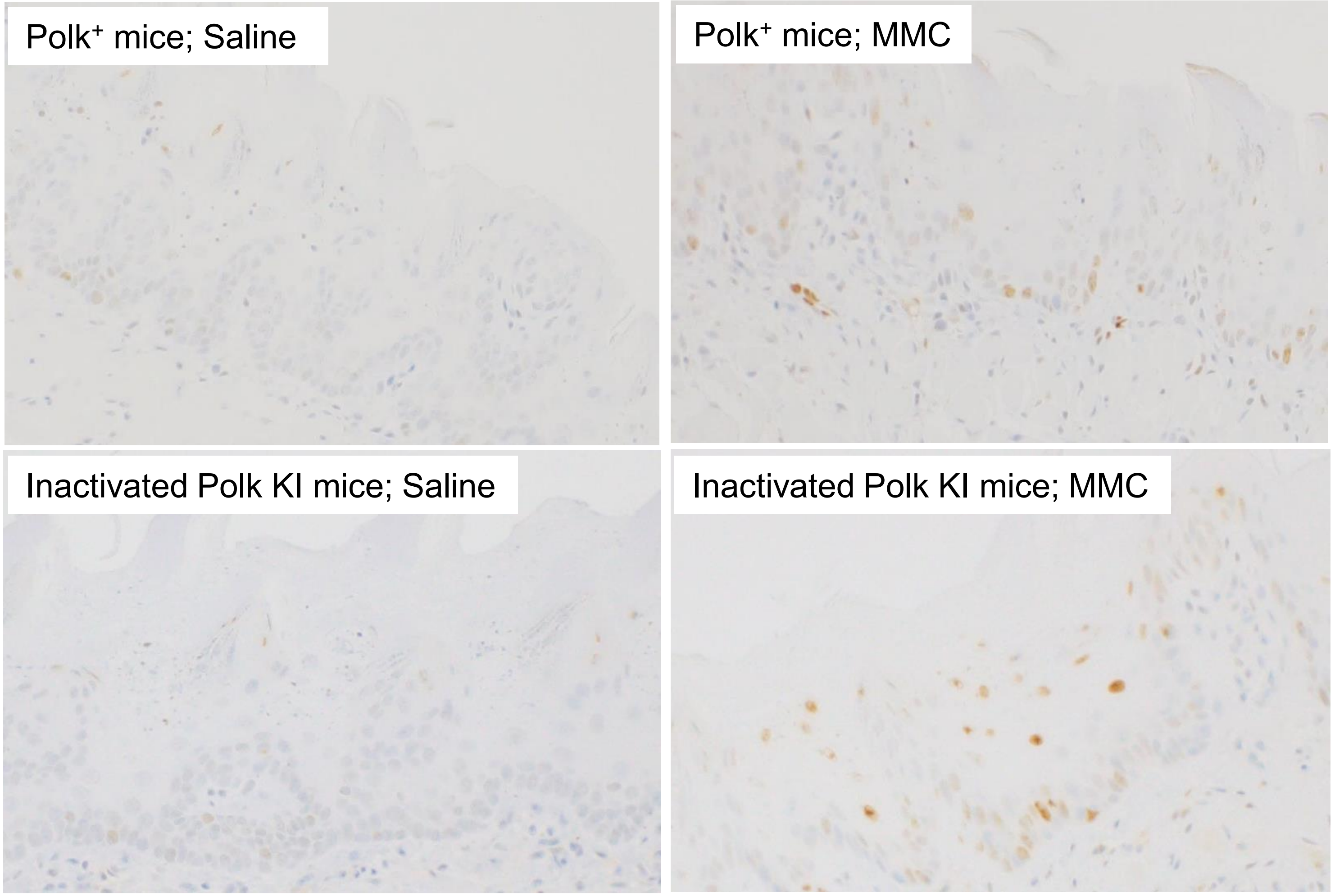

Mice;
  Inactivated Polk KI mice, Polk$^+$ mice

Treatment;
  Saline: Saline x 5 days
  MMC: Mitomycin C 1 mg/kg x 5 days

Staining;
  HE; hematoxylin-eosin
  γH2AX; γH2AX immunohistochemical stain

Bar represents 50 μm

Supplementary S3

Glandular stomach

HE

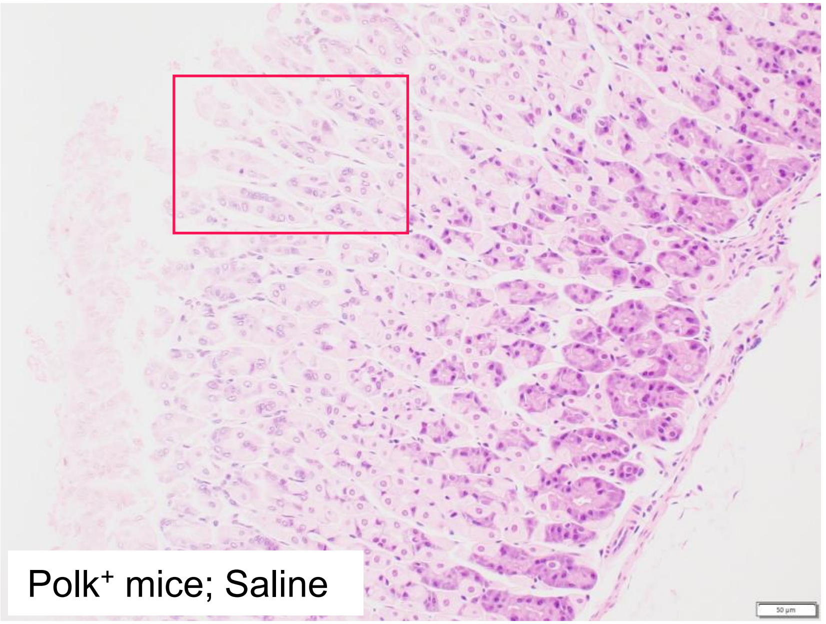

γH2AX

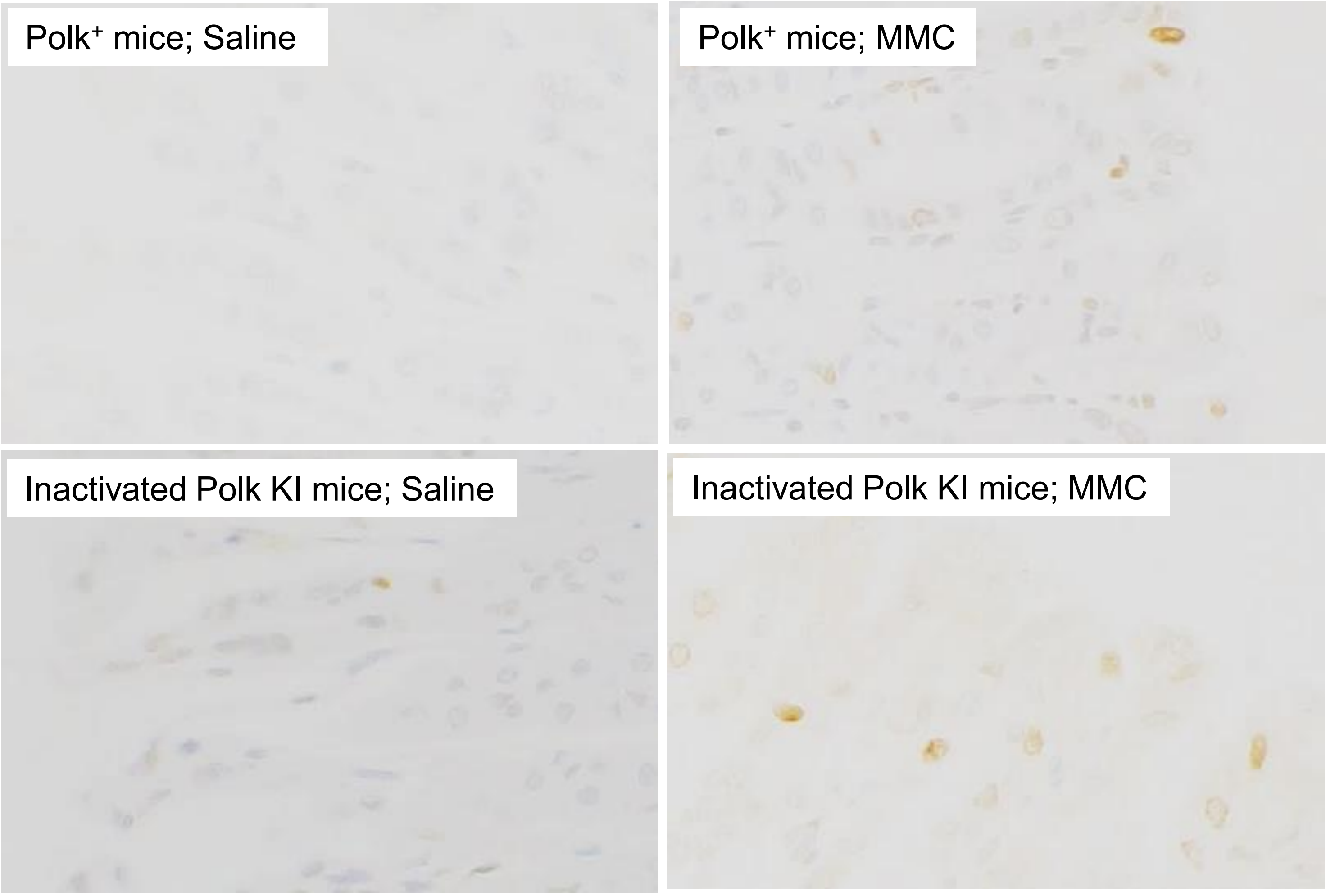

Mice;
Inactivated Polk KI mice, Polk⁺ mice

Treatment;
Saline: Saline x 5 days
MMC: Mitomycin C 1 mg/kg x 5 days

Staining;
HE; hematoxylin-eosin
γH2AX; γH2AX immunohistochemical stain

Bar represents 50 μm

Supplementary S4

Forestomach

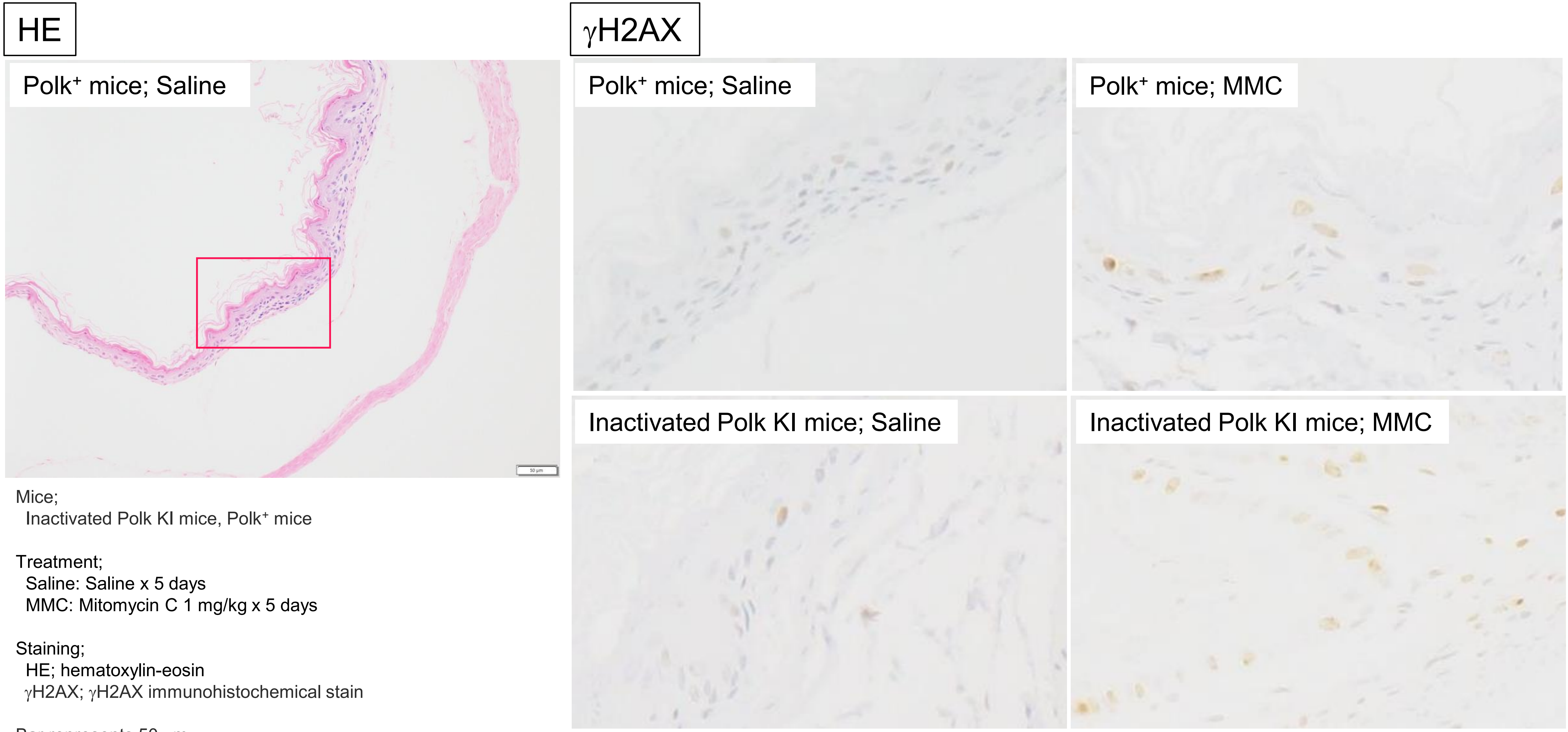

Mice;
Inactivated Polk KI mice, Polk^{+} mice

Treatment;
Saline: Saline x 5 days
MMC: Mitomycin C 1 mg/kg x 5 days

Staining;
HE; hematoxylin-eosin
γH2AX; γH2AX immunohistochemical stain

Bar represents 50 μm

Supplementary S5

Submandibular gland

HE

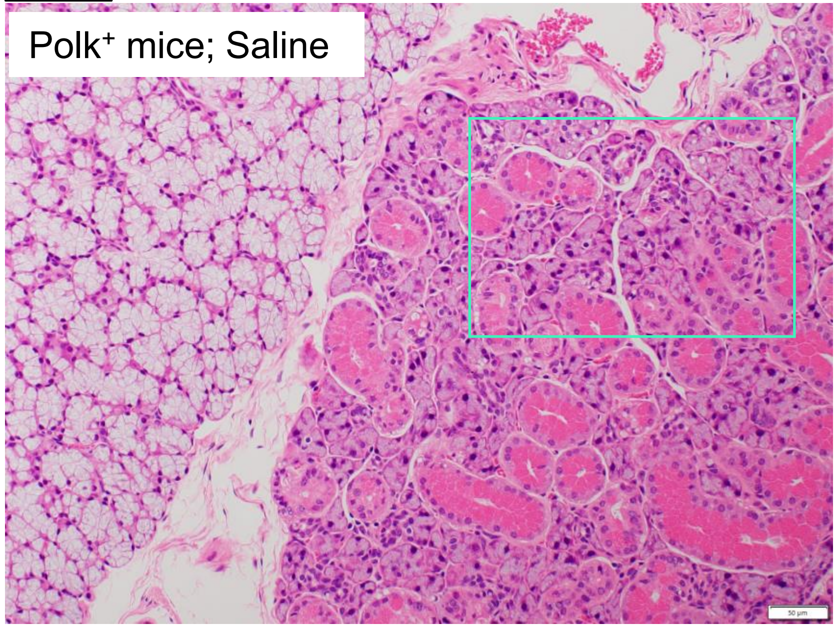

γH2AX

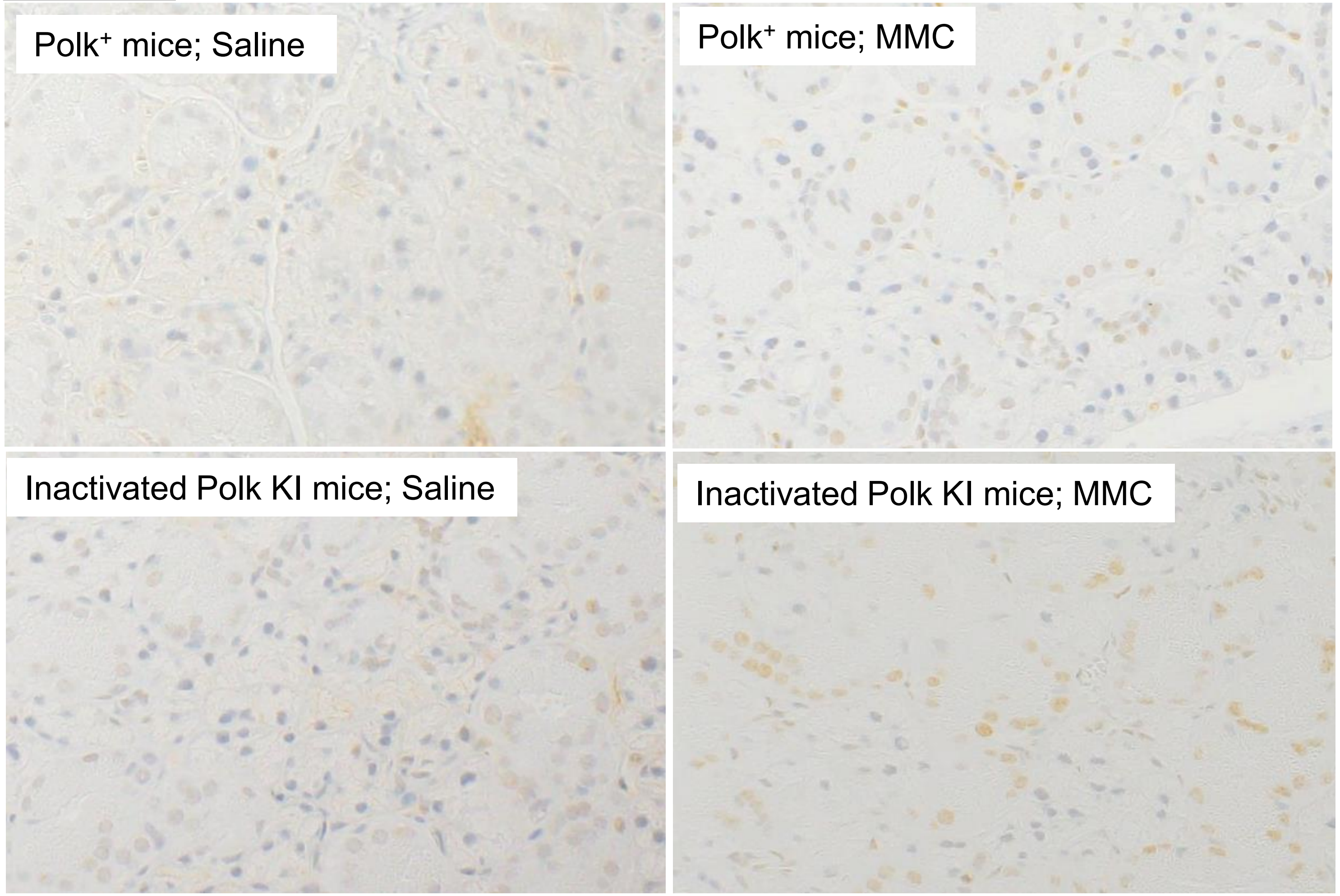

Mice;
Inactivated Polk KI mice, Polk+ mice

Treatment;
Saline: Saline x 5 days
MMC: Mitomycin C 1 mg/kg x 5 days

Staining;
HE; hematoxylin-eosin
γH2AX; γH2AX immunohistochemical stain

Bar represents 50 μm

Supplementary S6

Esophagus

HE

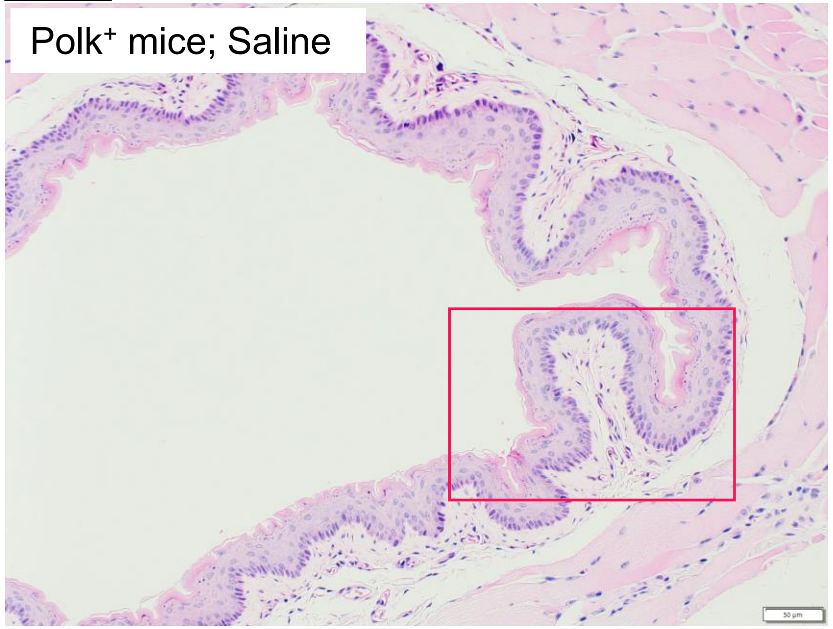

γH2AX

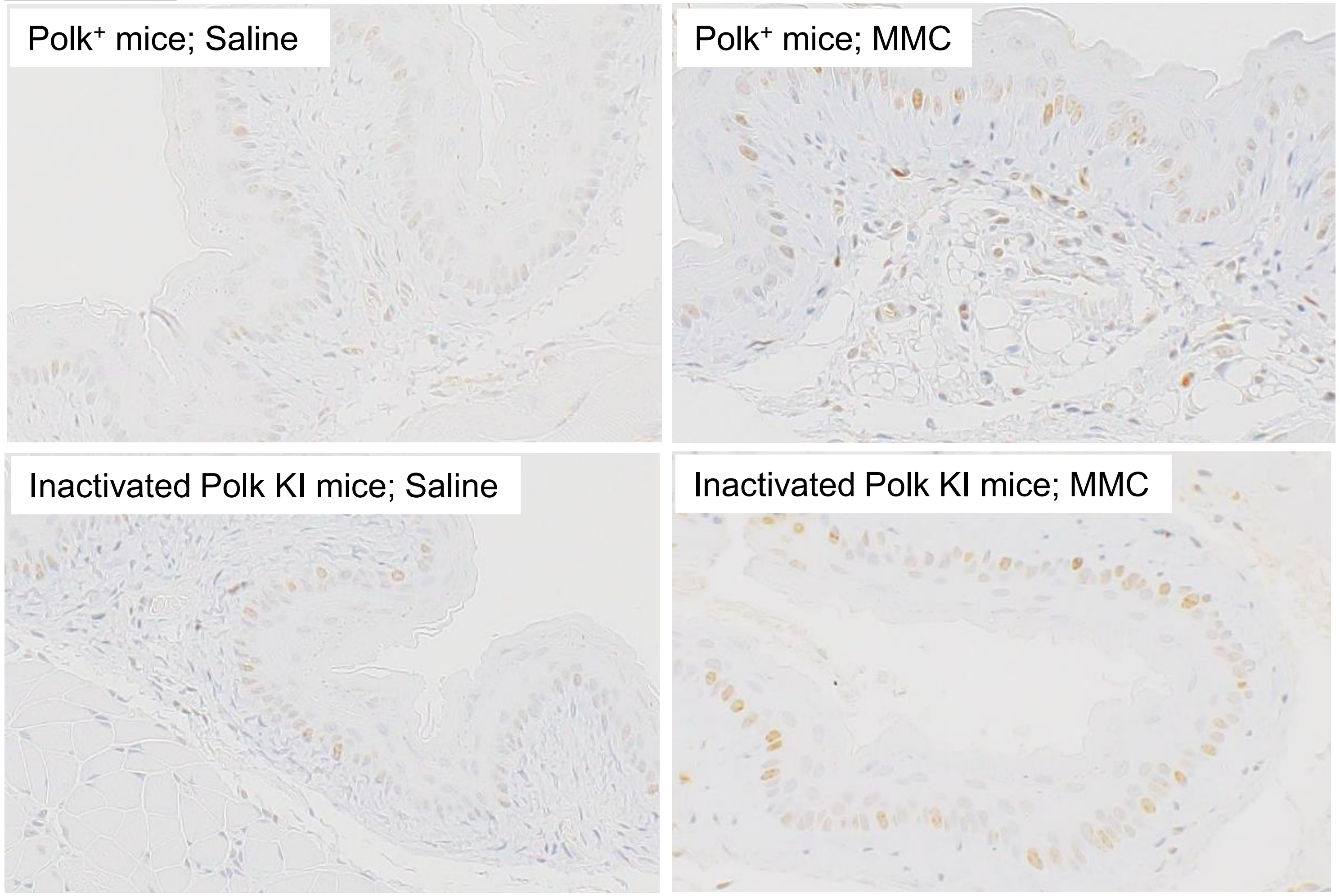

Mice;
  Inactivated Polk KI mice, Polk⁺ mice

Treatment;
  Saline: Saline x 5 days
  MMC: Mitomycin C 1 mg/kg x 5 days

Staining;
  HE; hematoxylin-eosin
  γH2AX; γH2AX immunohistochemical stain

Bar represents 50 μm

Supplementary S7

Mammary gland

HE

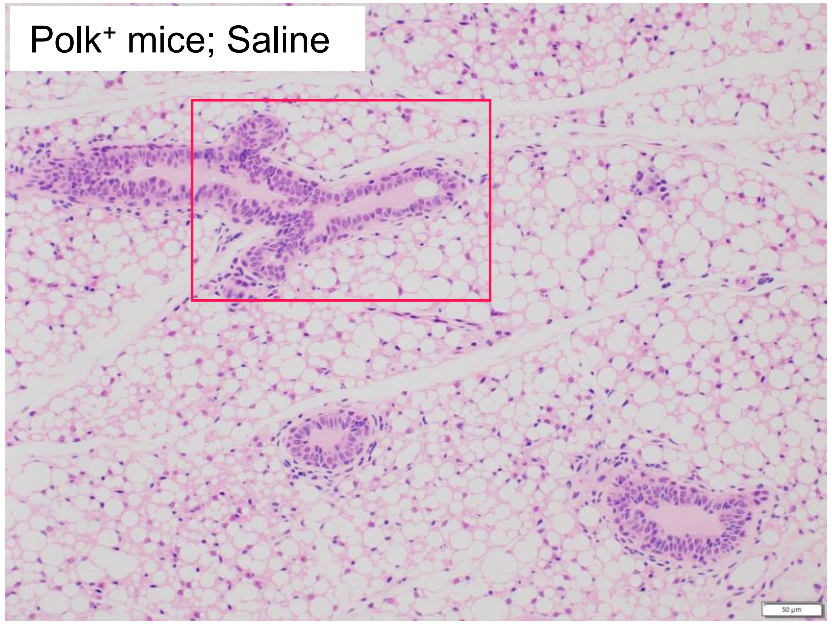

γH2AX

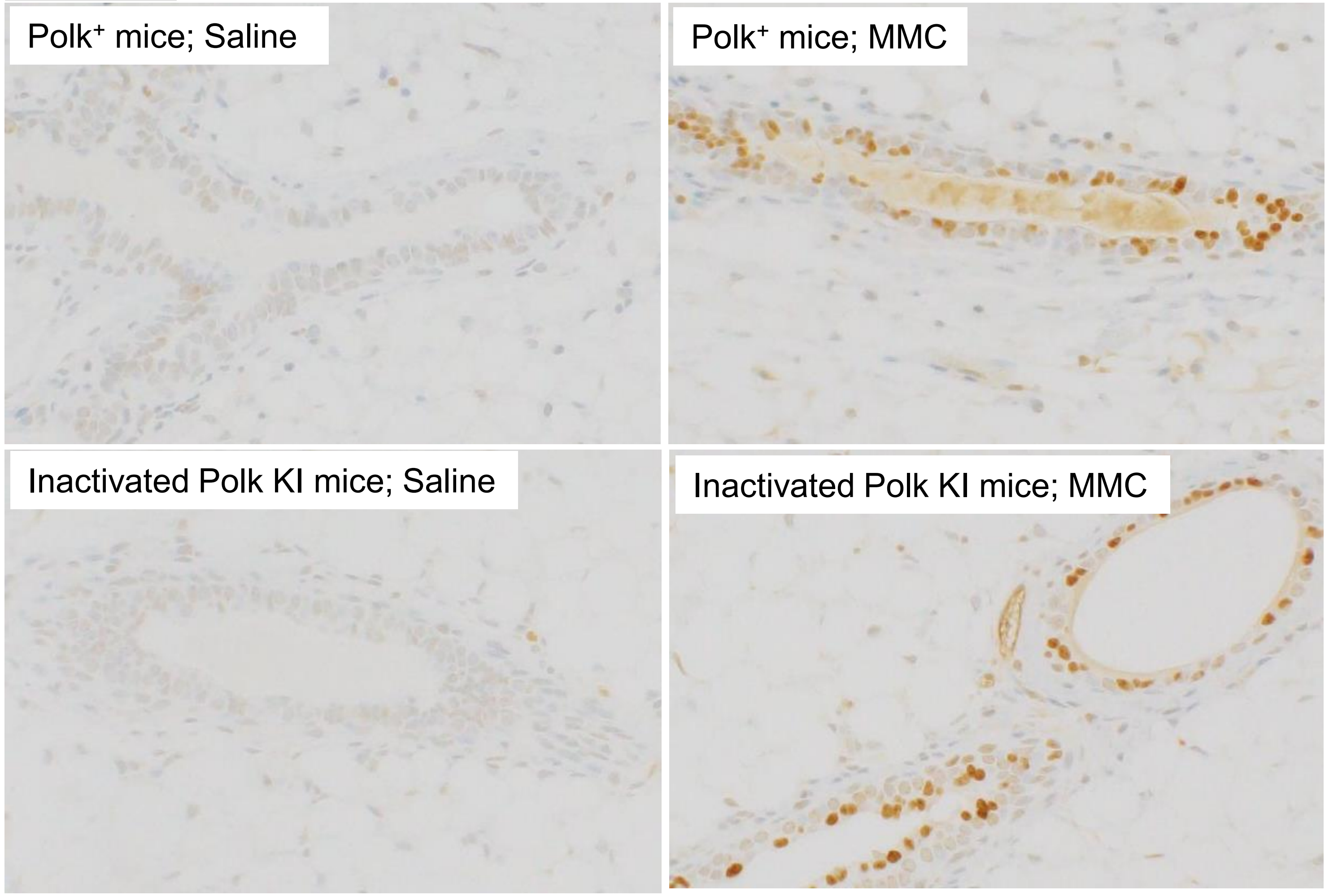

Mice;
  Inactivated Polk KI mice, Polk$^+$ mice

Treatment;
  Saline: Saline x 5 days
  MMC: Mitomycin C 1 mg/kg x 5 days

Staining;
  HE; hematoxylin-eosin
  γH2AX; γH2AX immunohistochemical stain

Bar represents 50 μm

Supplementary S8

Skin vascular endothelium

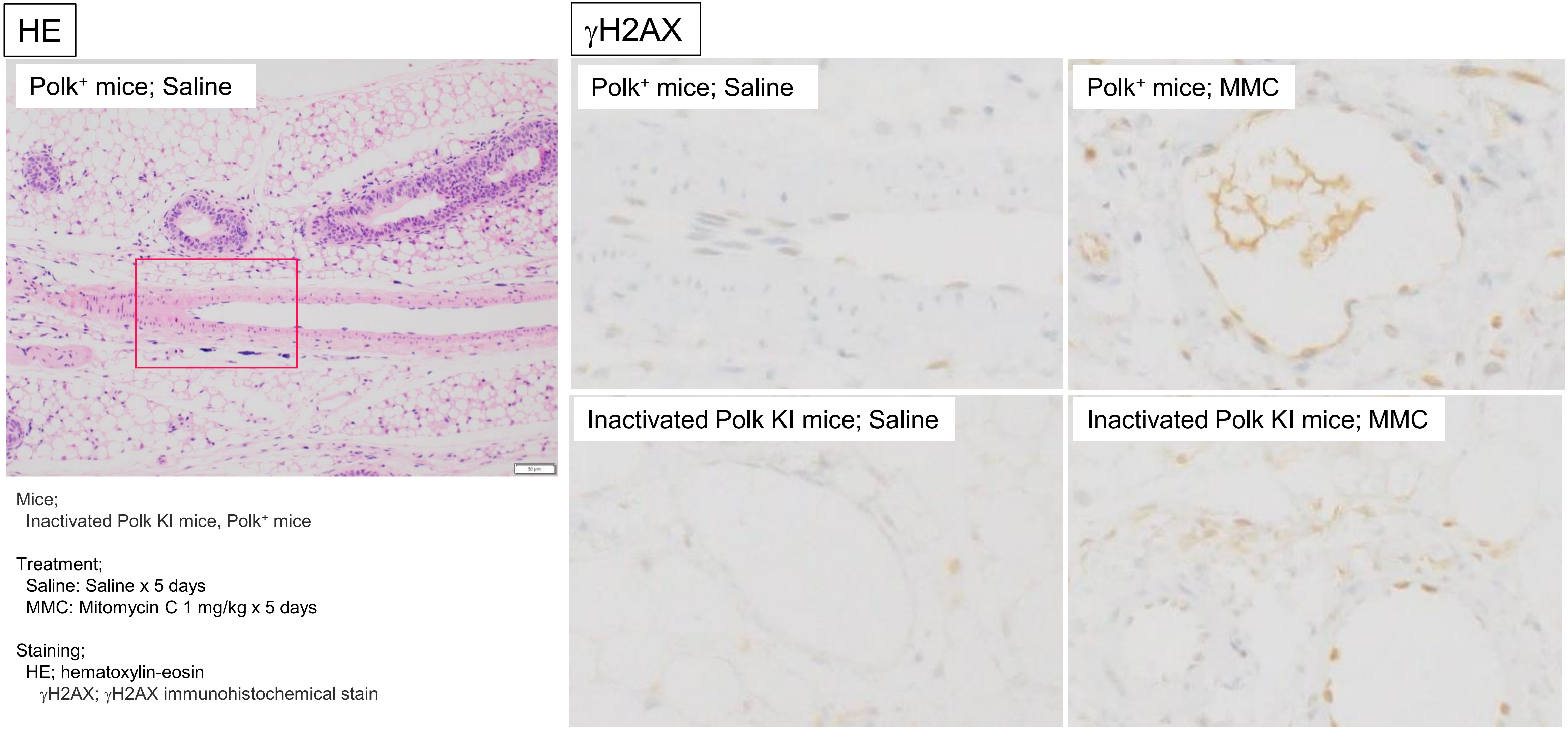

Mice;
Inactivated Polk KI mice, Polk^{+} mice

Treatment;
Saline: Saline x 5 days
MMC: Mitomycin C 1 mg/kg x 5 days

Staining;
HE; hematoxylin-eosin
γH2AX; γH2AX immunohistochemical stain

Bar represents 50 μm

Supplementary S9

Seminal vesicle

HE

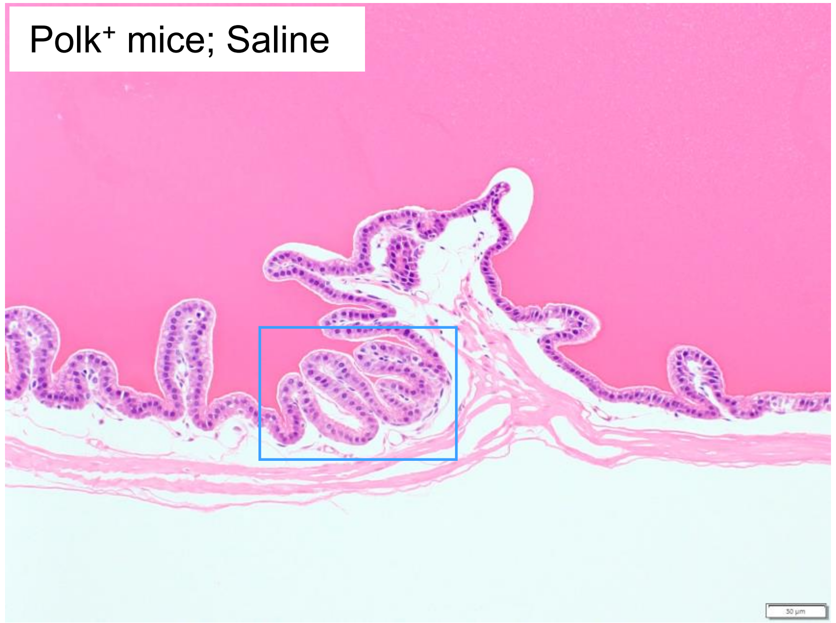

γH2AX

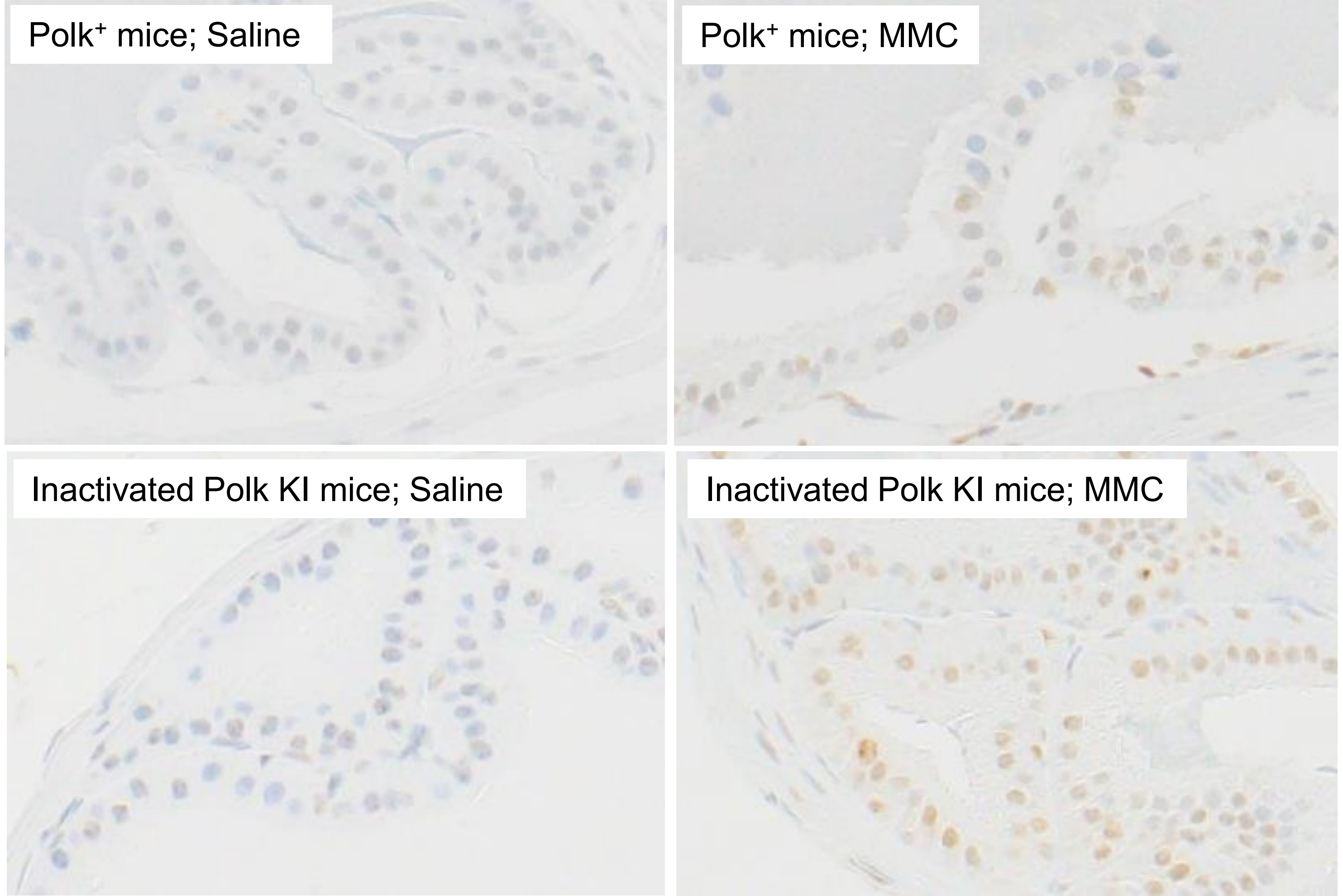

Mice;
Inactivated Polk KI mice, Polk^{+} mice

Treatment;
Saline: Saline x 5 days
MMC: Mitomycin C 1 mg/kg x 5 days

Staining;
HE; hematoxylin-eosin
γH2AX; γH2AX immunohistochemical stain

Bar represents 50 μm

Supplementary S10

Spleen

HE

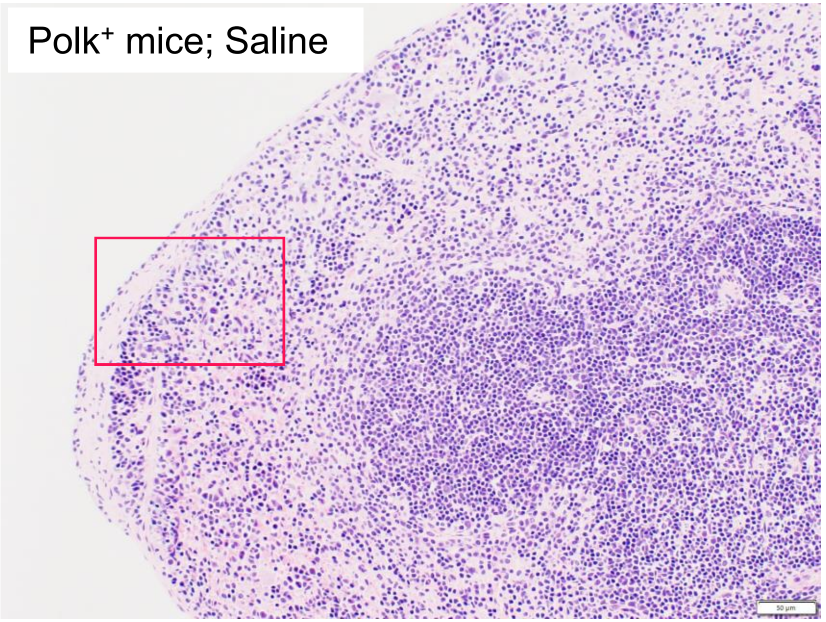

Mice;
Inactivated Polk KI mice, Polk+ mice

Treatment;
Saline: Saline x 5 days
MMC: Mitomycin C 1 mg/kg x 5 days

Staining;
HE; hematoxylin-eosin
γH2AX; γH2AX immunohistochemical stain

Bar represents 50 μm

γH2AX

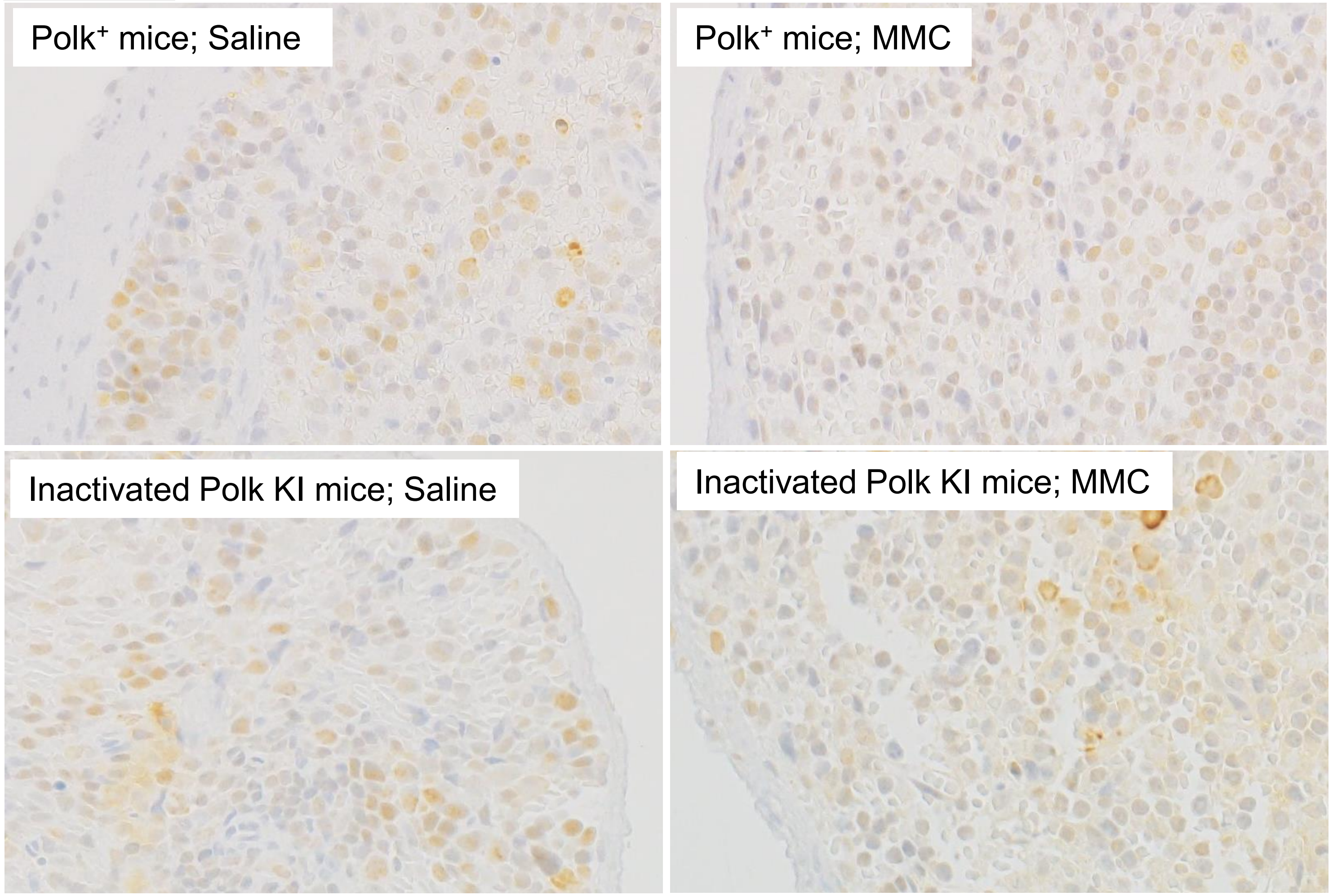

Supplementary S11

Ileum

HE

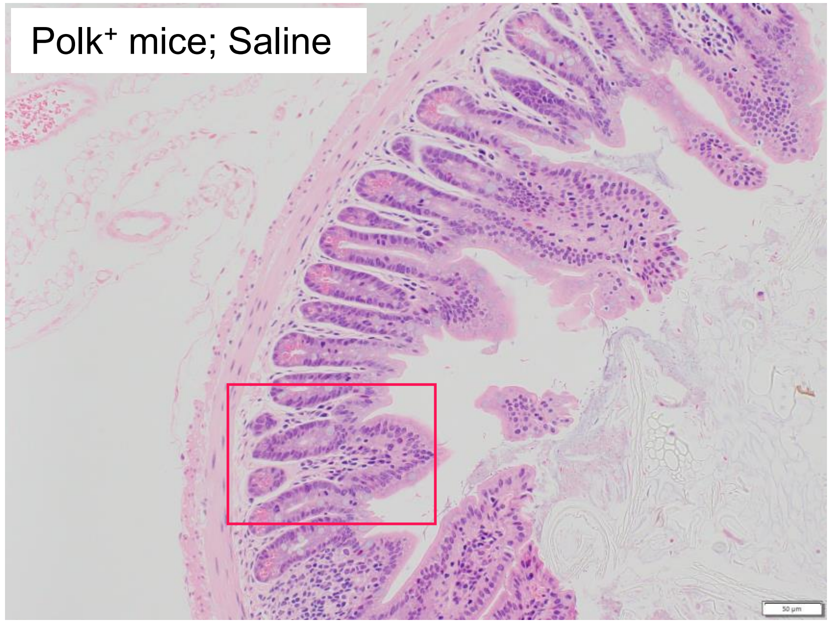

γH2AX

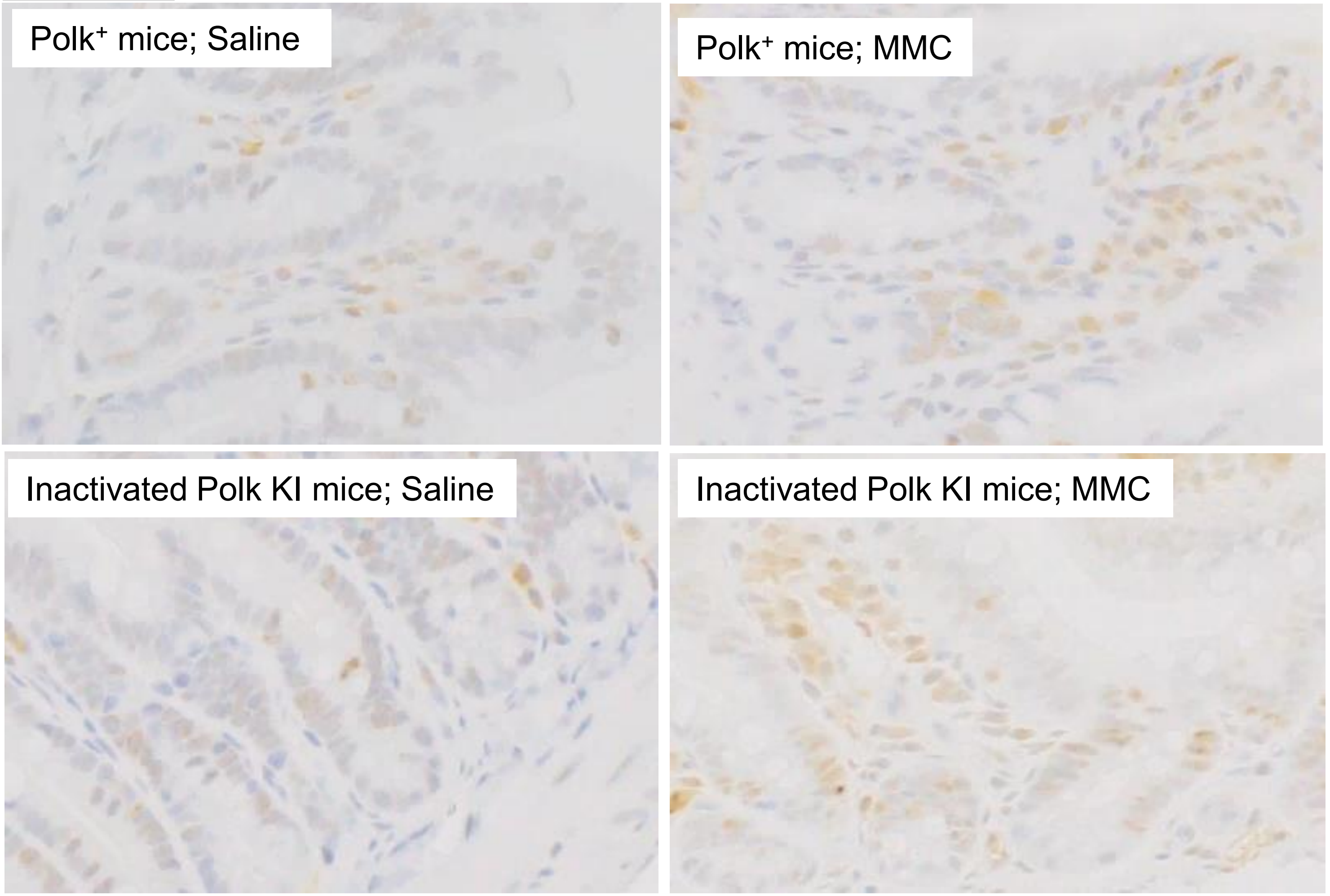

Mice;
Inactivated Polk KI mice, Polk^{+} mice

Treatment;
Saline: Saline x 5 days
MMC: Mitomycin C 1 mg/kg x 5 days

Staining;
HE; hematoxylin-eosin
γH2AX; γH2AX immunohistochemical stain

Bar represents 50 μm

Supplementary S12

Epididymis

HE

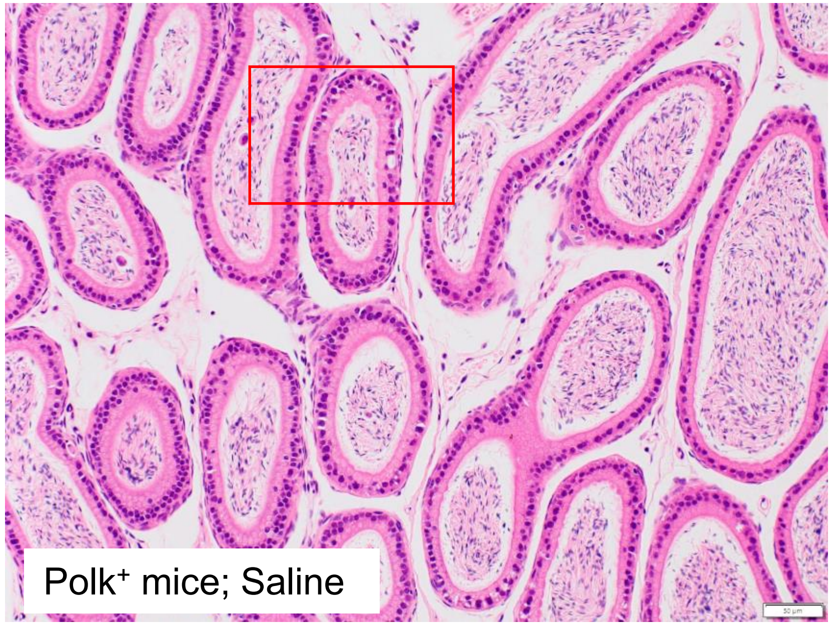

γH2AX

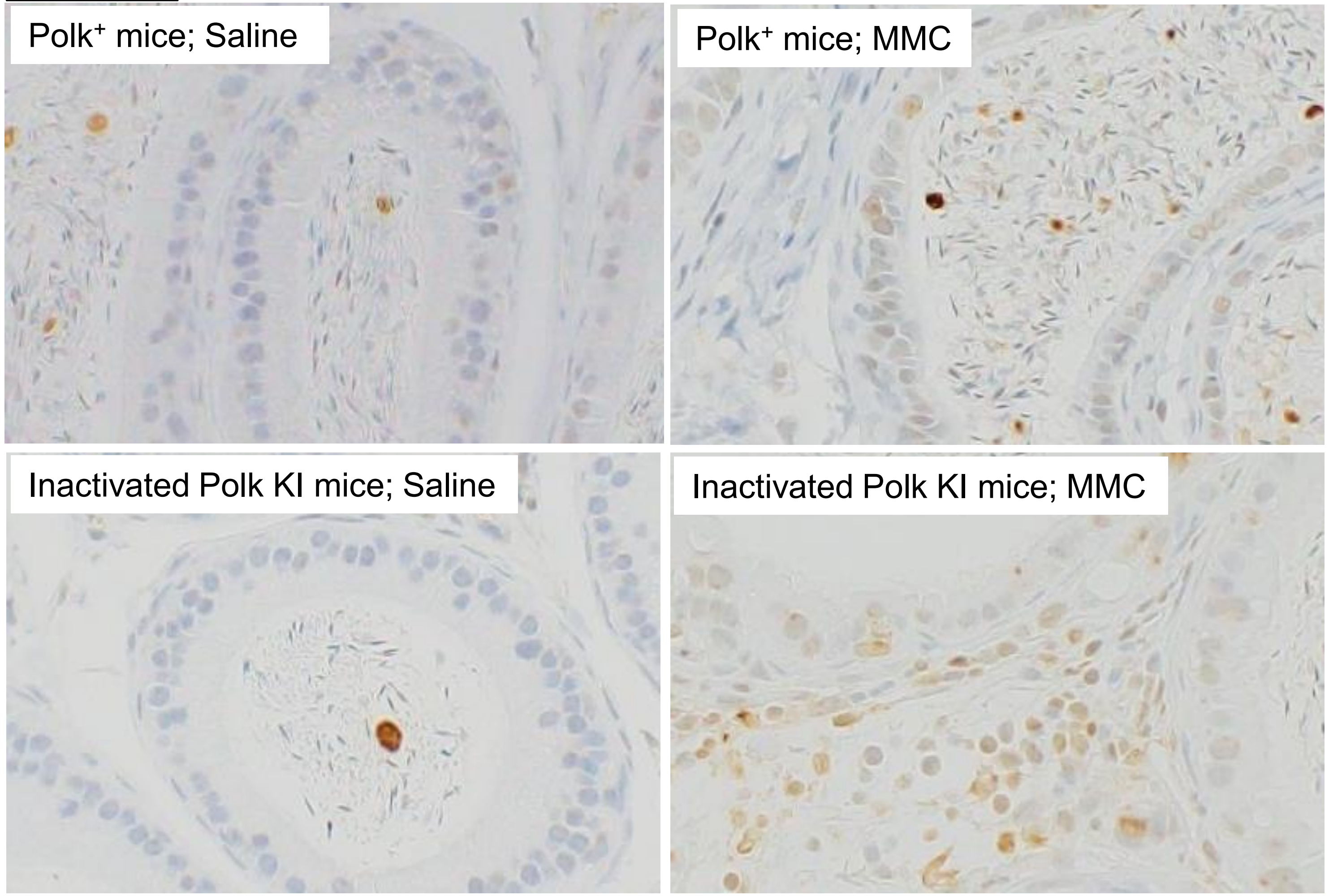

Mice;
Inactivated Polk KI mice, Polk⁺ mice

Treatment;
Saline: Saline x 5 days
MMC: Mitomycin C 1 mg/kg x 5 days

Staining;
HE; hematoxylin-eosin
γH2AX; γH2AX immunohistochemical stain

Bar represents 50 μm

Supplementary S13

lung

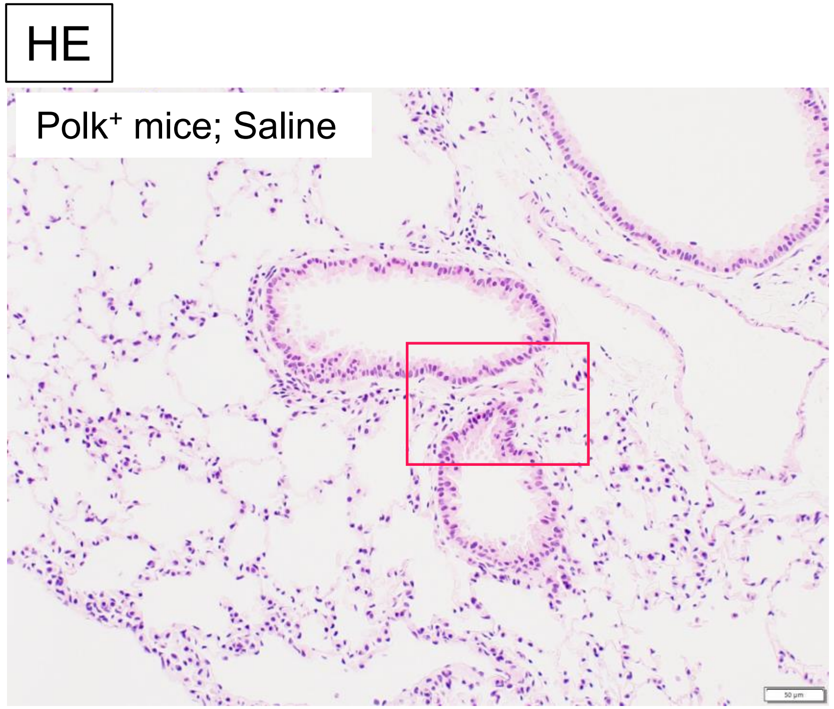

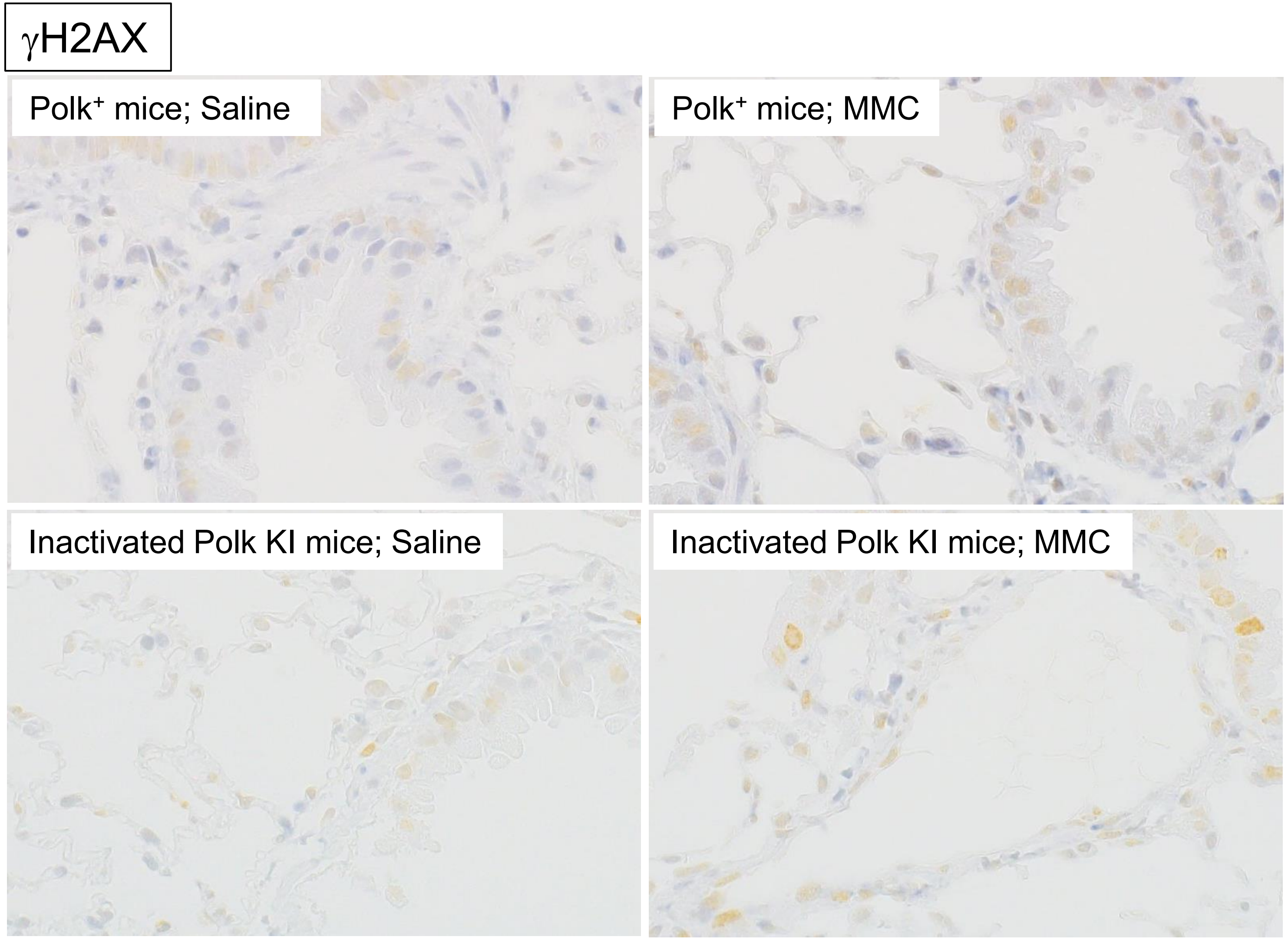

Mice;
  Inactivated Polk KI mice, $Polk^{+}$ mice

Treatment;
  Saline: Saline x 5 days
  MMC: Mitomycin C 1 mg/kg x 5 days

Staining;
  HE; hematoxylin-eosin
  γH2AX; γH2AX immunohistochemical stain

Bar represents 50 μm

Supplementary S14

kidney

HE

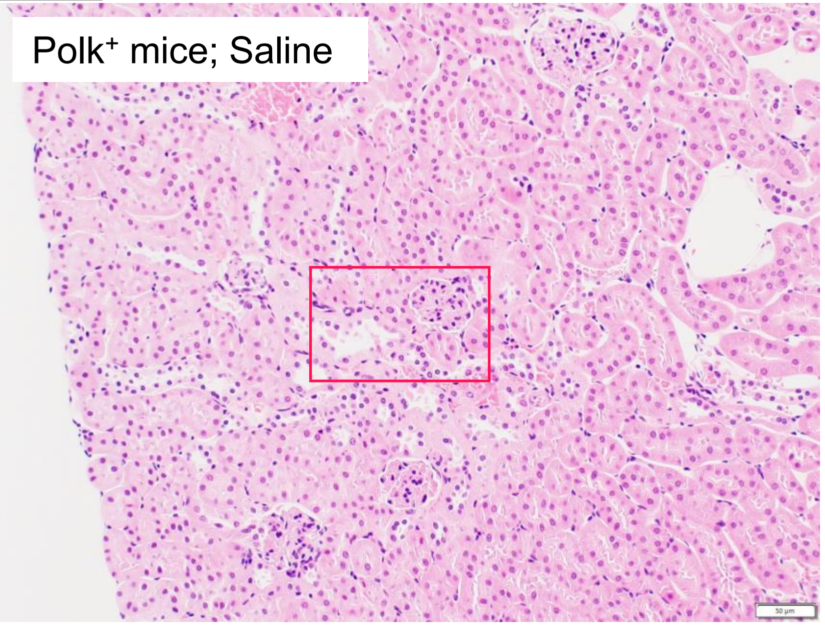

γH2AX

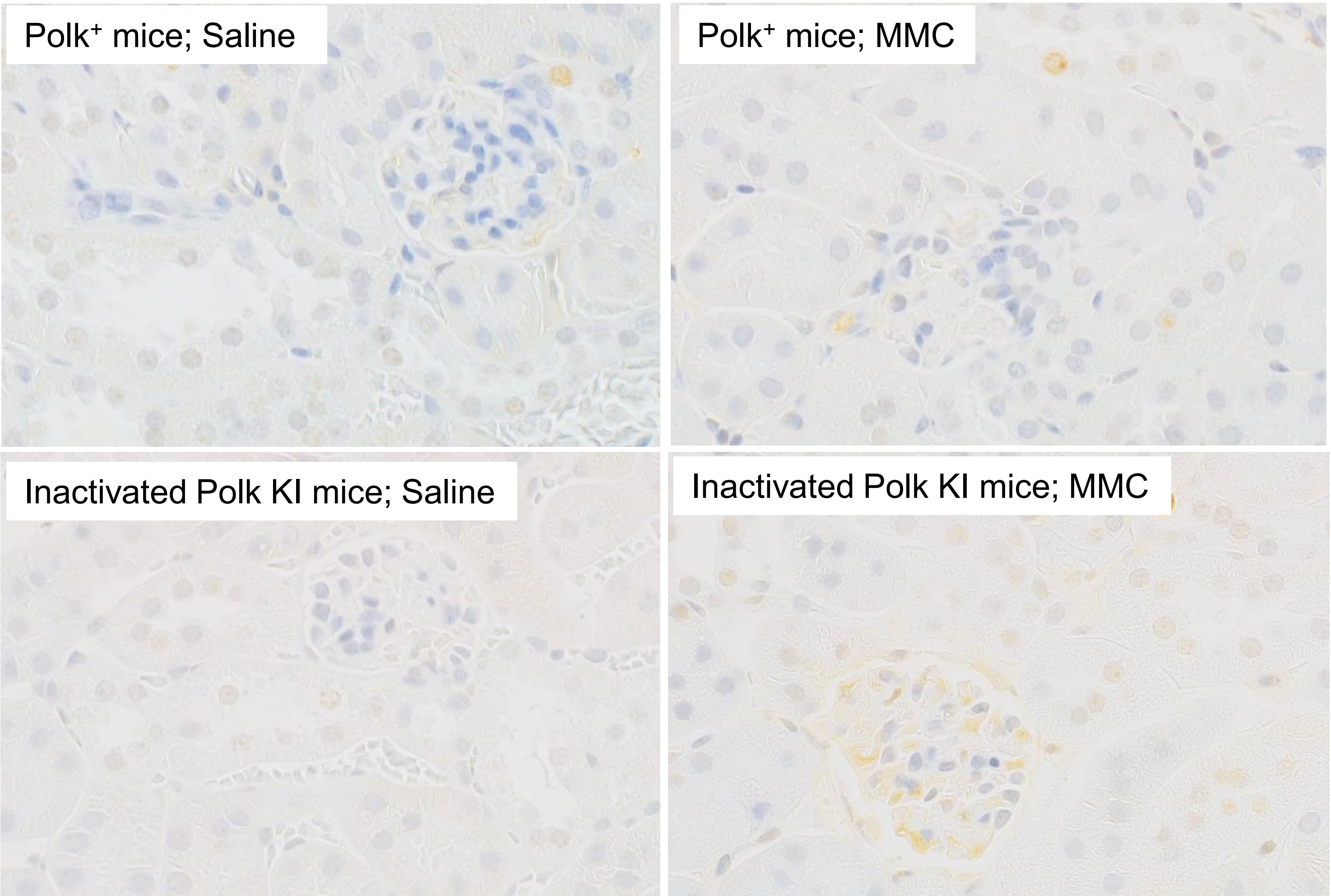

Mice;
Inactivated Polk KI mice, Polk^{+} mice

Treatment;
Saline: Saline x 5 days
MMC: Mitomycin C 1 mg/kg x 5 days

Staining;
HE; hematoxylin-eosin
γH2AX; γH2AX immunohistochemical stain

Bar represents 50 μm

Supplementary S15

Skin (hair follicle)

HE

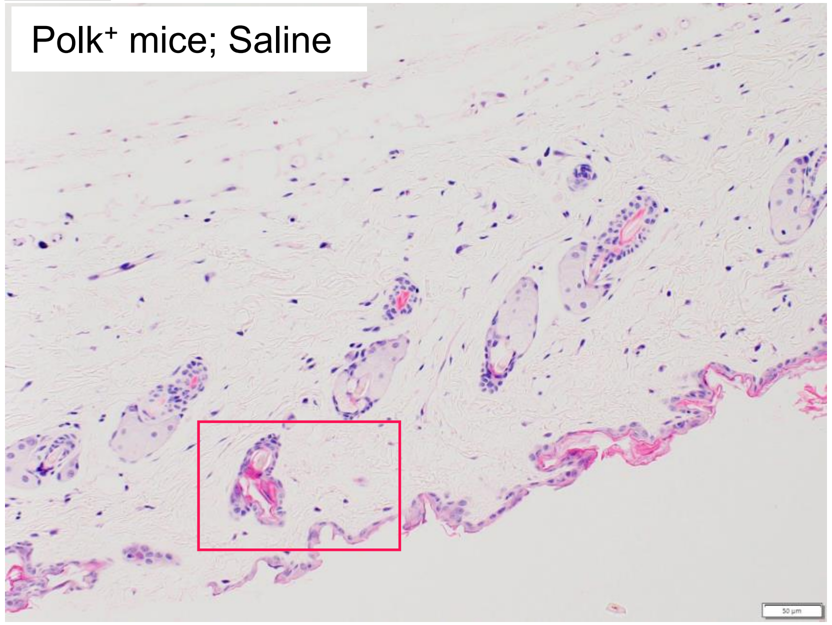

γH2AX

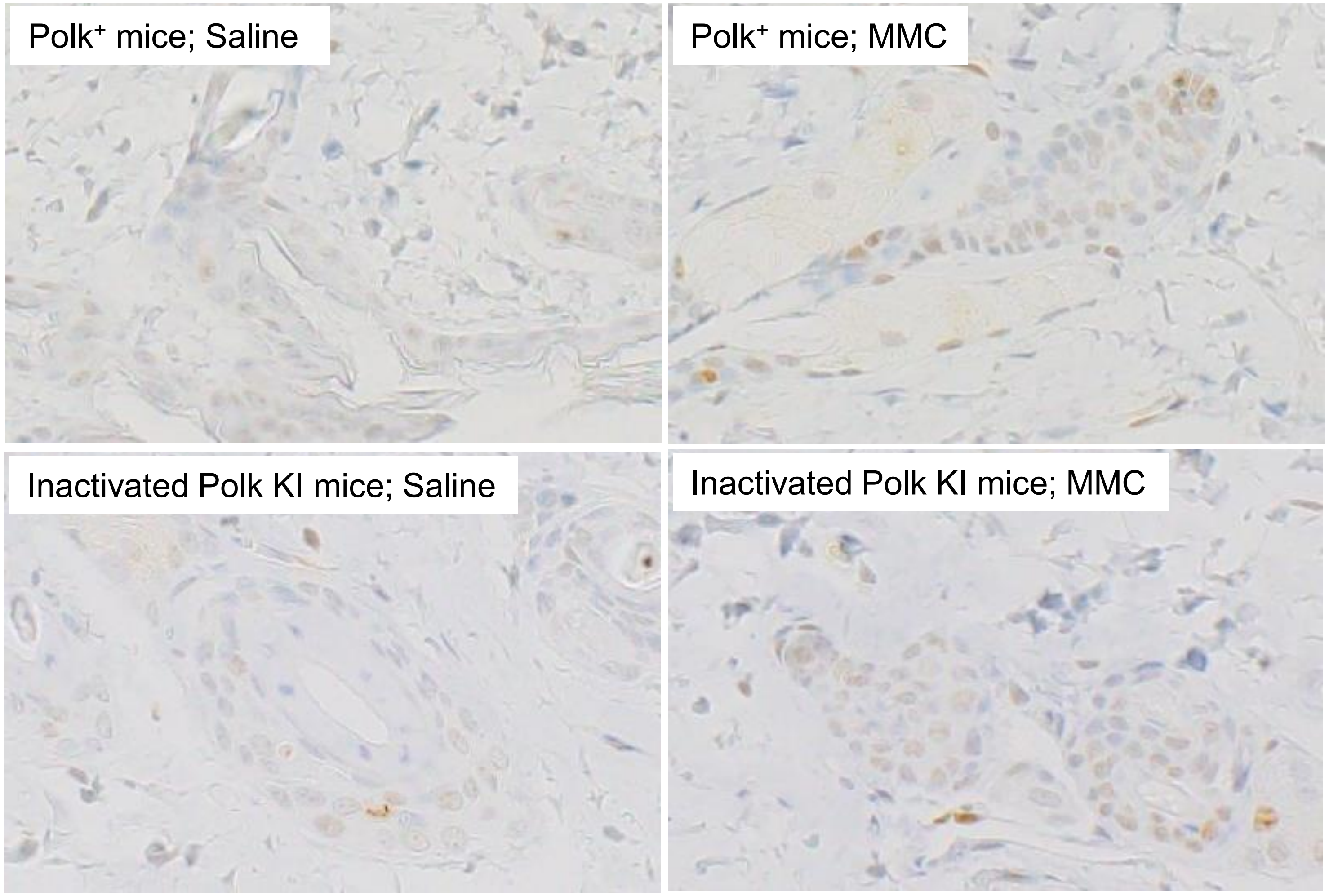

Mice;
Inactivated Polk KI mice, Polk+ mice

Treatment;
Saline: Saline x 5 days
MMC: Mitomycin C 1 mg/kg x 5 days

Staining;
HE; hematoxylin-eosin
γH2AX; γH2AX immunohistochemical stain

Bar represents 50 μm

Supplementary S16

Cornea

HE

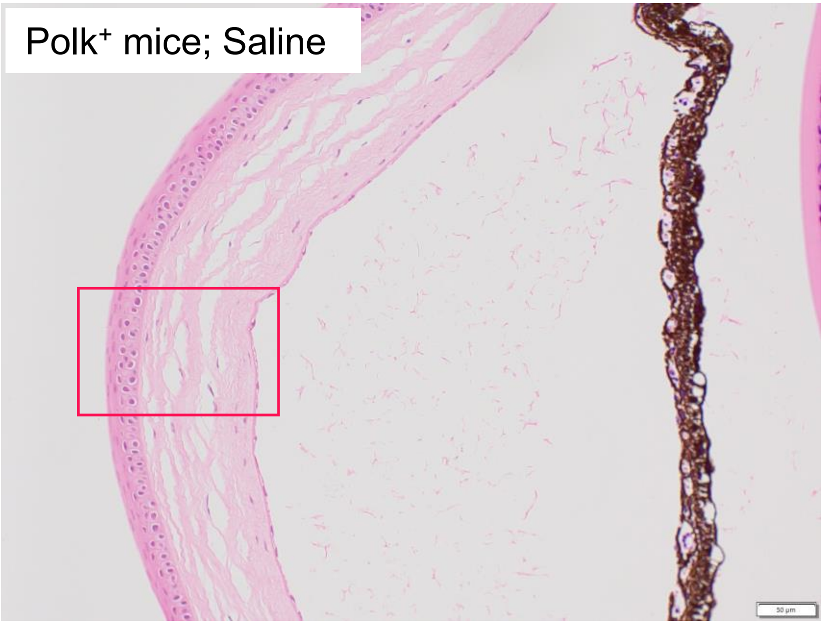

γH2AX

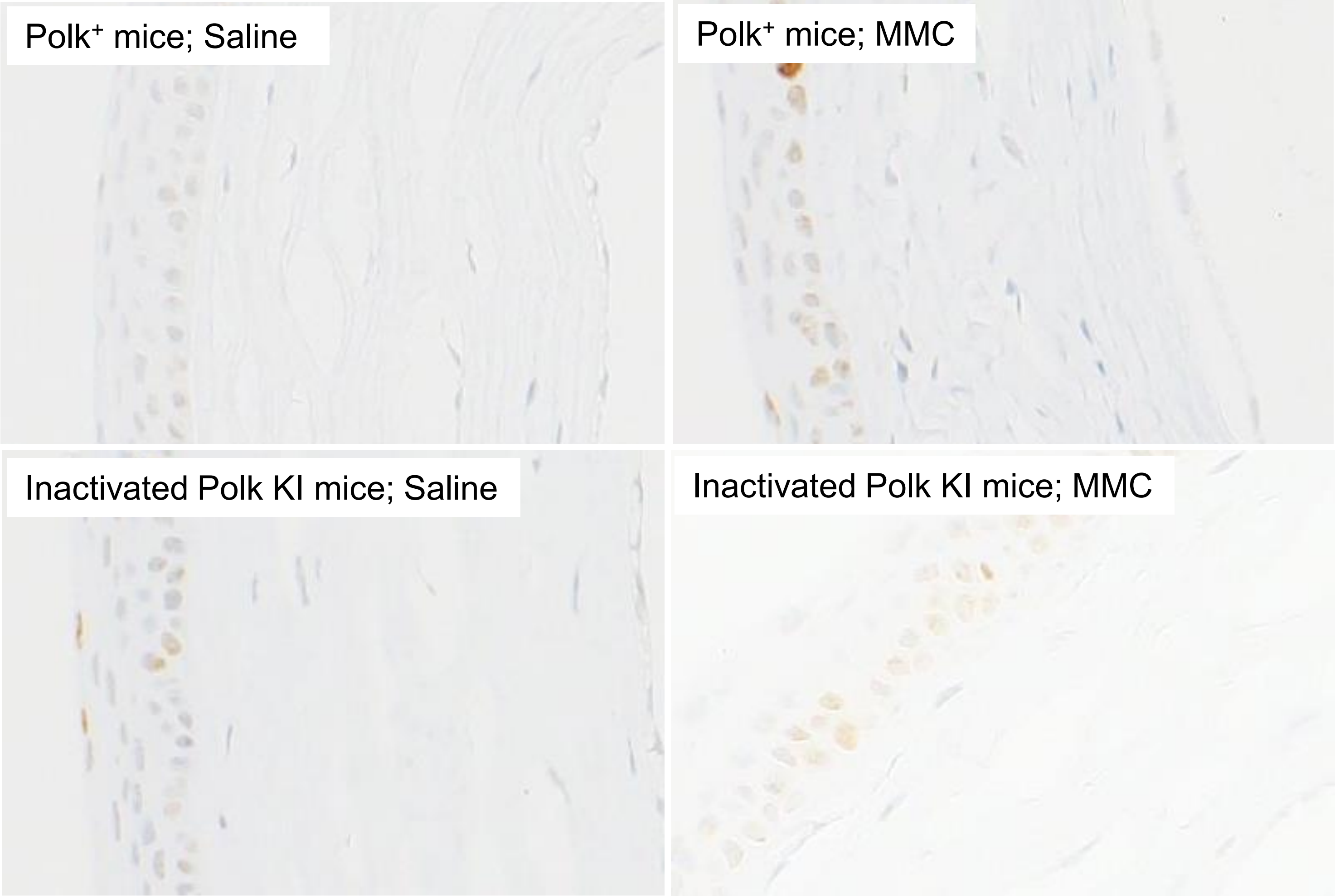

Mice;
  Inactivated Polk KI mice, Polk⁺ mice

Treatment;
  Saline: Saline x 5 days
  MMC: Mitomycin C 1 mg/kg x 5 days

Staining;
  HE; hematoxylin-eosin
  γH2AX; γH2AX immunohistochemical stain

Bar represents 50 μm

Supplementary S17

Epidermis

HE

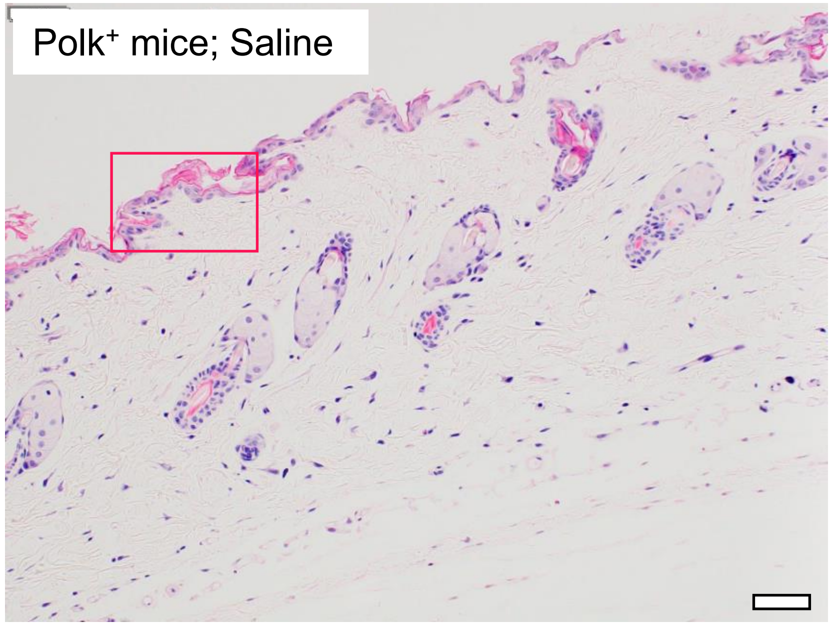

γH2AX

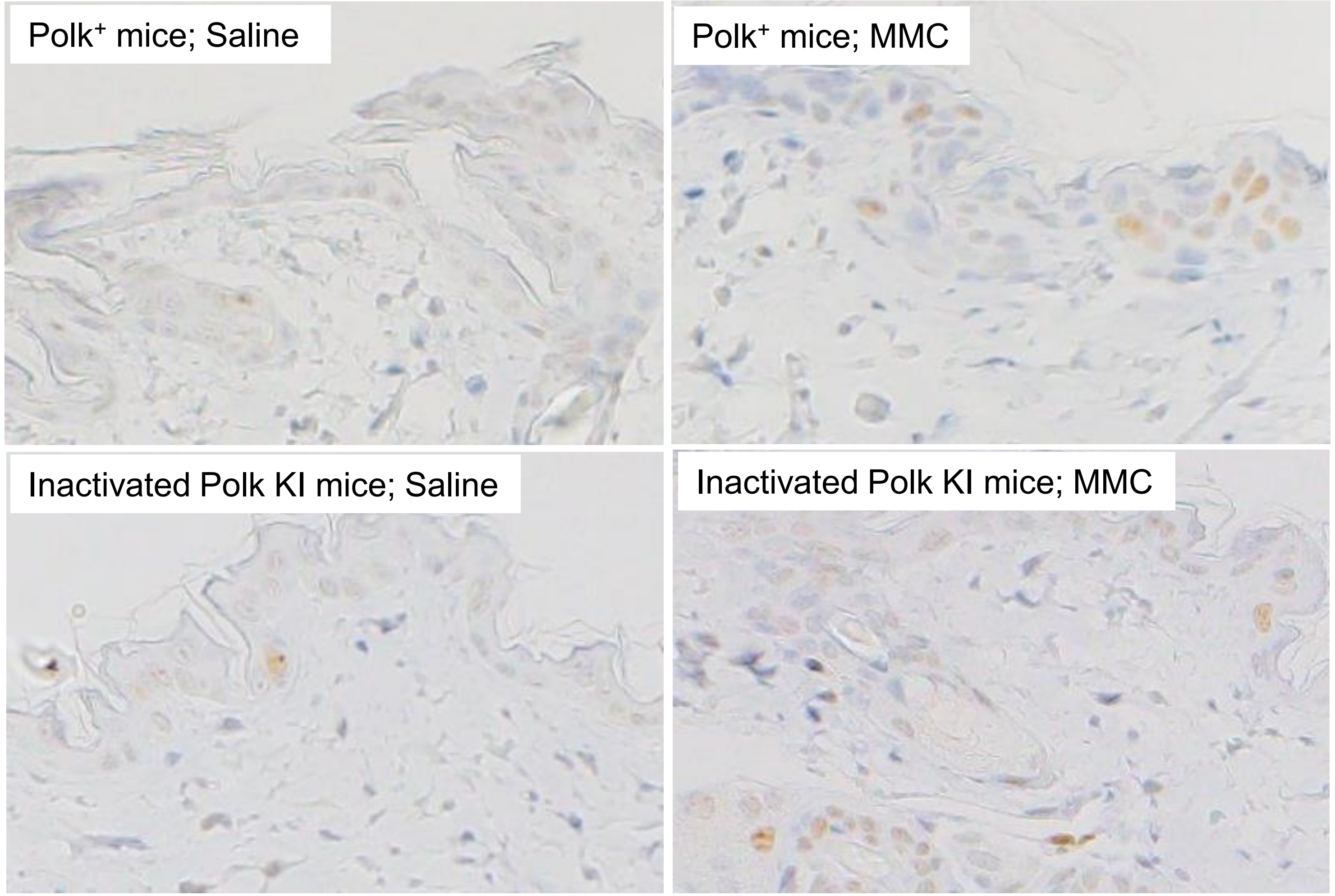

Mice;
Inactivated Polk KI mice, Polk$^+$ mice

Treatment;
Saline: Saline x 5 days
MMC: Mitomycin C 1 mg/kg x 5 days

Staining;
HE; hematoxylin-eosin
γH2AX; γH2AX immunohistochemical stain

Bar represents 50 μm

Supplementary S18

Colon

HE

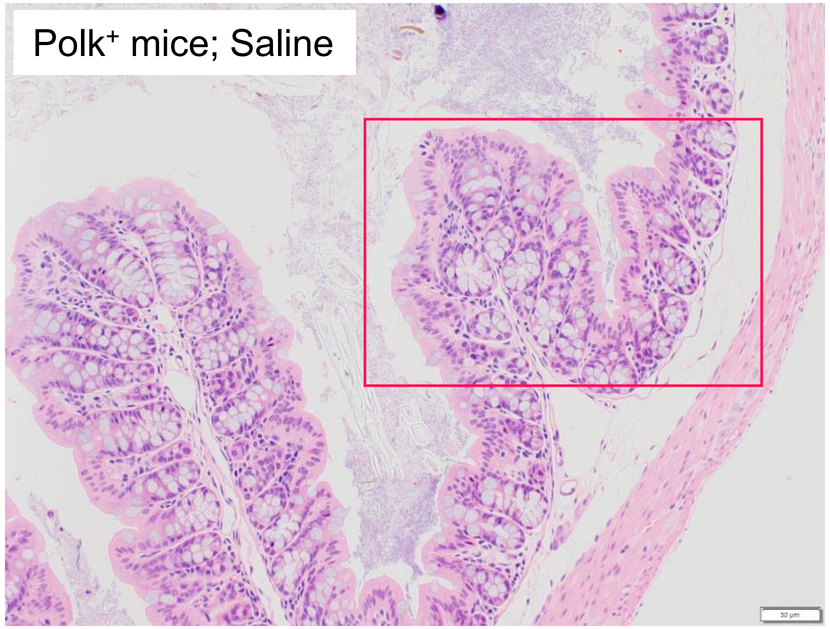

γH2AX

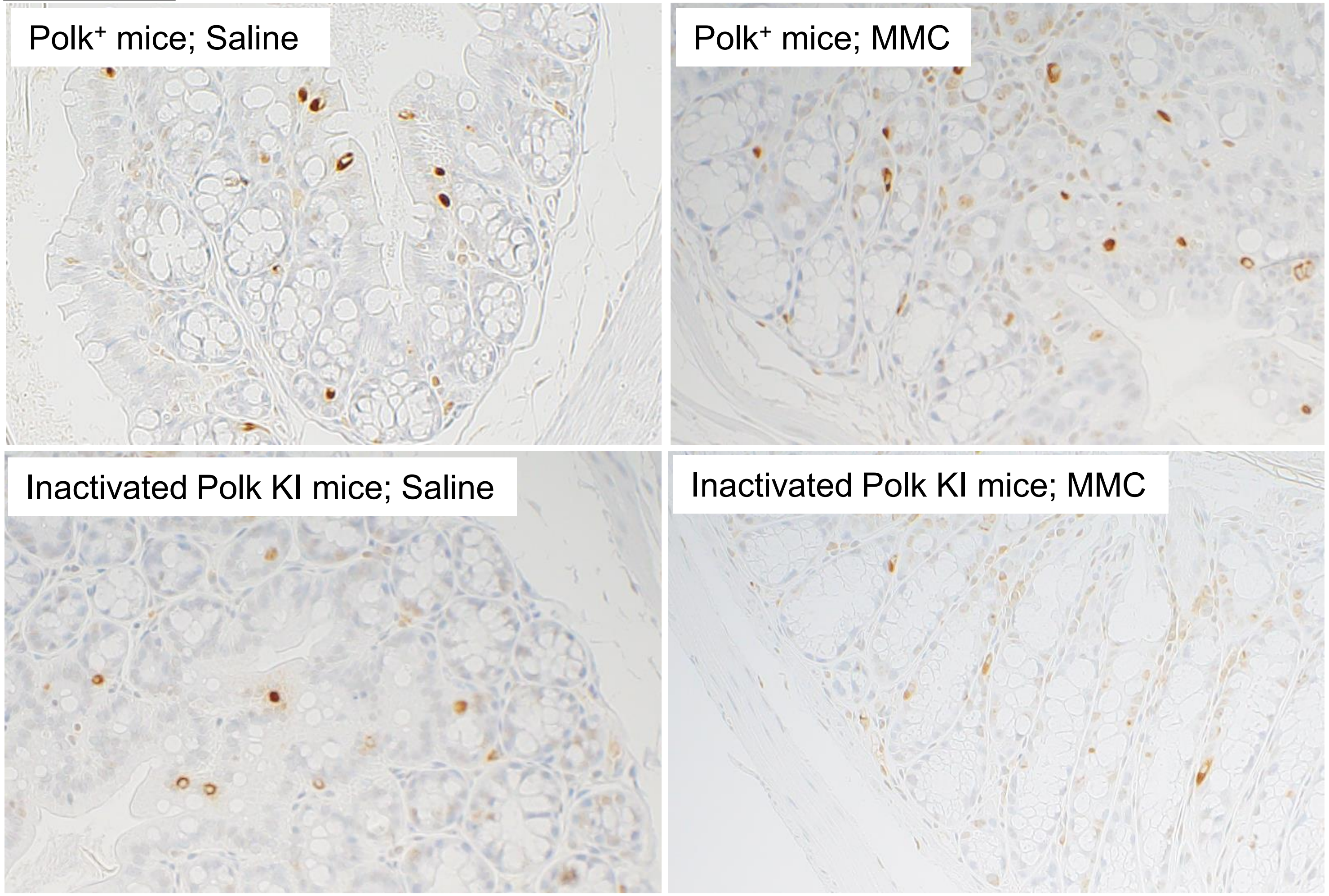

Mice;
Inactivated Polk KI mice, Polk$^+$ mice

Treatment;
Saline: Saline x 5 days
MMC: Mitomycin C 1 mg/kg x 5 days

Staining;
HE; hematoxylin-eosin
γH2AX; γH2AX immunohistochemical stain

Bar represents 50 μm

Supplementary S19

Tongue skeletal muscle

HE

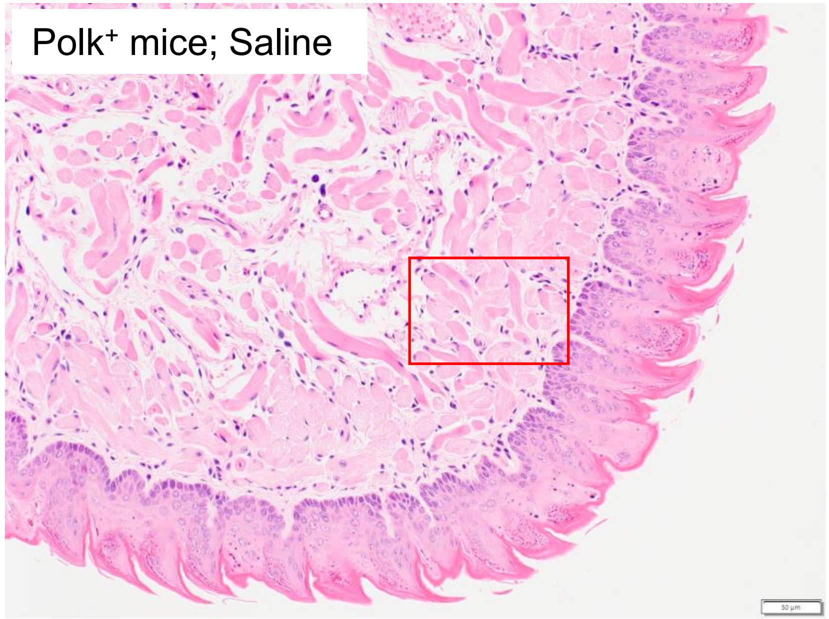

γH2AX

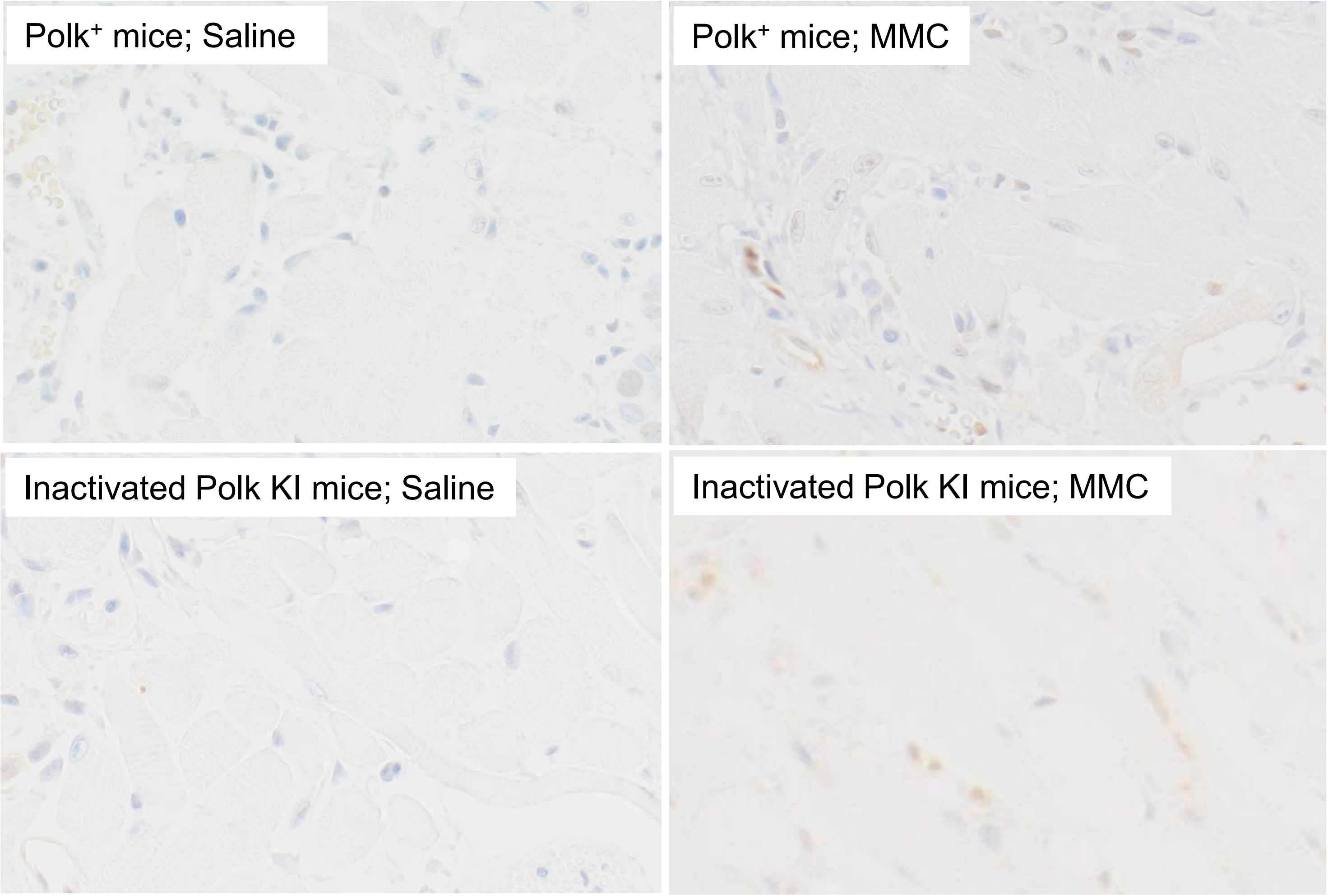

Mice;
Inactivated Polk KI mice, Polk$^+$ mice

Treatment;
Saline: Saline x 5 days
MMC: Mitomycin C 1 mg/kg x 5 days

Staining;
HE; hematoxylin-eosin
γH2AX; γH2AX immunohistochemical stain

Bar represents 50 μm

Supplementary S20

Heart

HE

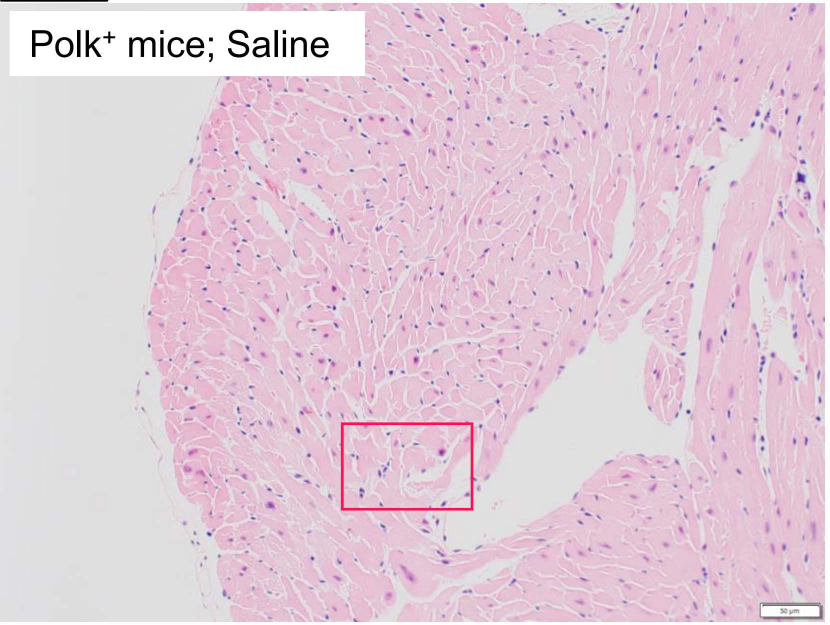

γH2AX

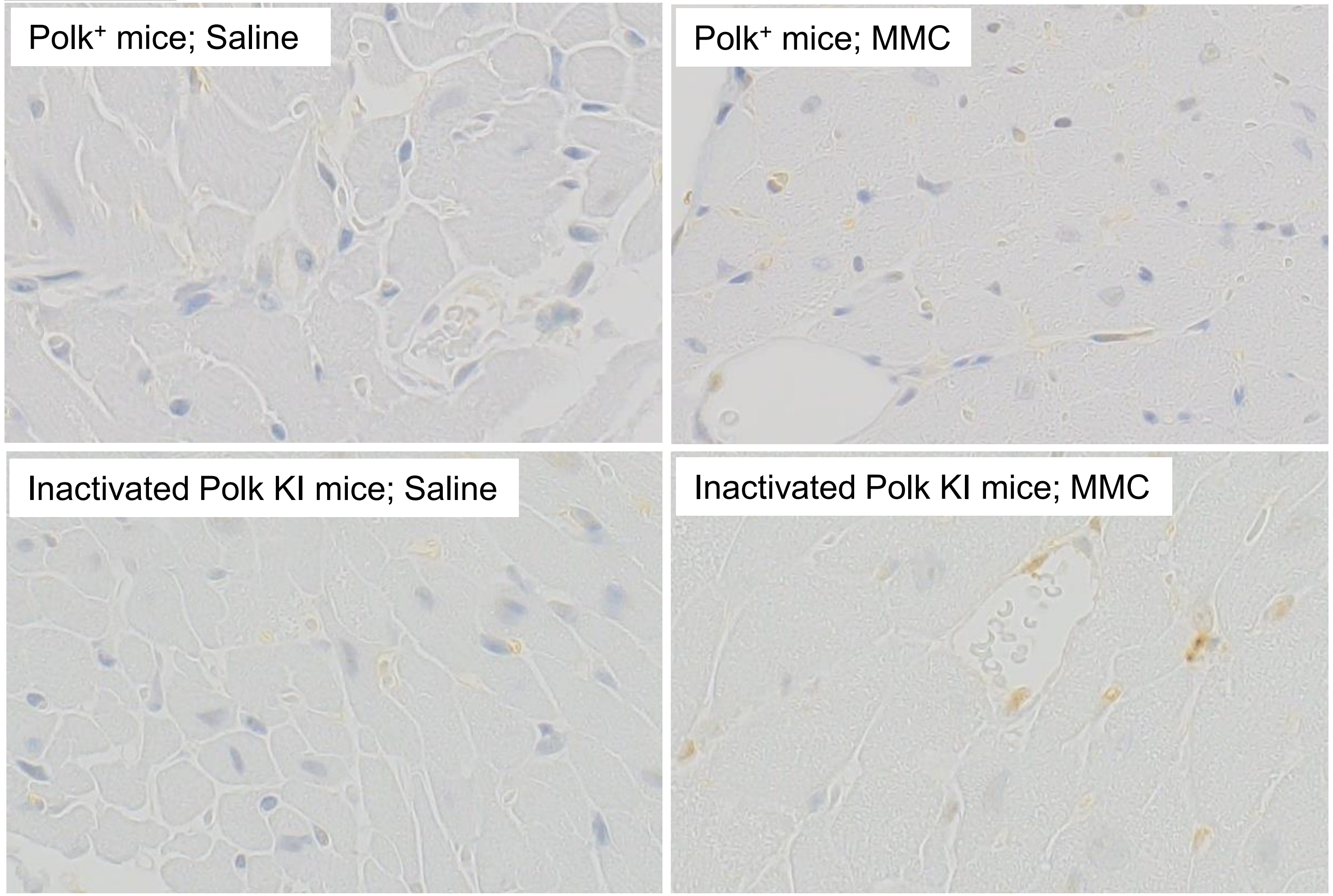

Mice;
Inactivated Polk KI mice, Polk^{+} mice

Treatment;
Saline: Saline x 5 days
MMC: Mitomycin C 1 mg/kg x 5 days

Staining;
HE; hematoxylin-eosin
γH2AX; γH2AX immunohistochemical stain

Bar represents 50 μm

Supplementary S21

Tracheal epithelium

HE

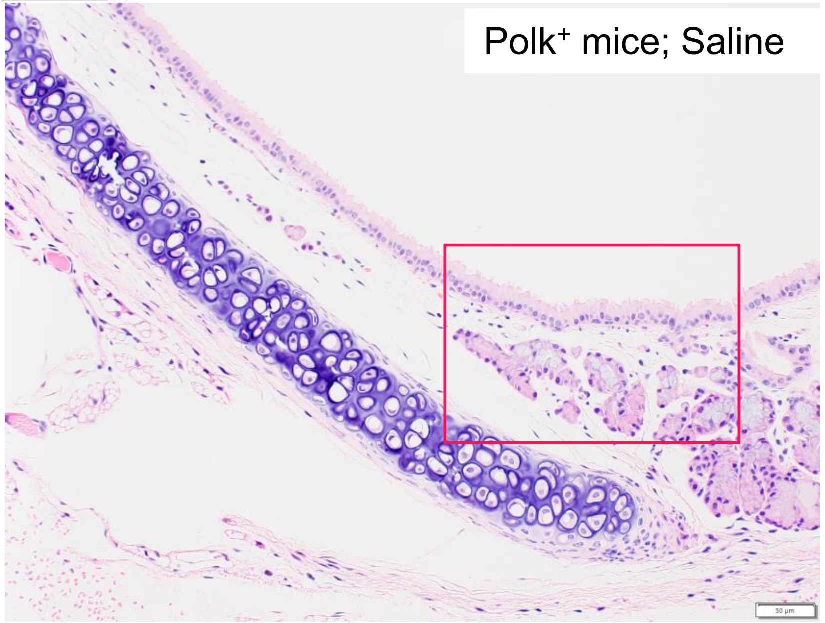

γH2AX

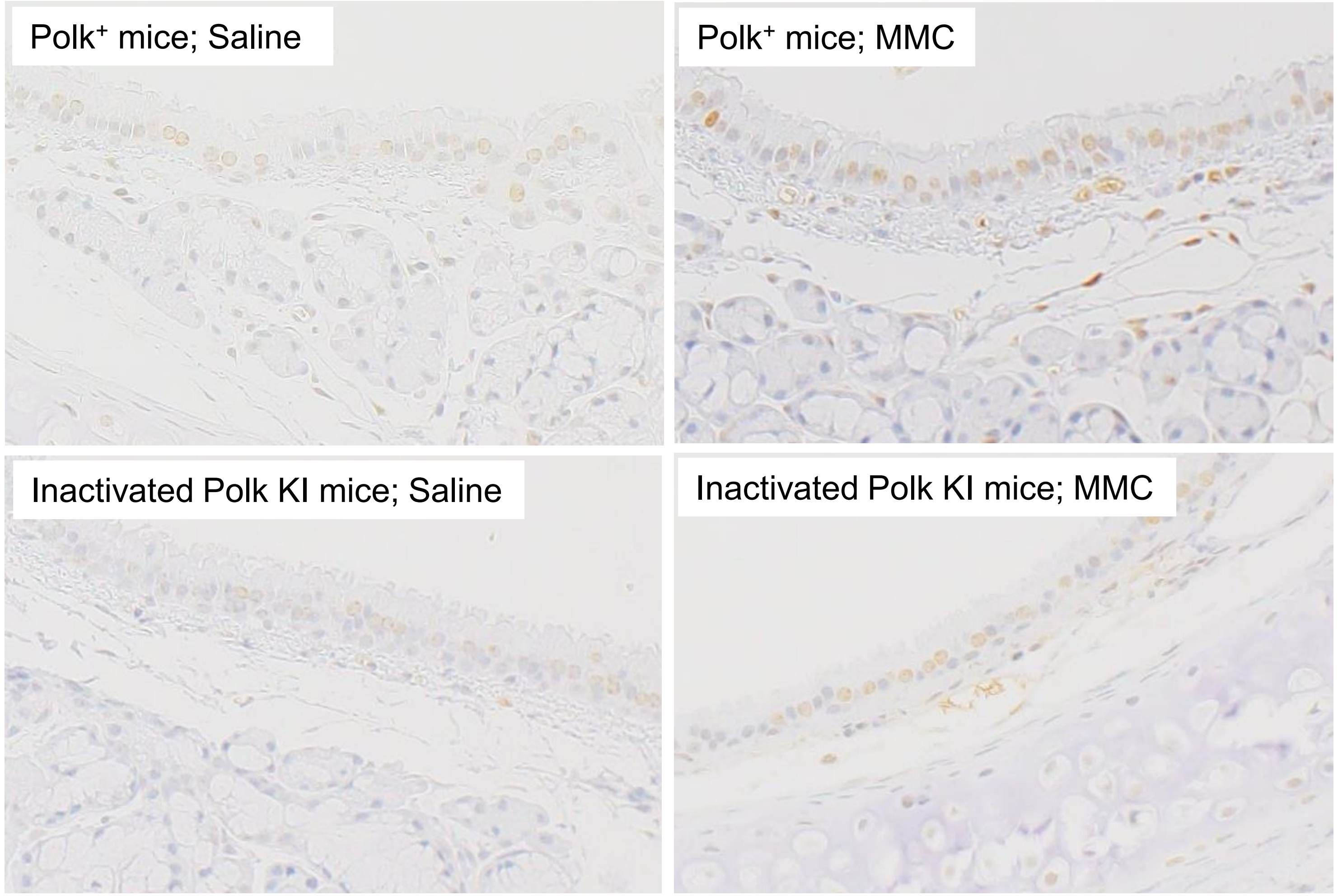

Mice;
Inactivated Polk KI mice, Polk^{+} mice

Treatment;
Saline: Saline x 5 days
MMC: Mitomycin C 1 mg/kg x 5 days

Staining;
HE; hematoxylin-eosin
γH2AX; γH2AX immunohistochemical stain

Bar represents 50 μm

Supplementary S22

Harder gland

HE

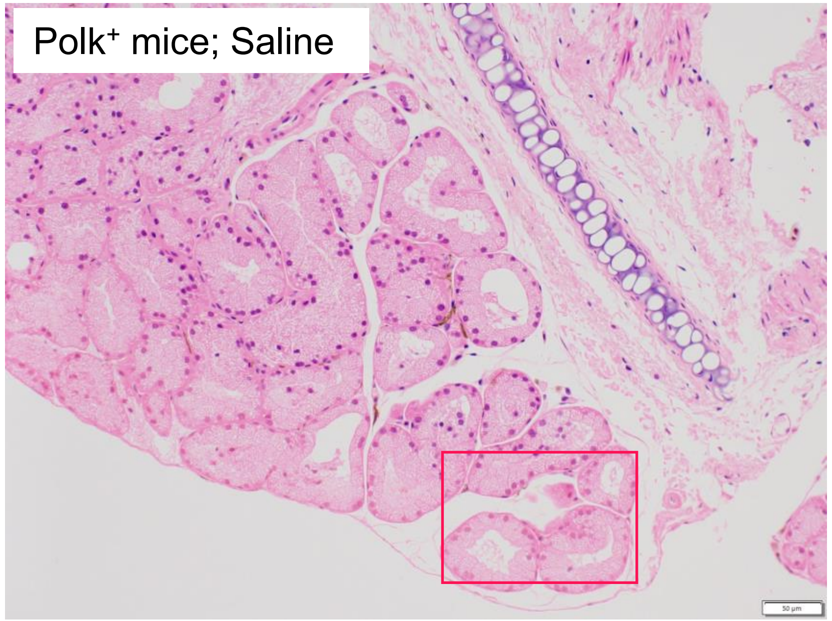

γH2AX

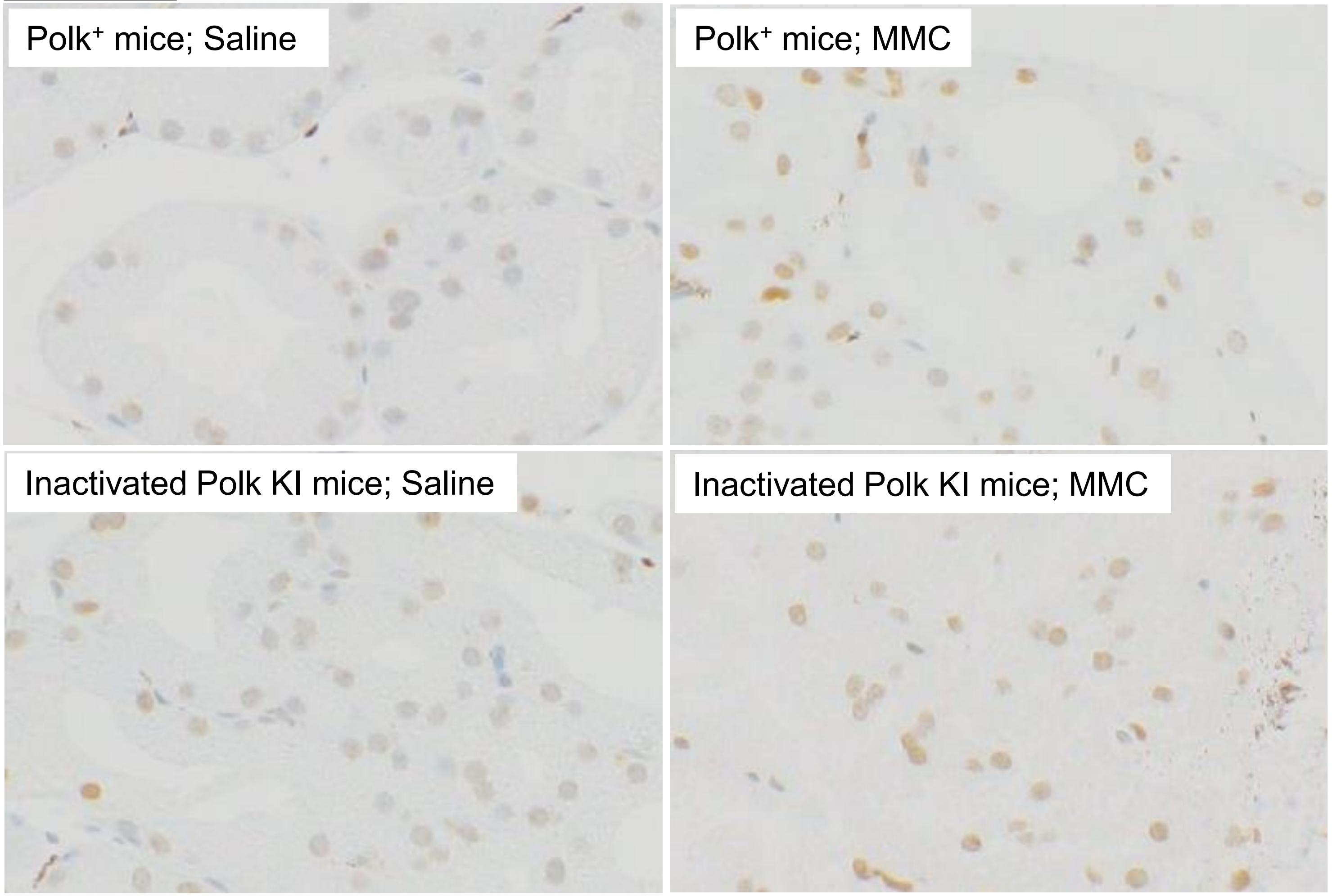

Mice;
Inactivated Polk KI mice, Polk⁺ mice

Treatment;
Saline: Saline x 5 days
MMC: Mitomycin C 1 mg/kg x 5 days

Staining;
HE; hematoxylin-eosin
γH2AX; γH2AX immunohistochemical stain

Bar represents 50 μm

Supplementary S23

Pancreas

HE

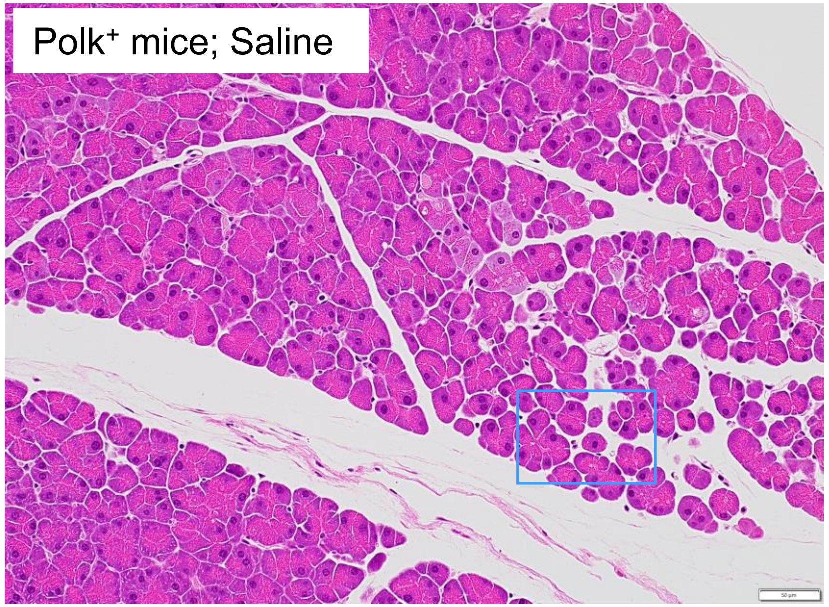

γH2AX

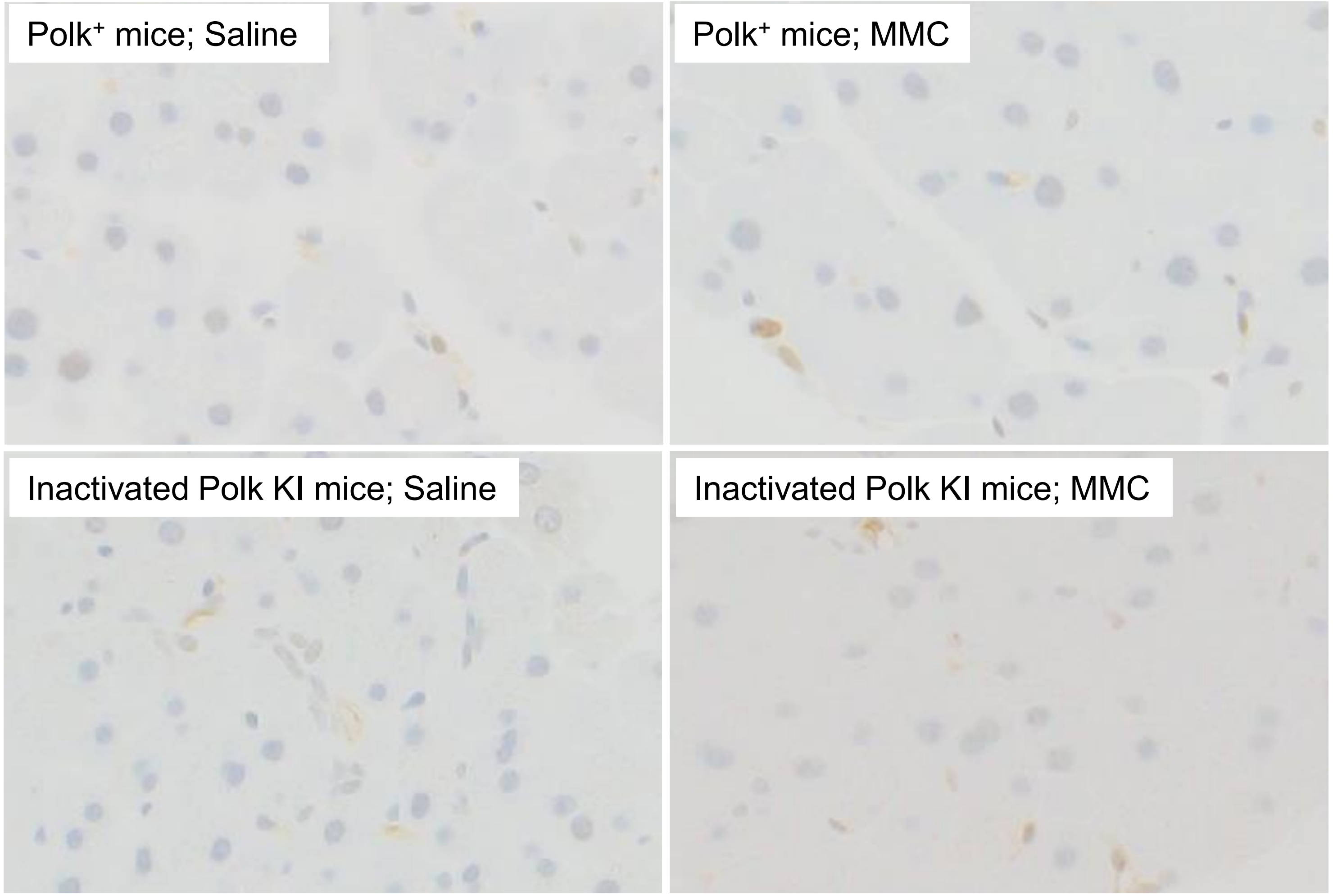

Mice;
Inactivated Polk KI mice, Polk$^+$ mice

Treatment;
Saline: Saline x 5 days
MMC: Mitomycin C 1 mg/kg x 5 days

Staining;
HE; hematoxylin-eosin
γH2AX; γH2AX immunohistochemical stain

Bar represents 50 μm

Supplementary S24

Brainstem

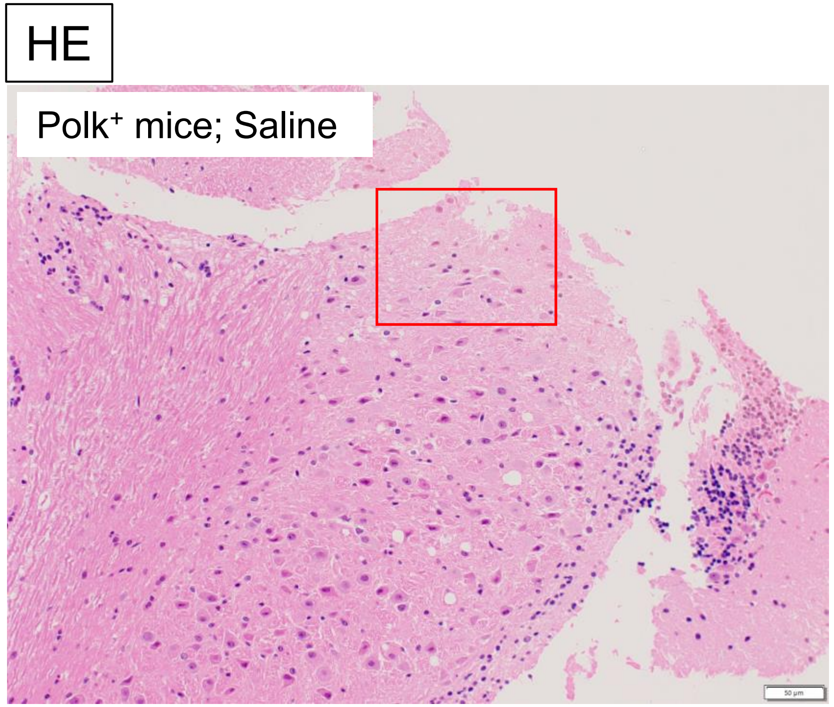

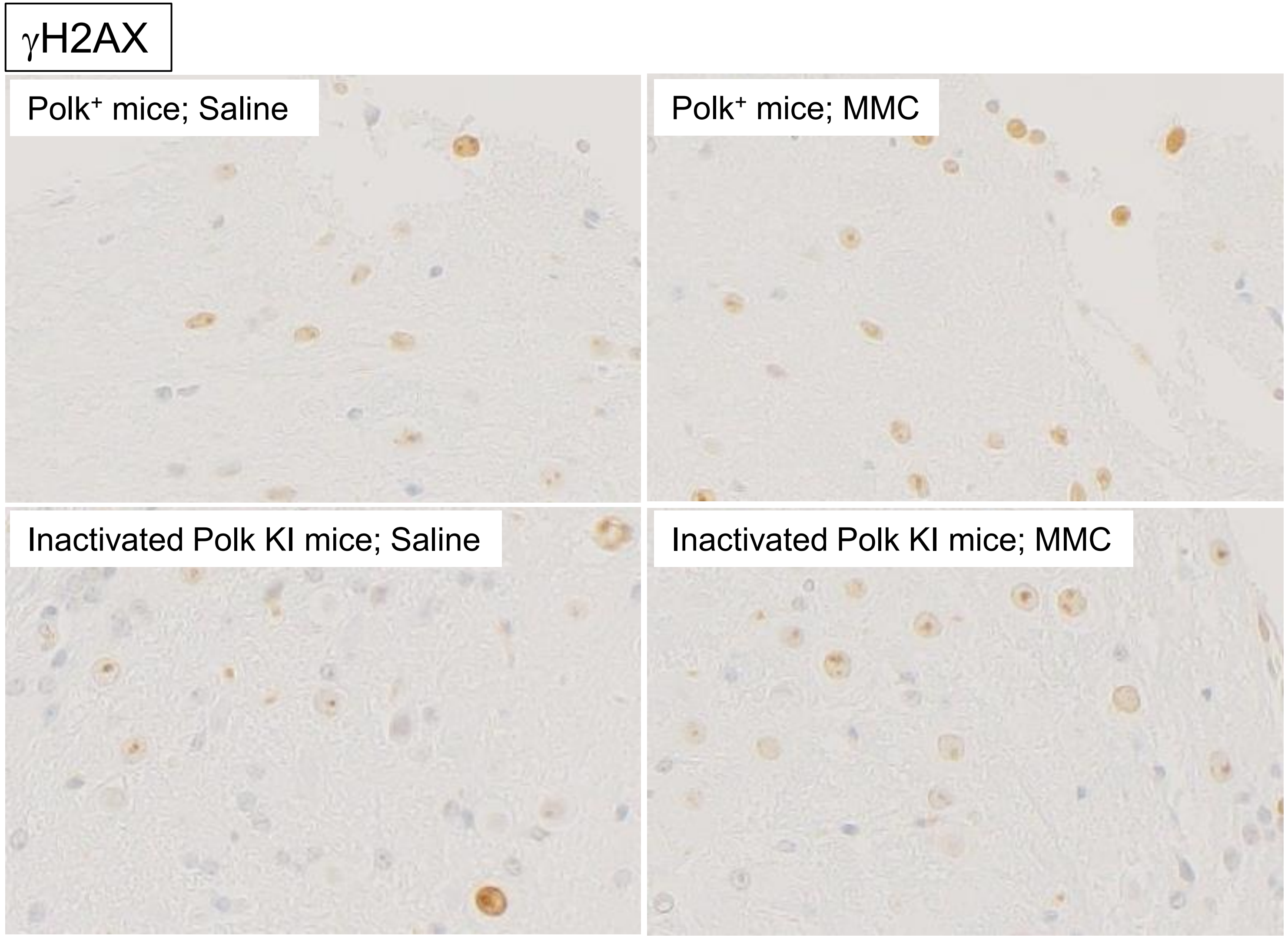

Mice;
Inactivated Polk KI mice, Polk^{+} mice

Treatment;
Saline: Saline x 5 days
MMC: Mitomycin C 1 mg/kg x 5 days

Staining;
HE; hematoxylin-eosin
γH2AX; γH2AX immunohistochemical stain

Bar represents 50 μm

Supplementary S25

Mesenteric lymph node

HE

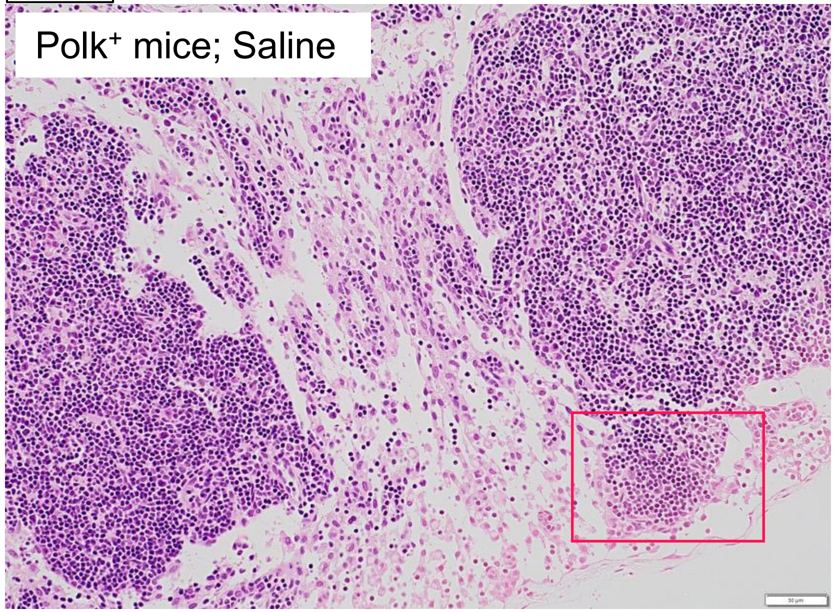

γH2AX

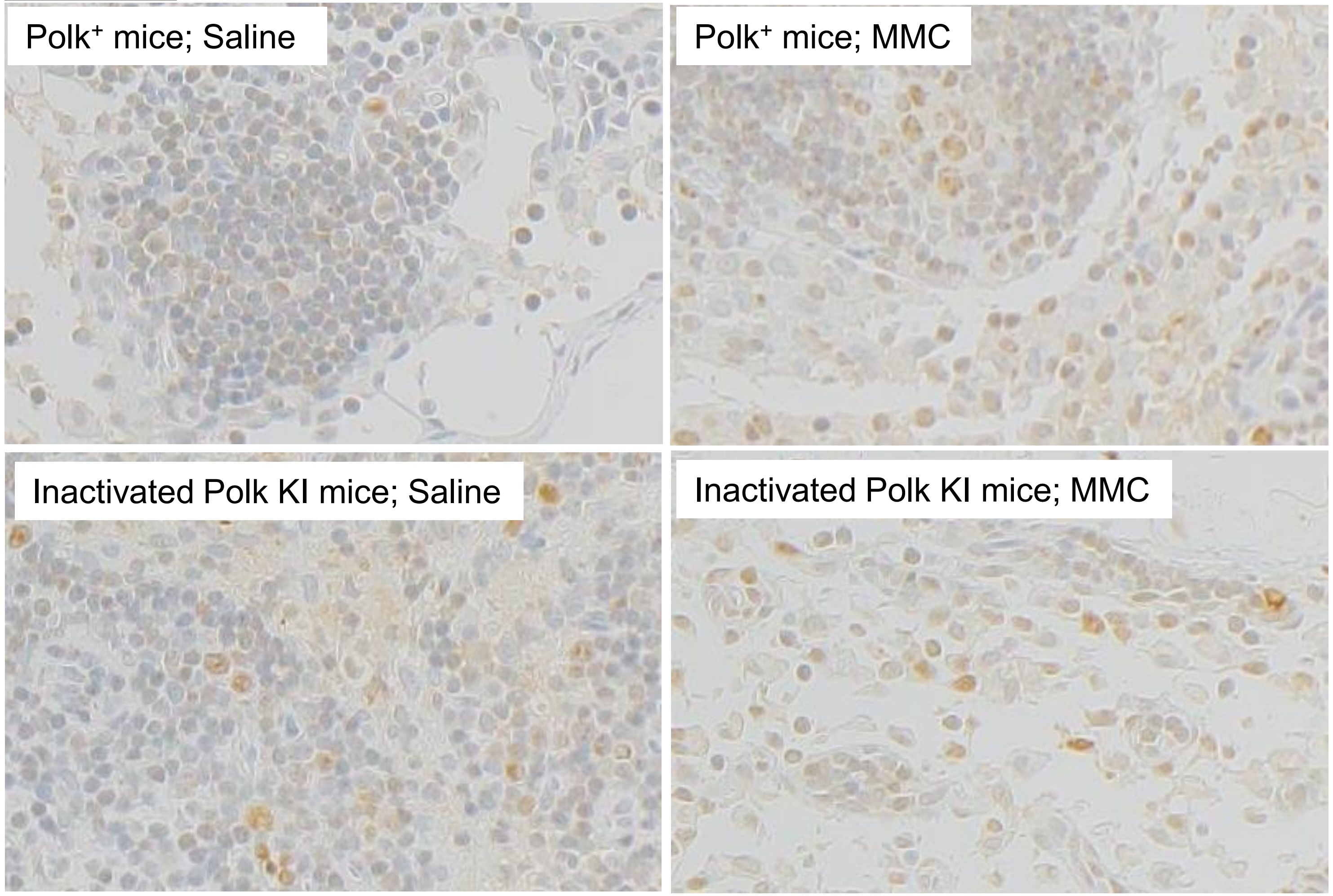

Mice;
Inactivated Polk KI mice, Polk^{+} mice

Treatment;
Saline: Saline x 5 days
MMC: Mitomycin C 1 mg/kg x 5 days

Staining;
HE; hematoxylin-eosin
γH2AX; γH2AX immunohistochemical stain

Bar represents 50 μm

Supplementary S26

Prostate

HE

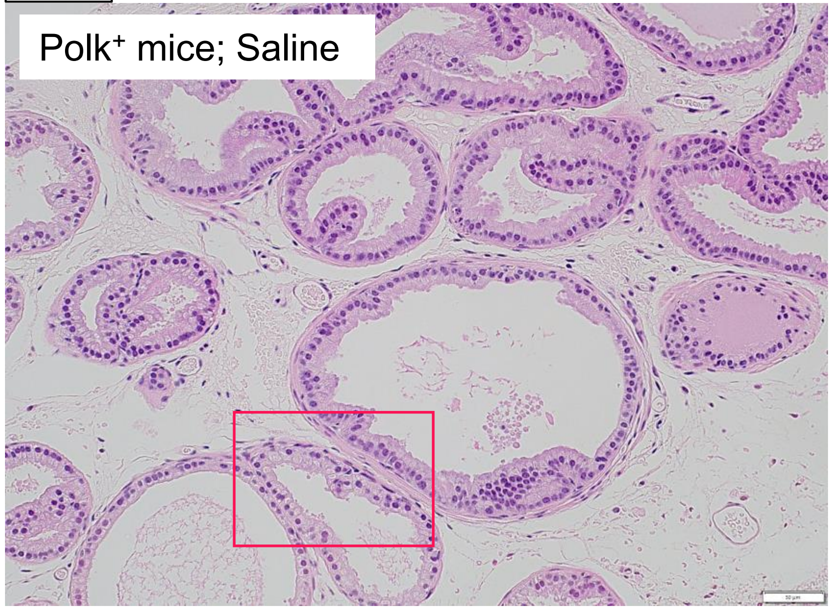

γH2AX

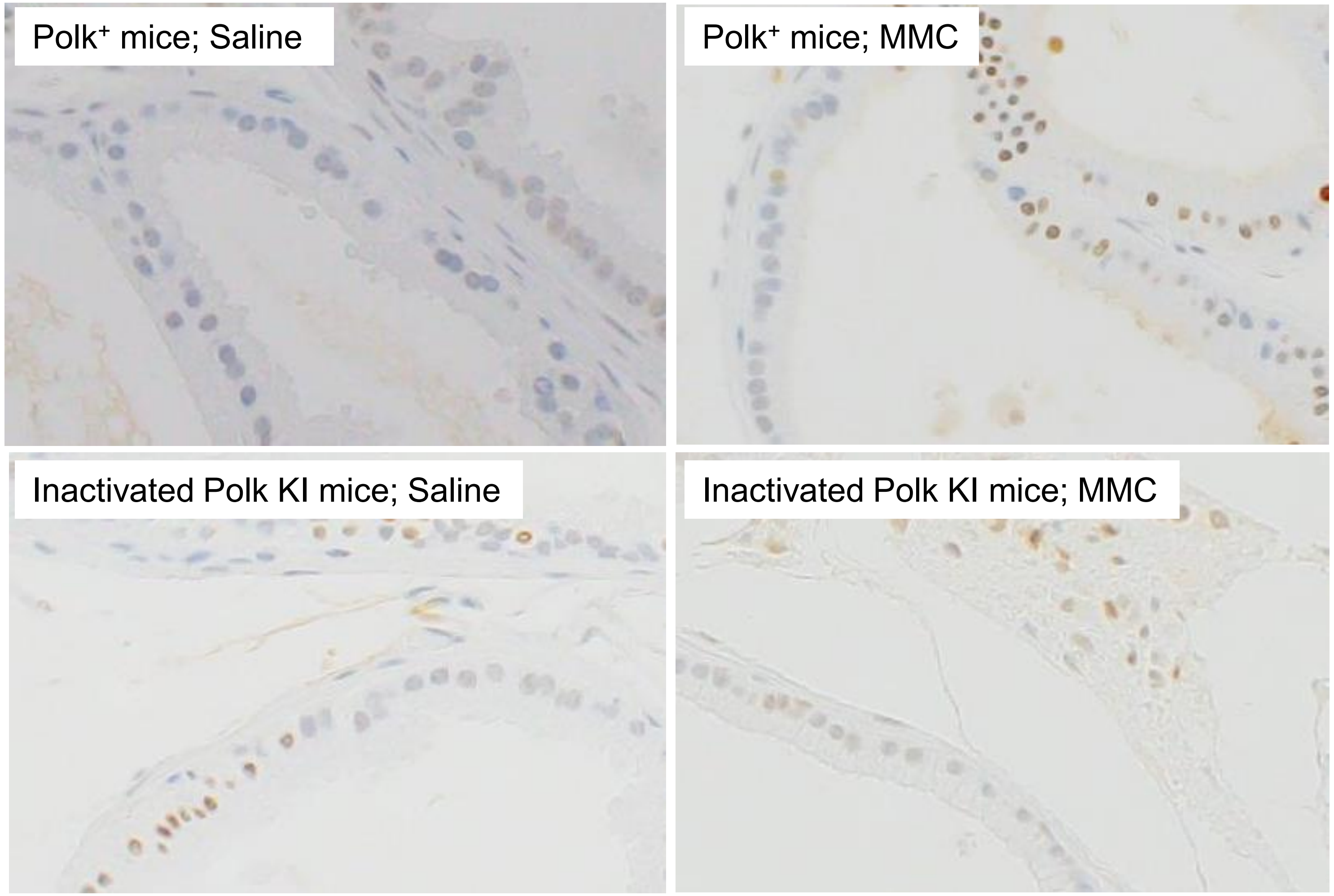

Mice;
Inactivated Polk KI mice, Polk^{+} mice

Treatment;
Saline: Saline x 5 days
MMC: Mitomycin C 1 mg/kg x 5 days

Staining;
HE; hematoxylin-eosin
γH2AX; γH2AX immunohistochemical stain

Bar represents 50 μm

Supplementary S27

Cerebellum

HE

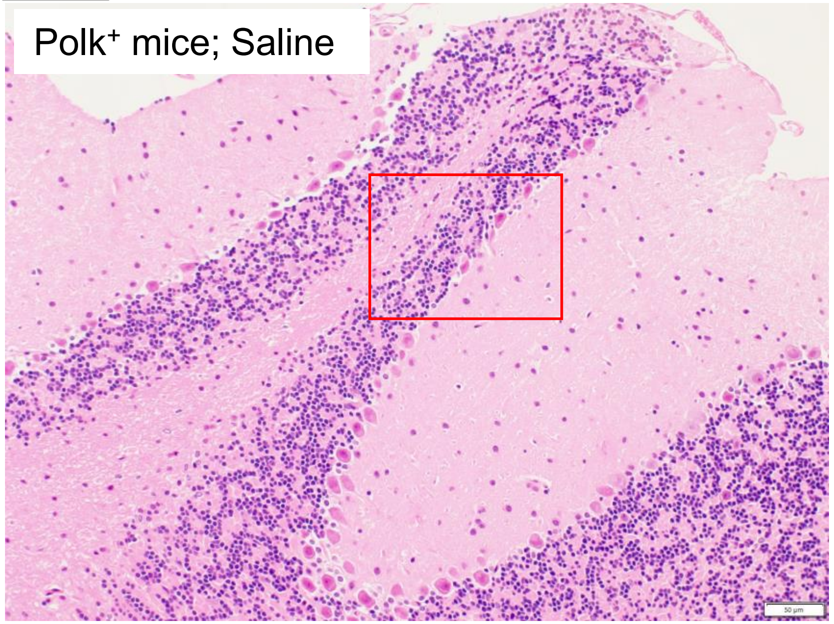

γH2AX

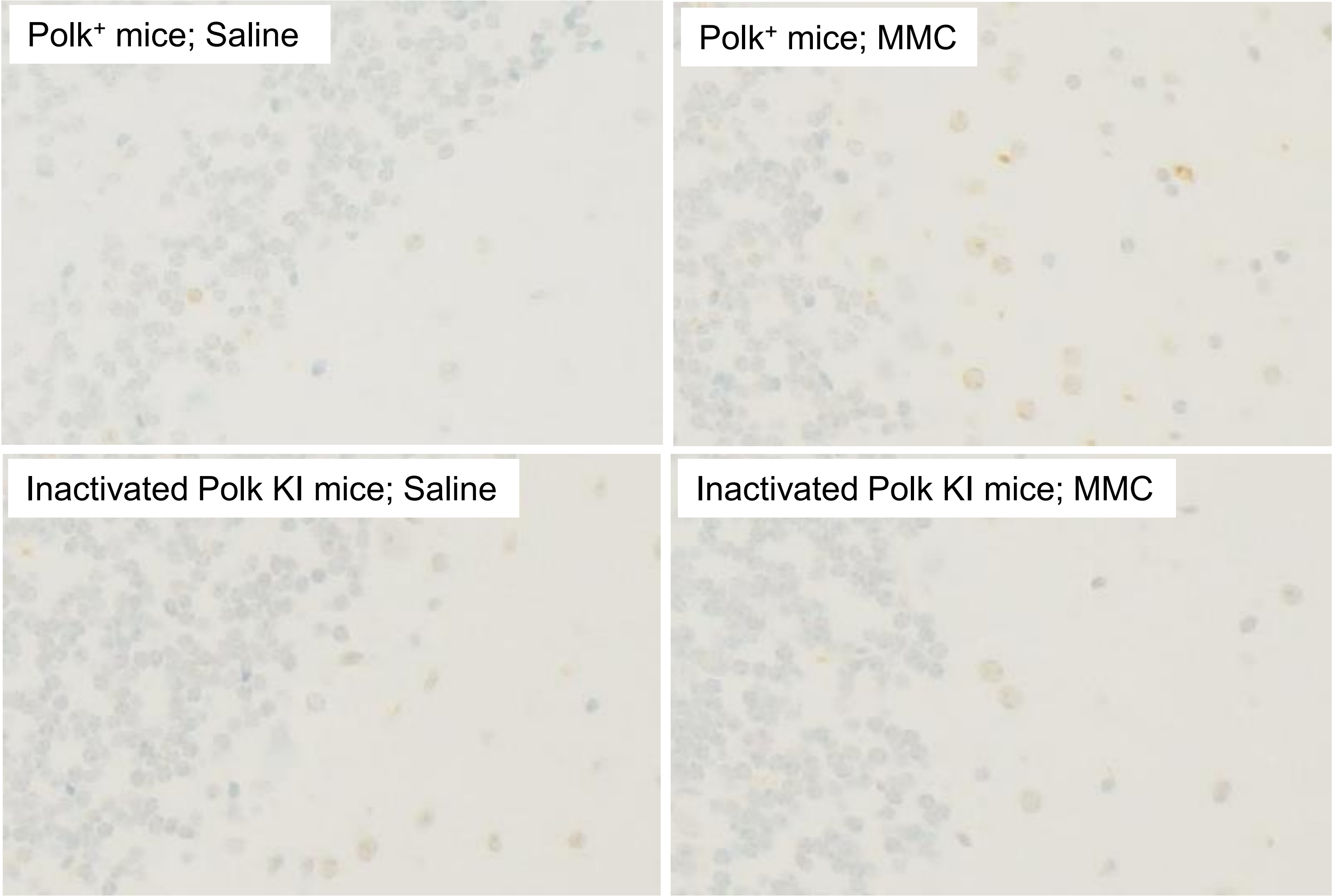

Mice;
Inactivated Polk KI mice, $Polk^{+}$ mice

Treatment;
Saline: Saline x 5 days
MMC: Mitomycin C 1 mg/kg x 5 days

Staining;
HE; hematoxylin-eosin
γH2AX; γH2AX immunohistochemical stain

Bar represents 50 μm

Supplementary S28

Thymus

HE

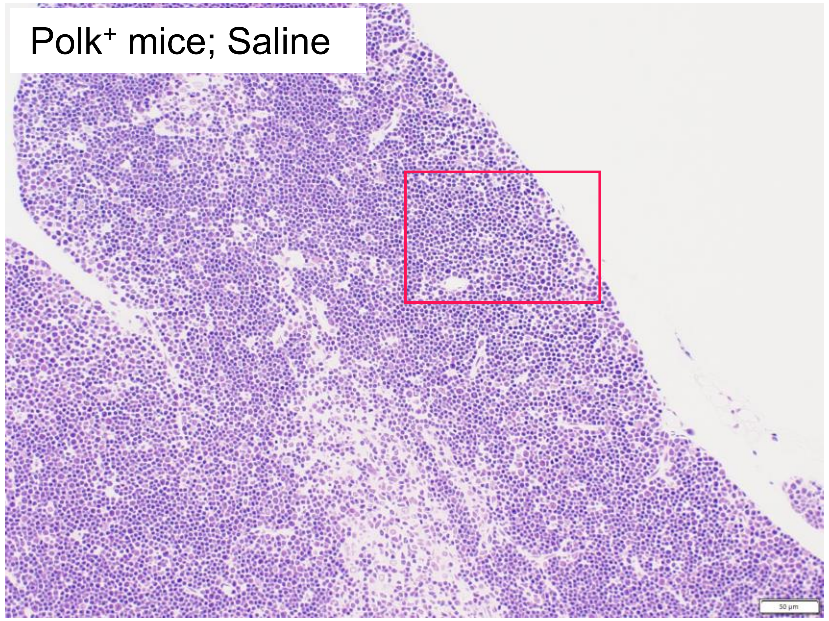

γH2AX

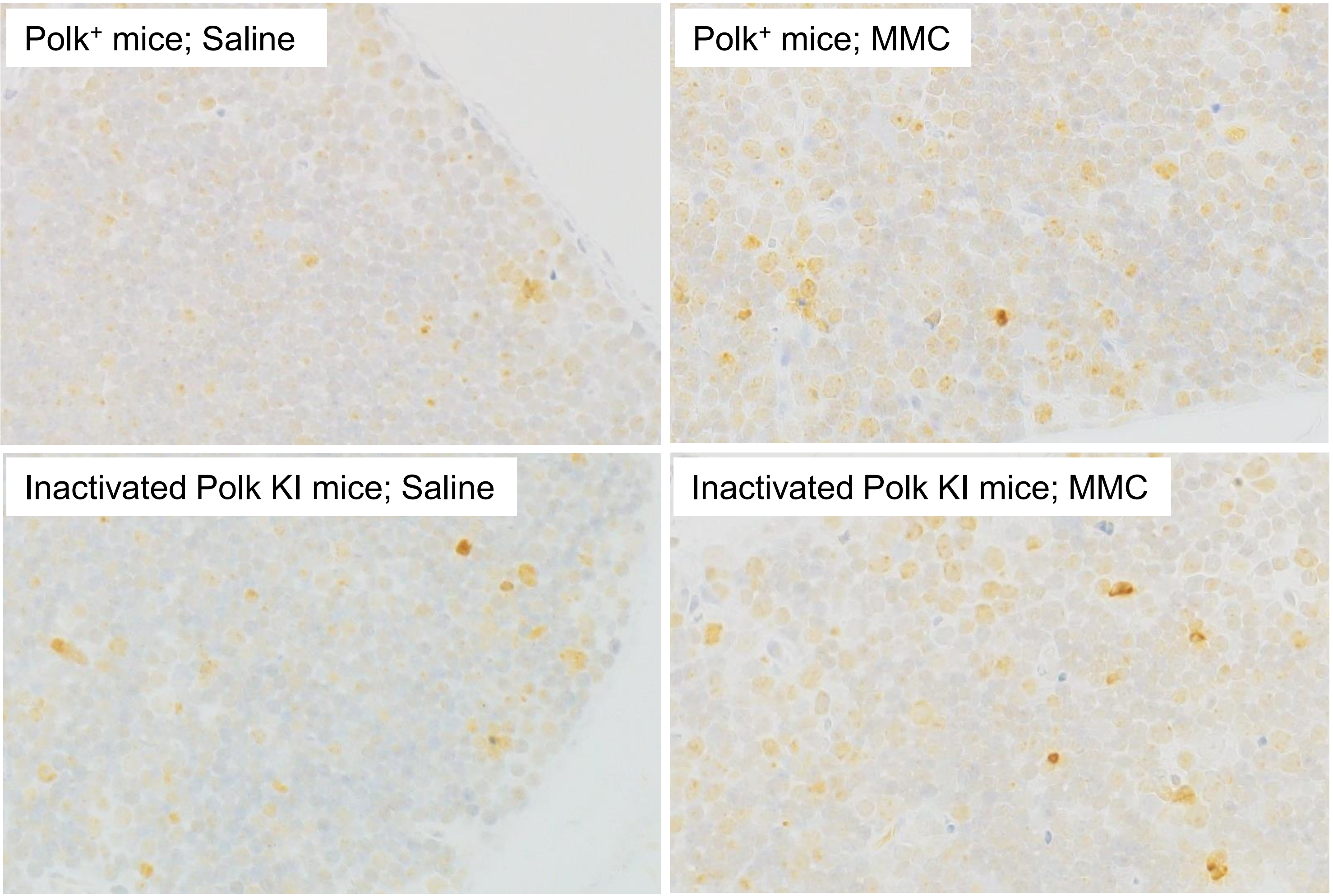

Mice;
Inactivated Polk KI mice, Polk^{+} mice

Treatment;
Saline: Saline x 5 days
MMC: Mitomycin C 1 mg/kg x 5 days

Staining;
HE; hematoxylin-eosin
γH2AX; γH2AX immunohistochemical stain

Bar represents 50 μm

Supplementary S29

Sublingual grand

HE

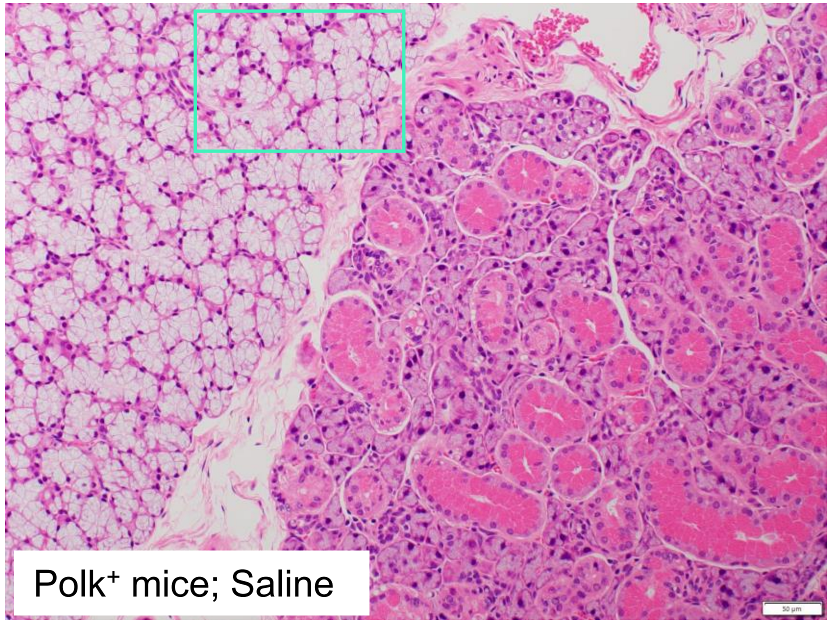

γH2AX

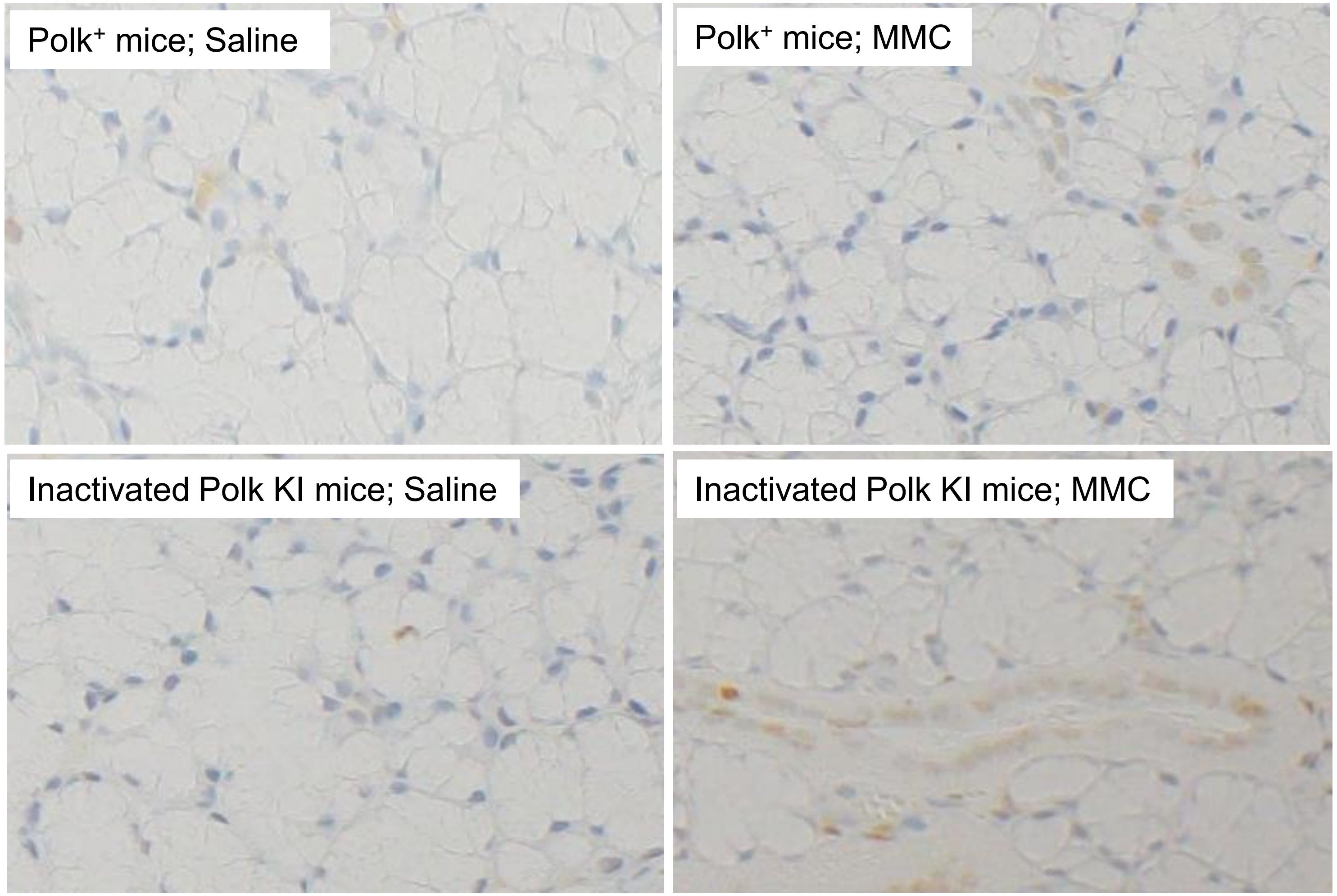

Mice;
  Inactivated Polk KI mice, Polk^{+} mice

Treatment;
  Saline: Saline x 5 days
  MMC: Mitomycin C 1 mg/kg x 5 days

Staining;
  HE; hematoxylin-eosin
  γH2AX; γH2AX immunohistochemical stain

Bar represents 50 μm

Supplementary S30

Retina

HE

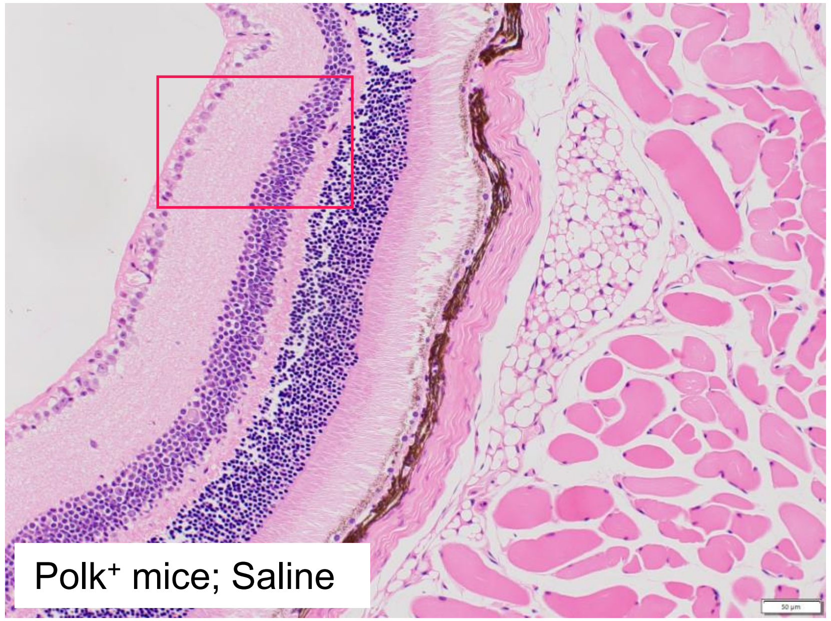

γH2AX

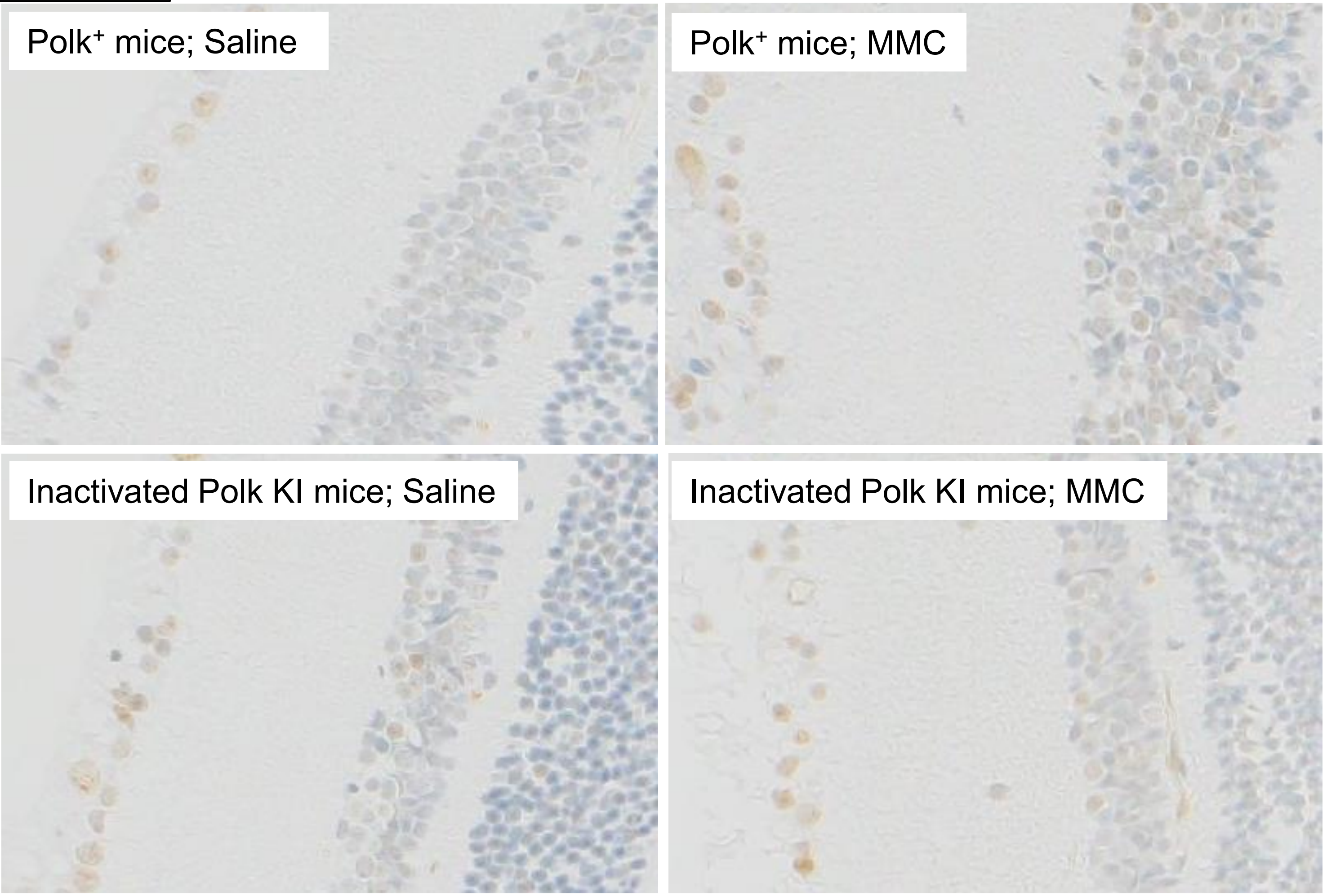

Mice;
Inactivated Polk KI mice, Polk^{+} mice

Treatment;
Saline: Saline x 5 days
MMC: Mitomycin C 1 mg/kg x 5 days

Staining;
HE; hematoxylin-eosin
γH2AX; γH2AX immunohistochemical stain

Bar represents 50 μm

Supplementary S31

Hippocampus

HE

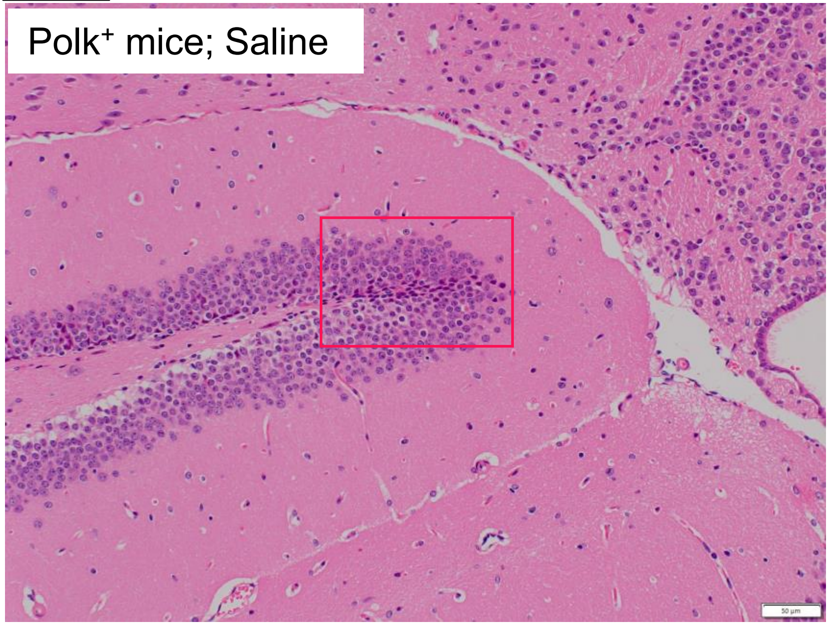

Mice;
Inactivated Polk KI mice, Polk$^+$ mice

Treatment;
Saline: Saline x 5 days
MMC: Mitomycin C 1 mg/kg x 5 days

Staining;
HE; hematoxylin-eosin
γH2AX; γH2AX immunohistochemical stain

Bar represents 50 μm

γH2AX

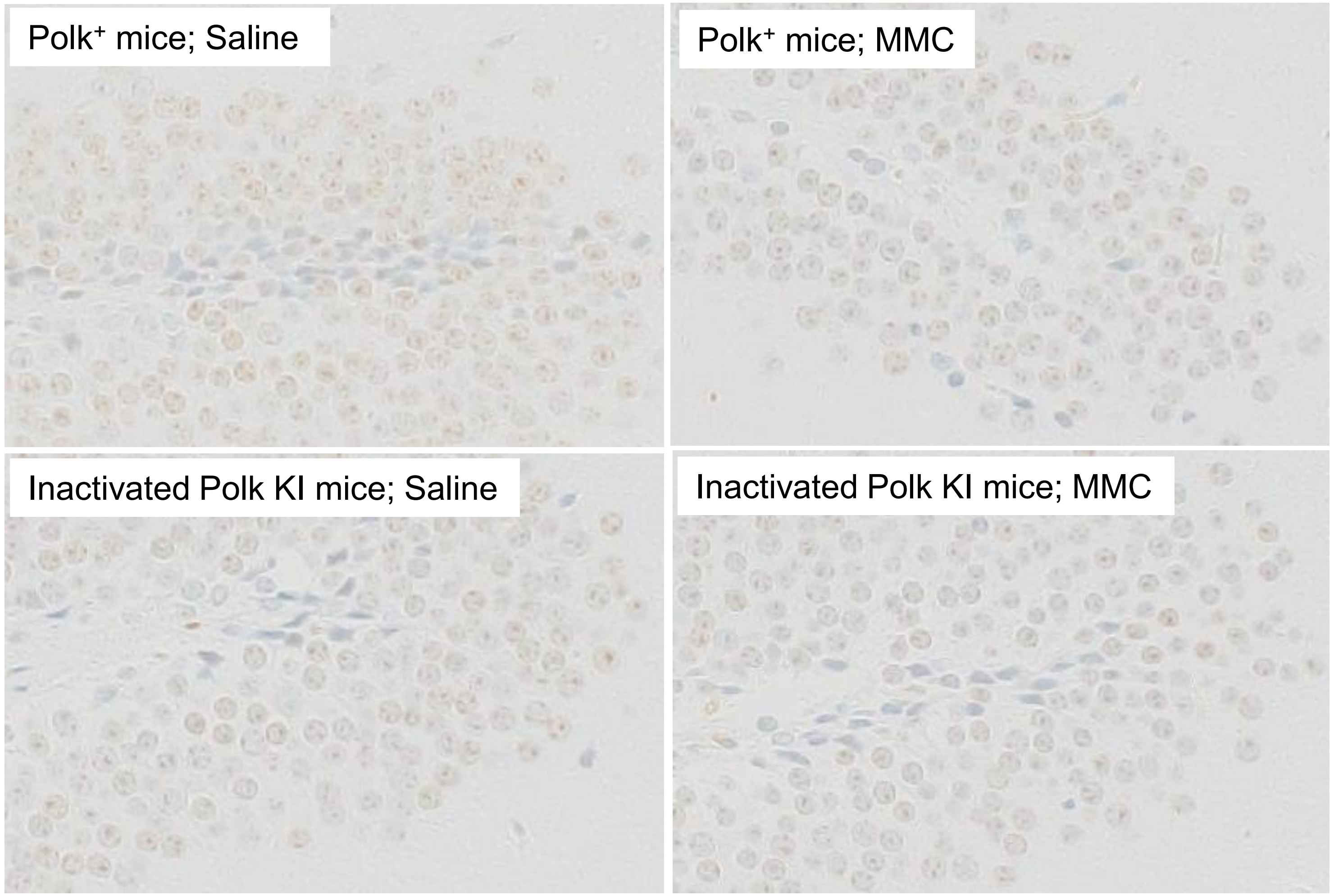

Supplementary S32

Cerebral cortex

HE

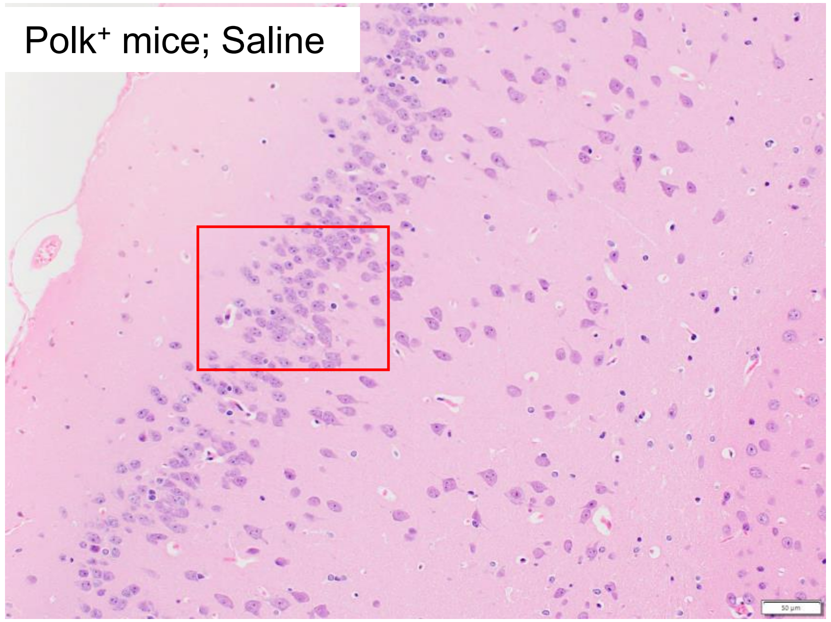

γH2AX

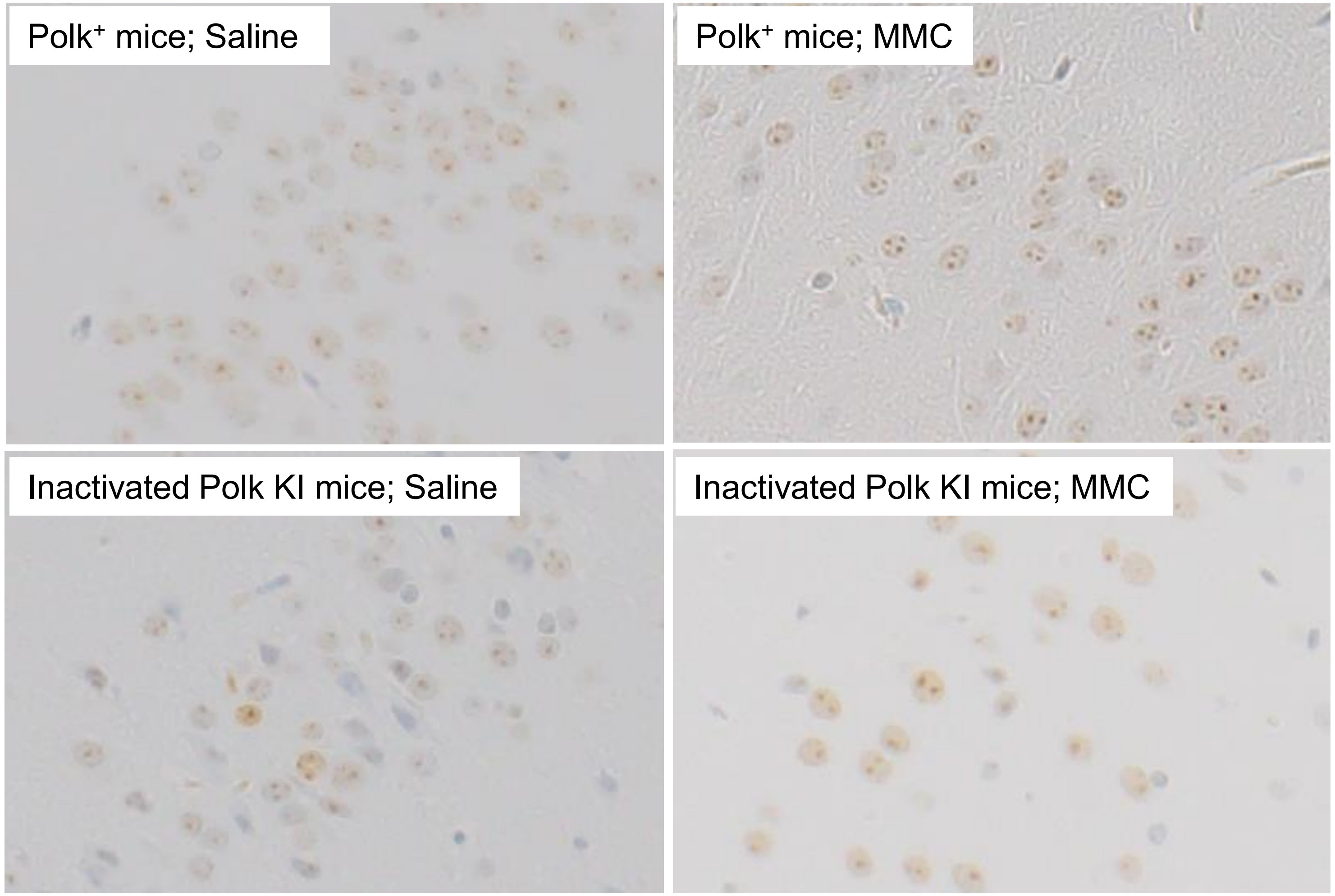

Mice;
Inactivated Polk KI mice, Polk$^+$ mice

Treatment;
Saline: Saline x 5 days
MMC: Mitomycin C 1 mg/kg x 5 days

Staining;
HE; hematoxylin-eosin
γH2AX; γH2AX immunohistochemical stain

Bar represents 50 μm

Supplementary S33

Cerebral white matter

HE

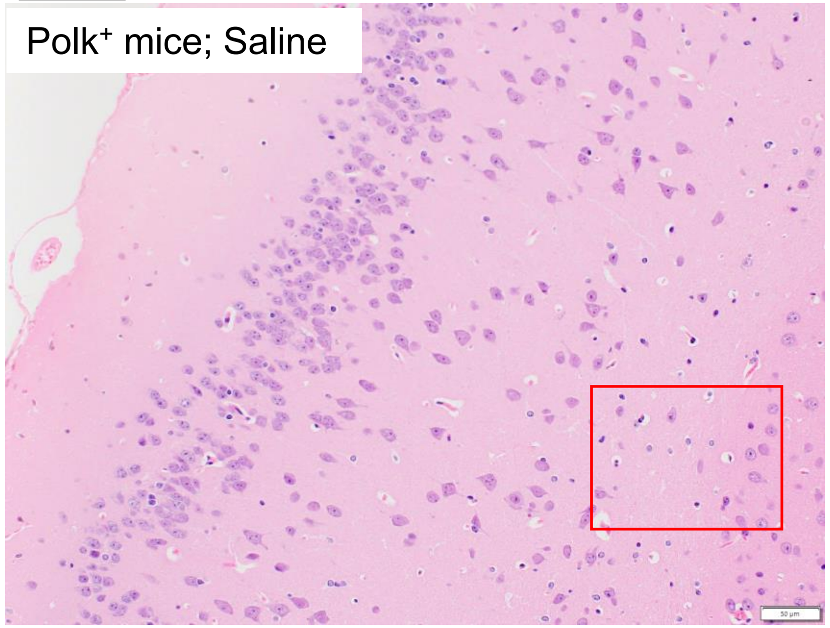

γH2AX

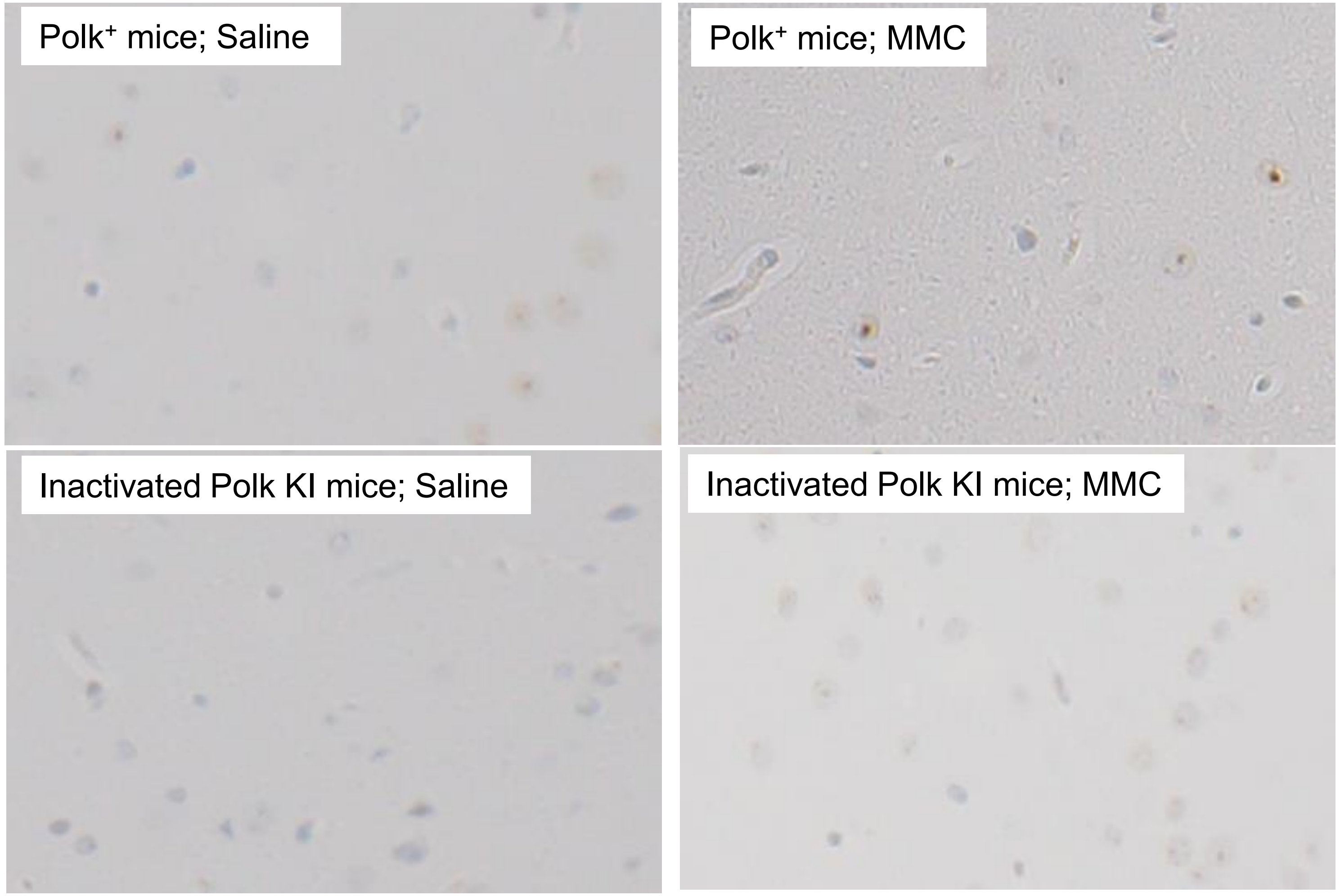

Mice;
 Inactivated Polk KI mice, Polk^{+} mice

Treatment;
 Saline: Saline x 5 days
 MMC: Mitomycin C 1 mg/kg x 5 days

Staining;
 HE; hematoxylin-eosin
 γH2AX; γH2AX immunohistochemical stain

Bar represents 50 μm

Supplementary S34

Group	Polk expression	Polk^{+} mice, Saline						Polk^{+} mice, MMC						Inactivated Polk KI mice, Saline						Inactivated Polk KI mice, MMC					
Treatmnet	RNA seq	Saline						MMC						Saline						MMC					
Dose	reference data	0 mg/kg x 5 day						1 mg/kg x 5 day						0 mg/kg x 5 day						1 mg/kg x 5 day					
Sex	(RPKM)	Male			Female			Male			Female			Male			Female			Male			Female		
Animal No.		1101	1102	1103	5101	5102	5103	1201	1202	1203	5201	5202	5203	1301	1302	1303	5301	5302	5303	1401	1402	1403	5401	5402	5403
1 Liver	0.212	0	0	0	0	0	1	2	2	2	2	2	2	0	0	0	1	2	0	4	3	4	3	3	3
2 Bladder	3.850	0	0	1	1	1	1	2	2	2	2	3	3	1	0	1	2	2	0	3	3	4	4	3	4
3 Adrenal cortex	0.459	0	0	0	0	0	1	1	4	1	2	2	2	1	0	1	3	3	3	3	3	3	3	3	4
4 Thyroid		1	1	2	0	missing	0	2	3	3	1	2	1	1	0	1	1	0	1	3	4	3	3	3	2
5 Testis (round spermatids)	3.604	0	0	0	NA	NA	NA	3	3	3	NA	NA	NA	0	0	0	NA	NA	NA	4	4	4	NA	NA	NA
6 Tongue basal epithelial		0	2	0	0	1	1	2	2	3	3	3	2	1	0	1	1	1	1	4	3	3	4	4	3
7 Glandular stomach	0.124	1	1	1	0	0	1	2	2	2	2	2	2	1	1	1	1	1	1	3	3	3	3	3	3
8 Forestomach	0.124	0	0	0	0	0	0	2	1	2	1	1	2	0	1	0	0	0	1	2	2	1	3	3	3
9 Submandibular gland		1	1	1	1	1	1	3	3	3	2	3	3	1	1	1	1	1	1	4	3	4	4	3	4
10 Esophagus		1	1	1	1	0	1	3	2	4	2	3	3	2	1	2	1	1	1	3	4	4	4	4	3
11 Mammary gland	0.609	missing	missing	missing	1	1	1	3	missing	4	3	3	3	missing	missing	missing	1	1	missing	4	4	missing	4	4	4
12 Subcutaneous vascular endothelium		0	0	0	0	0	0	4	1	1	3	2	2	1	0	0	0	0	0	3	3	1	4	3	3
13 Seminal vesicle		0	0	0	NA	NA	NA	0	2	1	NA	NA	NA	0	0	0	NA	NA	NA	1	3	1	NA	NA	NA
14 Spleen	0.301	1	2	2	2	3	2	2	1	2	2	1	2	2	2	2	2	3	2	2	2	2	3	2	3
15 Ileum	0.077	1	1	1	1	1	1	1	2	2	2	3	2	missing	1	2	1	1	1	1	2	3	3	3	4
16 Epididymis		1	2	1	NA	NA	NA	3	2	1	NA	NA	NA	1	2	1	NA	NA	NA	2	4	2	NA	NA	NA
17 Lung	0.825	0	0	0	0	0	0	1	1	2	2	2	1	0	0	0	0	2	0	2	1	2	3	2	3
18 Kidney	0.520	0	0	0	0	0	0	1	1	1	1	0	0	0	0	0	0	0	1	1	1	1	2	1	1
19 Hair follicle		1	0	0	0	0	1	1	1	2	1	1	2	1	1	0	1	0	0	2	2	1	2	2	2
20 Cornea		1	2	2	1	2	1	4	2	3	2	2	0	2	3	2	1	0	4	4	2	2	2	3	3
21 Epidermis		0	0	0	1	1	1	2	1	2	2	1	2	1	1	1	1	1	1	2	1	1	3	3	3
22 Colon	0.384	2	2	2	1	2	1	3	3	3	3	3	3	2	2	1	1	1	2	3	2	3	4	4	4
23 Tongue skeletal muscle		0	0	0	0	1	1	2	2	2	2	2	2	1	0	0	1	0	0	2	2	2	3	3	2
24 Heart	0.357	0	0	0	0	0	0	0	1	1	0	0	0	0	0	0	0	0	2	2	1	1	0	0	0
25 Trachea epithelium		2	2	2	1	1	2	3	4	3	4	3	3	missing	2	3	1	missing	1	4	4	3	3	4	4
26 Harderian gland		1	1	2	2	2	2	3	3	3	2	3	2	2	1	3	1	1	3	3	3	2	4	1	4
27 Pancreas		1	2	2	2	1	1	2	1	2	3	2	2	1	1	1	1	1	2	2	2	3	2	2	2
28 Brainstem		2	2	3	1	2	3	3	3	2	2	2	2	2	1	2	3	2	2	3	3	3	2	2	2
29 Testis (whole cells)	3.604	4	4	4	NA	NA	NA	4	4	4	NA	NA	NA	4	4	4	NA	NA	NA	4	4	4	NA	NA	NA
30 Mesenteric lymph node		1	1	1	1	1	1	2	3	2	2	2	2	1	1	1	1	1	1	2	2	2	2	2	3
31 Prostate		0	1	1	NA	NA	NA	3	2	2	NA	NA	NA	2	1	1	NA	NA	NA	3	2	2	NA	NA	NA
32 Cerebellum	1.710	1	1	1	0	1	1	2	2	2	1	2	2	1	1	1	0	0	1	2	2	2	2	1	2
33 Thymus	0.693	2	2	2	2	2	2	2	3	3	3	2	3	2	2	1	3	3	3	2	2	3	2	3	3
34 Sublingual gland		0	0	0	0	0	0	2	1	2	1	1	1	0	0	0	0	0	0	2	0	1	2	1	1
35 Retina		3	2	3	2	3	3	4	4	4	4	4	4	4	4	4	3	3	3	4	3	3	4	4	4
36 Hippocampus		4	4	4	2	2	4	4	missing	missing	4	missing	4	missing	4	4	3	3	missing	4	2	missing	4	4	missing
37 Cerebral cortex	1.443	3	3	3	2	2	2	4	3	4	3	4	3	3	3	3	2	3	3	4	3	2	4	2	3
38 Cerebral white matter		2	2	2	0	0	1	3	2	2	3	2	2	2	2	1	2	1	1	2	2	0	2	1	2

Polk expression data ;
https://www.ncbi.nlm.nih.gov/gene/27015/?report=expression

Supplementary S35

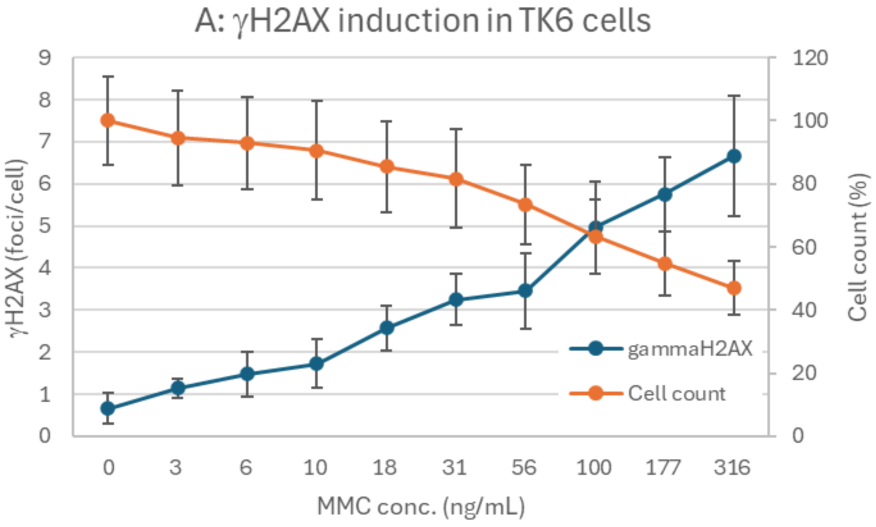

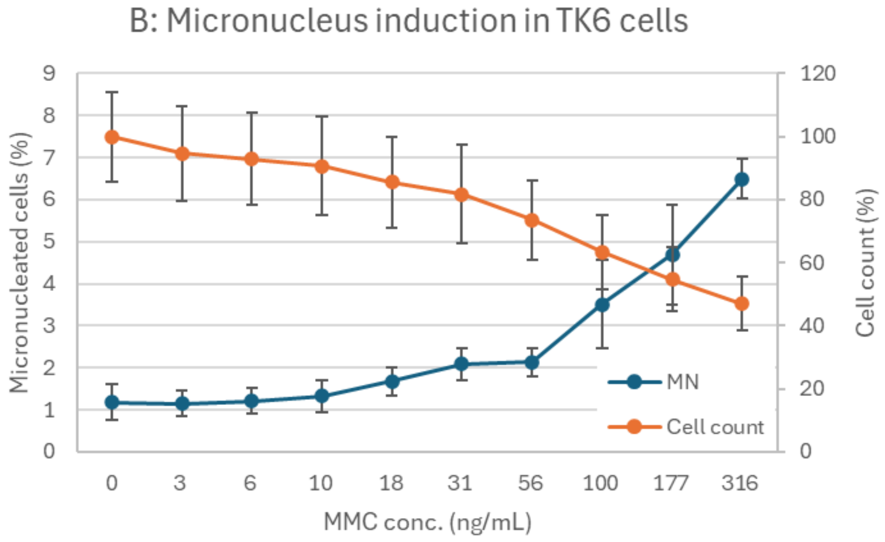

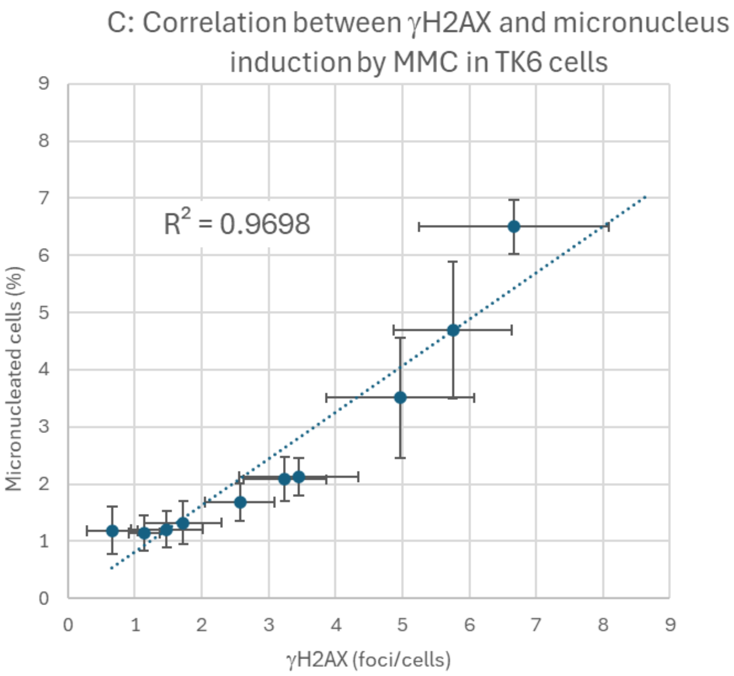

Mitomycin C (MMC) -induced γH2AX foci were highly correlated with micronucleated (MN) cell induction which were caused by DNA double strand breaks. TK6 cells were treated with MMC for 26h. γH2AX foci per cells (A) and incidence of micronucleated (MN) cells (B) were measured. Reduction of cell counts from the vehicle control are shown as a cytotoxicity indicator. Correlation of γH2AX foci and MN induction is shown (C).
Each point and bar represents mean and SD of 6 wells from 2 independent experiments. The experiments were conducted with the method previously described (Takeiri et al., Genes Environ, 2019;41:4).
